# α‐Amino‐*iso*‐Butyric Acid Foldamers Terminated with Rhodium(I) N‐Heterocyclic Carbene Catalysts

**DOI:** 10.1002/chem.202104293

**Published:** 2022-01-12

**Authors:** David P. Tilly, William Cullen, Heng Zhong, Romain Jamagne, Inigo Vitórica‐Yrezábal, Simon J. Webb

**Affiliations:** ^1^ Manchester Institute of Biotechnology University of Manchester 131 Princess St Manchester M1 7DN UK; ^2^ Department of Chemistry University of Manchester Oxford Road Manchester M13 9PL UK

**Keywords:** carbene ligands, conformation analysis, foldamers, homogeneous catalysis, rhodium

## Abstract

To investigate how remotely induced changes in ligand folding might affect catalysis by organometallic complexes, dynamic α‐amino‐*iso*‐butyric acid (Aib) peptide foldamers bearing rhodium(I) N‐heterocyclic carbene (NHC) complexes have been synthesized and studied. X‐ray crystallography of a foldamer with an N‐terminal azide and a C‐terminal Rh(NHC)(Cl)(diene) complex showed a racemate with a chiral axis in the Rh(NHC) complex and a distorted 3_10_ helical body. Replacing the azide with either one or two chiral L‐α‐methylvaline (L‐αMeVal) residues gave diastereoisomeric foldamers that each possessed point, helical and axial chirality. NMR spectroscopy revealed an unequal ratio of diastereoisomers for some foldamers, indicating that the chiral conformational preference of the N‐terminal residue(s) was relayed down the 1 nm helical body to the axially chiral Rh(NHC) complex. Although the remote chiral residue(s) did not affect the stereoselectivity of hydrosilylation reactions catalysed by these foldamers, these studies suggest a potential pathway towards remote conformational control of organometallic catalysts.

## Graphical abstract

**Another twist**: α‐Amino‐*iso*‐butyric acid foldamers terminated with rhodium(I) N‐heterocyclic carbene complexes have been shown to possess point, helical and axial chirality. Communication from chiral residues at the N‐termini of these 1 nm long foldamers to rhodium(I) complexes at the C‐termini led to unequal ratios of diastereoisomers, although this did not alter the stereoselectivity of hydrosilylation reactions catalyzed by these foldamers.

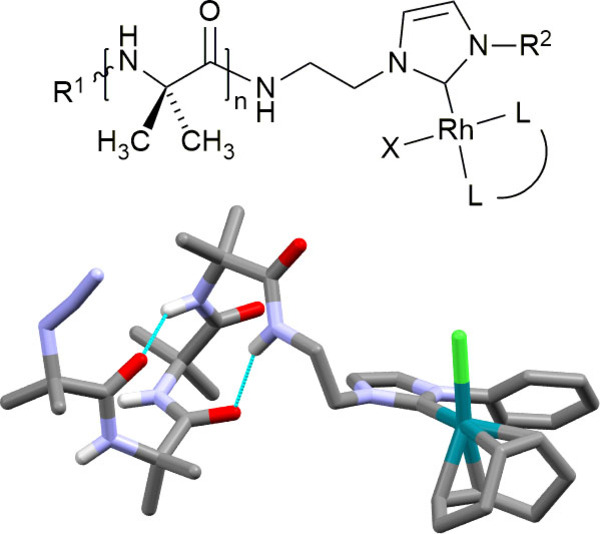

## Introduction

Metal complexes located within a folded polypeptide structure give many proteins their activity, such as haem embedded within haemoglobin. The folding of polypeptides can be mimicked using foldamers, synthetic oligomers that adopt well‐defined “folded” conformations. In recent years foldamers have been shown to have many applications, including as sensors, devices and catalysts.^[1]^ Within this latter area, several classes of foldamers, including protein‐foldamer hybrids,[^2^, ^3^, ^4^] have been shown to have catalytic activity that is either dependent upon or modulated by their folded structure.[^5^, ^6^, ^7^, ^8^, ^9^, ^10^, ^11^] Metal complexes, integrated within or pendant to a foldamer, can act as catalysts^[11]^ or provide essential structural motifs,[^12^, ^13^] including responsive centres that can alter the folded state^[14]^ or reporters of stereochemical complexity.[^15^, ^16^, ^17^] A diverse set of reactions has been catalysed by foldamers bearing metal complexes, such as hydrolysis,^[11]^ allylation,^[18]^ hydrogenation[^19^, ^20^] and hydrosilylation.[^21^, ^22^]

Foldamer catalysis also depends on foldamer conformation, which can be either static or dynamic at room temperature; dynamic foldamers rapidly interconvert between a handful of similarly stable conformations.[^23^, ^24^, ^25^] Foldamers largely composed of α‐amino‐*iso*‐butyric acid (Aib) are an important class of dynamic foldamer that have recently come to the fore due to their ability to relay information from a remote site to a reporting group. Aib foldamers with at least four Aib residues form 3_10_ helices that are stabilised by intramolecular hydrogen bonding.[^26^, ^27^] In solution, these foldamers rapidly interconvert between helices of right‐ (*P*) or left‐ (*M*) handed screw sense, which are present in an equal ratio in the absence of any chiral residues. However, the relative populations of *M* and *P* helix can be altered by attaching a “chiral controller” either covalently[^28^, ^29^, ^30^, ^31^, ^32^, ^33^] or non‐covalently[^34^, ^35^, ^36^, ^37^] to a terminus. The screw sense preference induced by a chiral controller can be quantified by calculating the helical excess (*h.e*., defined as ([*P*]−[*M*])/([*P*]+[*M*])).^[38]^ The incorporation of a spectroscopic “reporter” allows *h.e*. to be measured,[^39^, ^40^, ^41^, ^42^, ^43^] with a chiral reporter often used to identify the major screw sense. A chiral controller and a chiral reporter at opposite termini produce diastereoisomeric foldamers that often appear pseudo‐enantiomeric, for example, dominated by the helical preference of an N‐terminal chiral controller. A good example is ^13^C labelling of one methyl on an Aib, which produces a residue with negligible helical preference.^[33]^ However, when the groups at the termini both have a strong helical preference (for example, α‐methylvaline, αMeVal) then a region where the 3_10_ helix inverts its sense (a “tendril perversion”) can be identified.^[44]^ Instead of point chirality, as in αMeVal, other types of chirality can be incorporated. A biaryl axis with restricted rotation is one such example, where the observation of either diastereoisomers or apparent enantiomers depends upon the rate of rotation around this axis.[^45^, ^46^]

Attachment to a conformationally dynamic Aib foldamer could potentially alter the activity and selectivity of a catalytic organometallic complex. The Aib foldamer would provide a geometrically defined environment around the metal complex, one that can be made responsive to external stimuli, for example to light or to ligands binding to remote allosteric sites.[^28^, ^34^, ^35^, ^36^, ^37^] Rhodium(I) N‐heterocyclic carbenes (NHCs) are an attractive choice of catalytic organometallic complex, as they are often diamagnetic, air stable and known to catalyse a variety of reactions under mild conditions, such as the hydrosilylation of alkenes, alkynes, ketones and aldehydes.^[47]^ The dynamic behaviour of Aib foldamers may permit remote conformational changes to alter catalytic efficiency and the stereochemical outcome of catalysed reactions, similar to allosteric control in some metalloenzymes.[^48^, ^49^] Although catalytic hydrolysis and organocatalysis by functionalised Aib foldamers have been described,[^6^, ^11^] there are not yet any examples of organometallic catalysis by this class of dynamic foldamer. Herein we describe the synthesis of a family of Aib foldamers with a single rhodium(I) NHC complex at the C‐terminus, as well as their conformational and catalytic behavior.

## Results and Discussion

### Aib foldamers with Rh(NHC) at the C‐terminus.

**Figure 1:**
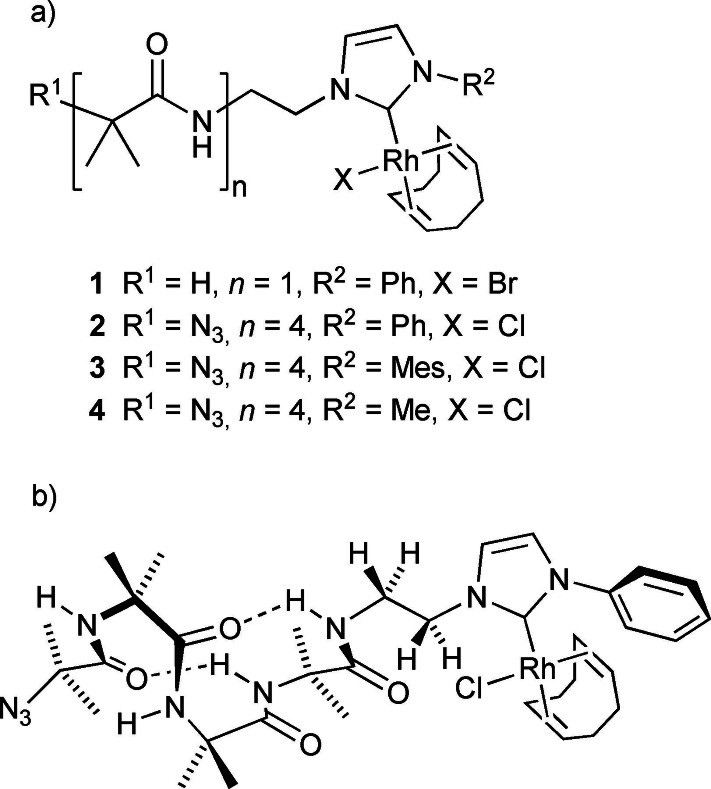
a) Foldamer rhodium‐NHC complexes **1**–**4**. b) Compound **2** shown folded into a 3_10_ helix.

An ethylene bridge was selected to attach the Rh(NHC) complex to the C‐terminus of the foldamer body (Figure 1a). It was hoped that this relatively short bridge combined with attachment to an imidazole nitrogen (instead of to C4 or C5) would bring the rhodium centre and Aib helix into proximity and allow the folded environment to affect catalysis by the rhodium(I) centre. Tetrameric foldamers were employed as this length is readily available through chemical synthesis and is the shortest length able to fold into a 3_10_ helix (Figure 1b). In the first series of foldamer complexes, **2**, **3** and **4**, the N‐terminus was a simple azide group and the substituent on the remaining NHC nitrogen was either methyl, phenyl or 2,4,6‐trimethylphenyl (mesityl, Mes). To provide an unfolded control compound, the foldamer was replaced with an *iso*‐butyric acid terminus (complex **1**). The remaining coordination sites on the rhodium(I) centre were occupied by a halide (Cl^−^ or Br^−^) and 1,5‐cyclooctadiene (COD).

**Scheme 1:**
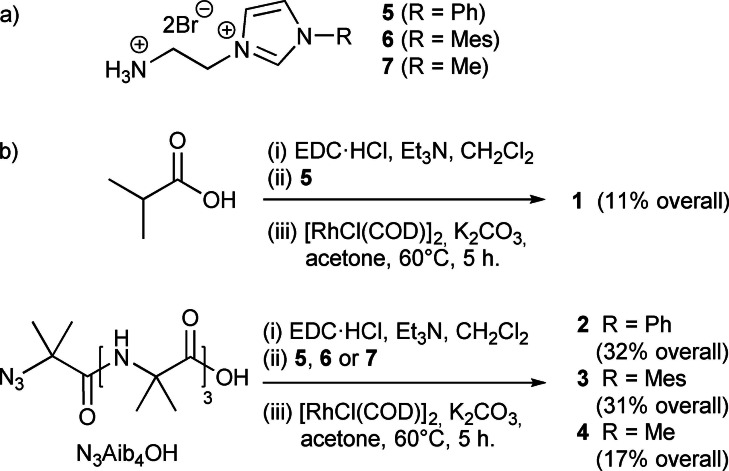
a) Imidazolium precursors **5**–**7**. b) Synthesis of control compound **1** and metal‐complexing foldamers **2**, **3** and **4**.

Control compound **1** was synthesised by *N*‐(3‐dimethylaminopropyl)‐*N*’‐ethylcarbodiimide (EDC)‐mediated condensation of *iso*‐butyric acid with the NHC precursor [NH_3_(CH_2_)_2_(Im−Ph)]^2+^ ⋅ 2Br^−^ **5** (Scheme 1). Complexation to rhodium(I) was performed by deprotonation of the imidazolium (Im−R) to give the NHC and metalation, following a modification of the procedures of Savka and Plenio (Scheme 1b).^[50]^ Treatment with K_2_CO_3_ in acetone at 60 °C in the presence of [RhCl(COD)]_2_ gave the product **1** as a yellow solid after preparative thin layer chromatography; the analogue with chloride in the place of bromide was also obtained but in lower yield.

Tetrameric Aib foldamer N_3_Aib_4_OH with an achiral azido group at the N‐terminus was synthesized using published conditions.[^38a^, ^40^, ^51^] This Aib tetramer was then ligated to the NHC precursors **5**, [NH_3_(CH_2_)_2_(Im‐Mes)]^2+^ ⋅ 2Br^−^ **6** and [NH_3_(CH_2_)_2_(Im−Me)]^2+^ ⋅ 2Br^−^ **7** respectively to give the imidazolium foldamers **2**, **3** and **4**. The rhodium was introduced using the same conditions used to make **1**. An alternative method, transmetallation of an intermediate silver(I) complex, was unsuccessful,^[52]^ which we attribute to cross‐reactivity between the silver NHC complex and the azide.^[53]^

**Figure 2:**
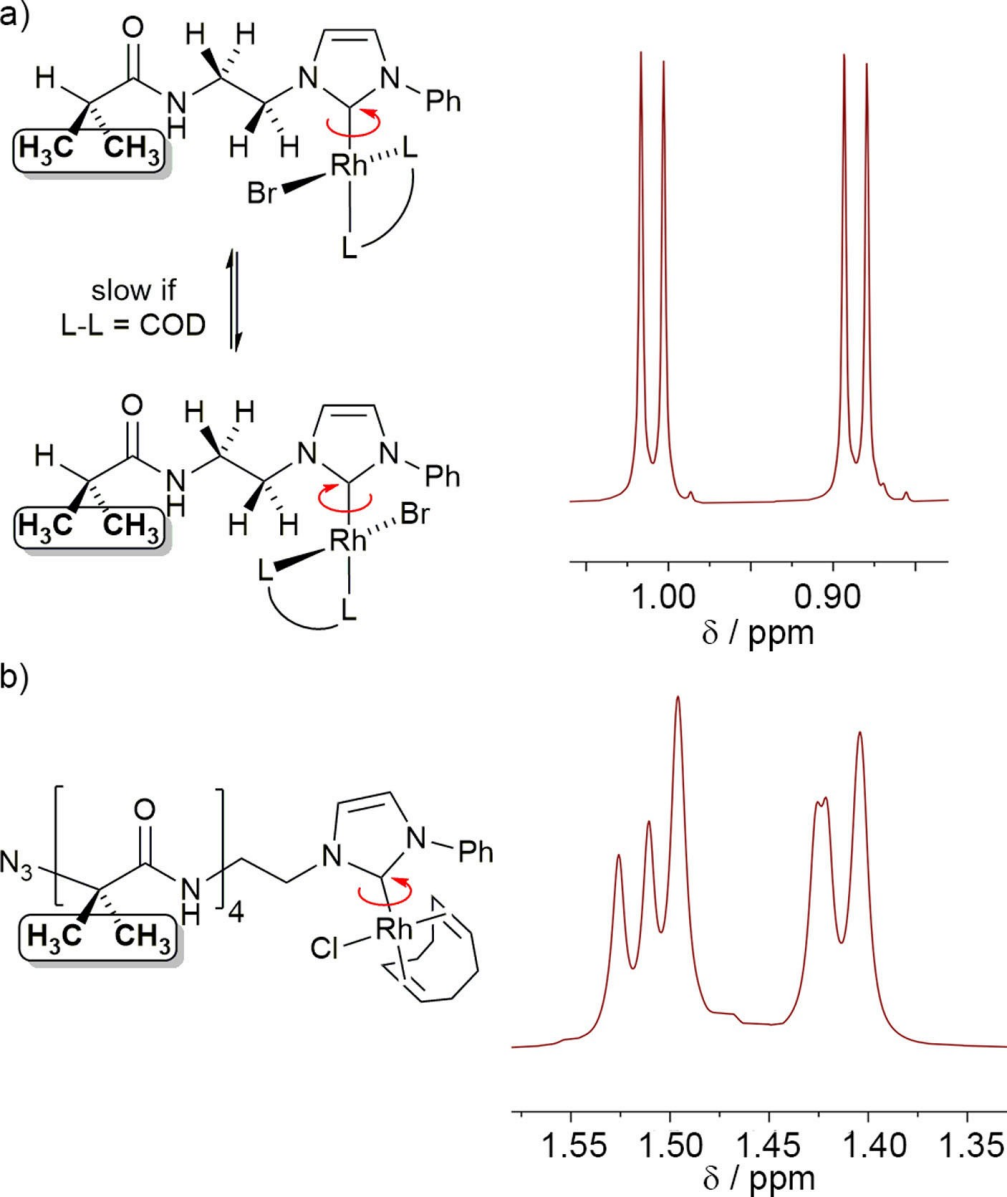
a) Enantiomers of **1** that arise from slow rotation around the NHC−Rh bond and corresponding partial NMR spectrum that shows anisotropic methyl resonances (each split by coupling to H). b) Partial NMR spectrum of **2** showing anisotropic methyl resonances (six of the eight Aib methyl resonances are resolved).

^1^H NMR spectroscopy of [(^*i*^PrC(O)NH(CH_2_)_2_‐NHC−Ph)Rh(Br)(COD)] **1** shows splitting of the methyl group resonances of the *iso*‐butyl group into anisochronous signals (Figure 2a). This splitting suggests that the Rh(NHC) complex is stereogenic and producing a racemic mixture. This also results in diastereotopic protons for the methylenes of the bridge between the NHC and the *iso*‐butylamide, which give increasingly anisotropic resonances the closer they are to the stereogenic Rh‐NHC centre; ^1^H−^13^C HSQC spectroscopy showed pairs of resonances at 5.47/4.30 ppm and 4.17/3.77 ppm for the methylenes closest to and furthest from the NHC respectively.

The stereogenic motif is proposed to be an axis of chirality that arises from restricted rotation around the Rh−C(NHC) bond (Figure 2a). The solid‐state structure of similar complexes, such as [(2,6‐di(^*i*^Pr)phenyl‐NHC‐(CH_2_)_3_Si(O^*i*^Pr)_3_)Rh(Cl)(COD)],^[54]^ shows that the Rh−Cl bond is oriented perpendicular to the plane of the NHC; the bis(alkene) ligand occupies the remaining two positions.^[55]^ If the NHC is not symmetrically substituted and Rh−C(NHC) rotation is slow, then a chiral axis is created.

The introduction of a slowly interconverting chiral axis is also consistent with the ^1^H and ^13^C NMR spectra of **2**–**4**. For example, the rhodium complex in **2** shows diastereotopic Aib methyl groups, six of which are resolved (Figure 2b). VT ^1^H NMR spectroscopy on **2** from −20 to 80 °C in CDCl_2_CDCl_2_ showed small changes in the chemical shift of some resonances and spectral sharpening, but no coalescence of these diastereotopic signals. The methylenes in the bridge between the NHC and the Aib foldamer body are also diastereotopic and these resonances are once again increasingly anisotropic the closer they are to the Rh‐NHC centre. The four resonances are found at 5.08/4.72 ppm and 4.02/3.70 ppm for the methylenes closest to and furthest from the NHC respectively. In the ^13^C NMR spectrum of **2**, a doublet at 182.2 ppm (^1^ *J* Rh−C=51.0 Hz) is consistent with coordination of the NHC to the rhodium(I) center.^[52]^

**Figure 3:**
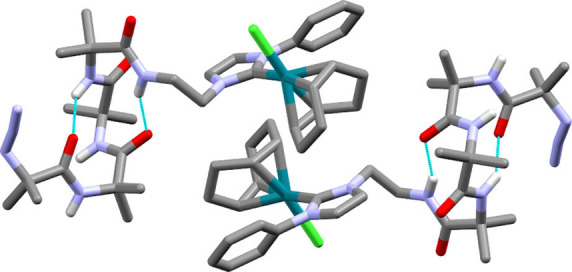
Solid state structure showing the enantiomeric forms of **2** with intramolecular hydrogen bonds shown. C atoms are shown in grey, N in light blue, O in red, Cl in green and Rh in teal. Some H atoms have been removed and acetonitrile of solvation not shown for clarity.

Interactions between the rhodium complex and the helical foldamer body are revealed in the crystal structure of **2** (Figure 3). Crystals suitable for X‐ray crystallographic structure determination were obtained by slow evaporation from acetonitrile. The solid state structure shows two intramolecular hydrogen bonds: one between the C=O of the first Aib and the NH of the fourth Aib and the other between the C=O of the second Aib and the NH of the ethylene bridge. This *i*→*i*+3 hydrogen bonding pattern creates a distorted 3_10_ helical structure. The geometry around the rhodium(I) centre is square planar with a coordinated chloride; the Rh to C(NHC) bond length is 2.020(4) Å and that of Rh to Cl is 2.4430(8) Å, both of which are similar to those in a comparable complex (Rh−C(NHC) 2.033(7) Å, Rh−Cl 2.4044(16) Å).^[56]^ Unlike the reported structure of [(BocNH(CH_2_)_2_‐NHC−Me)Rh(Cl)(NBD)],^[57]^ no hydrogen bond was observed between the chloride on rhodium and the C‐terminal NH, which is hydrogen bonded back into the foldamer body. The chloride is instead intermolecularly hydrogen bonded to the NH of the third Aib of a neighbouring foldamer (see Figure S8, Supporting Information). The COD ligand on rhodium appears to be a steric barrier that forces the methylene groups of ethylene bridge to the other side of the NHC (Figure 3), perhaps then influencing the helical sense of the Aib foldamer body. Both enantiomers of the chiral axis are present in the unit cell, with an *S_a_*‐chiral axis in the rhodium complex co‐existing with a *P* helix in the foldamer body and *vice versa* when an *R_a_*‐chiral axis is present in the rhodium complex (Figure 3).^[55a]^

The steric congestion around the rhodium(I) centre can be modulated by changing the substituents on the NHC or ligands on rhodium. Increasing the steric requirements of the NHC by exchanging phenyl for mesityl, giving [(N_3_Aib_4_NH(CH_2_)_2_‐NHC‐Mes)Rh(Cl)(COD)] **3**, led to increased anisotropy of protons in the CH_2_ groups of the bridge; the pairs of resonances are at 5.42/4.42 ppm and 4.09/3.66 ppm for the methylenes closest to and furthest from the NHC respectively. The methyl groups on the mesityl are found at three distinct positions, 2.39, 2.34 and 1.82 ppm, with the splitting of the *ortho* methyl resonances consistent with axial chirality in the Rh complex. Much like **2**, VT ^1^H NMR spectroscopy on this compound from −20 to 80 °C showed only small changes in the chemical shift of some resonances and a sharpening of the spectra. Although [(N_3_Aib_4_NH(CH_2_)_2_‐NHC−Me)Rh(Cl)(COD)] **4** has less steric demand around the rhodium(I) than **2** or **3**, it nonetheless also shows very restricted rotation; no clear coalescence was observed during VT ^1^H NMR spectroscopy from −20 to 80 °C.

**Figure 4:**
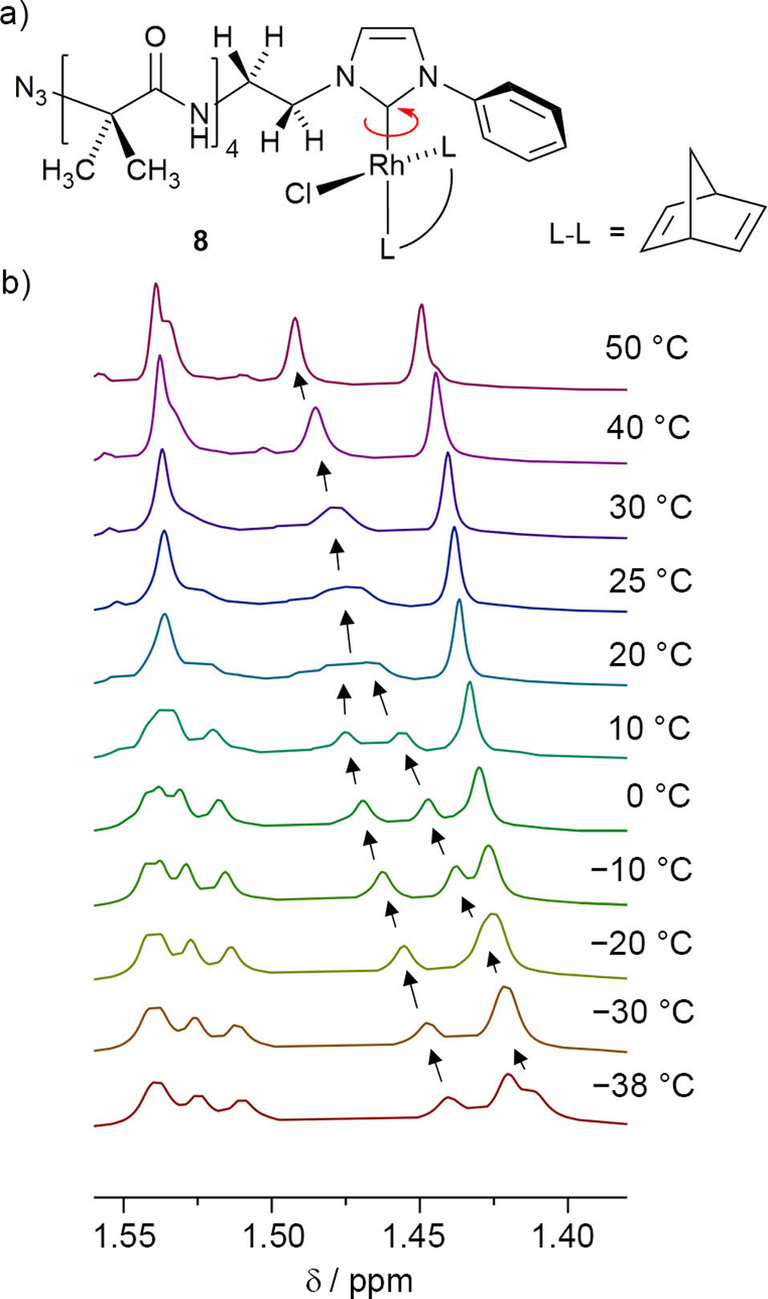
a) The structure of **8** showing the diastereotopic protons on the ethylene bridge and the diastereotopic methyl groups on the Aib residues. b) Partial ^1^H NMR spectra of foldamer **8** upon heating from −38 to 50 °C showing the resonances assigned to the Aib methyl groups.

Decreasing the steric demand of the bis(alkene) on the NHC can increase the rate of rotation around the Rh‐NHC axis, with the rotation barriers of norbornadiene (NBD) complexes measurable by ^1^H VT NMR spectroscopy^[58]^ unlike the COD complexes.^[59]^ A series of [(BocNHCH_2_CH_2_‐NHC−R)Rh(Cl)(NBD)] complexes are reported to have metal‐carbene bond rotational barriers between 55 and 59 kJ mol^−1^,^[58]^ and Δ*G* for Rh‐carbene rotation in [(R−NHC−Bn)Rh(Cl)(NBD)] complexes was measured as 57 to 63 kJ mol^−1^.^[60]^ An analogue of **2** was synthesised using [Rh(NBD)Cl]_2_ in the place of [Rh(COD)Cl]_2_. The resulting NBD complex, [(N_3_Aib_4_NH(CH_2_)_2_‐NHC−Ph)Rh(Cl)(NBD)] **8** (10 % overall yield) showed significant changes in the appearance of the ^1^H NMR spectrum at room temperature, with broader resonances, for example from the Aib methyl groups in the foldamer body (Figure 4). Variable temperature NMR spectroscopy was carried out on compound **8** from −38 to +50 °C in CDCl_3_, which caused these closely spaced Aib methyl resonances to move, broaden, then coalesce between 20° and 30 °C. At −38 °C, six resonances corresponding to the eight methyl groups are visible in the region 1.40 to 1.55 ppm, whereas at +50 °C only four methyl resonances are visible. These observations are commensurate with slow rotation around the Rh−C(NHC) bond at low temperatures providing a racemate with enantiomers not in exchange on the ^1^H NMR spectroscopy timescale. However, for NBD ligands these enantiomers interconvert rapidly at high temperatures, which in combination with rapid interchange of helical conformations in the dynamic Aib body^[61]^ produces averaging of the Aib methyl resonances and apparently isotropic signals.

Two resolved methyl resonances, at 1.44 and 1.41 ppm at −38 °C, were observed to coalesce to a single signal at 25 °C, with the resulting peak correlating to a single ^13^C NMR resonance in the HSQC spectrum. Although slow exchange of the resonances might not have been achieved at the lower temperature limit of CDCl_3_, estimation of Δ*ν* (12 Hz) and use of the Gutowsky‐Holm approximation^[62]^ gave an approximate Rh−C(NHC) rotational barrier (*E*_a_) of 65 kJ mol^−1^ for **8**.

### Aib foldamers with N‐terminal chiral residues and Rh(NHC) at the C‐terminus.

The attachment of a stereogenic centre to the N‐terminus of these foldamers, in addition to the stereogenic Rh−C(NHC) axis at the C‐terminus, should result in the formation of a pair of diastereoisomers. The terminal groups of the diastereoisomers will either induce the same screw‐sense preference (that is, either both *M* or both *P*, which gives a screw‐sense “match”) or induce opposing screw‐sense preferences (that is, one is *M* and the other *P*, which gives a screw‐sense “mismatch”); a “mismatch” can induce a “tendril perversion”.^[44]^ The ratio between the diastereoisomers will depend upon the energetic penalty for a screw‐sense “mismatch” between the screw‐sense preferences of the terminal groups compared to a screw‐sense “match”. A conceptually similar system was reported by Yashima and co‐workers, where linking a dynamic chiral metal centre to chiral 3_10_ helical foldamers gave diastereoisomers.^[15]^ Chirality was transferred from L‐Val residues down three 3_10_ helical peptide chains to a chiral yet labile Fe(II)(2,2’‐bipyridyl)_3_ centre, a complex that exhibits dynamic metal‐centred chirality that is on slow exchange on the ^1^H NMR spectroscopy timescale. In this case, the energetic penalty for a screw‐sense “mismatch” increased with increasing distance between the chiral residue and the metal complex, with the diastereoisomeric excess (*d.e*.) of the equilibrated mixture increasing from 0.3 % to 76 % as the foldamer portions were extended from two to six residues.

**Scheme 2:**
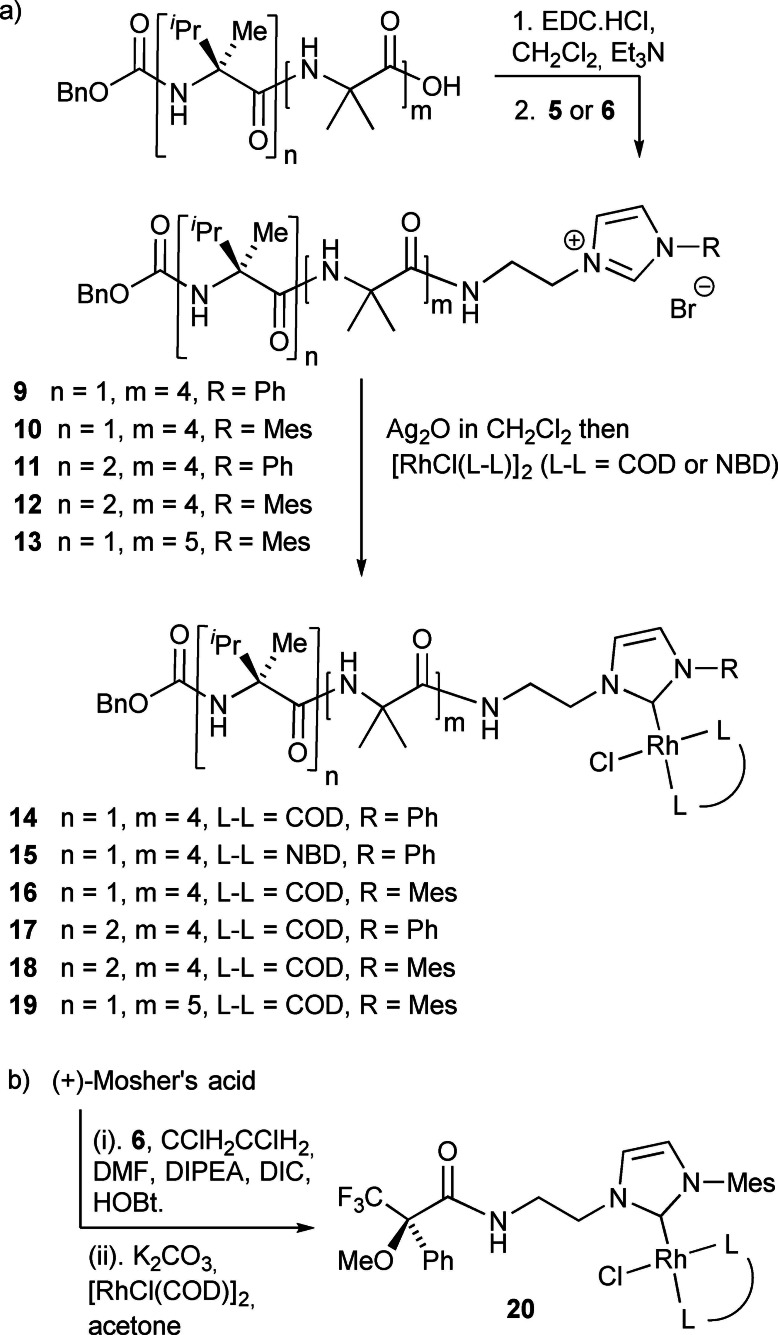
a) Synthesis of chiral Aib foldamers **14**–**19** bearing rhodium(I) NHC complexes at their C‐termini. b) Synthesis of chiral rhodium(I) NHC complex **20**.

Chiral residues with differing abilities to induce a helical excess (*h.e*.) were introduced at the N‐terminus to give compounds that combine point, helical and axial chirality (Scheme 2). An N‐terminal Cbz(L‐αMeVal) cap is known to induce predominately a right‐handed (*P*) 3_10_‐helix, with an *h.e*. of +52 % (*P : M*=76 : 24 %).^[32b]^ A [Cbz(L‐αMeVal)]_2_ cap at the N‐terminus still induces a *P* 3_10_ helix in the foldamer body but gives better control over the screw‐sense preference, providing an *h.e*. of +72 % (*P : M*=86 : 14 %).

As expected from other Aib foldamers bearing chiral N‐terminal groups,[^30^, ^32^, ^40^, ^41^] the ^1^H NMR spectra of precursors **9–13** showed anisotropic Aib methyl groups, as well as anisotropic methylene resonances in the ethylene linker to the NHC. Complexation of the rhodium centre was then performed by first forming the silver complex, by modification of the procedures of Wang and Lin,^[63]^ then transmetallating with [RhCl(L−L)]_2_ (L−L=NBD or COD).^[52]^ This transmetallation procedure was successful for these compounds, unlike those with an azide terminus, and using Ag_2_O instead of K_2_CO_3_ prevented the extensive formation of hydantoin by‐products.

**Scheme 3:**
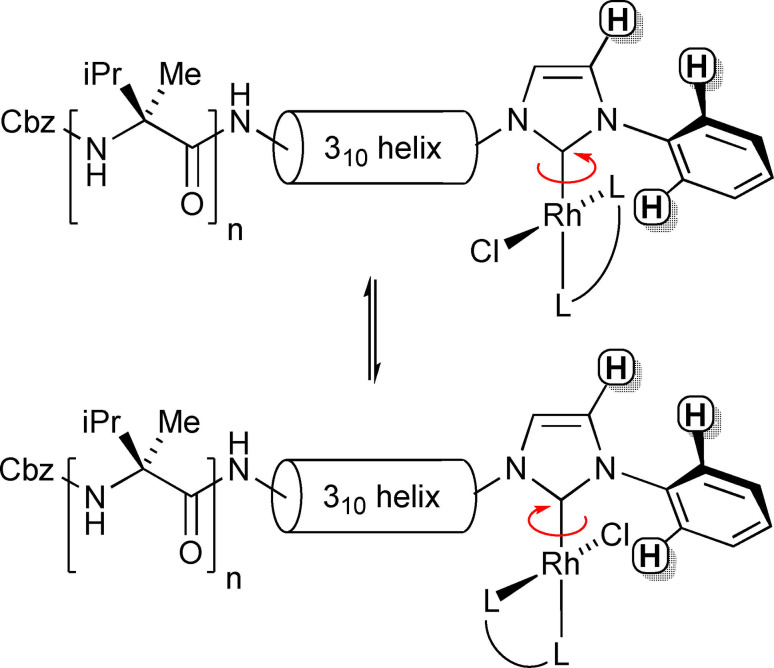
Foldamer diastereoisomers in **14** (*n*=1, L−L=COD), **15** (*n*=1, L−L=NBD) and **17** (*n*=2, L−L=COD) that arise due to hindered rotation around the Rh−C(NHC) axis. C4‐H NHC and *ortho*‐CH protons indicated.

**Figure 5:**
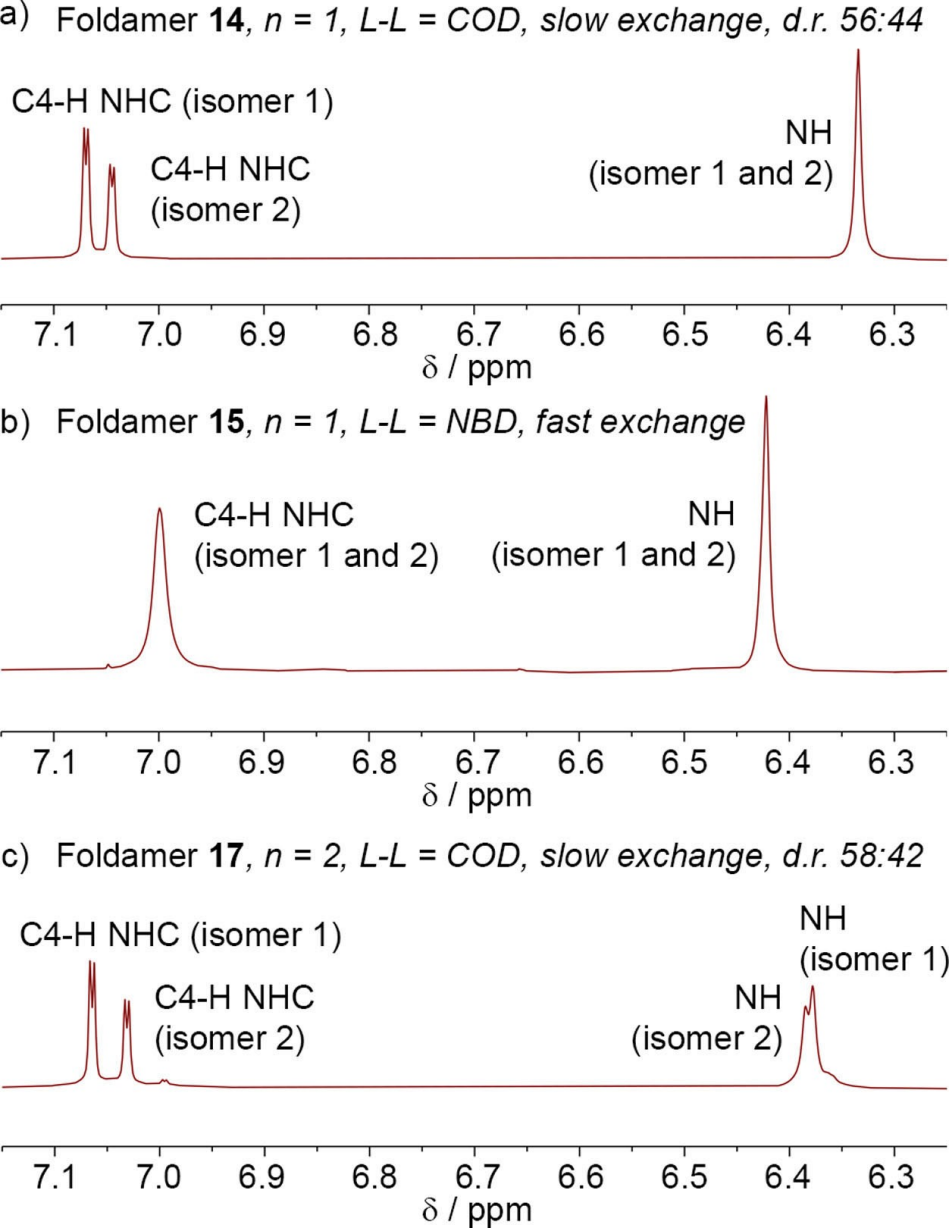
a–c) Partial ^1^H NMR spectra of [(Cbz‐(L‐αMeVal)_n_Aib_4_NH(CH_2_)_2_‐NHC−Ph)Rh(Cl)(L−L)] in CDCl_3_ at 25 °C from 7.15 to 6.25 ppm showing the C4‐H NHC proton and an NH proton from both diastereoisomers (isomers 1 and 2) of a) **14**; b) **15**; c) **17**.

The formation of two diastereoisomers (Scheme 3) was clear for these foldamers that have chiral groups distant from each other. For [(Cbz‐(L‐αMeVal)Aib_4_NH(CH_2_)_2_‐NHC−Ph)Rh(Cl)(COD)] **14**, two sets of eight resonances from the anisotropic Aib methyl groups were observed. The ^1^H NMR resonances for the NHC proton nearest the phenyl group (∼7.1 ppm, identified using NOESY) and an NH proton (∼6.4 ppm) show the two diastereoisomers clearly (Figure 5a). The two diastereoisomers are also clearly visible in the ^13^C NMR spectrum (see the Supporting Information). The HSQC spectrum shows that each set of signals in the ^1^H NMR spectrum correlate with a different set of ^13^C NMR resonances, supporting the presence of two diastereoisomers that are not in rapid exchange (see the Supporting Information).

Since rotation around the Rh−C(NHC) bond will interconvert the diastereoisomers (albeit slowly for COD complexes), the ratio of the two isomers indicates their relative stability. Integration of the relevant peaks in the ^1^H NMR spectrum of **14** shows that the two diastereoisomers are not present in an equal ratio but are in a 56 : 44 ratio (a *d.e*. of 12 %). This low *d.e*. suggests that the energetic penalty for a “mismatch” between the *P* screw‐sense induced by the N‐terminal L‐αMeVal and an *M* screw‐sense induced by one of the Rh(I)−C(NHC) conformations is small compared to “matching” of screw‐sense preferences, that is, when the other Rh(I)−C(NHC) conformation, which induces a *P* screw‐sense, is present.

**Figure 6:**
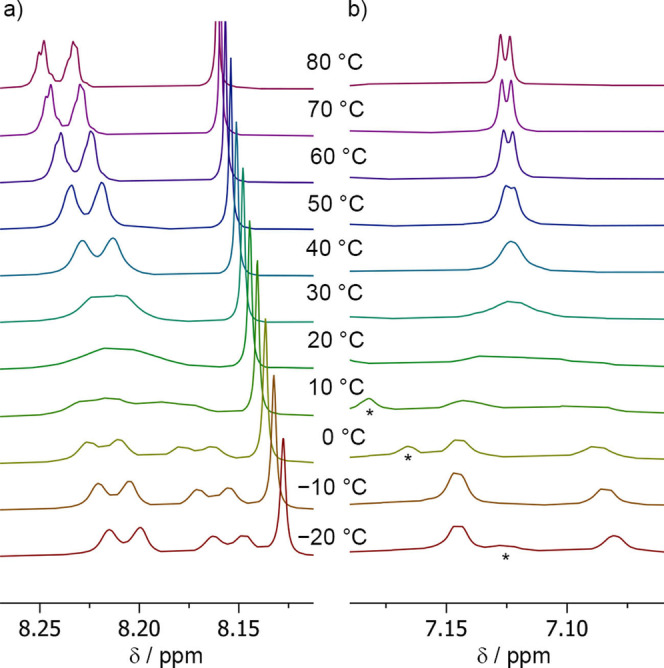
a, b) Partial ^1^H NMR spectra of foldamer **15** in CDCl_2_CDCl_2_ upon heating from −20 to 80 °C showing a) the resonances assigned to the *ortho* CH protons and b) the C4‐H NHC proton. The asterisk indicates an NH proton resonance that moves upon warming.

To confirm that the diastereoisomers of **14** arise from slow rotation around the Rh‐NHC bond, the bulky COD ligand was replaced with NBD. The resulting complex, [(Cbz‐(L‐αMeVal)Aib_4_NH(CH_2_)_2_‐NHC−Ph)Rh(Cl)(NBD)] **15**, was subjected to ^1^H VT NMR spectroscopy in CDCl_2_CDCl_2_ from −20 to +80 °C. The resonances from the *ortho*‐ protons on the NHC phenyl group (8.28–8.13 ppm) and the C4‐H NHC protons (7.15–7.05 ppm) were found to coalesce at 20 °C (Figure 6). Calculation of the exchange rates (*k*) from the VT NMR data by line shape analysis was consistent with a diastereomeric ratio (*d.r*.) of 55 : 45 and rate constants for exchange of ∼110 s^−1^ at room temperature. The activation energy for diastereoisomer interconversion was calculated as Δ*G* ^≠^=60.6 kJ mol^−1^ at 298 K, a value similar to both the *E*_a_ estimated for **8** and reported Rh−C(NHC) rotation barriers in Rh(I)NHC(NBD) complexes.[^58^, ^60^]

The magnitude of the energetic penalty for a mismatch may increase if stronger control is imparted from the N‐terminus. Adding another L‐αMeVal chiral controller at the N‐terminus gives a stronger preference for *P* helix. The effect of stronger N‐terminal chiral control on the *d.r*. was revealed in the ^1^H NMR spectrum of [(Cbz‐(L‐αMeVal)_2_Aib_4_NH(CH_2_)_2_‐NHC−Ph)Rh(Cl)(COD)] **17**, which like **14** shows two distinct sets of resonances in the ^1^H NMR spectrum from each diastereoisomer but with greater separation of the CH and NH chemical shifts (Figure 5c). Integration of the relevant peaks in the ^1^H NMR spectrum shows that the two diastereoisomers are present in a 58 : 42 equal ratio, a slight improvement of the *d.e*. to 16 %. However, replacing the phenyl group with the bulkier mesityl group in **18** resulted in a decrease in this ratio to 52 : 48. The appearance of the spectrum for [(Cbz‐(L‐αMeVal)Aib_5_NH(CH_2_)_2_‐NHC−Mes)Rh(Cl)(COD)] **19**, an analogue with the second L‐αMeVal chiral controller replaced with Aib shows a further decrease in the ratio (50 : 50 isomer ratio). The lower *d.e*. values for these analogues with Mes in the place of Ph suggest that bulky aryl groups on the NHC may make the rhodium(I) complex less susceptible to a chiral influence relayed from an N‐terminal residue.

### Catalysis by Aib foldamer rhodium(I) complexes

Several reactions are reported to be catalysed by Rh(NHC) complexes under mild conditions, with the hydrosilylation of alkynes, ketones and aldehydes particularly efficiently catalysed.[^58^, ^64^]

**Scheme 4:**
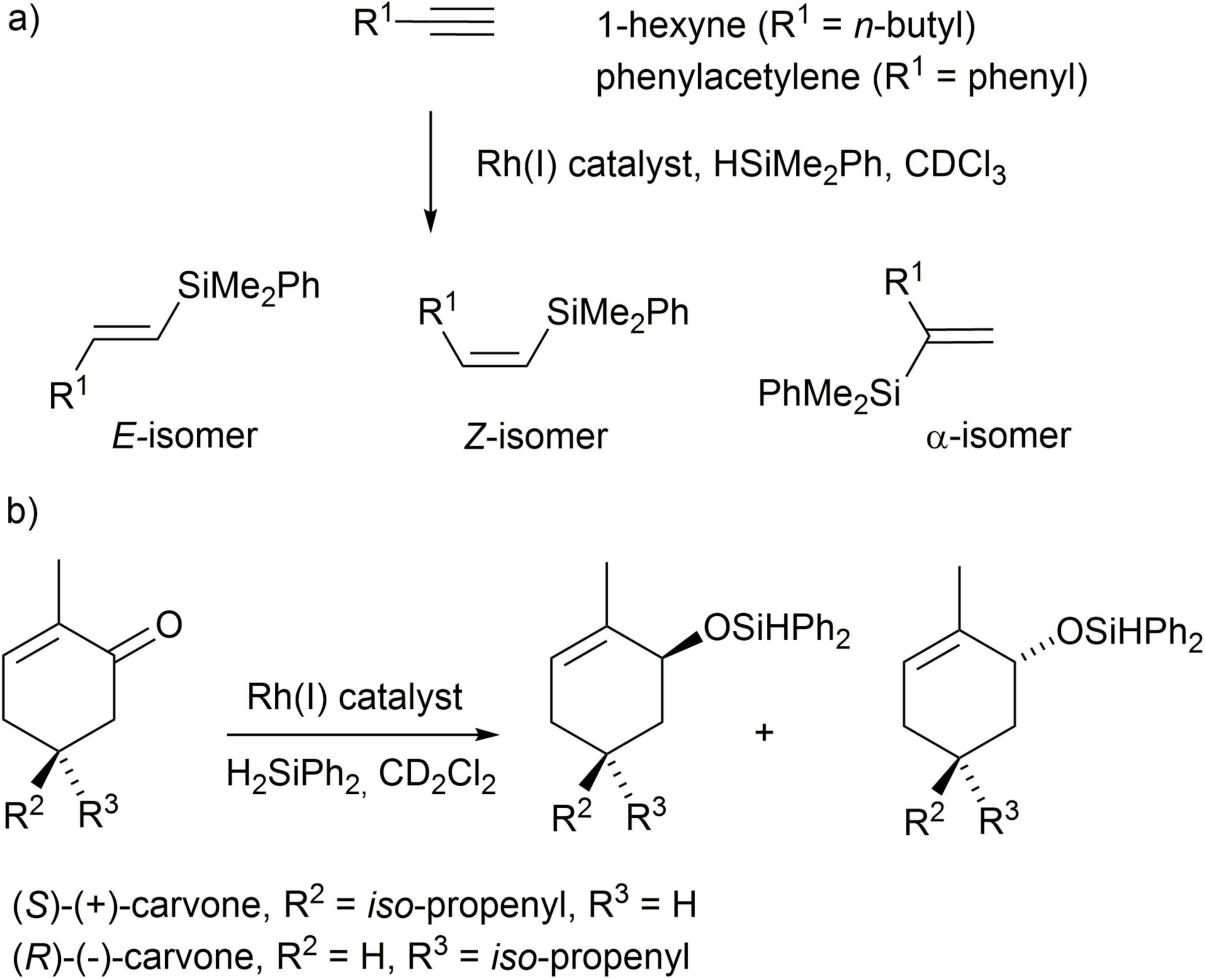
Rhodium(I) mediated hydrosilylation of a) 1‐hexyne and phenylacetylene with HSiMe_2_Ph; b) (*S*)‐(+)‐carvone and (*R*)‐(−)‐carvone with H_2_SiPh_2_.

We focused on how the folded structure adjacent to the Rh(NHC) may affect the outcome of catalysed reactions. Hydrosilylation reactions catalysed by non‐foldamer complexes similar to **1** have been reported, with [(BocNH(CH_2_)_2_‐NHC−R)Rh(Cl)(NBD)] and HSiMe_2_Ph giving up to 48 % silylation of 1‐hexyne after 2 h at 25 °C (R=Me). Steric hindrance around the NHC was shown to strongly decrease reaction rates (for example, 26 % conversion after 1 day for R=trityl). The *Z*‐isomer was initially favoured with significant amounts of *E*‐ and α‐product also observed (isomers shown in Scheme 4a), for example the *Z : E*:α ratio was 40 : 33 : 27 at 2 h for R=Me.^[58]^ Other closely related complexes gave almost complete hydrosilylation of phenylacetylene after 0.5 h at room temperature (*E*‐isomer predominates) and of 1‐hexyne after 1 h at 60 °C (*Z*‐isomer predominates).^[64b]^

Before measuring catalysis by Aib foldamers bearing rhodium(I)‐NHCs, two non‐folded complexes were assessed. Control compound **1** was a competent catalyst for the hydrosilylation of 1‐hexyne with HSiPhMe_2_ in CDCl_3_, giving 30 % conversion after 14 h and >95 % conversion after 44 h, with the *Z‐*isomer the major product (Table 1). Chiral analogue **20**, with (+)‐Mosher's acid at one end and mesityl at the other (Scheme 2b), had lower activity (54 % conversion after 44 h, 94 % after 119 h) and somewhat worse *Z‐* selectivity than **1** (Table 1 and Supporting Information).

**Table 1:** Products, reaction times and conversions for the hydrosilylation of 1‐hexyne by compound **1** and foldamers **2**, **3**, **14**, **19**, and **20**.

Compound	Ratio *Z : E*:α	Time [h]	Conversion
**1**	78 : 15 : 7	14	30 %
**1**	82 : 13 : 5	44	>95 %
**20**	71 : 21 : 8	119	94 %
**2**	50 : 32 : 18	224	77 %
**14**	61 : 26 : 11	29	68 %
**3**	83 : 11 : 7	48	84 %
**19**	70 : 22 : 8	47	96 %

**Figure 7:**
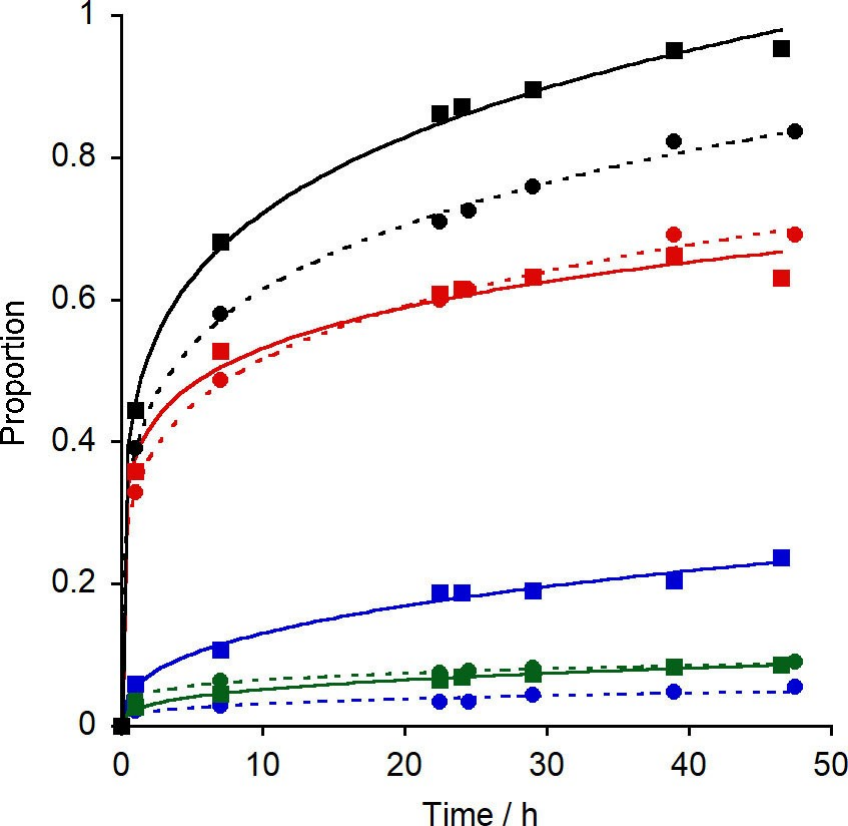
Time course showing the production of isomeric products from the catalysed hydrosilylation of 1‐hexyne (*Z* isomer: red symbols; *E* isomer: blue symbols; α‐isomer: green symbols). Catalysts were [(N_3_Aib_4_NH(CH_2_)_2_‐NHC−Ph)Rh(Cl)(COD)] **2** (circles) [(Cbz(L‐αMeVal)Aib_5_NH(CH_2_)_2_‐NHC−Ph)Rh(Cl)(COD)] **19** (squares). Overall conversion is shown for **2** (black circles) and **19** (black squares). Curve fits are to guide the eye.

We then assessed the catalytic activity of the foldamers. Overall reaction rates were generally between those of **1** and **20**, although Aib foldamers with azide N‐termini were slower. For example, 1‐hexyne was 68 % hydrosilylated after 29 h when using catalyst **14**, but 224 h with catalyst **2** were required to give a similar level of conversion (77 %). Leaving the former reaction mixture for longer led to the *Z*‐isomer and an allyl isomer becoming the major components (42 % and 31 % respectively), consistent with higher reactivity for this catalyst. Replacing the Ph on the NHC with Mes produced a strong increase in selectivity; foldamer **3** showed both higher reactivity and better *Z*‐selectivity than analogue **2** (Table 1). The N‐terminal chiral controller in catalyst **19** gave slightly improved conversion after 47 h (96 %) compared to **3** but with lower *Z‐* selectivity (Figure 7).

Applying the same catalysts and reaction conditions with phenylacetylene showed a similar pattern but with higher conversions (see the Supporting Information, Table S1). Catalyst **2** gave a ∼1 : 1 *Z : E* ratio (79 % conversion after 224 h), compared to a ∼2 : 1 *Z : E* ratio for **14** (97 % conversion after 103 h). Catalyst **3** gave a ∼6 : 1 *Z : E* ratio (74 % conversion after 48 h) compared to a ∼4 : 1 *Z : E* ratio for **19** (91 % after 48 h).

The catalytic hydrosilylation of carvone with H_2_SiPh_2_ (1.1 equiv.) in CD_2_Cl_2_ at room temperature was also assessed (Scheme 4b). Reduction of the ketone provides both *cis* and *trans* isomers of the alcohol, for example reduction with LiAlH_4_/AlCl_3_ provides the *cis* isomer preferentially (*cis*:*trans*=86 : 14).^[65]^ However reduction using a chiral Rh(I) catalyst has been shown to alter this diastereoselectivity, with higher selectivity observed for an (*S*) catalyst (*cis*:*trans* >99 : 1) compared to the enantiomeric (*R*) catalyst (*cis*:*trans* ≈79 : 21).^[64c]^ Instead of making both catalyst enantiomers, the same insight can be obtained by applying one catalyst enantiomer with both (+)‐ and (−)‐carvone. Any catalyst‐dependent stereoselectivity will be revealed as different *cis*:*trans* ratios in the respective carveol‐derived products.

Catalysis by racemic rhodium(I) complexes **2**–**3** and chiral L‐αMeVal functionalized rhodium catalysts **14**–**19** was assessed. Reaction mixtures generally stopped changing composition within 3 h, although those catalysed by L‐αMeVal‐capped foldamers finished more quickly and provided higher proportions of products. A preference for the *cis* isomer of the silyl ether was observed with racemic Aib foldamers **2** and **3** (*cis*:*trans* ≈75 : 25). A chiral N‐terminal residue did not significantly alter the ratio of *cis*:*trans* isomers produced from either carvone enantiomers. Foldamer **14** gave *cis*:*trans* ≈76 : 24 for both (+)‐ and (−)‐carvone, implying that the *d.r*. of **14** (56 : 44) is too small to change stereoselectivity during catalysis. Indeed, all the chiral foldamer catalysts **14**–**19** gave *cis*:*trans* ≈75 : 25 ratio with the carvone enantiomers, the same as that produced by the racemic complexes **2** and **3** (see the Supporting Information). Bringing a chiral centre closer to the rhodium(I) center did not generate any change in stereoselectivity during catalysis; both carvone enantiomers gave a 72 : 28 *cis*:*trans* ratio after hydrosilylation catalysed by **20**.

## Conclusion

The ability to relay conformational information across multi‐nanometre distances in biomimetic systems has wide implications in the fields of nanotechnology, artificial cells and molecular machines, particularly if this information can generate or alter a catalytic output.[^66^, ^67^] Although conformational dynamics in general are known to be important for the reactivity of several types of foldamers,^[68]^ only in a few cases have remote conformational changes been relayed along a foldamer to a catalytic site. Clayden and co‐workers showed that remote conformational differences in Aib foldamers can change the stereochemical outcome of organic reactions, including organocatalyzed reactions, at distances comparable to the size of proteins (up to 4 nm).[^6^, ^38a^, ^39^] The work herein shows how such Aib foldamer‐based information relays could be applied to the broader range of catalytic reactions available to organometallic complexes. Rhodium NHC complexes were attached to a peptide foldamer architecture and shown to remain active hydrosilylation catalysts. The hydrosilylation of alkynes by **2**, **3**, **14** and **19** showed the foldamer scaffold around the metal complex did not significantly change the rate of hydrosilylation under these conditions. This catalysis illustrates one of the benefits of organometallic complexes, which is their ability to catalyse a diverse set of reactions that are otherwise hard to access through organocatalysis, including metathesis, hydrogenation and aromatic/alkene/alkyne C−C bond formation.

Foldamers **2** to **19** are the first examples of foldamers that have a chiral axis located within a metal complex.^[69]^ Foldamers **9** to **19** therefore possess a mixture of point (in the αMeVal residue), helical (in the Aib_4_ body) and axial (in the Rh(NHC)) chirality. Few foldamers have been developed that combine these stereochemical motifs. Diemer et al. placed an axially chiral dibenzazepinyl group at the N‐terminus of a family of Aib foldamers, but since the axial chiral dibenzazepinyl group was in fast exchange no diastereoisomers were visible in the ^1^H NMR spectra.^[45]^ Similarly, Yashima and co‐workers placed an axially chiral but rapidly interconverting bipyridyl bridge between the N‐termini of two (Ac_6_c)_4_‐(L–Val)‐Aib foldamers. The axial chirality could be fixed by oxidation of the pyridyl nitrogens, which gave (a*R*) enriched isomers with a *d.e*. of 26 %.^[46]^ Although the 16 % *d.e*. observed in **17**, which has the powerful chiral inducer Cbz(L‐αMeVal)_2_, is comparable to that of the complex described by Yashima and co‐workers, it is poor when compared to the +72 % *h.e*. induced by the same N‐terminal group in Cbz(αMeVal)_2_Aib_4_GlyNH_2_.^[32b]^

The ability to measure the *d.r*. in the Rh(NHC) foldamer catalysts using ^1^H NMR spectroscopy allowed us to identify poor stereochemical communication from the (Aib)_4_ core, through the ethylene bridge, to the Rh(NHC) complex as the most likely reason for the absence of changes in *d.e*. after the hydrosilylation of carvone enantiomers. To replicate the influence of peptide structure on catalysis by a metal ion in metalloenzymes, foldamers with the catalytic centre more tightly integrated into the folded structure may be needed; further work in this area is ongoing.

## Conflict of interest

The authors declare no conflict of interest.

1

## Supporting information

As a service to our authors and readers, this journal provides supporting information supplied by the authors. Such materials are peer reviewed and may be re‐organized for online delivery, but are not copy‐edited or typeset. Technical support issues arising from supporting information (other than missing files) should be addressed to the authors.

Supporting Information

## Data Availability

The data that support the findings of this study are available in the supplementary material of this article.
