# Supplementary material for: α‐Amino‐iso‐Butyric Acid Foldamers Terminated with Rhodium(I) N‐Heterocyclic Carbene Catalysts
Source: Chemistry. 2022 Jan 12;28(9):e202104293. doi: 10.1002/chem.202104293 (PMC9305545; doi:10.1002/chem.202104293)
Supplement: Supplementary file 1 — Supporting Information [file CHEM-28-0-s001.pdf]

Chemistry–A European Journal

Supporting Information

α-Amino-*iso*-Butyric Acid Foldamers Terminated with Rhodium(I) N-Heterocyclic Carbene Catalysts

David P. Tilly, William Cullen, Heng Zhong, Romain Jamagne, Inigo Vitórica-Yrezábal, and Simon J. Webb*

TABLE OF CONTENTS

1. Chemical synthesis: instruments **4**

2. Chemical synthesis: materials **5**

2.1 Abbreviations 5

2.2 Materials 5

2.3 General experimental procedures 5
2.3.1 General procedure 1: Hydrogenation of N_3Aib_nOR (for n = 1-4) 5
2.3.2 General procedure 2: Synthesis of N_3Aib_nOR 5
2.3.3 General procedure 3: Synthesis of N_3Aib_nOH 6
2.3.4 General procedure 4: Synthesis of $[XAib_nNH(CH_2)_2(Im\text{-}Ar)]^+Br^-$ 6
2.3.5 General procedure 5: Synthesis of $[(N_3Aib_nNH(CH_2)_2NHC\text{-}Ar)Rh(L^1)(L^2)]$ 6
2.3.6 General procedure 6: Synthesis of $[(Cbz(L\text{-}\alpha MeVal)_mAib_nNH(CH_2)_2NHC\text{-}Ar)Rh(L^1)(L^2)]$ 6

3. Synthetic procedures **7**

3.1 2-Azido-2-methylpropionic acid N_3AibOH 7

3.2 N_3AibO^tBu 7

3.3 NH_2AibO^tBu 7

3.4 $N_3Aib_2O^tBu$ 7

3.5 $H_2NAib_2O^tBu$ 8

3.6 $N_3Aib_3O^tBu$ 8

3.7 $NH_2Aib_3O^tBu$ 8

3.8 $N_3Aib_4O^tBu$ 8

3.9 $NH_2Aib_4O^tBu$ 9

3.10 $N_3Aib_5O^tBu$ 9

3.11 $NH_2Aib_5O^tBu$ 9

3.12 Cbz-(L-αMeVal)-Aib_4O^tBu 10

3.13 Cbz-(L-αMeVal)-Aib_5O^tBu 10

3.14 Cbz-(L-αMeVal)$_2$-Aib_4O^tBu 11

3.15 N_3Aib_4OH 12

3.16 Cbz-(L-αMeVal)-Aib_4OH 12

3.17 Cbz-(L-αMeVal)-Aib_5OH 12

3.18 Cbz-(L-αMeVal)$_2$-Aib_4OH 13

3.19 *N*-Mesitylimidazole 13

3.20 *N*-Phenylimidazole 14

3.21 1-(2-Aminoethyl)-3-(phenyl)-1H-imidazol-3-ium bromide, hydrobromide salt, **5** 14

3.22 1-(2-Aminoethyl)-3-(2,4,6-trimethylphenyl)-1H-imidazol-3-ium bromide, HBr salt, **6** 14

3.23 1-(2-Aminoethyl)-3-(methyl)-1H-imidazol-3-ium bromide, hydrobromide salt, **7** 15

3.24 1-(2-Isobutyramidoethyl)-3-phenyl-1H-imidazol-3-ium bromide, Precursor **S1** 15

3.25 Compound **1**, $[(^iPrC(O)NH(CH_2)_2\text{-}NHC\text{-}Ph)Rh(Cl)(COD)]$ 15

3.26 $N_3Aib_4(CH_2)_2(Im)Ph^+Br^-$, Precursor **S2** 17

3.27 Foldamer **2**, $[N_3Aib_4NH(CH_2)_2\text{-}NHC\text{-}Ph)Rh(Cl)(COD)]$ 17

3.28 Foldamer **8**, $[N_3Aib_4NH(CH_2)_2\text{-}NHC\text{-}Ph)Rh(Cl)(NBD)]$ 18

3.29 [N_3Aib$_4$(CH_2)$_2$(Im-Mes)]$^+$Br$^-$, Precursor **S3** 18

3.30 Foldamer **3**, [(N_3Aib$_4$NH(CH_2)$_2$-NHC-Mes)Rh(Cl)(COD)] 19

3.31 [N_3Aib$_4$(CH_2)$_2$(Im-Me)]$^+$Br$^-$, Precursor **S4** 19

3.32 Foldamer **4**, [(N_3Aib$_4$NH(CH_2)$_2$-NHC-Me)Rh(Cl)(COD)] 20

3.33 Precursor **9**, [Cbz-(L-αMeVal)Aib$_4$NH(CH_2)$_2$(Im-Ph)]$^+$Br$^-$ 20

3.34 Foldamer **14**, [(Cbz-(L-αMeVal)Aib$_4$NH(CH_2)$_2$-NHC-Ph)Rh(Cl)(COD)] 21

3.35 Foldamer **15** [(Cbz-(L-αMeVal)Aib$_4$NH(CH_2)$_2$-NHC-Ph)Rh(Cl)(NBD) 22

3.36 Precursor **10**, [Cbz-(L-αMeVal)Aib$_4$NH(CH_2)$_2$(Im-Mes)]$^+$Br$^-$ 22

3.37 Foldamer **16**, [(Cbz-(L-αMeVal)Aib$_4$NH(CH_2)$_2$-NHC-Mes)Rh(Cl)(COD)] 23

3.38 Precursor **11**, [(Cbz-(L-αMeVal)$_2$Aib$_4$NH(CH_2)$_2$(Im-Ph)]$^+$Br$^-$ 24

3.39 Foldamer **17** [(Cbz-(L-αMeVal)$_2$Aib$_4$NH(CH_2)$_2$-NHC-Ph)Rh(Cl)(COD)] 25

3.40 Precursor **12**, [Cbz-(L-αMeVal)$_2$Aib$_4$NH(CH_2)$_2$(Im-Mes)]$^+$Br$^-$ 26

3.41 Foldamer **18**, [(Cbz-(L-αMeVal)$_2$Aib$_4$NH(CH_2)$_2$-NHC-Mes)Rh(Cl)(COD)] 26

3.42 Precursor **13**, [(Cbz-(L-αMeVal)Aib$_5$NH(CH_2)$_2$(Im-Mes)]$^+$Br$^-$ 27

3.43 Foldamer **19**, [(Cbz-(L-αMeVal)Aib$_5$NH(CH_2)$_2$-NHC-Mes)Rh(Cl)(COD)] 28

3.44 Intermediate [(+)-Mosher-NH(CH_2)$_2$(Im-Mes)]$^+$Br$^-$ 29

3.45 Foldamer **20**, [(+)-MosherNH(CH_2)$_2$-NHC-Mes)Rh(Cl)(COD)] 30

4. ^{1}H and ^{13}C NMR spectra of synthesized compounds 31

4.1 Cbz-(L-αMeVal)-Aib$_5$O^{t}Bu 31

4.2 Cbz-(L-αMeVal)-Aib$_5$OH 33

4.3 1-(2-Aminoethyl)-3-(phenyl)-1H-imidazol-3-ium bromide, hydrobromide salt **5** 36

4.4 1-(2-Aminoethyl)-3-(2,4,6-methylphenyl)-1H-imidazol-3-ium bromide, HBr salt **6** 37

4.5 Precursor S1 1-(2-isobutyramidoethyl)-3-phenyl-1H-imidazol-3-ium bromide 38

4.6 Compound **1** 40

4.7 Precursor **S2** [N_3Aib$_4$(CH_2)$_2$(Im-Ph)]$^+$Br$^-$ 47

4.8 Foldamer **2** [(N_3Aib$_4$NH(CH_2)$_2$-NHC-Ph)Rh(Cl)COD)] 49

4.9 Foldamer **8**, [(N_3Aib$_4$NH(CH_2)$_2$-NHC-Ph)Rh(Cl)(NBD)] 53

4.10 Precursor **S3** [N_3Aib$_4$(CH_2)$_2$(Im-Mes)]$^+$Br$^-$ 56

4.11 Foldamer **3** [(N_3Aib$_4$NH(CH_2)$_2$-NHC-Mes)Rh(Cl)COD)] 58

4.12 Precursor **S4** [N_3Aib$_4$(CH_2)$_2$(Im-Me)]$^+$Br$^-$ 62

4.13 Foldamer **4** [(N_3Aib$_4$NH(CH_2)$_2$-NHC-Me)Rh(Cl)COD)] 64

4.14 Precursor **9** [Cbz-(L-αMeVal)Aib$_4$NH(CH_2)$_2$(Im-Ph)]$^+$Br$^-$ 65

4.15 Foldamer **14** [(Cbz-(L-αMeVal)Aib$_4$NH(CH_2)$_2$-NHC-Ph)Rh(Cl)(COD)] 69

4.16 Foldamer **15** [(Cbz-(L-αMeVal)Aib$_4$NH(CH_2)$_2$-NHC-Ph)Rh(Cl)(NBD)] 77

4.17 Precursor **10**, [Cbz-(L-αMeVal)Aib$_4$NH(CH_2)$_2$(Im-Mes)]$^+$Br 80

4.18 Foldamer **16**, [(Cbz-(L-αMeVal)Aib$_4$NH(CH_2)$_2$-NHC-Mes)Rh(Cl)(COD)] 83

4.19 Precursor **11** [Cbz-(L-αMeVal)$_2$Aib$_4$NH(CH_2)$_2$(Im-Ph)]$^+$Br$^-$ 87

4.20 Foldamer **17** [(Cbz-(L-αMeVal)$_2$Aib$_4$NH(CH_2)$_2$-NHC-Ph)Rh(Cl)(COD)] 90

4.21 Precursor **12** [Cbz-(L-αMeVal)$_2$Aib$_4$NH(CH_2)$_2$(Im-Mes)]$^+$Br$^-$ 94

4.22 Foldamer **18** [(Cbz-(L-αMeVal)$_2$Aib$_4$NH(CH_2)$_2$-NHC-Mes)Rh(Cl)(COD)] 97

4.23 Precursor **13** [Cbz-(L-αMeVal)Aib$_5$NH(CH_2)$_2$(Im-Mes)]$^+$Br$^-$ 101

4.24 Foldamer **19** [(Cbz-(L-αMeVal)$Aib_5NH(CH_2)_2$-NHC-Mes)Rh(Cl)(COD)] 104

4.25 Intermediate [(+)-Mosher-$NH(CH_2)_2$(Im-Mes)]$^+Br^-$ 107

4.26 Foldamer **20** [(+)-Mosher$NH(CH_2)_2$-NHC-Mes)Rh(Cl)(COD)] 110

5. Variable temperature ^{1}H NMR studies (VT-NMR) 114

5.1 Procedure 114

5.2 Fitting 114

5.2.1 Procedure for determining the activation energy 120

6. Catalysis 122

6.1 Procedure for alkyne hydrosilylation 122

6.2 Procedure for carvone hydrosilylation 124

7. Crystal data and structure refinement 127

7.1 Foldamer **2** 127

8. References 135

1. Chemical synthesis: instruments

Nuclear Magnetic Resonance (NMR) spectra were recorded on Bruker Ultrashield 400 MHz and 500 MHz spectrometers in deuterated solvents. All NMR characterisation experiments were recorded at 25 °C unless otherwise stated. Spectra were calibrated using the residual solvent signals for $CDCl_3$ (δH: 7.26 ppm; δC: 77.16 ppm), CD_2Cl_2 (δH: 5.32 ppm; δC: 53.84 ppm) and $(CD_3)_2SO$ (δH: 2.50 ppm; δC: 39.52 ppm) as appropriate. Coupling constants (J) are reported in Hertz (Hz) and rounded to 0.1 Hz. Chemical shifts (δ) were measured in parts per million (ppm). Splitting patterns are illustrated as follows: singlet (s), doublet (d), triplet (t), multiplet (m), broad (b) or some combination of these. Assignments of the peaks were made based on chemical shifts, coupling constants, COSY, NOESY, HSQC and HMBC data. ^{1}H–^{13}C HSQC experiments were used to assign the paired signals of the diastereotopic groups of the relevant rhodium foldamers.

Infrared spectra were recorded on a Thermo Scientific Nicolet iS5 FTIR Spectrometer with samples applied as neat films. Absorption maxima (ν_{max}) of interest are quoted in wavenumbers (ν in cm^{-1}).

Low- and high-resolution mass spectra were recorded by staff at the University of Manchester. Electrospray (ES) spectra were recorded on a Waters Platform II. High-resolution mass spectra (HRMS) were recorded on a Thermo Finnigan MAT95XP and are accurate to ±0.001 Da.

Melting points were measured on a Stuart SMP10 melting point apparatus and are uncorrected.

X-ray crystallography was carried out using a dual source Rigaku FR-X rotating anode diffractometer using CuK_α wavelength radiation (λ = 1.54184) at a temperature of 100 K at the University of Manchester.

2. Chemical synthesis: materials

2.1 Abbreviations

The following abbreviations have been used: Aib = aminoisobutyric acid, α-methylvaline = αMeVal, COD = 1,5-cyclooctadiene, DIPEA = *N,N*-diisopropylethylamine, EDC·HCl = *N*-(3-dimethylaminopropyl)-1-*N′*-ethylcarbodiimide, EtOAc = ethyl acetate, EtOH = ethanol, DMF = *N,N*-dimethylformamide, DCM = dichloromethane, Et_2O = diethylether, HOBt = 1-hydroxybenzotriazole, Im = imidazolium, MeOH = methanol, NBD = norbornadiene = bicyclo[2.2.1]hepta-2,5-diene, THF = tetrahydrofuran, NEt_3 = triethylamine, TLC = thin layer chromatography.

2.2 Materials

All reactions were carried out in oven-dried glassware under an atmosphere of nitrogen using standard anhydrous techniques. Anhydrous tetrahydrofuran, acetonitrile, dichloromethane, acetone and *N*,*N*-dimethylformamide were purchased from Sigma-Aldrich. Petroleum ether refers to the fraction of light petroleum ether boiling between 40 and 60 °C. All other solvents and commercially available reagents were used as received without further purification

All products were concentrated on a rotary evaporator followed by connection to a high vacuum system to remove any residual solvent. Preparative chromatography (flash) was performed on normal phase silica gel (Merck 60H, 40-60 nm, 230–300 mesh). Analytical thin layer chromatography (TLC) was performed using pre-coated plates (Macherey-Nagel Polygram SIL G/UV254). Visualisation was achieved by way of UV light (at 254 nm), and staining with either potassium permanganate, phosphomolybdic acid in ethanol, or ninhydrin in ethanol where appropriate. Stained TLC plates were heated for visualization.

2.3 General experimental procedures

2.3.1 General procedure 1: Hydrogenation of N_3Aib_nOR (for n = 1-4)

N_3Aib_nOR (1 eq) was dissolved in EtOH (5 mL/1 mmol) under a nitrogen atmosphere. Pd/C (10%, 10 mg/1 mmol) was added and the reaction mixture stirred under an atmosphere of H_2 until the starting material was consumed (monitored by TLC). The mixture was then filtered through a pad of Celite® 535, the Celite® 535 was washed with EtOAc, then the filtrate was concentrated. The residue was purified by chromatography on silica where necessary.

2.3.2 General procedure 2: Synthesis of N_3Aib_nOR

N_3AibOH (2 eq) was dissolved in dry CH_2Cl_2 (4 mL/1 mmol). EDC*HCl (2 eq) was added in portions and stirred for 15 min. Afterwards, HOBt (0.1 eq) and Et_3N (1.2 eq) were added dropwise and stirred for another 15 min. NH_2Aib_nOR (1 eq) was then added and the mixture was stirred at room temperature until completion of the reaction (monitored by TLC). The reaction mixture was washed with 5% aqueous $KHSO_4$ solution (2 × 20 mL), sat. $NaHCO_3$ aqueous solution (2 × 20 mL). The organic layers were dried over $MgSO_4$, filtered, then the solvent was removed under reduced pressure.

2.3.3 General procedure 3: Synthesis of N_3Aib_nOH

$N_3Aib_nO^tBu$ (1 eq) was dissolved in a 1:2 mixture of trifluoroacetic acid (1 mL/1 mmol) and CH_2Cl_2 (2 mL/1mmol). The resulting mixture was stirred at room temperature under a nitrogen atmosphere until completion of the reaction (monitored by TLC). The solvent was removed under reduced pressure, the residue was redissolved in Et_2O (5 × 30 mL) then the solvent was removed under reduced pressure (co-evaporation to remove remaining traces of trifluoroacetic acid).

2.3.4 General procedure 4: Synthesis of $[XAib_nNH(CH_2)_2(Im\text{-}Ar)]^+Br^-$

To a solution of $XAib_nOH$ (1 mmol) in dry dichloromethane (25 mL) was added *N*-(3-dimethylaminopropyl)-*N'*-ethylcarbodiimide EDC.HCl (0.384 g, 2 mmol). The mixture was cooled to 0°C, triethylamine (0.29 mL, 2 mmol) was added and the mixture was stirred for 3 hours at 20 °C. The resulting mixture was concentrated, dissolved in acetonitrile (20 mL). The arylimidazolium ethylammonium dibromide salt (1.1 mmol) was added, followed with triethylamine (0.17 mL, 1.18 mmol) and the suspension was heated at 80 °C for 3 days (the suspension solubilised upon heating). The mixture was concentrated under reduced pressure; the residue was purified by chromatography on silica (eluent CH_2Cl_2:MeOH 95:5). The fractions containing the product but contaminated with triethylammonium salt impurity were purified by a second column chromatography on basic Al_2O_3 (DCM:MeOH 95:5) to yield the imidazolium bromide salt as a colorless oil. The products do not stain well with ninhydrin or $KMnO_4$ and are only weakly UV absorbing.

2.3.5 General procedure 5: Synthesis of $[(N_3Aib_nNH(CH_2)_2NHC\text{-}Ar)Rh(L^1)(L^2)]$

Under a nitrogen atmosphere, to a 0.03 M solution of $[N_3Aib_nNH(CH_2)_2(Im\text{-}Ar)]^+Br^-$(1 equiv.) in dry acetone was added potassium carbonate K_2CO_3 (3 equiv.) and $[Rh(L^1)(L^2)]_2$ (0.5 equiv., L = ligand, L^1: cyclooctadiene or norbornadiene, L^2: chloride). The mixture was heated at 60 °C for 5 h. The resulting mixture was concentrated, the residue was purified using chromatography on silica (eluent 100 % CH_2Cl_2 then a gradient to CH_2Cl_2:MeOH 90:10) to afford the product as a yellow solid.

2.3.6 General procedure 6: Synthesis of $[(Cbz(L\text{-}\alpha MeVal)_mAib_nNH(CH_2)_2NHC\text{-}Ar)Rh(L^1)(L^2)]$

Under a nitrogen atmosphere, to a 0.03 M solution of $[Cbz(L\text{-}\alpha MeVal)_mAib_nNH(CH_2)_2(Im\text{-}Ar)]^+Br-$ (1 equiv.) in dry CH_2Cl_2 was added silver oxide (1 equiv) at 20 °C and the mixture was stirred for 2 hours under an inert atmosphere in the dark. The dirhodium(I) dichloride $[Rh(ligand)Cl]_2$ (ligand = COD or NBD) (0.5 equiv) was then added and the mixture was stirred for 12 h in the dark at 20 °C. The mixture was concentrated, then the residue was purified by chromatography on silica (eluent dichloromethane then CH_2Cl_2:MeOH 95:5) to provide the compound as a yellow solid.

3. Synthetic procedures

3.1 2-Azido-2-methylpropionic acid N_3AibOH

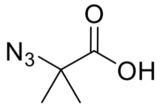

2-Bromo-2-methylpropionic acid (15.0 g, 89.9 mmol) was dissolved in dry DMF (60 mL). To this solution sodium azide (8.75 g, 134.7 mmol) was slowly added and the resulting mixture stirred at room temperature for 72 h under a nitrogen atmosphere. The reaction mixture was diluted with H_2O (40 mL) forming a clear solution and acidified to pH = 2 with 1 M HCl (30 mL) and extracted with *tert*-butyl methyl ether (3 × 50 mL). The organic layer was washed with 1 M HCl (4 × 20 mL), dried over $MgSO_4$, filtered and the solvent removed under reduced pressure (the compound is moderately volatile at low pressure) to yield the compound as a colorless oil, which was used in the next step without further purification (11.34 g, 87.9 mmol, 98%). **^{1}H NMR** (400 MHz, $CDCl_3$) δ 1.46 (s, 6 H, 2 × CH_3); **^{13}C NMR** (100 MHz, $CDCl_3$) δ 178.9 (*C*=O), 62.8 (*C*), 24.3 (2 x CH_3). Spectroscopic data is consistent with the reported data in the literature.[1]

3.2 N_3AibO^{t}Bu

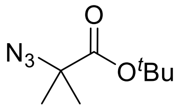

Sodium azide (6.65 g, 100.8 mmol) was dissolved in dry DMF (50 mL). To the resulting white suspension tert-butyl-2-bromo-2-methylpropionate (12.5 mL, 67.2 mmol) was added and the reaction mixture was stirred at room temperature for 72 h under a nitrogen atmosphere. The resulting white suspension was diluted with H_2O (40 mL) and acidified to pH = 2 with 1M HCl (30 mL) and extracted with tert-butyl methyl ether (3 × 50 mL). The organic layer was washed with 1M HCl (4 × 20 mL), dried over $MgSO_4$, filtered and the solvent removed (the compound is moderately volatile at low pressure) to yield the compound as a pale yellow oil, which was used in the next step without further purification (12.10 g, 64.8 mmol, 96%). **^{1}H NMR** (400 MHz, $CDCl_3$) δ 1.43 (s, 9 H, $(CH_3)_3C$, 1.35 (s, 6 H, 2 × CH_3); **^{13}C NMR** (100 MHz, $CDCl_3$) δ 171.8 (*C*=O), 82.4 ($C(CH_3)_3$), 63.4 ($C(CH_3)_2$), 27.9 (3C, $(CH_3)_3C$), 24.3 (2C, $(CH_3)_2C$). Spectroscopic data is consistent with the reported data in the literature.[1]

3.3 NH_2AibO^{t}Bu

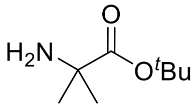

N_3AibO^{t}Bu (5.90 g, 30.75 mmol) was hydrogenated using procedure 1 and the product was isolated as a pale yellow oil (the compound is moderately volatile at low pressure). (4.82 g, 30.27 mmol, 98%). **^{1}H NMR** (400 MHz, $CDCl_3$) δ 1.43 (s, 9 H, $(CH_3)_3C$), 1.35 (s, 6 H, $(CH_3)_2C$); **^{13}C NMR** (100 MHz, $CDCl_3$) δ 177.4 (*C*=O), 80.6 ($C(CH_3)_3$), 55.0 ($C(CH_3)_2$), 27.9 (3C, $(CH_3)_3C$), 27.7 (2C, $(CH_3)_2C$). Spectroscopic data is consistent with the reported data in the literature.[1]

3.4 N_3Aib_2O^{t}Bu

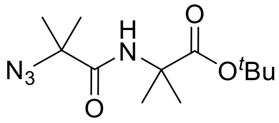

H_2N-AibO^{t}Bu (2.40 g, 15.1 mmol) was coupled following procedure 2 and the product was purified by flash chromatography (SiO_2/petroleum ether:EtOAc 85:15). The product was isolated as a pale yellow oil. (3.7 g, 14.1 mmol, 93%).**^{1}H NMR** (400 MHz, $CDCl_3$) *δ* 7.03 (s, 1 H, N*H*), 1.45 (s, 6 H, $(CH_3)_2C$), 1.44 (s, 6 H, $(CH_3)_2C$), 1.39 (s, 9 H, $(CH_3)_3C$); **^{13}C NMR** (100 MHz, $CDCl_3$) δ 173.5 (*C*=O), 171.1 (*C*=O), 81.7 (*C*$(CH_3)_3$), 56.7 (*C*$(CH_3)_2$), 27.8 (3C, $(CH_3)_3C$), 24.3 (4C, 2 x $(CH_3)_2C$). Spectroscopic data is consistent with the reported data in the literature.[1]

3.5 *$H_2NAib_2O^tBu$*

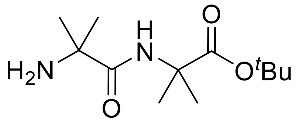

$N_3Aib_2O^tBu$ (1.92 g, 7.10 mmol) was hydrogenated following procedure 1 and the product was isolated as a white solid (1.56 g, 6.38 mmol, 90 %). **^{1}H NMR** (400 MHz, $CDCl_3$) *δ* 7.98 - 8.03 (m, 1 H, N*H*), 1.44 (s, 6H, $(CH_3)_2C$), 1.39 (s, 9 H, $(CH_3)_3C$), 1.28 (s, 6H, $(CH_3)_2C$); **^{13}C NMR** (100 MHz, $CDCl_3$) δ 176.0 (*C*=O), 174.0 (*C*=O), 81.2 (*C*$(CH_3)_3$), 56.2 (*C*$(CH_3)_2$), 54.8 (*C*$(CH_3)_2$), 29.0 (2C, $(CH_3)_2C$), 27.9 (3C, $(CH_3)_3C$), 24.5 (2C, $CH_3)_2C$). Spectroscopic data is consistent with the reported data in the literature.[1]

3.6 *$N_3Aib_3O^tBu$*

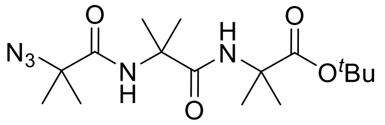

H_2N-Aib_2OtBu (3.41 g, 13.9 mmol) was coupled to N_3AibOH following procedure 2 and the product was purified by flash chromatography (SiO_2/petroleum ether:EtOAc 85:15). The product was isolated as a white solid (3.15 g, 11.6 mmol, 83 %). **TLC** Petroleum ether:EtOAc 85:15 R_f = 0.20; **^{1}H NMR** (400 MHz, $CDCl_3$) δ 7.14 (s, 1 H, N*H*), 6.92 (s, 1H, N*H*), 1.49 (s, 6 H, $(CH_3)_2C$), 1.47 (s, 6 H, $(CH_3)_2C$), 1.46 (s, 6 H, $(CH_3)_2C$), 1.39 (s, 9 H, $(CH_3)_3C$); **^{13}C NMR** (100 MHz, $CDCl_3$) δ 174.1 (*C*=O), 172.9 (*C*=O), 171.8 (*C*=O), 81.8 (*C*$(CH_3)_3$), 64.4 (*C*$(CH_3)_2$), 57.1 (*C*$(CH_3)_2$), 57.0 (*C*$(CH_3)_2$), 27.8 (3C, $(CH_3)_3C$), 24.9 (2C, $(CH_3)_2C$), 24.3 (2C, $(CH_3)_2C$), 24.0 (2C, $(CH_3)_2C$). Spectroscopic data is consistent with the reported data in the literature.[1]

3.7 *$NH_2Aib_3O^tBu$*

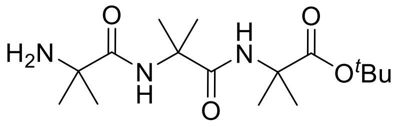

$N_3Aib_3O^tBu$ (0.50 g, 1.45 mmol) was hydrogenated following procedure 1 and the product was isolated as a white solid (0.42 g, 1.27 mmol, 88 %). **^{1}H NMR** (400 MHz, $CDCl_3$) δ 8.10 (s, 1 H, N*H*), 7.40 (s, 1 H, N*H*), 1.45 (s, 6H, 2 × CH_3), 1.42 (s, 6 H, 2 × CH_3), 1.37 (s, 9H, 3 × CH_3), 1.28 (s, 6 H, 2 × CH_3) ppm; **^{13}C NMR** (100 MHz, $CDCl_3$) δ 177.7 (*C*=O), 174.0 (*C*=O), 173.4 (*C*=O), 81.2 (*C*$(CH_3)_3$), 56.9 (*C*$(CH_3)_2$), 56.5 (*C*$(CH_3)_2$), 54.9 (*C*$(CH_3)_2$), 28.9 (3C, $(CH_3)_3C$), 27.8 (2C, $(CH_3)_2C$), 25.1 (2C, $(CH_3)_2C$), 24.2 (2C, $(CH_3)_2C$). Spectroscopic data is consistent with the reported data in the literature.[1]

3.8 *$N_3Aib_4O^tBu$*

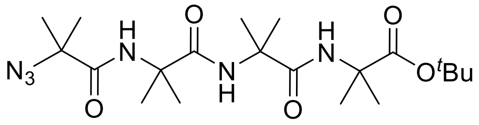

$NH_2Aib_3O^tBu$ (0.40 g, 1.21 mmol) was coupled to N_3AibOH following procedure 2 and the product was purified by flash chromatography (SiO_2/petroleum ether:EtOAc 85:15). The product was isolated as a white solid (0.40 g, 0.9 mmol, 75 %). **^{1}H NMR** (400 MHz, $CDCl_3$) δ 7.00 (s, 1H, N*H*), 6.96 (s, 1H, N*H*), 6.51 (s, 1H, N*H*), 1.53 (s, 6H, $(CH_3)_2C$), 1.52 (s, 6H, $(CH_3)_2C$), 1.51 (s, 6H, $(CH_3)_2C$), 1.48 (s, 6H, $(CH_3)_2C$), 1.44 (s, 9H, $(CH_3)_3C$). **^{13}C NMR** (101 MHz, $CDCl_3$) δ 173.9 (*C*=O), 173.1 (*C*=O), 172.5 (*C*=O), 172.4 (*C*=O), 81.1 ($C(CH_3)_3$), 64.3 ($C(CH_3)_2$), 57.2 ($C(CH_3)_2$), 57.0 ($C(CH_3)_2$), 56.7 ($C(CH_3)_2$), 28.0 (3C, $(CH_3)_3C$), 25.3 ($(CH_3)_2C$), 25.1 ($(CH_3)_2C$), 24.5 ($(CH_3)_2C$), 24.5 ($(CH_3)_2C$). Spectroscopic data is consistent with the reported data in the literature.[2]

3.9 $NH_2Aib_4O^tBu$

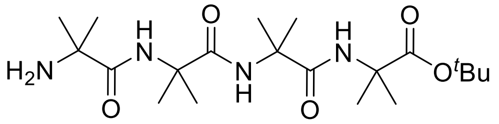

$N_3Aib_4O^tBu$ (0.25 g, 0.58 mmol) was hydrogenated following procedure 1 and the product was isolated as a white solid (0.18 g, 0.43 mmol, 74 %) **^{1}H NMR** (400 MHz, $CDCl_3$) δ 8.16 (s, 1H, N*H*), 7.26 (s, 1H, N*H*), 6.84 (s, 1H, N*H*), 1.51 - 1.48 (bs, 6H, 2 x CH_3C), 1.48 – 1.45 (bs, 18H, 6 x CH_3C), 1.44 – 1.41 (bs, 9H, 3 x CH_3C). **^{13}C NMR** (101 MHz, $CDCl_3$) δ 175.4 (*C*=O), 174.3 (*C*=O), 173.8 (*C*=O), 173.6 (*C*=O), 81.0 ($C(CH_3)_3$), 56.9 ($C(CH_3)_2$), 56.7 ($C(CH_3)_2$), 56.5 ($C(CH_3)_2$), 55.9 ($C(CH_3)_2$), 27.7 (3C, $(CH_3)_3C$), 26.6 (2C, $(CH_3)_2C$), 25.0 (2C, $(CH_3)_2C$), 24.6 (2C, $(CH_3)_2C$), 24.3 (2C, $(CH_3)_2C$). Spectroscopic data is consistent with the reported data in the literature.[1]

3.10 $N_3Aib_5O^tBu$

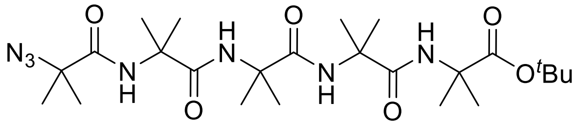

$NH_2Aib_4O^tBu$ (0.18 g, 0.43 mmol) was coupled to N_3AibOH following procedure 2 and the product was isolated as a white solid (0.161 g, 0.3 mmol, 70 %) after flash chromatography (SiO_2/petroleum ether:EtOAc 85:15). **^{1}H NMR** (400 MHz,$CDCl_3$) δ 7.18 (s, 1H, N*H*), 7.09 (s, 1H, N*H*), 6.86 (s, 1H, N*H*), 6.14 (s, 1H, N*H*), 1.53 (s, 6H, 2 × CH_{3Aib}),1.48 (s, 12H, 4 × CH_{3Aib}), 1.45 (s, 6H, 2 × CH_{3Aib}), 1.42(s, 15H, 2 x $CH_{3\ Aib}$, $(CH_3)_3C$);**^{13}C NMR** (100 MHz, $CDCl_3$) δ 173.9 (*C*=O), 173.5 (*C*=O), 172.8 (*C*=O), 172.7 (*C*=O), 172.4 (*C*=O), 80.0 ($C(CH_3)_3$), 64.0 (C_{Aib}), 56.9 (C_{Aib}), 56.8 (C_{Aib}), 56.8 (C_{Aib}), 56.1 (C_{Aib}), 27.8 (3C, $(CH_3)_3C$), 25.3 (4C, 2 x $(CH_3)_2C_{Aib}$), 24.8 (2C, $(CH_3)_2C_{Aib}$), 24.7 (2C, $(CH_3)_2C_{Aib}$), 24.3 (2C, $(CH_3)_2C_{Aib}$). Spectroscopic data is consistent with the reported data in the literature.[3]

3.11 $NH_2Aib_5O^tBu$

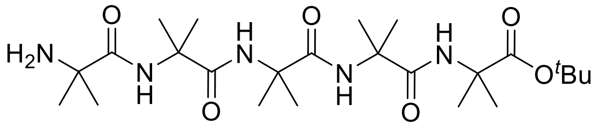

N_3Aib_5OtBu (0.300 g, 0.68 mmol) was hydrogenated following procedure 1 and the product was isolated as a white solid (0.282 g, 0.68 mmol, quantitative). **^{1}H NMR** (400 MHz, Acetone d6) δ 8.96 (s, 3H, NH_3^+), 8.61 (s, 1H, N*H*), 7.97 (s, 1H, N*H*), 7.67 (s, 1H, N*H*), 7.58 (s, 1H, N*H*), 1.78 (s, 6H, 2 x $(CH_3)C$), 1.50 (s, 6H, 2 x $(CH_3)C$), 1.48 (s, 6H, 2 x $(CH_3)C$), 1.46 – 1.43 (bs, 9H, 3 x $(CH_3)C$), 1.43 – 1.40 (bs, 9H, 3 x $(CH_3)C$). **^{13}C NMR** (101 MHz, Acetone) δ 175.3 (*C*=O), 175.3 (*C*=O), 175.2 (*C*=O), 175.0 (*C*=O), 172.7 (*C*=O), 81.4 ($C(CH_3)_3$), 58.7 ($C(CH_3)_2$), 58.2 ($C(CH_3)_2$), 57.6 ($C(CH_3)_2$), 57.3 ($C(CH_3)_2$), 56.9 ($C(CH_3)_2$), 28.0 (3C, $(CH_3)_3C$), 25.5 (2C, $(CH_3)_2C$), 25.3 (2C, $(CH_3)_2C$), 25.2 (2C, $(CH_3)_2C$), 24.9 (2C, $(CH_3)_2C$), 24.3 (2C, $(CH_3)_2C$). Spectroscopic data is consistent with the reported data in the literature.[4]

3.12 *Cbz-(L-αMeVal)-Aib$_4$O^{t}Bu*

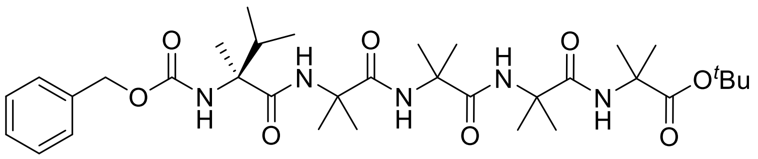

To a solution of Cbz-L-α-methylvaline (Cbz-(L-αMeVal)OH, 0.2 g, 1.26 mmol) in dry dichloromethane (4 mL) at 0 °C under nitrogen atmosphere was added pyridine (0.28 mL, 3.5 mmol, 2.8 equiv.) then cyanuric fluoride (0.15 mL, 1.76 mmol, 1.4 equiv) and the mixture was stirred at 0 °C for one hour then at 20 °C for two hours. The mixture was washed with ice-cold water then the organic phase was dried over $MgSO_4$, concentrated, to afford the acyl fluoride that was used in the next step without further purification. To a stirred solution of H_2NAib$_4$O^{t}Bu (0.240 g, 0.578 mmol) and DIPEA (0.17 mL, 1.16 mmol) in dry dichloromethane (10 mL) was added the solution of Cbz-(L-αMeVal)F (0.186 g, 1.15 mmol) in dry dichloromethane (5 mL) and the reaction stirred at 20 °C under nitrogen for 3 days. The mixture was concentrated, then ethyl acetate (50 mL) was added and the organic phase was washed with aqueous 5 % $KHSO_4$ (3 x 30 mL), water (3 x 30 mL), then aqueous saturated $NaHCO_3$ (3 x 30 mL). The organic phase was dried ($MgSO_4$), concentrated under reduced pressure. The white solid was triturated in diethylether then filtered on a filter paper. The solid on the filter paper was dissolved in methanol, concentration of methanol under reduced pressure provided the product as a white solid (0.282 g, 0.51 mmol, 88 %). **^{1}H NMR** (400 MHz, $CDCl_3$) δ 7.38 – 7.15 (m, 9H, 3N*H*, 5 x C*H*$_{Ar}$), 6.21 (s, 1H, N*H*$_{Cbz}$), 5.39 (s, 1H, NH), 5.08 (d, *J* = 12.2 Hz, 1H, C*H*$_2$Ph), 4.93 (d, *J* = 12.2 Hz, 1H, C*H*$_2$Ph), 1.88 – 1.79 (m, 1H, C*H*(CH$_3$)$_2$), 1.44 (s, 3H, C*H*$_3$), 1.39 (s, 3H, C*H*$_3$), 1.37 (s, 3H, C*H*$_3$), 1.36 (s, 3H, C*H*$_3$), 1.35 (s, 6H, 2 x C*H*$_3$), 1.31 (d, *J* = 3.0 Hz, 15H, 2 x C*H*$_3$, (C*H*$_3$)$_3$C), 1.11 (s, 3H, C*H*$_3$), 0.87 (d, *J* = 6.7 Hz, 3H, (C*H*$_3$)$_2$CH)), 0.83 (d, *J* = 6.8 Hz, 3H, (C*H*$_3$)$_2$CH)). **^{13}C NMR** (101 MHz, $CDCl_3$) δ 174.2 (*C*=O), 174.1 (*C*=O), 173.9 (*C*=O), 173.8 (*C*=O), 172.6 (*C*=O), 156.2 (*C*=O$_{Cbz}$), 136.2 (*C*), 128.8 (2C, 2 x *C*H$_{Ph}$), 128.7 (*C*H$_{Ph}$), 128.4 (2C, 2 x *C*H$_{Ph}$), 79.8 (*C*(CH$_3$)$_3$), 67.5 (*C*H$_2$Ph), 63.1 (*C*$_{Val}$), 57.0 (*C*$_{Aib}$), 56.8 (*C*$_{Aib}$), 56.8 (*C*$_{Aib}$), 56.1 (*C*$_{Aib}$), 35.6 (*C*H(CH$_3$)$_2$), 28.0 (3C, (*C*H$_3$)$_3$C), 27.3 (*C*H$_{3Aib}$), 27.0 (*C*H$_{3Aib}$), 26.9 (*C*H$_{3Aib}$), 25.7 (*C*H$_{3Aib}$), 24.2 (*C*H$_{3Aib}$), 23.9 (*C*H$_{3Aib}$), 23.6 (*C*H$_{3Aib}$), 23.5 (*C*H$_{3Aib}$), 17.7 (*C*H$_{3\ Val}$), 17.4 ((*C*H$_3$)$_2$CH$_{Val}$), 17.3 ((*C*H$_3$)$_2$CH$_{Val}$). **FTIR (neat)** ν_{max} = 3421, 3341, 3307, 3232, 2980, 2940, 2237, 1729, 1697, 1683, 1667, 1638, 1537, 1501, 1456, 1384, 1363, 1308, 1269, 1224, 1213, 1147, 1071, 1038 cm^{-1} . **MP** 215-216 °C. Spectroscopic data is consistent with the reported data in the literature.[5]

3.13 *Cbz-(L-αMeVal)-Aib$_5$O^{t}Bu*

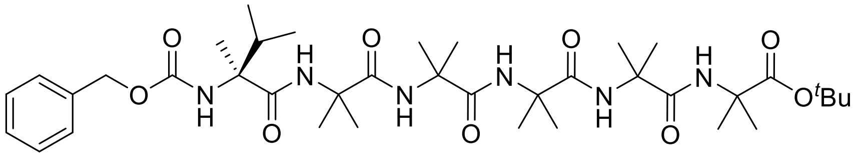

To a solution of Cbz-L-α-methylvaline (Cbz-(L-αMeVal)OH, 0.2 g, 1.26 mmol) in dry dichloromethane (4 mL) at 0 °C under inert atmosphere was added pyridine (0.28 mL, 3.5 mmol, 2.8 equiv.) then cyanuric fluoride (0.15 mL, 1.76 mmol, 1.4 equiv) and the mixture was stirred at 0 °C for one hour then at 20 °C for two hours. The mixture was washed with ice-cold water then the organic phase was dried over $MgSO_4$, concentrated, to afford the acyl fluoride that was used in the next step without further purification. The residue was dissolved in dry dichloromethane (10 mL), then H_2NAib$_5$O^{t}Bu (0.261 g, 0.63 mmol, 0.5 equiv) was added, followed by diisopropylethylamine (0.18 mL, 1.26 mmol), the resulting mixture was stirred at 20 °C under inert atmosphere for 3 days. After concentration, ethyl acetate (50 mL) was added, the organic phase was extracted with $KHSO_4$ aqueous (5 %) (3 x 30 mL), then with water (3 x 30 mL), then with $NaHCO_3$ aqueous (3 x 30 mL). The organic phase was dried ($MgSO_4$), concentrated and the residue was purified by chromatography on silica (eluent petroleum ether : ethyl acetate 1:3) to afford the product (0.14 g, 0.252

mmol, 40 %). **^{1}H NMR** (400 MHz, $CDCl_3$) δ 7.57 (s, 1H, N*H*), 7.46 (s, 1H, N*H*), 7.37 (s, 6H, N*H*, 5 x C*H*$_{Ph}$), 7.27 (s, 1H, N*H*), 6.28 (s, 1H, N*HCbz*), 5.28 (s, 1H, N*H*), 5.18 (d, *J* = 12.2 Hz, 1H, C*H*$_2$Ph), 5.03 (d, *J* = 12.2 Hz, 1H, C*H*$_2$Ph), 1.93 (m, 1H, C*H*(CH_3)$_2$), 1.52 – 1.45 (m, 18H, 6 x C*H*$_{3\ Aib}$), 1.43 (m, 21 H, 7 x C*H*$_3$), 1.20 (s, 3H, C*H*$_3$), 0.98 (d, *J* = 6.8 Hz, 3H, (C*H*$_3$)$_2$CH), 0.95 (d, *J* = 6.8 Hz, 3H, (C*H*$_3$)$_2$CH). **^{13}C NMR** (101 MHz, $CDCl_3$) δ 175.0 (*C*=O), 174.3 (*C*=O), 174.2 (*C*=O), 174.1 (*C*=O), 173.7 (*C*=O), 172.7 (*C*=O), 156.2 (*C*=O$_{Cbz}$), 136.0 (*C*), 128.9 (2C, 2 x *C*H$_{Ph}$), 128.8 (*C*H$_{Ph}$), 128.4 (2C, 2 x *C*H$_{Ph}$), 79.8 (*C*(CH_3)$_3$), 67.7 (*C*H$_2$Ph), 63.2 (*C*$_{MeVal}$), 57.0 (*C*$_{Aib}$), 56.9 (*C*$_{Aib}$), 56.8 (2C, 2 x *C*$_{Aib}$), 56.1 (*C*$_{Aib}$), 35.8 (*C*H(CH_3)$_2$), 28.0 (3C, (*C*H$_3$)$_3$C), 27.4 (*C*H$_{3Aib}$), 27.0 (*C*H$_{3Aib}$), 26.9 (*C*H$_{3Aib}$), 26.8 (*C*H$_{3Aib}$), 25.6 (*C*H$_{3Aib}$), 24.3 (*C*H$_{3Aib}$), 23.9 (*C*H$_{3Aib}$), 23.7 (*C*H$_{3Aib}$), 23.5 (2C, 2 x *C*H$_{3Aib}$), 17.8 (*C*H$_{3\ Val}$), 17.4 ((*C*H$_3$)$_2$CH$_{Val}$), 17.3 ((*C*H$_3$)$_2$CH$_{Val}$). **FTIR (neat)** ν_{max} = 3310, 2981, 2935, 1656, 1519, 1451, 1374, 1262, 1145, 591 cm^{-1}. **HR – MS** (ESI, positive ion mode) – *m/z* for $[C_{38}H_{62}N_6O_9Na]^+$ 769.4470, found 769.4436. **MP** 212-214 °C.

3.14 Cbz-(L-αMeVal)$_2$-Aib$_4$O^{t}Bu

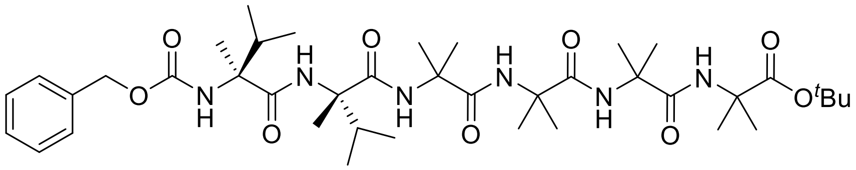

To a solution of Cbz-(L-αMeVal)Aib$_4$OtBu (280 mg 0.229 mmol) dissolved in ethanol was added palladium on carbon (10 % ww). The suspension was placed under nitrogen atmosphere by operating a series of vacuum/nitrogen cycles inside the flask, then hydrogen atmosphere was established by placing the inside of the flask under vacuum then under hydrogen. The reaction was stirred at 20 °C until consumption of the starting material (the reaction was monitored by TLC with an eluent of dichloromethane : methanol 9:1 using ninhydrin stain). At the completion of the reaction, the hydrogen atmosphere in the flask was replaced by nitrogen via vacuum/nitrogen cycle and the suspension was filtered on Celite. After rinsing the celite with ethanol, the organic phase was concentrated to give H$_2$N(L-αMeVal)Aib$_4$OtBu that required no further purification (121 mg, 2.29 mmol). The resulting compound was dissolved in dry dichloromethane (5 mL) and DIPEA (0.08 mL, 0.457 mmol) was added, then a solution of Cbz-(L-αMeVa)F (0.122 g, 0.457 mmol) in dry dichloromethane (1 mL) was added. The reaction was stirred at 20°C under inert atmosphere for 5 d. The resulting mixture as concentrated under reduced pressure, the residue was dissolved in EtOAc (10 mL) and washed with 5% $KHSO_4$ aqueous solution (2 × 10 mL), then with aqueous saturated $NaHCO_3$ solution (2 × 10 mL) then with brine (10 mL). The organic phase was dried ($MgSO_4$) then concentrated under reduced pressure. Purification by column chromatography on silica (eluent CH_2Cl_2:MeOH; 99:1 to 95:5) gave the product as a white solid (158 mg, 0.205 mmol, 90%). **^{1}H NMR** (400 MHz, $CDCl_3$) *δ* 7.66 (s, 1H, N*H*), 7.48 (s, 1H, N*H*), 7.38 (s, 1H, N*H*), 7.35 (s, 1H, N*H*), 7.33 – 7.25 (m, 5H, 5 x C*H*$_{Ph}$), 6.31 (s, 1H, N*H*$_{aMeVal}$), 5.84 (s, 1H, N*H*$_{aMeVal}$), 5.10 (d, *J* = 12.1 Hz, 1H, C*H*$_2$Ph$_{AB}$), 4.93 (d, *J* = 12.2 Hz, 1H, C*H*$_2$Ph$_{AB}$), 1.85 (sept, *J* = 6.9 Hz, 1H, C*H*(CH_3)$_2$), 1.48 (s, 3H, C*H*$_3$), 1.44 – 1.41 (m, 7H, 2 x C*H*$_3$C, C*H*(CH_3)$_2$), 1.41 – 1.38 (m, 9H, 3 x C*H*$_3$C), 1.37 – 1.35 (m, 9H, 3 x C*H*$_3$C), 1.35 – 1.32 (s, 9H, 3 x C*H*$_3$C), 1.30 (s, 3H, C*H*$_3$C), 0.90 (d, *J* = 6.9 Hz, 3H, C*H*$_3$CH), 0.88 (d, *J* = 6.9 Hz, 3H, C*H*$_3$CH), 0.69 (d, *J* = 6.7 Hz, 6H, 2 x C*H*$_3$CH). **^{13}C NMR** (101 MHz, $CDCl_3$) *δ* 175.2 (*C*=O), 174.3 (*C*=O), 174.3 (2C, 2 x *C*=O), 172.5 (*C*=O$_{aMeVal}$), 172.2 (*C*=O$_{aMeVal}$), 156.5 (*C*=O$_{Cbz}$), 136.0 (*C*$_{Ph}$), 128.7 (2C, 2 x *C*H$_{Ph}$), 128.6 (2C, 2 x *C*H$_{Ph}$), 128.6 (*C*H$_{Ph\ para}$), 79.7 (*C*(CH_3)$_3$), 67.5 (*C*H$_2$Ph), 63.5 (*C*$_{aMeVal}$), 62.3 (*C*$_{aMeVal}$), 56.9 (2C, 2 x *C*$_{Aib}$), 56.7 (*C*$_{Aib}$), 56.1 (*C*$_{Aib}$), 35.9 (*C*H(CH_3)$_2$), 35.7 (*C*H(CH_3)$_2$), 27.9 (3C, (*C*H$_3$)$_3$C), 27.8 (*C*H$_3$C$_{Aib}$), 27.4 (*C*H$_3$C$_{Aib}$), 27.3 (*C*H$_3$C$_{Aib}$), 25.8 (*C*H$_3$C$_{Aib}$), 23.9 (*C*H$_3$C$_{Aib}$), 23.2 (*C*H$_3$C$_{Aib}$), 23.0 (*C*H$_3$C$_{Aib}$), 22.9 (*C*H$_3$C$_{Aib}$), 18.2 (*C*H$_3$C$_{aMeVal}$), 18.0 (*C*H$_3$C$_{aMeVal}$), 17.5 (*C*H$_3$CH$_{aMeVal}$), 17.3(*C*H$_3$CH$_{aMeVal}$), 17.0 (*C*H$_3$CH$_{aMeVal}$), 17.0 (*C*H$_3$CH$_{aMeVal}$). Spectroscopic data is consistent with the reported data in the literature.[6]

3.15 N_3Aib_4OH

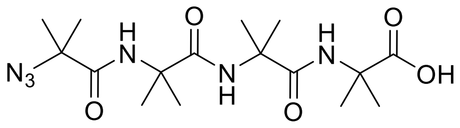

Following general procedure 3 with $N_3Aib_4O^tBu$ (0.7 g, 1.59 mmol) led to N_3Aib_4OH as a white solid (0.61 g, quantitative). **^{1}H NMR** (400 MHz, DMSO) δ 7.85 (s, 1H, N*H*), 7.46 (s, 1H, N*H*), 7.26 (s, 1H, N*H*), 1.45 (s, 6H, $(CH_3)_2C$), 1.35 (s, 6H, $(CH_3)_2C$), 1.34 (s, 6H, $(CH_3)_2C$), 1.29 (s, 6H, $(CH_3)_2C$). **^{13}C NMR** (101 MHz, DMSO) δ 175.6 (*C*=O), 173.3 (*C*=O), 172.6 (*C*=O), 171.9 (*C*=O), 63.6 ($C(CH_3)_2$), 56.4 ($C(CH_3)_2$), 55.6 ($C(CH_3)_2$), 54.8 ($C(CH_3)_2$), 24.7 (2C, $(CH_3)_2C$), 24.5 (2C, $(CH_3)_2C$), 24.4 (2C, $(CH_3)_2C$), 23.9 (2C, $(CH_3)_2C$). **^{1}H-NMR** (400 MHz, $CDCl_3$) δ 7.37 (bs, 1H, N*H*), 6.92 (s, 1H, N*H*), 6.27 (s, 1H, N*H*), 1.57 (s, 6H, $(CH_3)_2C$), 1.53 (s, 6H, $(CH_3)_2C$), 1.49 (s, 6H, $(CH_3)_2C$), 1.46 (s, 6H, $(CH_3)_2C$). **^{13}C-NMR** (101 MHz, $CDCl_3$) δ 177.4 (*C*=O), 174.9 (*C*=O), 174.3 (*C*=O), 173.3 (*C*=O), 64.0 ($C(CH_3)_2$), 57.2 ($C(CH_3)_2$), 57.1 ($C(CH_3)_2$), 57.1 ($C(CH_3)_2$), 25.1 (2C, $(CH_3)_2C$), 24.9 (2C, $(CH_3)_2C$), 24.8 (2C, $(CH_3)_2C$), 24.4 (2C, $(CH_3)_2C$). Spectroscopic data is consistent with the reported data in the literature.[2]

3.16 Cbz-(L-αMeVal)-Aib_4OH

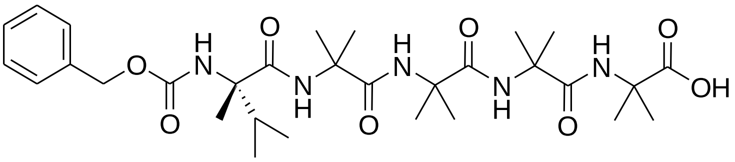

Following general procedure 3 with Cbz-(L-αMeVal)Aib_4O^tBu (0.28 g, 0.5 mmol) (the mixture was stirred at 20 °C for 5 hours, completion of the reaction was monitored by thin layer chromatography), the product was obtained as a white solid (0.25 g, 0.5 mmol, quantitative). **^{1}H NMR** (400 MHz, $CDCl_3$) δ 7.73 (s, 1H, N*H*), 7.53 (s, 1H, N*H*), 7.46 (s, 1H, N*H*), 7.36 – 7.24 (m, 5H, 5 x CH_{Ph}), 6.33 (s, 1H, N*H*), 5.28 (s, 1H,N*H*), 5.11 (d, *J* = 12.2 Hz, 1H, CH_2Ph), 4.97 (d, *J* = 12.2 Hz, 1H, CH_2Ph), 1.87 (p, *J* = 6.8 Hz, 1H, $CH(CH_3)_2$), 1.53 (s, 3H, $(CH_3)C$), 1.50 (s, 3H, $(CH_3)C$), 1.44 (s, 3H, $(CH_3)C$), 1.41 (s, 3H, $(CH_3)C$), 1.36 (s, 3H, $(CH_3)C$), 1.35 (s, 3H, $(CH_3)C$), 1.34 (s, 3H, $(CH_3)C$), 1.33 (s, 3H, $(CH_3)C$), 1.13 (s, 3H, $(CH_3)C$), 0.91 (d, *J* = 6.8 Hz, 3H, $(CH_3)CH$), 0.87 (d, *J* = 6.8 Hz, 3H, $(CH_3)CH$). **^{13}C NMR** (101 MHz, $CDCl_3$) δ 188.8 (CO_2H), 176.4 (*C*=O), 175.7 (*C*=O), 174.0 (*C*=O), 173.0 (*C*=O), 156.1 ($C{=}O_{Cbz}$), 136.0 (C_{Ph}), 128.8 (2C, 2 x CH_{Ph}), 128.7 (CH_{Ph}), 128.1 (2C, 2 x CH_{Ph}), 67.5 ($C(CH_3)(i\text{Pr})$), 63.0 (CH_2Ph), 57.7 ($C(CH_3)_2$), 57.0 ($C(CH_3)_2$), 56.9 ($C(CH_3)_2$), 56.6 ($C(CH_3)_2$), 35.5 ($CH(CH_3)_2$), 26.6 (2 x CH_3), 26.5 (CH_3), 25.6 (CH_3), 24.6 (CH_3), 23.9 (CH_3), 23.5 (CH_3), 23.2 (CH_3), 17.4 (CH_3), 17.3 (CH_3), 17.1 (CH_3). Spectroscopic data is consistent with the reported data in the literature.[6]

3.17 Cbz-(L-αMeVal)-Aib_5OH

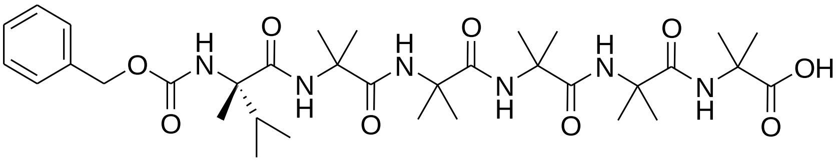

Following general procedure 3, a solution of Cbz-(L-αMeVal)Aib_5OtBu (0.12 g, 0.16 mmol) in CH_2Cl_2 (4 mL) and trifluoroacetic acid (2 mL) was stirred for 5h. After completion, the reaction mixture was concentrated under reduced pressure to yield Cbz-L-αMeValAib_5OH as a white solid (93 mg, 0.135 mmol, 84 %). **^{1}H NMR** (400 MHz, $CDCl_3$) δ 7.76 (s, 1H, N*H*), 7.72 (s, 1H, N*H*), 7.62 (s, 1H, N*H*), 7.52 (s, 1H, N*H*), 7.41 – 7.30 (m, 5H, 5 x CH_{Ph}), 6.54 (s, 1H, N*H*), 5.51 (s, 1H, N*H*), 5.18 (d, *J* = 12.3 Hz, 1H, CH_2Ph), 5.04 (d, *J* = 12.2 Hz, 1H, CH_2Ph), 1.97 (p, *J* = 6.9 Hz, 2H, $CH(CH_3)_2$), 1.61 (s, 3H, $(CH_3)C$), 1.58 (s, 3H, $(CH_3)C$), 1.52 – 1.46 (m, 9H, $(CH_3)_3C$), 1.44 (s, 3H, $(CH_3)C$), 1.43 (s, 3H, $(CH_3)C$), 1.42 (s, 3H, $(CH_3)C$), 1.40 (s, 3H, $(CH_3)C$), 1.39 (s, 3H, $(CH_3)C$), 1.21 (s, 3H, $(CH_3)C$), 0.99 (d, *J* = 6.7 Hz, 3H, $(CH_3)CH$), 0.94 (d, *J* = 6.8 Hz, 3H, $(CH_3)CH$). **^{13}C NMR** (101 MHz, $CDCl_3$) δ 176.8

(C=O), 176.75 (C=O), 175.9 (C=O), 175.8 (C=O), 175.1 (C=O), 174.3 (C=O), 156.5 ($C=O_{Cbz}$), 136.9 (C), 128.7 (2C, 2 x CH), 128.2 (CH), 127.8 (2C, 2 x CH), 67.0 ($C(CH_3)(i\text{Pr})$), 63.0 (CH_2Ph), 57.4 ($C(CH_3)_2$), 56.9 ($C(CH_3)_2$), 56.8 ($C(CH_3)_2$), 56.7 (2C, 2 x $C(CH_3)_2$), 34.8 ($CH(CH_3)_2$), 26.5 (4C, 3 x CH_3), 26.1 (CH_3), 24.5 (CH_3), 23.7 (CH_3), 23.4 (CH_3), 23.2 (2C, 2 x CH_3), 17.5 (CH_3), 17.3 (CH_3), 16.7 (CH_3). **FTIR (neat)** ν_{max} = 3289 2985 2939 1652 1521 1451 1375 1220 1162 663 cm^{-1}. **HR – MS** (ESI, positive ion mode) – *m/z* for $[C_{34}H_{54}N_6O_9Na]^+$ 713.3844, found 713.3816. **MP** 219-221 °C.

3.18 Cbz-(L-αMeVal)$_2$-Aib$_4$OH

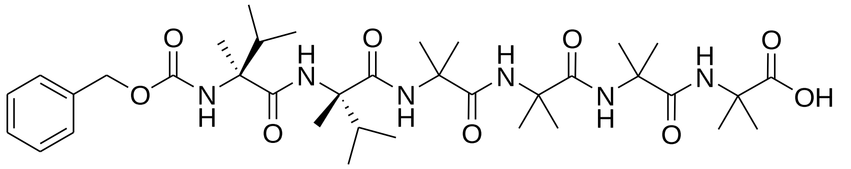

Following general procedure 3 with a solution of Cbz-(L-αMeVal)$_2$Aib$_4$OtBu (0.153 g, 0.198 mmol) in dry dichloromethane and trifluoroacetic acid at 20 °C for 18 h, the product was obtained after purification by chromatography on silica (eluent CH_2Cl_2:MeOH 95:5 to 90:10) as a white solid (0.13 g, 0.18 mmol, 92%). **^{1}H NMR** (400 MHz, $CDCl_3$) δ 7.89 (s, 1H, NH), 7.81 (s, 1H, NH), 7.64 (s, 1H, NH), 7.57 (s, 1H, NH), 7.36 (m, 5H, 5 x CH_{Ph}), 7.26 (s, 1H, NH), 6.48 (s, 1H, NH), 5.55 (d, J = 11.4 Hz, 1H, CH_2Ph), 5.17 (d, J = 12.1 Hz, 1H, CH_2Ph), 5.02 (d, J = 12.1 Hz, 1H), 1.91 (spt, J = 6.8 Hz, 1H, CH($CH_3)_2$), 1.62 (s, 3H, CH_3), 1.57 (s, 3H, CH_3), 1.54 (sept, J = 7.2 Hz, 1H, CH($CH_3)_2$), 1.49 (s, 3H, CH_3), 1.48 (s, 6H, CH_3), 1.45 (s, 6H, CH_3), 1.42 (s, 6H, CH_3), 1.38 (s, 3H, CH_3), 0.99 (d, J = 6.7 Hz, 3H, (CH_3)$_2$CH), 0.94 (d, J = 6.8 Hz, 3H, (CH_3)$_2$CH), 0.84 – 0.71 (m, 6H, 2 x (CH_3)$_2$CH). **^{13}C NMR** (101 MHz, $CDCl_3$) δ 176.9 (C=O), 175.9 (C=O), 175.6 (C=O), 175.6 (C=O), 173.0 (C=O), 172.6 (C=O), 156.5 ($C=O_{Cbz}$), 136.1 (C_{Ph}), 128.4 (2C, 2 x CH_{Ph}), 128.3 (CH_{Ph}), 128.2 (2C, 2 x CH_{Ph}), 67.1 (CH_2Ph_{Cbz}), 63.3 (C), 62.2 (C), 56.7 (2C), 56.7 (C), 56.4 (C), 35.5 (CH($CH_3)_2$), 35.5 (CH($CH_3)_2$), 26.9 (CH_3), 26.8 (CH_3), 26.8 (CH_3), 24.7 (CH_3), 24.6 (CH_3), 22.7 (CH_3), 22.6 (CH_3), 22.5 (CH_3), 17.8 (CH_3), 17.7 (CH_3), 17.1 (CH_3), 16.8 (CH_3), 16.7 (CH_3), 16.7 (CH_3). Spectroscopic data is consistent with the reported data in the literature.[6]

3.19 N-Mesitylimidazole

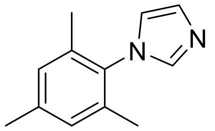

Following a reported procedure,[7] to 2,4,6-trimethylaniline (3.1 mL, 22 mmol) dissolved in methanol (30 mL) was added an aqueous glyoxal solution (40 % w in water) (3.2 mL, 22 mmol) and the mixture was stirred at 20 °C for 16 h, forming a yellow suspension. A suspension of ammonium chloride (2.4 g, 44 mmol) mixed with formaldehyde (37 % aqueous) (3.6 mL, 44 mmol) in methanol (31 mL) was added to the yellow suspension, and the resulting mixture was heated to reflux for 1 h. Phosphoric acid (85 % H_3PO_4, 2 mL) was then added and the mixture was heated at reflux for 8 h. The mixture was concentrated under vacuum, water was added, followed with solid potassium hydroxide until pH 8 was reached. The mixture was extracted using dichloromethane, the combined organic phases were washed with water, dried using sodium sulphate, then concentrated. Recrystallisation from *n*-hexane afforded *N*-mesitylimidazole as a brown solid (2.13 g, 11.4 mmol, 52 %). **^{1}H NMR** (400 MHz, $CDCl_3$) δ 7.43 (d, J = 1.1 Hz, 1H, CH_{Imid}), 7.23 (d, J = 1.1 Hz, 1H, CH_{Imid}), 6.96 (s, 2H, 2 x CH_{Ph}), 6.89 (d, J = 1.1 Hz, 1H, CH_{Imid}), 2.33 (s, 3H, CH_3), 1.98 (s, 6H, 2 x CH_3). Spectroscopic data is consistent with the reported data in the literature.[7]

3.20 N-Phenylimidazole

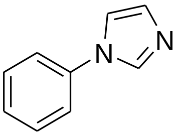

Following a reported procedure,[8] to a solution of bromobenzene (1.44 mL, 13.5 mmol) in dimethylsulfoxide (30 mL) were added imidazole (1.38 g, 20.3 mmol), copper (I) oxide Cu_2O (192 mg, 1.35 mmol), then potassium hydroxide (1.5 g, 16 mmol). The mixture was heated under inert atmosphere at 130 °C for 3 days. After cooling down to 20 °C, water (40 mL) then ethyl acetate (40 mL) were added and the mixture was filtered using a Büchner funnel. The filtrate was extracted with ethyl acetate (3 x 30 mL), the combined organic phases were washed with brine (30 mL), dried (Na_2SO_4), concentrated under vacuum. The residue was purified using silica gel chromatography (eluent/ ethyl acetate 100 %) to afford the *N*-phenylimidazole as a white powder (1.5 g, 10.4 mmol, 77 %). **^{1}H NMR** (400 MHz, $CDCl_3$) δ 7.88 (s, 1H, CH_{Imid}), 7.51 (d, *J* = 7.1 Hz, 2H, 2 x CH_{Ph}), 7.46 – 7.35 (m, 3H, 3 x CH_{Ph}), 7.31 (s, 1H, CH_{Imid}), 7.23 (s, 1H, CH_{Imid}). Spectroscopic data is consistent with the reported data in the literature.[8]

3.21 1-(2-Aminoethyl)-3-(phenyl)-1H-imidazol-3-ium bromide, hydrobromide salt, 5

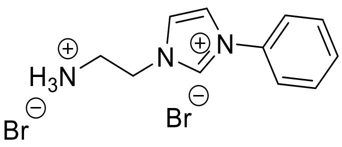

To *N*-phenylimidazole (1.5 g, 10.0 mmol) in acetonitrile (70 mL) was added 2-bromoethylammonium bromide (2.1 g, 13 mmol), the mixture was heated at 80 °C for 2 days. The solid was filtered on sintered funnel from hot acetonitrile. The resulting solid was recrystallized from ethanol to afford the compound as a white solid (1.98 g, 6.5 mmol, 66 %). TLC eluent: ethyl acetate 100 %. **^{1}H NMR** (400 MHz, DMSO) δ 9.98 (d, *J* = 1.7 Hz, 1H, CH_{im}), 8.39 (t, *J* = 1.9 Hz, 1H, CH_{im}), 8.35 – 8.10 (m, 3H, NH_3^+), 8.08 (t, *J* = 1.8 Hz, 1H, CH_{im}), 7.82 (dd, *J* = 7.9, 1.7 Hz, 2H, 2 x CH_{Ph}), 7.69 (dd, *J* = 8.6, 7.0 Hz, 2H, 2 x CH_{Ph}), 7.61 (dd, *J* = 8.6, 1.7 Hz, 1H, CH_{Ph}), 4.59 (t, *J* = 5.8 Hz, 2H, CH_2), 3.50 (t, *J* = 5.8 Hz, 2H, CH_2). **^{13}C NMR** (101 MHz, DMSO) δ 136.3 (C_{Ph}), 134.7 (CH_{Imid}), 130.2 (2C, 2 x CH_{Ph}), 129.9 (CH_{Ph}), 123.6 (CH_{Imid}), 121.8 (2C, 2 x CH_{Ph}), 121.2 (CH_{Im}), 46.8 ($CH_{2\ imid}$), 38.3 (CH_2NH_2). **FTIR (neat)** ν_{max} = 3355, 2921, 1628, 1511, 1263, 1201, 1049, 1017 cm^{-1} **HR – MS** (ESI, positive ion mode) – *m/z* for $[C_{11}H_{14}N_3]^+$ 188.1182, found 188.1189.

3.22 1-(2-Aminoethyl)-3-(2,4,6-trimethylphenyl)-1H-imidazol-3-ium bromide, HBr salt, 6

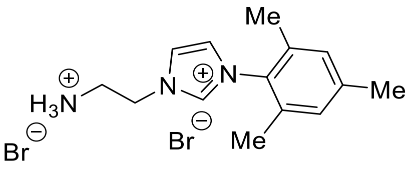

To *N*-mesitylimidazole (2.263 g, 12.2 mmol) in acetonitrile (70 mL) at 80 °C was added 2-bromoethylammonium bromide (2.5 g (3 × 833 mg), 12.2 mmol) in three portions over 3 hours. The mixture was heated at 80 °C for 2 days. The mixture was concentrated under vacuum, the residue was purified by silica gel chromatography (dry loading, eluent CH_2Cl_2 100 % then a gradient CH_2Cl_2: MeOH 9 : 1 to 1 : 1). The product required further purification by recrystallization in hot ethanol to provide the compound as a white solid (2.16 g, 9.39 mmol, 77 %). **^{1}H NMR** (400 MHz, DMSO) δ 9.51 (t, *J* = 1.6 Hz, 1H, CH_{Im}), 8.31 – 8.19 (m, 2H, NH_2), 8.18 (t, *J* = 1.8 Hz, 1H, CH_{Im}), 7.99 (t, *J* = 1.8 Hz, 1H, CH_{Im}), 7.15 (s, 2H, 2 x CH), 4.60 (t, *J* = 6.1 Hz, 2H, CH_2), 3.52 (t, *J* = 6.1 Hz, 2H, CH_2), 2.34 (s, 3H, CH_3), 2.08 (s, 6H, 2 x CH_3). **^{13}C NMR** (101 MHz, DMSO) δ 140.2

(CH_{im}), 138.0 (*C*), 134.4 (2C, 2 x *C*Me), 131.1 (*C*), 129.3 (2C, 2 x *C*H), 124.1 (CH_{im}), 123.3 (CH_{im}), 46.8 (CH_2), 37.9 (CH_2), 20.6 (CH_3), 17.2 (2C, 2 x CH_3). **FTIR (neat)** ν_{max} = 3290, 2929, 1628, 1263, 1017, 799 cm^{-1} **HR – MS** (ESI, positive ion mode) – *m/z* for $[C_{14}H_{20}N_3]^+$ 230.1657, found 230.1652.

3.23 1-(2-Aminoethyl)-3-(methyl)-1H-imidazol-3-ium bromide, hydrobromide salt, 7

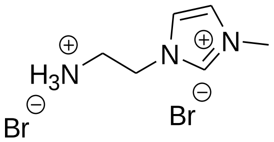

The compound was synthesised following a reported procedure.[9] **^{1}H NMR** (400 MHz, MeOD) δ 9.14 (d, *J* = 1.9 Hz, 1H, $NCHN_{Imid}$), 7.77 (q, *J* = 1.8 Hz, 1H, CH_{imid}), 7.68 (t, *J* = 1.8 Hz, 1H, CH_{imid}), 4.63 (t, *J* = 6.2 Hz, 2H, CH_2), 4.00 (s, 3H, CH_3), 3.58 (t, *J* = 6.2 Hz, 2H, CH_2). Spectroscopic data is consistent with the reported data in the literature.[9]

3.24 1-(2-Isobutyramidoethyl)-3-phenyl-1H-imidazol-3-ium bromide, Precursor S1

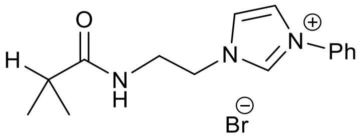

To a solution of 2-methylpropanoic acid (0.026 mL, 0.28 mmol) in dry dichloromethane (2 mL) was added EDC.HCl (108 mg, 0.56 mmol, 2 equiv) and triethylamine (0.08 mL, 0.56 mmol, 2 equiv) and the mixture was stirred at 20 °C over 4 hours then concentrated under reduced pressure. The residue was dissolved in dry acetonitrile (2 mL) and 1-(2-aminoethyl)-3-(phenyl)-1H-imidazol-3-ium bromide, hydrobromide salt **5** (112 mg, 0.4 mmol) was added followed with triethylamine (0.07 mL, 0.5 mmol). The suspension was stirred at 80 °C for 3 days after which the mixture was concentrated under reduced pressure, dissolved in dichloromethane, the organic phase was washed with saturated aqueous ammonium chloride, then with saturated sodium hydrogencarbonate, then with brine, dried (Na_2SO_4), concentrated, and the residue was purified by chromatography on silica (eluent: dichloromethane:methanol 9:1) to afford the product as an oil (20 mg, 0.08 mmol, 28 %). **^{1}H NMR** (400 MHz, MeOD) *δ* 9.67 (s, 1H, NC*H*N), 8.09 (d, *J* = 2.0 Hz, 1H, CH_{Imid}), 7.87 (d, *J* = 2.0 Hz, 1H, CH_{Imid}), 7.75 (dd, *J* = 8.3, 1.1 Hz, 2H, 2 x $CH_{Ph\ ortho}$), 7.70 – 7.58 (m, 3H, 3 x CH_{Ph}), 4.47 (dd, *J* = 6.4, 4.9 Hz, 2H, CH_2-Imid), 3.74 (dd, *J* = 6.4, 4.9 Hz, 2H, CH_2NH), 2.50 – 2.41 (m, 1H, C*H*(CH_3)$_2$), 1.04 (d, *J* = 6.9 Hz, 6H, (CH_3)$_2$CH). **^{13}C NMR** (101 MHz, MeOD) *δ* 180.8 (*C*=O), 136.7 (N*C*HN), 136.4 (C_{Ph}), 131.5 (2C, 2 x $CH_{Ph\ meta}$), 131.4 ($CH_{Ph\ para}$), 125.2 (CH_{imid}), 123.3 (CH_{imid}), 122.7 (2C, 2 x $CH_{Ph\ ortho}$), 51.1 (CH_2-Imid), 39.9 (CH_2NH), 36.2 (1C, *C*H(CH_3)$_2$), 19.9 (2C, 2 x CH_3CH). **FTIR (neat)** ν_{max} = 3320, 2980, 2930, 2110, 1660, 1224 cm^{-1}. **HR – MS** (ESI, positive ion mode) – *m/z* for $[C_{15}H_{20}N_3O]^+$ 258.1601, found 258.1590. **MP** 105 - 110 °C.

3.25 Compound 1, [(^{i}PrC(O)NH(CH_2)$_2$-NHC-Ph)Rh(Cl)(COD)]

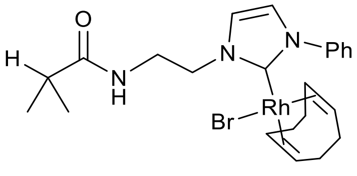

Following general procedure 5 with precursor **S1** (0.02 g, 0.06 mmol) in acetone (1.5 mL), potassium carbonate (0.016 g, 0.12 mmol) and [Rh(COD)$_2$Cl$_2$] (0.015mg, 0.03 mmol, 0.5 equiv), chromatography on silica (eluent dichloromethane:methanol 95:5) afforded two compounds both as yellow oils (bromide salt: 13 mg, 0.023 mmol, 40 %; chloride salt: 6 mg, 0.012 mmol, 20 %). Those were identified as the bromide salt and the chloride salt. Anion exchange was performed on both solids by stirring each of them with NaBr (1 equiv) in

dry methanol for 6 hours; the reaction was monitored by thin layer chromatography, which showed the chloride salt evolved into the bromide salt whereas the bromide salt did not evolve).

Bromide salt:

^{1}H NMR (500 MHz, $CDCl_3$) δ 8.22 – 8.12 (dd, *J* = 8.0 Hz, 1.2 Hz, 2H, 2 x $CH_{Ph\ ortho}$), 7.55 (dd, *J* = 8.4, 7.1 Hz, 2H, 2 x $CH_{Ph\ meta}$), 7.50 – 7.43 (dt, *J* = 7.5, 1.8 Hz, 1H, $CH_{Ph\ para}$), 7.17 (d, *J* = 2.0 Hz, 1H, CH_{Imid}), 7.12 – 7.06 (m, 1H, N*H*), 7.05 (d, *J* = 2.0 Hz, 1H, CH_{Imid}), 5.47 (ddd, *J* = 13.6, 10.2, 5.0 Hz, 1H, 0.5 x CH_2Imid), 5.15 (td, *J* = 7.9, 4.1 Hz, 1H, C*H*=CH_{COD}), 5.04 – 4.95 (m, 1H, C*H*=CH_{COD}), 4.30 (dt, *J* = 13.8, 4.6 Hz, 1H, CH_2Imid), 4.17 (dddd, *J* = 15.1, 10.3, 7.2, 4.9 Hz, 1H, CH_2NH_{AB}), 3.82 – 3.72 (m, 1H, CH_2NH_{AB}), 3.32 – 3.26 (m, 1H, C*H*=CH_{COD}), 2.69 – 2.63 (m, 1H, C*H*=CH_{COD}), 2.46 (hept, *J* = 6.9 Hz, 1H, C*H*(CH_3)$_2$), 2.41 – 2.31 (m, 1H, C_aH_{2COD}), 2.31 – 2.21 (m, 1H, C_cH_{2COD}), 2.06 (ddt, *J* = 14.5, 8.7, 7.6 Hz, 1H, C_dH_{2COD}), 1.88 – 1.75 (m, 3H, C_aH_{2COD}, C_cH_{2COD}, C_dH_{2COD}), 1.46 (dt, *J* = 9.2, 6.2 Hz, 2H, C_bH_{2COD}), 1.08 (d, *J* = 6.9 Hz, 3H, CH_3CH), 0.95 (d, *J* = 6.9 Hz, 3H, CH_3CH). **^{13}C NMR** (126 MHz, $CDCl_3$) δ 183.3 (d, *J* = 50.4 Hz, C_{imid}Rh) 178.6 (*C*=O), 140.3 (C_{Ph}), 129.1 (2C, 2 x $CH_{Ph\ meta}$), 128.2 ($CH_{Ph\ para}$), 124.8 (2C, 2 x $CH_{Ph\ ortho}$), 122.0 (CH_{imid}), 121.2 (CH_{imid}), 97.9 (d, *J* = 6.9 Hz, *C*H=CH_{COD}), 97.6 (d, *J* = 7.2 Hz, *C*H=CH_{COD}), 70.0 (d, *J* = 14.5 Hz, *C*H=CH_{COD}), 69.67 (d, *J* = 14.7 Hz, *C*H=CH_{COD}), 49.2 (1C, CH_2Imid), 38.2 (1C, CH_2NH), 35.2 (*C*H(CH_3)$_2$), 33.6 (CH_{2COD}), 31.4 (CH_{2COD}), 29.4 (CH_{2COD}), 28.6 (CH_{2COD}), 19.7 (CH_3CH), 19.5 (CH_3CH). **FTIR (neat)** ν_{max} = 3280, 2925, 1650, 1528, 1498, 1476, 1237, 910, 726, 693 cm^{-1}. **HR – MS** (ESI, positive ion mode) – *m/z* for $[C_{23}H_{31}N_3ORh]^+$ 468.1517, found 468.1505.

Chloride salt:

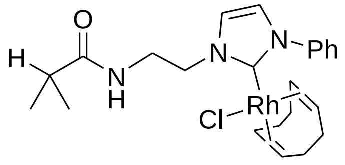

^{1}H NMR (500 MHz, $CDCl_3$) δ 8.14 (dd, *J* = 8.4, 1.2 Hz, 2H, 2 x $CH_{Ph\ ortho}$), 7.56 (dd, *J* = 8.4, 7.1 Hz, H, 2 x $CH_{Ph\ meta}$), 7.48 (dt, *J* = 7.4, 1.7 Hz, 1H, $CH_{Ph\ para}$), 7.43 - 7.37 (bs, 1H, N*H*), 7.16 (d, *J* = 2.0 Hz, 1H, CH_{Imid}), 7.03 (d, *J* = 2.0 Hz, 1H, CH_{Imid}), 5.52 – 5.40 (ddd, *J* = 13.8, 10.3, 4.7 Hz, 1H, CH_2Imid), 5.10 – 5.05 (m, 1H, C*H*=CH_{COD}), 4.93 – 4.88 (m, 1H, C*H*=CH_{COD}), 4.32 (dt, *J* = 13.8, 4.7 Hz, 1H, CH_2Imid), 4.17 – 4.08 (dddd, J = 14.8H, 10.3, 7.0, 4.7 Hz, CH_2NH_{AB}), 3.80 (dtd, *J* = 14.8, 4.7, 3.2 Hz, 1H, CH_2NH_{AB}), 3.24 (ddd, *J* = 7.2, 4.4, 2.8 Hz, 1H, C*H*=CH_{COD}) 2.64 – 2.55 (m, 1H, C*H*=CH_{COD}), 2.46 (hept, *J* = 6.9 Hz, 1H, C*H*(CH_3)$_2$), 2.38 (ddt, *J* = 13.5, 9.5, 6.8 Hz, 1H, C_aH_{2COD}), 2.32 – 2.23 (m, 1H, C_cH_{2COD}), 2.08 (dq, *J* = 14.2, 8.1 Hz, 1H, C_dH_{2COD}), 1.93 – 1.83 (m, 1H, C_aH_{2COD}), 1.84 – 1.71 (m, 2H, C_cH_{2COD}, C_dH_{2COD}), 1.50 (dt, *J* = 8.4, 6.1 Hz, 2H, C_bH_{2COD}), 1.06 (d, *J* = 6.9 Hz, 3H, CH_3CH), 0.98 – 0.90 (d, *J* = 6.9 Hz, 3H, CH_3CH). **^{13}C NMR** (126 MHz, $CDCl_3$) δ 183.3 (d, *J* = 50.9 Hz, C_{imid}Rh) 178.7 (*C*=O), 140.4 (C_{Ph}), 129.1 (2C, 2 x $CH_{Ph\ meta}$), 128.3 ($CH_{Ph\ para}$), 124.9 (2C, 2 x $CH_{Ph\ ortho}$), 122.0 (CH_{imid}), 121.3 (CH_{imid}), 98.3 (d, *J* = 7.0 Hz, *C*H=CH_{COD}), 98.0 (d, *J* = 7.0 Hz, *C*H=CH_{COD}), 68.9 (d, *J* = 14.5 Hz, *C*H=CH_{COD}), 68.6 (d, *J* = 14.9 Hz, *C*H=CH_{COD}), 49.4 (1C, CH_2Imid), 38.5(1C, CH_2NH), 35.1 (*C*H(CH_3)$_2$), 33.7 (CH_{2COD}), 31.7 (CH_{2COD}), 29.1 (CH_{2COD}), 28.5 (CH_{2COD}), 19.7 (CH_3CH), 19.5 (CH_3CH). **FTIR (neat)** ν_{max} = 3280, 2925, 1650, 1528, 1498, 1476, 1237, 910, 726, 693 cm^{-1}. **HR – MS** (ESI, positive ion mode) – *m/z* for $[C_{23}H_{31}N_3OClRhNa]^+$ 526.1103, found 526.1086, *m/z* for $[C_{23}H_{31}N_3ORh]^+$ 468.1522, found 468.1504.

3.26 N3Aib4(CH2)2(Im)Ph+Br−, Precursor S2

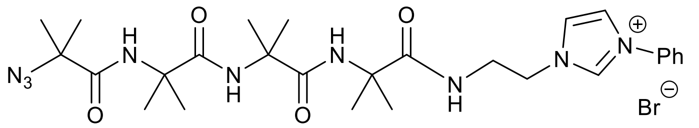

Synthesised following general procedure 4 using 1-(2-aminoethyl)-3-(phenyl)-1H-imidazol-3-ium bromide, hydrobromide salt **5** (0.384 g, 1.1 mmol) and N_3Aib_4OH, which afforded the target compound as foam (0.270 g, 0.49 mmol, 49 %). **^{1}H NMR** (400 MHz, $CDCl_3$) δ 9.96 – 9,89 (m, 1H, C*H*$_{imid}$), 8.37 (s, 1H, N*H*), 8.05 – 7.98 (m, 1H, C*H*$_{imid}$), 7.86 (t, *J* = 6.1 Hz, 1H, N*H*CH_2), 7.75 – 7.68 (m, 2H, 2 x C*H*$_{Ph}$), 7.66 (s, 1H, C*H*$_{imid}$), 7.61 – 7.48 (m, 4H, N*H*, 3 x C*H*$_{Ph}$), 7.08 (s, 1H, N*H*), 4.68 (dd, *J* = 6.2, 3.3 Hz, 2H, C*H*$_2$Imid), 3.85 – 3.69 (m, 2H, C*H*$_2$NH), 1.58 (s, 6H, 2 x C*H*$_{3Aib}$), 1.51 (s, 6H, 2 x C*H*$_{3Aib}$), 1.44 (s, 6H, 2 x C*H*$_{3Aib}$), 1.35 (s, 6H, 2 x C*H*$_{3Aib}$). **^{13}C NMR** (101 MHz, $CDCl_3$) δ 176.4 (*C*=O), 175.9 (*C*=O), 175.0 (*C*=O), 173.5 (*C*=O), 135.8 (*C*H$_{Imid}$), 134.8 (*C*), 130.4 (2C, 2 x *C*H$_{Ph}$), 130.3 (*C*H$_{Ph}$), 124.7 (*C*H$_{Im}$), 122.3 (2C, 2 x *C*H$_{Ph}$), 120.6 (*C*H$_{Im}$), 63.8 (*C*Me$_2$), 57.1 (*C*Me$_2$), 56.9 (*C*Me$_2$), 56.9 (*C*Me$_2$), 50.5 (*C*H$_2$), 39.8 (*C*H$_2$), 25.3 (2C, (*C*H$_3$)$_2$C), 24.9 (2C, (*C*H$_3$)$_2$C), 24.7 (2C, (*C*H$_3$)$_2$C), 24.2 (2C, (*C*H$_3$)$_2$C). **FTIR (neat)** ν_{max} = 3323, 2987, 2936, 2114, 1659, 1538, 1224 cm^{-1}. **HR – MS** (ESI, positive ion mode) – *m/z* for $[C_{27}H_{40}N_9O_4]^+$ 554.3198, found 554.3184.

3.27 Foldamer 2, [N3Aib4NH(CH2)2-NHC-Ph)Rh(Cl)(COD)]

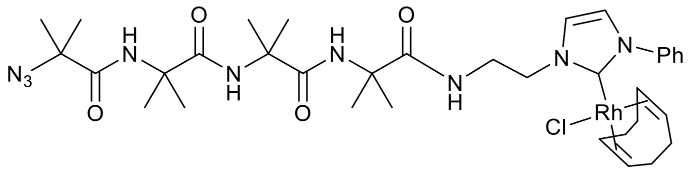

Following general procedure 5, with $[N_3Aib_4NH(CH_2)_2(Im\text{-}Ph)]^+Br^-$ (precursor **S2**) (0.094 g, 0.148 mmol), dry acetone (4.5 mL), potassium carbonate K_2CO_3 (0.022 g, 0.22 mmol), bis(1,5-cyclooctadiene)dirhodium(I) dichloride ($[Rh(COD)Cl]_2$, 0.037 g, 0.074 mmol), afforded the product as a yellow solid (0.073 g, 0.096 mmol, 65 %). **^{1}H NMR** (400 MHz, $CDCl_3$) δ 8.15 (d, *J* = 7.7 Hz, 2H, 2 x C*H*$_{Ph\ ortho}$), 7.67 (dd, *J* = 6.8, 4.5 Hz, 1H, N*H*CH_2), 7.51 (m, 2 Hz, 2H, 2 x C*H*$_{Ph\ meta}$), 7.46 (d, *J* = 2.0 Hz, 1H, C*H*$_{Im}$), 7.41 (t, *J* = 7.4 Hz, 1H, C*H*$_{Ph\ para}$), 7.15 (s, 1H, N*H*), 7.06 (d, *J* = 2.0 Hz, 1H, C*H*$_{Im}$), 7.03 (s, 1H, N*H*), 6.50 (s, 1H, N*H*), 5.11 – 5.05 (m, 1H, C*H*$_2$N$_{imid}$), 5.05 – 4.97 (m, 1H, C*H*=CH$_{cod}$), 4.91 – 4.83 (m, 1H, C*H*=CH$_{cod}$), 4.79 – 4.67 (m, 1H, C*H*$_2$N$_{imid}$), 4.02 (m, 1H, C*H*$_2$NH), 3.70 (m, 1H, C*H*$_2$NH), 3.31 (m, 1H, C*H*=CH$_{COD}$), 2.52 (m, 1H, C*H*=CH$_{COD}$), 2.42 – 2.30 (m, 1H, C*H*$_2$CH$_{cod\ a}$), 2.29 – 2.19 (m, 1H, C*H*$_2$CH$_{cod\ b}$), 2.13 – 2.03 (m, 1H, C*H*$_{2\ COD\ c}$), 1.87 – 1.80 (m, 1H, C*H*$_{2\ COD\ a}$), 1.80 – 1.68 (m, 2H, C*H*$_{2\ COD\ b}$, C*H*$_{2\ COD\ c}$), 1.55 – 1.42 (m, 2H, C*H*$_{2\ COD\ d}$), 1.53 (s, 3H, $(CH_3)_2C$), 1.51 (s, 3H, $(CH_3)_2C$), 1.50 – 1.48 (m, 6H, 2 x $(CH_3)_2C$), 1.43 (s, 3H, $(CH_3)_2C$), 1.42 (s, 3H, $(CH_3)_2C$), 1.41 – 1.39 (m, 6H, 2 x $(CH_3)_2C$). **^{13}C NMR** (101 MHz, $CDCl_3$) δ 182.2 (d, $J_{Rh\text{-}C}$ = 51.0 Hz, N*C*N$_{imid}$) , 175.8 (*C*=O), 173.7 (*C*=O), 173.4 (*C*=O), 173.0 (*C*=O), 140.5 (*C*$_{Ph}$), 128.9 (2C, 2 x *C*H$_{Ph\ meta}$), 127.8 (*C*H$_{Ph\ para}$), 124.6 (2C, 2 x *C*H$_{Ph\ ortho}$), 123.3 (*C*H$_{imid}$), 121.0 (*C*H$_{imid}$), 97.6 (d, $J_{Rh\text{-}C}$ = 7.0 Hz, *C*H$_{COD}$), 97.4 (d, $J_{Rh\text{-}C}$ = 7.0 Hz, *C*H$_{COD}$), 69.08 (d, $J_{Rh\text{-}C}$ = 14.5 Hz, *C*H$_{COD}$), 68.56 (d, $J_{Rh\text{-}C}$ = 14.5 Hz, *C*H$_{COD}$), 64.0 (*C*$(CH_3)_2N_3$), 57.4 (*C*$(CH_3)_2$), 57.0 (*C*$(CH_3)_2$), 57.0 (*C*$(CH_3)_2$), 50.6 (*C*H$_2$N$_{imid}$), 40.7 (*C*H$_2$NH), 33.4 (*C*H$_{2\ COD}$), 31.8 (*C*H$_{2\ COD}$), 29.0 (*C*H$_{2\ COD}$), 28.5 (*C*H$_{2\ COD}$), 26.1 (1C, (*C*H$_3$)$_2$C), 25.8 (1C, (*C*H$_3$)$_2$C), 25.0 (1C, (*C*H$_3$)$_2$C), 25.0 (1C, (*C*H$_3$)$_2$C), 24.8 (1C, (*C*H$_3$)$_2$C), 24.6 (1C, (*C*H$_3$)$_2$C), 24.4 (2C, (*C*H$_3$)$_2$C). **FTIR (neat)** ν_{max} = 3305, 2984, 2936, 2878, 2834, 2111, 1651, 1519, 1383, 1222, 909, 730 cm^{-1}. **HR – MS** (ESI, positive ion mode) – *m/z* for $[C_{35}H_{51}N_9O_4Rh]^+$ 764.3114, found 764.3090. **MP** 143-145 °C.

3.28 Foldamer 8, [$N_3Aib_4NH(CH_2)_2$-NHC-Ph)Rh(Cl)(NBD)]

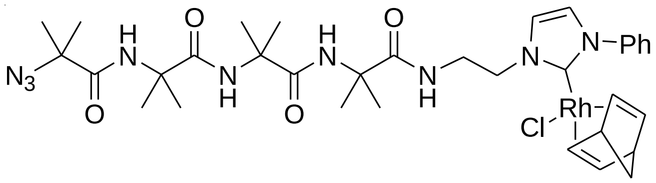

Synthesised following general procedure 5 with [$N_3Aib_4(CH_2)_2$(Im-Ph)]$^+$Br$^-$ (precursor **S2**) (0.04 g, 0.066 mmol) in dry acetone (2 mL), bicyclo[2.2.1]hepta-2,5-diene-rhodium(I) chloride dimer [Rh(NBD)Cl]$_2$ (0.015 g, 0.033 mmol) and K_2CO_3 (0.018 g, 0.13 mmol). The residue obtained was purified by column chromatography (SiO_2, EtOAc/DCM 3:1 then DCM/MeOH 9:1), followed by preparative TLC (DCM/Acetone/MeOH 75:20:5) to yield [$N_3Aib_4NH(CH_2)_2$(Im)PhRh(Cl)(NBD)] as a yellow solid (0.012 g, 0.014 mmol, 20 %). **^{1}H NMR** (400 MHz, $CDCl_3$) δ 8.19 – 8.09 (d, *J* = 7.3 Hz, 2H, 2 x C$H_{Ph\ ortho}$), 7.60 – 7.53 (m, 3H, 2 x C$H_{Ph\ meta}$, N*H*CH$_2$), 7.47 (t, *J* = 7.4 Hz, 1H, C$H_{Ph\ para}$), 7.37 (d, *J* = 2.0 Hz, 1H, CH_{imid}), 7.10 (s, 1H, N*H*), 7.03 (d, *J* = 2.0 Hz, 1H, CH_{imid}), 6.88 (s, 1H, N*H*), 6.21 (s, 1H, N*H*), 5.14 – 5.02 (m, 1H, CH_2-Imid), 4.83 – 4.73 (m, 2H, 2 x C*H*=CH $_{nbd}$), 4.66 – 4.53 (m, 1H, CH_2-Imid), 4.23 – 4.11 (m, 1H, CH_2NH), 3.76 – 3.71 (m, 1H, CH_2NH), 3.71 – 3.63 (m, 1H, C*H*CH$_{2\ nbd}$), 3.59 – 3.49 (m, 1H, C*H*=CH$_{nbd}$), 3.33 – 3.24 (m, 1H, C*H*CH$_{2\ nbd}$), 2.60 – 2.48 (m, 1H, C*H*=CH$_{nbd}$), 1.57 – 1.50 (m, 12H, 2 x (CH_3)$_2$C$_{Aib}$), 1.49 – 1.46 (s, 6H, (CH_3)$_2$C$_{Aib}$), 1.45 – 1.43 (s, 6H, (CH_3)$_2$C$_{Aib}$), 1.17 (dt, *J* = 8.4, 1.7 Hz, 1H, C$H_{2\ NBD}$), 1.12 (dt, *J* = 8.4, 1.7 Hz, 1H, C$H_{2\ NBD}$). **^{13}C NMR** (126 MHz, $CDCl_3$) δ 184.8 (d, *J* = 57.4 Hz, *C*=Rh), 175.8 (*C*=ONHCH$_2$), 173.5 (*C*=O$_{Aib}$), 173.3 (*C*=O$_{Aib}$), 173.2 (*C*=O$_{Aib}$), 140.8 (*C*), 129.0 (2C, 2 x *C*H$_{Ph\ meta}$), 128.0 (*C*H$_{Ph\ para}$), 124.4 (2C, 2 x *C*H$_{Ph\ ortho}$), 123.8 (*C*H$_{Imid}$), 120.9 (*C*H$_{Imid}$), 76.3 (m, 1C, *C*H=CH$_{NBD}$), 75.8 (m, 1C, *C*H=CH$_{NBD}$), 64.2 (N$_3$*C*$_{Aib}$), 63.3 (d, *J* = 4.9 Hz, 1C, *C*H$_{2NBD}$), 57.7 (*C*$_{Aib}$), 57.2 (*C*$_{Aib}$), 57.1 (*C*$_{Aib}$), 51.0 (*C*HCH$_{2NBD}$), 50.9 (*C*HCH$_{2NBD}$), 50.8 (*C*H$_2$-Imid), 50.1 (m, 1C, *C*H=CH$_{NBD)}$, 48.8 (m, 1C, *C*H=CH$_{NBD}$), 40.9 (*C*H$_2$NH), 26.4 (*C*H$_3$C$_{Aib}$), 26.1 (*C*H$_3$C$_{Aib}$), 25.3 (*C*H$_3$C$_{Aib}$), 25.2 (*C*H$_3$C$_{Aib}$), 25.0 (*C*H$_3$C$_{Aib}$), 24.9 (*C*H$_3$C$_{Aib}$), 24.6 (2C, 2 x *C*H$_3$C$_{Aib}$). **FTIR (neat)** ν_{max} = 3323, 2928, 2110, 1670, 1526, 1501, 1456, 1390, 1275, 1230, 1172 cm^{-1} **HR – MS** (ESI, positive ion mode) – *m/z* for [$C_{34}H_{47}N_9O_4Rh^+$] 748.2801, found 748.2779. **MP** 160 – 164 °C.

3.29 [$N_3Aib_4(CH_2)_2$(Im-Mes)]$^+$Br$^-$, Precursor S3

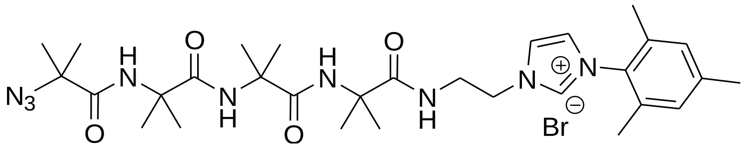

Synthesised following general procedure 4 using 1-(2-aminoethyl)-3-(2,4,6-trimethylphenyl)-1H-imidazol-3-ium bromide, hydrobromide salt **6** (0.43 g, 1.1 mmol) and N_3Aib_4OH, which afforded the compound as a white solid (0.262 g, 0.44 mmol, 44 %). **^{1}H NMR** (400 MHz, $CDCl_3$) δ 9.54 (dd, *J* = 3.6, 1.7 Hz, 1H, NC*H*N, 8.16 – 8.13 (m, 2H, CH_{imid}, N*H*), 7.80 (t, *J* = 6.1 Hz, 1H, N*H*CH$_2$), 7.56 (s, 1H, N*H*), 7.07 (dd, *J* = 3.6, 1.7 Hz, 1H, CH_{im}), 7.02 (s, 1H, N*H*), 6.95 (s, 2H, 2 x CH_{Mes}), 4.73 (t, *J* = 6.0 Hz, 2H, CH_2-Imid), 3.68 (t, *J* = 6.0 Hz, 2H, CH_2NH), 2.30 (s, 3H, CH_{3Mes}), 2.05 (s, 6H, 2 x CH_{3Mes}), 1.54 (s, 6H, 2 x CH_3), 1.47 (s, 6H, 2 x CH_3), 1.40 (s, 6H, 2 x CH_3), 1.26 (s, 6H, 2 x CH_3). **^{13}C NMR** (101 MHz, $CDCl_3$) δ 176.3 (*C*=O), 175.8 (*C*=O), 175.0 (*C*=O), 173.5 (*C*=O), 141.1 (*C*H$_{Imid}$), 137.5 (*C*$_{Mes}$), 134.5 (2C, 2 x *C*H$_{Mes}$), 131.0 (*C*$_{Mes}$), 129.7 (2C, 2 x *C*$_{Mes}$), 125.0 (*C*H$_{Imid}$), 122.6 (*C*H$_{Imid}$), 63.8 (*C*Me$_2$), 57.1 (*C*Me$_2$), 56.9 (2C, 2 x *C*Me$_2$), 50.8 (*C*H$_2$), 40.5 (*C*H$_2$), 25.3 (2C, (*C*H$_3$)$_2$C), 24.8 (2C, (*C*H$_3$)$_2$C), 24.7 (2C, (*C*H$_3$)$_2$C), 24.3 (2C, (*C*H$_3$)$_2$C), 21.1 (*C*H$_{3Mes}$), 17.6 (2C, 2 x *C*H$_{3Mes}$). **FTIR (neat)** ν_{max} = 3318, 2982, 2113, 1654, 1521, 1363, 1211, 1027 cm^{-1} **HR – MS** (ESI, positive ion mode) – *m/z* for [$C_{30}H_{46}N_9O_4$]$^+$ 596.3667, found 596.3681. **MP** 120 °C.

3.30 Foldamer 3, [(N$_3$Aib$_4$NH(CH$_2$)$_2$-NHC-Mes)Rh(Cl)(COD)]

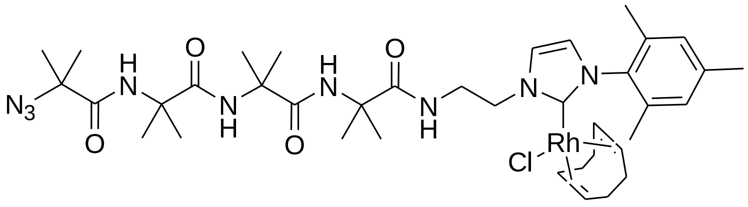

Following general procedure 5 with [N$_3$Aib$_4$NH(CH$_2$)$_2$(Im-Mes)]$^+$Br$^-$ (precursor **S3**) (0.12 g, 0.18 mmol), dry acetone (5 mL), potassium carbonate K$_2$CO$_3$ (0.036 g, 0.36 mmol), bis(1,5-cyclooctadiene)dirhodium(I) dichloride (0.043 g, 0.09 mmol) afforded the product as a yellow solid (0.105 g, 0.126 mmol, 70 %). **^{1}H NMR** (400 MHz, CDCl$_3$) δ 7.65 (dd, *J* = 7.1, 4.6 Hz, 1H, N*H*CH$_2$), 7.40 (d, *J* = 1.9 Hz, 1H, C*H*$_{Imid}$), 7.08 (s, 1H, N*H*$_{Aib}$), 7.03 (s, 1H, C*H*$_{Mes}$), 6.98 (s, 1H, N*H*$_{Aib}$), 6.87 (s, 1H, C*H*$_{Mes}$), 6.63 (d, *J* = 1.9 Hz, 1H, C*H*$_{Imid}$), 6.40 (s, 1H, N*H*$_{Aib}$), 5.42 (dt, *J* = 14.3, 4.4 Hz, 1H, C*H*$_2$Imid), 4.90 (dt, *J* = 7.5 Hz, 1H, CH=CHcod), 4.79 (td, *J* = 7.5, 3.7 Hz, 1H, CH=CHcod), 4.42 (ddd, *J* = 13.8, 9.5, 4.1 Hz, 1H, C*H*$_2$Imid), 4.09 (dddd, *J* = 13.7, 9.4, 7.2, 4.1 Hz, 1H, CH$_2$NH), 3.81 – 3.73 (m, 1H, CH=CHcod), 3.73 – 3.60 (m, 1H, CH$_2$NH), 3.16 – 3.06 (m, 1H, CH=CHcod), 2.39 (s, 3H, C*H*$_{3Mes}$), 2.41 – 2.37 (m, 1H, C*H*$_{2COD\ a}$), 2.34 (s, 3H, C*H*$_{3Mes}$), 2.20 – 2.09 (m, 1H, C*H*$_{2COD\ b}$), 2.03 – 1.93 (m, 1H, C*H*$_{2COD\ c}$), 1.86 – 1.78 (m, 1H, C*H*$_{2COD\ a}$), 1.80 (s, 3H, C*H*$_{3Mes}$), 1.79 – 1.73 (m, 1H, C*H*$_{2COD\ c}$), 1.71 – 1.63 (m, 1H, C*H*$_{2COD\ b}$), 1.52-1.39 (m, 2H, m, 1H, C*H*$_{2COD\ d}$), 1.52 (s, 3H, (C*H*$_3$)$_2$C$_{Aib}$), 1.51 (bs, 6H, (C*H*$_3$)$_2$C$_{Aib}$), 1.49 (s, 3H, (C*H*$_3$)$_2$C$_{Aib}$), 1.46 (bs, 6H, s, 3H, (C*H*$_3$)$_2$C$_{Aib}$), 1.41 (s, 3H, (C*H*$_3$)$_2$C$_{Aib}$), 1.41 (s, 3H, (C*H*$_3$)$_2$C$_{Aib}$). **^{13}C NMR** (101 MHz, CDCl$_3$) δ 180.9 (d, *J* = 50.7 Hz, *C*-Rh), 175.5 (*C*=O), 173.6 (*C*=O), 173.3 (*C*=O), 173.1 (*C*=O), 138.4(*C*$_{Mes}$), 136.9 (*C*$_{Mes}$(CH$_3$)), 136.4 (*C*$_{Mes}$(CH$_3$)), 134.9 (*C*$_{Mes}$), 129.4 (*C*H$_{Mes}$), 128.2 (*C*H$_{Mes}$), 123.4 (*C*H$_{imid}$), 122.8 (*C*H$_{imid}$), 96.2 (d, *J*$_{Rh-C}$ = 7.2 Hz, *C*H=CH$_{COD}$), 96.0 (d, *J*$_{Rh-C}$ = 6.6 Hz, *C*H=CH$_{COD}$), 69.8 (d, *J*$_{Rh-C}$ = 14.3 Hz, *C*H=CH$_{COD}$), 69.1 (d, *J*$_{Rh-C}$ = 14.5 Hz, *C*H=CH$_{COD}$), 64.0 (*C*(CH$_3$)$_2$N$_3$), 57.5 (*C*(CH$_3$)$_2$), 57.0 (*C*(CH$_3$)$_2$), 57.0 (*C*(CH$_3$)$_2$), 51.4 (*C*H$_2$N$_{imid}$), 40.8 (*C*H$_2$NH), 33.9 (*C*H$_{2COD\ a}$), 31.3 (*C*H$_{2\ COD\ d}$), 29.5 (*C*H$_{2\ COD\ c}$), 28.3 (*C*H$_{2\ COD\ b}$), 25.8 (1C, (*C*H$_3$)$_2$C), 25.7 (1C, (*C*H$_3$)$_2$C), 25.5 (1C, (*C*H$_3$)$_2$C), 25.3 (1C, (*C*H$_3$)$_2$C), 25.0 (1C, (*C*H$_3$)$_2$C), 24.8 (1C, (*C*H$_3$)$_2$C), 24.4 (1C, (*C*H$_3$)$_2$C), 24.4 (1C, (*C*H$_3$)$_2$C), 21.1 (*C*H$_3$Ph), 20.5 (*C*H$_3$Ph), 17.9 (*C*H$_3$Ph). **FTIR (neat)** ν_{max} = 3328, 2929, 2114, 1662, 1522, 1261, 1019, 802 cm^{-1}. **HR – MS** (ESI, positive ion mode) – *m/z* for [C$_{38}$H$_{57}$N$_9$O$_4$Rh]$^+$ 806.3583, found 806.3572. **MP** 130-135 °C

3.31 [N$_3$Aib$_4$(CH$_2$)$_2$(Im-Me)]$^+$Br$^-$, Precursor S4

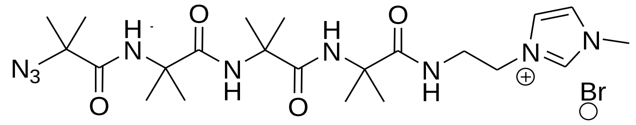

Synthesised following general procedure 4 using 1-(2-aminoethyl)-3-(methyl)-1H-imidazol-3-ium bromide, hydrobromide salt **7** (0.244 g, 0.85 mmol) and N$_3$Aib$_4$OH, which afforded the compound as a white solid (0.163 g, 0.29 mmol, 57 %). **^{1}H NMR** (400 MHz, CDCl$_3$) δ 9.25 (s, 1H, NC*H*N$_{Imid}$), 8.33 (s, 1H, N*H*), 7.67 (d, *J* = 1.9 Hz, 1H, C*H*$_{Imid}$), 7.64 (t, *J* = 6.1 Hz, 1H, N*H*CH$_2$), 7.57 (s, 1H, C*H*$_{Imid}$), 7.33 (s, 1H, N*H*), 6.97 (s, 1H, N*H*), 4.58 – 4.42 (m, 2H, C*H*$_2$Imid), 4.03 (s, 3H, C*H*$_3$Imid), 3.68 – 3.59 (m, 2H, C*H*$_2$NH), 1.60 (s, 6H, (C*H*$_3$)$_2$C), 1.54 (s, 6H, (C*H*$_3$)$_2$C), 1.47 (s, 6H, (C*H*$_3$)$_2$C), 1.45 (s, 6H, (C*H*$_3$)$_2$C). **^{1}H NMR** (400 MHz, MeOD) δ 8.84 (s, 1H, NC*H*N$_{Imid}$), 7.79 (t, 1H, N*H*CH$_2$), 7.65 (d, *J* = 2.0 Hz, 1H, C*H*$_{Imid}$), 7.54 (d, *J* = 2.0 Hz, 1H, C*H*$_{Imid}$), 4.39 (dd, *J* = 6.4, 4.1 Hz, 2H, C*H*$_2$Imid), 3.95 (s, 3H, Me-Imid), 3.67 – 3.60 (m, 2H, C*H*$_2$NH), 1.52 (s, 6H, (C*H*$_3$)$_2$C), 1.46 (s, 6H, (C*H*$_3$)$_2$C), 1.44 (s, 6H, (C*H*$_3$)$_2$C), 1.38 (s, 6H, (C*H*$_3$)$_2$C). **^{13}C NMR** (101 MHz, MeOD) *δ* 178.3 (C=O$_{Aib}$), 177.0 (C=O$_{Aib}$), 176.4 (C=O$_{Aib}$), 174.8 (C=O$_{Aib}$), 138.7 (NCHNImid), 124.7 (CH$_{Imid}$), 123.8 (CH$_{Imid}$), 64.7 （N$_3$*C*), 58.2 (*C*(CH$_3$)$_2$), 58.2 (*C*(CH$_3$)$_2$), 57.9 (*C*(CH$_3$)$_2$), 40.7 (*C*H$_2$NH), 40.6 (*C*H$_2$Imid), 36.6 (N*C*H$_3$), 25.8 (2C, (*C*H$_3$)$_2$C$_{Aib}$), 25.3 (2C, (*C*H$_3$)$_2$C$_{Aib}$), 24.8 (2C, (*C*H$_3$)$_2$C$_{Aib}$), 24.6 (2C, (*C*H$_3$)$_2$C$_{Aib}$). **FTIR (neat)** ν_{max} = 3313, 2112, 1654, 1455, 1384, 1363, 1168 cm^{-1}. **HR – MS** (ESI, positive ion mode) – *m/z* for [C$_{30}$H$_{46}$N$_9$O$_4$]$^+$ 492.3029, found 492.3041. **M.P.** The white solid changes to brown 196 °C, turns black at 240 °C.

3.32 Foldamer 4, [(N_3Aib$_4$NH(CH_2)$_2$-NHC-Me)Rh(Cl)(COD)]

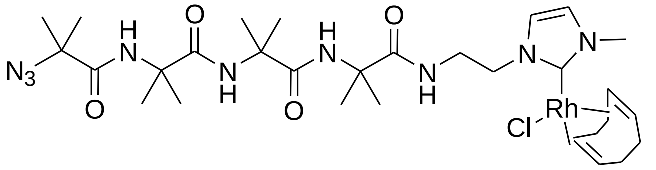

Following general procedure 5 with [N_3Aib$_4$NH(CH_2)$_2$(Im-Me)]$^+$Br$^-$ (precursor **S4**) (0.028 g, 0.05 mmol), dry acetone (10 mL), potassium carbonate K_2CO_3 (0.0138 g, 0.1 mmol), bis(1,5-cyclooctadiene)dirhodium(I) dichloride [Rh(COD)Cl]$_2$ (0.0124 g, 0.025 mmol) afforded the product as a yellow oil (0.011 g, 0.014 mmol, 30 %). **^{1}H NMR** (500 MHz, $CDCl_3$) δ 7.48 (t, *J* = 5.7 Hz, 1H, N*H*CH_2), 7.17 (d, *J* = 1.9 Hz, CH_{Imid}), 7.13 (s, 1H, N*H*), 6.94 (s, 1H, N*H*), 6.74 (d, *J* = 1.9 Hz, 1H, CH_{Imid}), 6.32 (s, 1H, N*H*), 5.09 – 4.98 (m, 2H, 2 x C*H*=CH_{COD}), 4.69 – 4.55 (m, 2H, CH_2imid), 4.00 (s, 3H, CH_3Imid), 3.95 – 3.83 (m, 1H, CH_2NH), 3.83 – 3.71 (m, 1H, CH_2NH), 3.40 – 3.32 (m, 2H, 2 x C*H*=CH_{COD}), 2.44 – 2.26 (m, 4H, 2 x CH_{2COD}), 1.96 – 1.78 (m, 4H, 2 x CH_{2COD}), 1.51 (s, 3H, CH_{3Aib}), 1.51 (s, 3H, CH_{3Aib}), 1.50 (s, 3H, CH_{3Aib}), 1.49 (s, 3H, CH_{3Aib}), 1.48 (s, 3H, CH_{3Aib}), 1.46 (s, 3H, CH_{3Aib}), 1.43 (s, 3H, CH_{3Aib}), 1.41 (s, 3H, CH_{3Aib}). **^{13}C NMR** (126 MHz, $CDCl_3$) δ 181.5 (d, *J* = 50.1 Hz, *C*=Rh), 175.7 (*C*=O_{Aib}), 173.3(*C*=O_{Aib}), 173.2 (*C*=O_{Aib}), 172.9 (*C*=O_{Aib}), 122.6 (*C*H_{imid}), 121.8 (*C*H_{imid}), 97.8 (d, J_{Rh-C} = 6.8 Hz, *C*H=CH_{cod}), 97.7 (d, J_{Rh-C} = 6.8 Hz, *C*H=CH_{cod}), 69.5 (d, J_{Rh-C} = 14.6 Hz, *C*H=CH_{cod}), 68.9 (d, J_{Rh-C} = 14.6 Hz, *C*H=CH_{cod}), 64.2 (*C*$_{Aib}$$N_3$), 57.3 (*C*$_{Aib}$), 57.0 (2C, 2 x C_{Aib}), 49.7 (*C*H_2-Imid), 40.7 (*C*H_2NH), 37.9 (*C*H_3-Imid), 33.2 (*C*$H_{2\ COD}$), 32.5 (*C*$H_{2\ COD}$), 29.8 (*C*$H_{2\ COD}$), 29.0 (*C*$H_{2\ COD}$), 25.8 ($CH_{3\ Aib}$), 25.6 ($CH_{3\ Aib}$), 25.3 ($CH_{3\ Aib}$), 25.2 ($CH_{3\ Aib}$), 25.1 ($CH_{3\ Aib}$), 25.0 ($CH_{3\ Aib}$), 24.5 ($CH_{3\ Aib}$), 24.5 ($CH_{3\ Aib}$). **FTIR (neat)** ν_{max} = 3320, 2929, 2112, 1662, 1525, 1244 cm^{-1}. **HR – MS** (ESI, positive ion mode) – *m/z* for [$C_{30}H_{49}N_9O_4Rh$]$^+$ 702.2963, found 702.2948.

3.33 Precursor 9, [Cbz-(L-αMeVal)Aib$_4$NH(CH_2)$_2$(Im-Ph)]$^+$Br$^-$

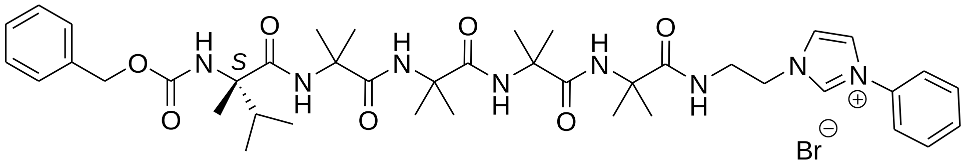

Following general procedure 4 with [Cbz-(L-αMeVal)Aib$_4$OH (0.125 g, 0.25 mmol) in dry dichloromethane (12 mL) was added *N*-(3-dimethylaminopropyl)-*N′*-ethylcarbodiimide EDC.HCl (48 mg, 0.25 mmol). The mixture was stirred at 20 °C for 10 minutes then was cooled to 0°C and triethylamine (0.075 mL, 0.5 mmol) was added. The mixture was stirred for 2 hours at 20 °C, then was concentrated, then dissolved in acetonitrile (15 mL). 1-(2-Aminoethyl)-3-(phenyl)-1H-imidazol-3-ium bromide, hydrobromide salt **5** (148 mg, 0.425 mmol, 1.7 equiv.) was added to the mixture, followed with triethylamine (0.1 mL, 0.7 mmol) and the suspension was heated at 80 °C for 3 days. The mixture was concentrated under reduced pressure, the residue was purified by chromatography on silica (eluent CH_2Cl_2:MeOH 90:10) then by a second chromatography on basic alumina (CH_2Cl_2 then CH_2Cl_2:MeOH) to afford the compound as a oil (112 mg, 0.15 mmol, 61 %). **^{1}H NMR** (400 MHz, $CDCl_3$) δ 9.82 (s, 1H, CH_{imid}), 8.12 (t, *J* = 1.9 Hz, 1H, CH_{imid}), 7.90 (s, 1H, N*H*), 7.79 (s, 1H, N*H*), 7.73 – 7.68 (m, 1H, N*H*CH_2), 7.68 – 7.62 (d, *J* = 6.8 Hz, 2H, 2 x C$H_{Ph\ ortho}$), 7.51 (t, *J* = 1.8 Hz, 1H, CH_{imid}), 7.50 – 7.44 (m, 3H, 3 x C$H_{Ph\ meta\ para}$), 7.41 (d, *J* = 7.4 Hz, 2H, 2 x C$H_{Bn\ ortho}$), 7.26 (dd, *J* = 7.5, 7.4 Hz, 2H, 2 x C$H_{Bn\ meta}$), 7.18 (s, 1H, N*H*), 7.17 (d, *J* = 7.5 Hz, 1H, C$H_{Bn\ para}$), 7.12 (s, 1H, N*H*), 6.68 (s, 1H, N*H*), 5.14 (d, *J* = 13.1 Hz, 1H, CH_2Ph), 4.94 (d, *J* = 13.1 Hz, 1H, CH_2Ph), 4.77 (ddd, *J* = 14.0, 5.6, 2.3 Hz, 1H, CH_2imid), 4.57 (ddd, *J* = 14.0, 9.0, 2.2 Hz, 1H, CH_2imid), 4.05 – 3.92 (m, 1H, CH_2NH), 3.40 – 3.28 (m, 1H, CH_2NH), 2.43 (p, *J* = 6.7 Hz, 1H, C*H*(CH_3)$_2$), 1.43 (s, 3H, C$H_3$$C_{Aib}$), 1.36 (s, 3H, C$H_3$$C_{Aib}$), 1.35 (s, 3H, C$H_3$$C_{Aib}$), 1.32 (s, 3H, C$H_3$$C_{Aib}$), 1.28 (s, 3H, C$H_3$$C_{Aib}$), 1.27 (s, 3H, C$H_3$$C_{aMeVal}$), 1.23 (s, 3H, C$H_3$$C_{Aib}$), 1.18 (s, 3H, C$H_3$$C_{Aib}$), 1.10 (s, 3H, C$H_3$$C_{Aib}$), 0.92 (d, *J* = 6.6 Hz, 3H, (CH_3)$_2$CH), 0.78 (d, *J* = 6.8 Hz, 3H, (CH_3)$_2$CH). **^{13}C NMR** (101 MHz, $CDCl_3$) δ 176.7 (*C*=O_{Aib}), 176.4 (*C*=O_{Aib}), 175.6 (*C*=O_{val}), 175.4 (*C*=O_{Aib}), 175.4 (*C*=O_{Aib}), 156.7 (*C*=O_{Cbz}), 137.4 (*C*$_{Bn}$), 135.6 (N*C*HN_{imid}), 134.9 (*C*$_{Ph}$), 130.3 (2C, 2 x $CH_{Ph\ ortho}$), 130.1 (*C*$H_{Ph\ para}$),

128.6 (2C, 2 x $CH_{Bn\ meta}$), 127.7 ($CH_{Bn\ para}$), 127.0 (2C, 2 x $CH_{Bn\ ortho}$), 125.0 (CH_{imid}), 122.4 (2C, 2 x CH_{Ph}), 120.5 (CH_{imid}), 66.5 (CH_2Ph), 63.0 (C_{iPrCH3}), 57.0 (C_{Aib}), 56.7 (C_{Aib}), 56.6 (C_{Aib}), 56.2 (C_{Aib}), 50.5 (CH_2N_{imid}), 39.9 (CH_2NH), 34.1 ($CH(CH_3)_2$), 27.3 ((CH_3)C_{Aib}), 26.7 (2C, 2 x ((CH_3)C_{Aib}), 26.3 (((CH_3)C_{Aib}), 23.2 (((CH_3)C_{Aib}), 22.9 (2C, 2 x (CH_3)C_{Aib} CH_3), 22.8 (((CH_3)C_{Aib}), 17.3 (1C, (CH_3)$_2$CH), 17.1 (1C, (CH_3)$_2$CH), 15.7 (CH_3C_{aMeVal}). **FTIR (neat)** ν_{max} = 3307, 2991, 2941, 1656, 1531, 1456, 1392, 1261, 1237 cm^{-1}. **HR – MS** (ESI, positive ion mode) – *m/z* for $[C_{41}H_{59}N_8O_7]^+$ 775.4501, found 775.4483.

3.34 Foldamer 14, [(Cbz-(L-αMeVal)Aib₄NH(CH₂)₂-NHC-Ph)Rh(Cl)(COD)]

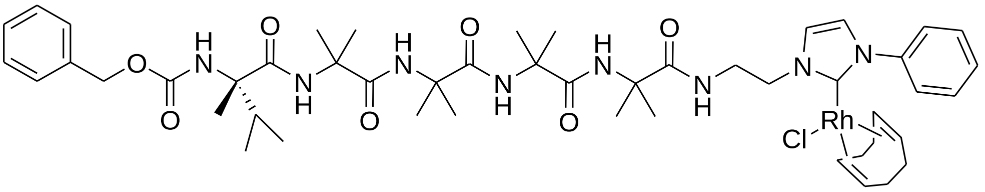

Following general procedure 6 with [Cbz-(L-αMeVal)Aib$_4$NH(CH$_2$)$_2$(Im-Ph)]$^+$Br$^-$ **9** (0.054 g, 0.072 mmol) in dry CH_2Cl_2 (2 mL), silver oxide (0.017 g, 0.072 mmol), bis(1,5-cyclooctadiene)dirhodium(I) dichloride [Rh(COD)Cl]$_2$ (0.018 g, 0.036 mmol), the compound was obtained as a yellow solid (0.047 g, 0.047 mmol, 65 %) as a mixture of diastereomers (ratio 56:44 determined by integration of ^{1}H NMR signals). **^{1}H NMR** (500 MHz, $CDCl_3$) δ 8.21 – 8.16 (m, 2H, 2 x $CH_{Ph\ ortho}$), 7.90 (dd, *J* = 7.6, 3.8 Hz, 0.44H, N*H*$CH_{2\ dia2}$), 7.83 (t, *J* = 5.9 Hz, 0.56H, N*H*$CH_{2\ dia1}$), 7.68 (s, 1H, N*H*), 7.69 – 7.61 (m, 0.56H, $CH_{imid\ dia1}$), 7.55 – 7.48 (m, 3H, 2x $CH_{Ph\ meta}$, N*H*), 7.46 - 7.39 (m, 0.5H, $CH_{imid\ dia2}$), 7.43 - 7.38 (m, 1H, $CH_{Ph\ para}$), 7.38-7.33 (m, 5H, 5 x CH_{Bn}), 7.26 (s, 1H, N*H*), 7.07 (d, *J* = 2.0 Hz, 0.56H, $CH_{Imid\ dia1}$), 7.04 (d, *J* = 2.0 Hz, 0.44H, $CH_{Imid\ dia2}$), 6.33 (s, 1H, N*H*), 5.38 (ddd, *J* = 14.3, 5.9, 3.3 Hz, 0.5H, CH_2Imid$_{dia\ 1}$), 5.29 (s, 0.5H, $NH_{Cbz\ dia2}$), 5.28 (s, 0.5H, $NH_{Cbz\ dia1}$), 5.23 – 5.15 (m, 0.5H, CH_2Imid$_{dia\ 2}$), 5.19 (d, *J* = 12.2 Hz, 0.5H, $CH_{2Ph\ dia\ 1}$), 5.17 (d, *J* = 12.2 Hz, 0.5H, $CH_{2Ph\ dia\ 2}$), 5.04 (d, *J* = 12.2 Hz, 0.5H, $CH_{2Ph\ dia\ 2}$), 5.03 (d, *J* = 12.2 Hz, 0.5 H, $CH_{2Ph\ dia\ 1}$), 5.06 – 5.00 (m, 1H, C*H*=CH_{cod}), 4.96 – 4.87 (m, 1H, C*H*=CH_{cod}), 4.65 (ddd, *J* = 14.3, 8.8, 3.2 Hz, 0.5H, CH_2Imid$_{dia\ 1}$), 4.56 (ddd, *J* = 13.7, 8.3, 5.3 Hz, 0.5H, $CH_{2Imid\ dia\ 2}$), 4.17- 4.08 (dtd, *J* = 17.3, 8.8, 4.7 Hz, 0.5H, $CH_2NH_{\ dia1}$), 4.08- 3.99 (m, 0.5H, $CH_2NH_{\ dia2}$), 3.80 – 3.70 (m, 0.5H, $CH_2NH_{\ dia2}$), 3.61 - 3.53 (m, 0.5H, $CH_2NH_{\ dia1}$), 3.45-3.37 (m, 1H, C*H*=CH_{cod}), 2.58-2.52 (m, 1H, C*H*=CH_{cod}), 2.40 – 2.35 (ddt, *J* = 13.7, 9.3, 6.6 Hz, 1H, $CH_{2\ COD\ a}$), 2.32 - 2.20 (m, 1H, $CH_{2\ COD\ b}$), 2.16 - 2.05 (m, 1H, $CH_{2\ COD\ c}$), 2.03 - 1.81 (m, 1H, C*H*(CH_3)$_2$), 1.88 - 1.77 (m, 1H, $CH_{2\ COD\ a}$), 1.79 – 1.71 (m, 2H, $CH_{2\ COD\ c}$, $CH_{2\ COD\ b}$), 1.63 (s, 1.5H, (CH_3C)$_{Aib\ dia}$), 1.61 (s, 1.5H, (CH_3C)$_{Aib\ dia}$), 1.51 (s, 3H, C(CH_3)$_2$), 1.54 - 1.46 (m, 8H, $CH_{2\ COD\ d}$, $CH_{2\ COD\ d}$, C(CH_3)$_2$, C(CH_3)$_2$), 1.45 - 1.43 (m, 6H, C(CH_3)$_2$, C(CH_3)$_2$), 1.41 (s, 3H, CH_3C_{iPr}), 1.40 -1.25 (m, 3H, C(CH_3)$_2$), 1.21 – 1.17 (m, 3H, C(CH_3)$_2$), 0.98 (d, *J* = 6.8 Hz, 1.5H, $CH_3CH_{\ dia\ 1}$), 0.98 (d, *J* = 6.8 Hz, 1.5H, $CH_3CH_{\ dia\ 2}$), 0.95 (d, *J* = 6.8 Hz, 3H, CH_3CH). **^{13}C NMR** (126 MHz, $CDCl_3$) δ 182.6 (d, *J* = 51.4 Hz, *C*-Rh$_{dia\ 2}$), 181.8 (d, *J* = 50.7 Hz, *C*-Rh *dia1*), 176.2 (*C*=O$_{dia}$), 176.2 (*C*=O$_{dia}$), 175.5 (*C*=O$_{dia}$), 174.9 (*C*=O$_{dia}$), 174.7 (*C*=O$_{dia}$), 174.0 (*C*=O$_{dia}$), 172.9 (*C*=O$_{dia}$), 172.8 (*C*=O$_{dia}$), 156.2 (*C*=$O_{Cbz\ dia1\ dia2}$), 140.8 ($C_{Ph\ dia1}$), 140.8 ($C_{Ph\ dia2}$), 136.0 ($C_{Bn\ dia1}$), 136.0 ($C_{Bn\ dia2}$), 128.9 – 128.7 (m, 12C, 2 x $CH_{Ph\ meta\ dia1}$, 2 x $CH_{Ph\ meta\ dia2}$, 8 x $CH_{Bn\ dias}$), 128.4 (2C, 2 x $CH_{Bn\ dia1}$), 128.3 (2C, 2x $CH_{Bn\ dia2}$), 127.7 ($CH_{Ph\ para\ dia\ 2}$), 127.6 ($CH_{Ph\ para\ dia\ 1}$), 124.9 (2C, 2x $CH_{Ph\ ortho\ dia\ 2}$), 124.8 (2C, 2x $CH_{Ph\ ortho\ dia1}$), 123.8 ($CH_{imid\ dia1}$), 123.6 ($CH_{imid\ dia2}$), 121.0 ($CH_{Imid\ dia1}$), 120.8 ($CH_{Imid\ dia2}$), 97.6 (d, *J* = 7.1 Hz, *C*H=$CH_{cod\ dia}$), 97.4 (d, *J* = 6.8 Hz, *C*H=$CH_{cod\ dia}$), 97.1 (d, *J* = 7.4 Hz, *C*H=$CH_{cod\ dia}$), 97.1 (d, *J* = 7.0 Hz, *C*H=$CH_{cod\ dia}$), 69.3 (d, *J* = 15.6 Hz, *C*H=$CH_{cod\ dia}$), 69.2 (d, *J* = 15.3 Hz, *C*H=$CH_{cod\ dia}$), 68.7 (d, *J* = 14.1 Hz, *C*H=$CH_{cod\ dia}$), 68.6 (d, *J* = 13.2 Hz, *C*H=$CH_{cod\ dia}$), 67.7 (CH_2Ph_{dia}), 67.7 (CH_2Ph_{dia}), 63.2 ($C_{aMeVal}CH_{3\ dia\ 1}$), 63.1 ($C_{aMeVa}lCH_{3\ dia\ 2}$), 57.5 (*C*(CH_3)$_{2\ dia\ 1}$), 57.4 (*C*(CH_3)$_{2\ dia\ 2}$), 57.0 (*C*(CH_3)$_{2\ dia\ 1}$), 57.0 (*C*(CH_3)$_{2\ dia\ 2}$), 56.9 (*C*(CH_3)$_{2\ dia\ 2}$), 56.9 (*C*(CH_3)$_{2\ dia\ 1}$), 56.8 (*C*(CH_3)$_{2\ dia2}$), 56.7 (*C*(CH_3)$_{2\ dia\ 1}$), 50.9 (CH_2Imid$_{dia\ 1}$), 50.8 (CH_2Imid$_{dia\ 2}$), 41.3 ($CH_2NH_{\ dia1}$), 40.7 ($CH_2NH_{\ dia2}$), 35.8 ($CH(CH_3)_{2\ dia\ 1}$), 35.6 ($CH(CH_3)_{2\ dia\ 2}$), 33.5 ($CH_{2\ COD\ a\ dia1}$), 33.3 ($CH2_{\ COD\ a\ dia2}$), 31.9 ($CH_{2\ COD\ d\ dia\ 1}$), 31.9 ($CH_{2\ COD\ d\ dia\ 2}$), 29.1 ($CH_{2\ COD\ c\ dia\ 1}$), 29.0 ($CH_{2\ COD\ c\ dia\ 2}$), 28.6 ($CH_{2\ COD\ b\ dia\ 2}$), 28.6 ($CH_{2\ COD\ b\ dia\ 1}$), 28.1 – 26.2 (8C, 8 x (CH_3)C_{Aib}), 24.0 - 22.7 (8C, 8 x (CH_3)C_{Aib}), 17.7 ((*C*H_3)$C_{aMeVal\ dia2}$), 17.6 ((*C*H_3)$C_{aMeVal\ dia1}$), 17.4 ($CH_3CH_{\ dia2}$), 17.4 ($CH_3CH_{\ dia1}$), 17.3 ($CH_3CH_{\ dia\ 2}$), 17.3 ($CH_3CH_{\ dia\ 1}$). **FTIR (neat)** ν_{max} = 3311, 2991, 2937, 2878,

1662, 1531, 1460, 1392, 1266, 1233 cm^{-1} **HR – MS** (ESI, positive ion mode) – *m/z* for $[C_{49}H_{70}N_8O_7Rh]^+$ 985.4417, found 985.4386. **MP**: 145 °C.

3.35 Foldamer 15 [(Cbz-(L-αMeVal)Aib$_4$NH(CH$_2$)$_2$-NHC-Ph)Rh(Cl)(NBD)

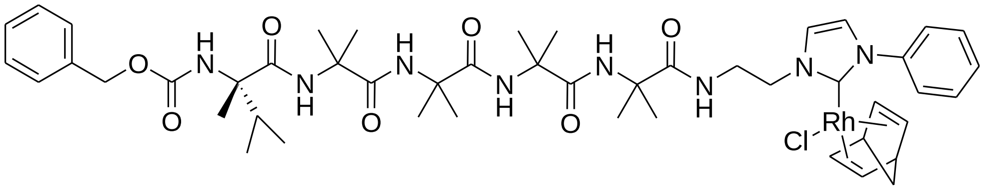

Following general procedure 6 with [Cbz-(L-αMeVal)Aib$_4$NH(CH$_2$)$_2$(Im-Ph)]$^+$Br$^-$ **9** (0.054 g, 0.072 mmol) in dry CH_2Cl_2 (2 mL), silver oxide (0.017 g, 0.072 mmol), bicyclo[2.2.1]hepta-2,5-diene-rhodium(I) chloride dimer $[Rh(NBD)Cl]_2$ (0.018 g, 0.036 mmol), the compound was obtained as a yellow solid (0.045 g, 0.044 mmol, 62 %) as a mixture of diastereomers (ratio 53: 47 based on integration of ^{1}H NMR signals of CH_2Ph and NHCH$_2$; ratio 55: 45 based on fitting of VT-NMR data). **^{1}H NMR** (500 MHz, $CDCl_3$) δ 8.15 (d, *J* = 7.7 Hz, 2H, 2 x C$H_{\text{Ph ortho}}$), 7.82 (s, 1H, NHCH$_2$), 7.68 (s, 1H, NH), 7.56 (t, *J* = 7.8 Hz, 2H, 2 x C$H_{\text{Ph meta}}$), 7.50 (m, 1H, CH_{imid}), 7.48 – 7.42 (m, 1H, C$H_{\text{Ph para}}$), 7.42 – 7.30 (m, 6H, NH, 5 x CH_{Bn}), 7.28 (s, 1H, NH), 7.00 (m, 1H, CH_{imid}), 6.42 (s, 1H, NH), 5.41 (s, 1H, NH_{Cbz}), 5.35 – 5.24 (m 1H, CH_2-Imid), 5.18 (d, *J* = 12.3 Hz, 1H, CH_2Ph), 5.03 (d, *J* = 12.3 Hz, 1H, CH_2Ph), 4.73 – 4.67 (m, 2H, 2 x CH=CH $_{\text{NBD}}$), 4.57 – 4.35 (m, 1H, CH_2-Imid), 4.33 – 4.07 (m, 1H, CH_2NH), 3.90 – 3.47 (m, 3H, CH_2NH, CH=CH$_{\text{NBD}}$, CHCH$_{2\text{NBD}}$), 3.33 – 3.21 (m, 1H, CHCH$_{2\text{NBD}}$), 2.45 (s, 1H, CH=CH$_{\text{NBD}}$), 2.01 – 1.92 (m, 1H, CH(CH$_3$)$_2$), 1.60 (s, 3H, CH_3C$_{\text{Aib}}$), 1.50 (s, 3H, CH_3C$_{\text{Aib}}$), 1.47 (s, 3H, CH_3C$_{\text{Aib}}$), 1.47 (s, 3H, CH_3C$_{\text{Aib}}$), 1.43 (s, 3H, CH_3C$_{\text{Aib}}$), 1.41 (s, 3H, CH_3C$_{\text{Aib}}$), 1.40 (s, 3H, CH_3C$_{\text{MeVal}}$), 1.38 (s, 3H, CH_3C$_{\text{Aib}}$), 1.19 (s, 3H, CH_3C$_{\text{Aib}}$), 1.15 – 1.09 (m, 2H, C$H_{2\text{NBD}}$), 0.97 (d, *J* = 6.9 Hz, 3H, CH_3CH), 0.93 (d, *J* = 6.9 Hz, 3H, CH_3CH). **^{13}C NMR** (126 MHz, $CDCl_3$) δ 183.3 (C=Rh), 176.1 (C=O$_{\text{Aib}}$), 175.6 (C=O$_{\text{Aib}}$), 174.8 (C=O$_{\text{Aib}}$), 174.1 (C=O$_{\text{Aib}}$), 173.1 (C=O$_{\text{MeVal}}$), 156.3 (C=O$_{\text{Cbz}}$), 140.9 (C_{Ph}), 136.2 (C_{Bn}), 128.9 (2C, 2 x CH$_{\text{Ph}}$), 128.8 (2C, 2 x CH$_{\text{Bn}}$), 128.8 (CH$_{\text{Bn}}$), 128.2 (2C, 2 x CH$_{\text{Bn}}$), 127.7 (CH$_{\text{Ph para}}$), 124.5 (2C, 2 x CH$_{\text{Ph orthio}}$), 124.1 (CH $_{\text{imid}}$), 120.4 (CH $_{\text{imid}}$), 76.5 (2C, 2 x CH=CH$_{\text{NBD}}$), 67.6 (CH$_2$Ph), 63.2 (C_{MeVal}), 63.0 (d, *J* = 4.4 Hz, 1C, CH$_{2\text{NBD dia}}$), 57.5 (C_{Aib}), 57.0 (C_{Aib}), 56.8 (C_{Aib}), 56.7 (C_{Aib}), 50.7 (2C, 2 x CHCH$_{2\text{ NBD}}$), 49.3 (2C, 2x CH=CH$_{\text{NBD}}$), 48.2 (m, CH$_2$-Imid), 41.2 (m, CH$_2$NH), 35.7 (CH(CH$_3$)$_2$), 28.4 – 26.4 (4 x CH$_3$C$_{\text{Aib}}$), 24.6 – 22.9 (4 x CH$_3$C$_{\text{Aib}}$), 17.6 (CH$_3$C $_{\text{MeVal}}$), 17.4 (CH$_3$CH$_{\text{MeVal}}$), 17.3 (CH$_3$CH$_{\text{MeVal}}$). **FTIR (neat)** ν_{max} = 3311, 2987, 2941, 1657, 1527, 1499, 1264, 1229 cm^{-1}. **HR – MS** (ESI, positive ion mode) – *m/z* for $[C_{48}H_{66}N_8O_7Rh]^+$ 969.4104, found 969.4073. **MP:** undergoes degradation from 124 °C.

3.36 Precursor 10, [Cbz-(L-αMeVal)Aib$_4$NH(CH$_2$)$_2$(Im-Mes)]$^+$Br$^-$

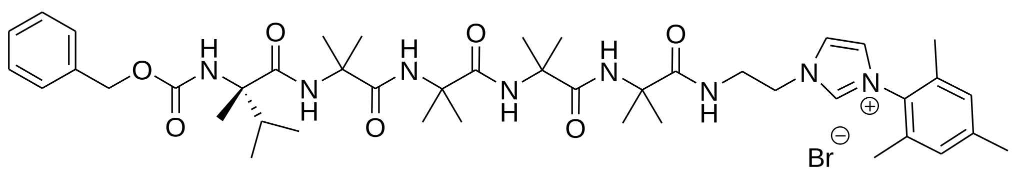

Following general procedure 4, to a solution of Cbz-(L-αMeVal)Aib$_4$OH (0.125 g, 0.25 mmol) in dry dichloromethane (12 mL) was added *N*-(3-dimethylaminopropyl)-*N'*-ethylcarbodiimide EDC.HCl (0.048 g, 0.25 mmol). The mixture was stirred at 20 °C for 10 minutes then was cooled to 0°C and triethylamine (0.075 mL, 0.5 mmol) was added. The mixture was stirred for 2 hours at 20 °C, then was concentrated, then dissolved in acetonitrile (15 mL). 1-(2-Aminoethyl)-3-(mesityl)-1H-imidazol-3-ium bromide, hydrobromide salt (166 mg, 0.425 mmol, 1.7 equiv.) was added to the mixture, followed with triethylamine (0.1 mL, 0.7 mmol) and the suspension was heated at 80 °C for 3 days. The mixture was concentrated under reduced pressure, the residue was purified by chromatography on silica (eluent CH_2Cl_2:MeOH 90:10) then by a second chromatography on basic alumina (CH_2Cl_2 then CH_2Cl_2:MeOH) to afford the compound as a colorless oil (0.112 g, 0.14 mmol, 57 %). **^{1}H NMR** (400 MHz, $CDCl_3$) δ 9.52 (d, *J* = 1.7 Hz, 1H, NCH_{imid}N), 8.18 (t, *J* = 1.8 Hz, 1H,

CH_{imid}), 7.92 (s, 1H, N*H*), 7.78 (s, 1H, N*H*), 7.67 (dd, *J* = 7.7, 4.6 Hz, 1H, N*H*CH_2), 7.44 – 7.32 (m, 2H, 2 x CH_{Ph}), 7.26 (t, *J* = 7.5 Hz, 2H, 2 x CH_{Ph}), 7.19 (d, *J* = 7.3 Hz, 1H, C$H_{Ph\ para}$), 7.13 (m, 2H, 2 x N*H*), 7.00 (d, *J* = 1.8 Hz, 1H, CH_{imid}), 6.92 (s, 1H, CH_{Mes}), 6.90 (s, 1H, CH_{Mes}), 6.76 (s, 1H, N*H*), 5.13 (d, *J* = 13.1 Hz, 1H,CH_2Ph), 4.94 (d, *J* = 13.1 Hz, 1H, CH_2Ph), 4.85 – 4.73 (m, 1H, CH_2N_{imid}), 4.71 – 4.57 (m, 1H, CH_2N_{imid}), 3.97 (m, 1H, CH_2NH), 3.30 (m, 1H, CH_2NH), 2.38 (d, *J* = 6.8 Hz, 1H, C*H*($CH_3)_2$), 2.26 (s, 3H, CH_3Ph), 2.04 (s, 3H, CH_3Ph), 1.99 (s, 3H, CH_3Ph), 1.39 (s, 3H, CH_3), 1.35 (s, 3H, CH_3), 1.31 (s, 3H, CH_3), 1.30 (s, 3H, CH_3), 1.26 (s, 3H, CH_3), 1.25 (s, 3H, CH_3), 1.18 (s, 3H, CH_3), 1.12 (s, 3H, CH_3), 1.10 (s, 3H, CH_3), 0.91 (d, *J* = 6.7 Hz, 3H, (C$H_3)_2$CH), 0.77 (d, *J* = 6.8 Hz, 3H, (C$H_3)_2$CH). **^{13}C NMR** (101 MHz, $CDCl_3$) δ 176.6 (*C*=O_{Aib}), 176.4 (*C*=O_{Aib}), 175.6 (*C*=O_{Aib}), 175.5 (*C*=O_{Aib}), 175.4 (*C*=O_{aMeVal}), 156.8 (*C*=O_{Cbz}), 140.9 (C_{Mes}-CH_{3para}), 137.6 (*C*H_{imid}), 137.4 (C_{Bn}), 134.8 ($C_{MesCH3ortho}$), 134.3 ($C_{MesCH3ortho}$), 131.1 ($C_{Mesityl}$), 129.8 (*C*HMes), 129.5 (*C*HMes), 128.6 (2C, 2 x CH_{Ph}), 127.8 (CH_{Ph}), 127.1 (2C, 2 x CH_{Ph}), 125.1 (CH_{imid}), 122.5 (CH_{imid}), 66.5 (CH_2Ph), 63.0 (C_{aMeVal}), 57.1 (C_{Aib}), 56.6 (C_{Aib}), 56.6 (C_{Aib}), 56.2 (C_{Aib}), 50.6 (CH_2N_{imid}), 40.5 (CH_2NH), 34.2 (*C*H($CH_3)_2$), 27.4 - 26.3 (4 X $CH_{3\ Aib}$), 23.2-22.6 (4 X $CH_{3\ Aib}$), 21.1 ($CH_{3Phpara}$), 17.6 - 17.2 (2 x ($CH_3)_2$CH, 2 x $CH_{3Mesortho}$), 15.8 ($CH_{3aMeVal}$). **FTIR (neat)** ν_{max} = 331, 2924, 2857, 1750, 1666, 1535, 1213, 1039 cm^{-1}. **HR – MS** (ESI, positive ion mode) – *m/z* for $[C_{44}H_{65}N_8O_7]^+$ 817.4971, found 817.4955.

3.37 Foldamer 16, [(Cbz-(L-αMeVal)Aib₄NH(CH₂)₂-NHC-Mes)Rh(Cl)(COD)]

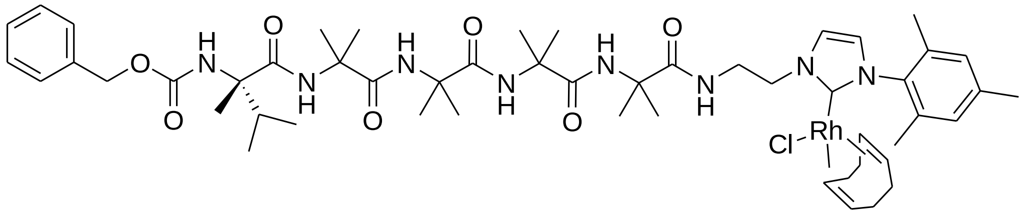

Following general procedure 6 with [Cbz-(L-αMeVal)$Aib_4NH(CH_2)_2$(Im-Mes)]$^+$Br$^-$ **10** (0.065 g, 0.082 mmol), dichloromethane (5 mL), silver oxide (0.019 g, 0.082 mmol), $[Rh(COD)Cl]_2$ (0.021 g, 0.041 mmol), the compound was obtained as a yellow solid (0.043 g, 0.041 mmol, 50%) as a mixture of diastereomers (ratio 45:55 determined by integration of ^{1}H NMR signals of CH_{imid} and CH_{Mes} signals). **^{1}H NMR** (400 MHz, $CDCl_3$) δ 7.86 – 7.79 (m, 1H, N*H*CH_2), 7.66 (s, 0.5H, NH_{dia}), 7.55 (s, 0.5H, NH_{dia}), 7.55 (d, *J* = 1.9 Hz, 0.5 H, C$H_{Imid\ dia1}$), 7.52 – 7.48 (m, 1H, NH), 7.39 – 7.34 (m, 4H, 4 x CH_{Bn}), 7.31 – 7.28 (m, 1H, CH_{Bn}), 7.37 (m, 0.5H, C$H_{Imid\ dia2}$), 7.22 (s, 0.5H, N*H*), 7.21 (s, 0.5H, N*H*), 7.06 (s, 1H, C$H_{Mes\ dias}$), 6.89 (s, 0.5H, C$H_{Mes\ dia}$), 6.88 (s, 0.5H, C$H_{Mes\ dia}$), 6.66 (d, *J* = 1.9 Hz, 0.5H, C$H_{Imid\ dia1}$), 6.61 (d, *J* = 1.8 Hz, 1H, C$H_{Imid\ dia2}$), 6.31 (s, 1H, N*H*), 5.64 – 5.51 (m, 1H, CH_2Imid $_{dia}$), 5.27 (s, 0.5H, N*H*Cbz$_{dia}$), 5.25 (s, 0.5H, N*H*Cbz$_{dia}$), 5.19 (d, *J* = 12.2 Hz, 1H, CH_2Ph$_{dias}$), 5.04 (dd, *J* = 12.2 Hz, 1H, CH_2Ph$_{dias}$), 4.91 – 4.79 (m, 1H, CH=CH$_{COD\ dia}$), 4.77 – 4.71 (m, 1H, CH=CH$_{COD\ dia}$), 4.45 – 4.35 (m, 0.5 H, CH_2Imid $_{dia}$), 4.29 – 4.25 (m, 0.5H, CH_2Imid $_{dia}$), 4.22 – 4.26 (m, 0.5H, CH_2NH), 4.19 – 4.22 (m, 0.5H, CH_2NH), 3.94 – 3.71 (m, 0.5H, CH_2NH), 3.90 – 3.79 (m, 1H, C*H*=CH$_{COD}$), 3.63 – 3.54 (m, 0.5H, CH_2NH), 3.09 – 2.98 (m, 1H, C*H*=CH$_{COD}$), 2.52 – 2.31 (m, 1H, CH_2CH$_{COD}$), 2.44 (s, 1.5H, C$H_{3Mes\ dia}$), 2.43 (s, 1.5H, C$H_{3Mes\ dia}$) 2.36 (s, 3H, C$H_{3Mes\ dia}$), 2.23 – 2.11 (m, 1H, CH_2CH$_{COD}$), 2.09 – 1.91 (m, 1H, CH_2CH$_{COD}$), 2.04 - 1.89 (m, 1H, C*H*($CH_3)_2$), 1.97 – 1.82 (m, 1H, CH_2CH$_{COD}$), 1.89 (s, 1.5H, C$H_{3Mes\ dia}$), 1.85 (s, 1.5H, C$H_{3Mes\ dia}$), 1.84 – 1.66 (m, 1H, CH_2CH$_{COD}$), 1.78 – 1.60 (m, 1H, CH_2CH$_{COD}$), 1.60 – 1.44 (m, 2H, CH_2CH$_{COD}$), 1.57 (s, 3H, CH_3), 1.52 – 1.36 (m, 18H, 6 x CH_3C), 1.25 (s, 3H, CH_3C), 1.19 (s, 1.5H, CH_3C$_{dia}$), 1.17 (s, 1.5H, CH_3C$_{dia}$), 0.98 (d, *J* = 6.7 Hz, 3H, (C$H_3)_2$CH), 0.95 (d, *J* = 6.8 Hz, 3H, (C$H_3)_2$CH). **^{13}C NMR** (126 MHz, $CDCl_3$) δ 181.2 (d, *J* = 50.4 Hz, 0.5C, *C*=Rh $_{dia}$), 181.1 (d, *J* = 51.3 Hz, 0.5C, *C*=Rh $_{dia}$), 175.9 (0.5C, *C*=O $_{Aib\ dia}$), 175.8 (0.5C, *C*=O $_{Aib\ dia}$), 175.6 (0.5C, *C*=O $_{Aib\ dia}$), 175.5 (0.5C, *C*=O $_{Aib\ dia}$), 174.8 (0.5C, *C*=O $_{Aib\ dia}$), 174.6 (0.5C, *C*=O $_{Aib\ dia}$), 174.0 (0.5C, *C*=O $_{Aib\ dia}$), 174.0 (0.5C, *C*=O $_{Aib\ dia}$), 172.9 (*C*=O $_{aMeVal}$), 156.2 (0.5C, *C*=O $_{Cbz\ dia}$), 156.2 (0.5C, *C*=O $_{Cbz\ dia}$), 138.3 (0.5C, C_{Mes}), 138.2 (0.5C, C_{Mes}), 137.3 (0.5C, C_{Mes}), 137.2 (0.5C, C_{Mes}), 136.8 (0.5C, C_{Mes}), 136.7 (0.5C, C_{Mes}), 136.0 (0.5C, C_{Bn}), 136.0 (0.5C, C_{Bn}), 135.2 (0.5C, C_{Mes}), 135.0 (0.5C, C_{Mes}), 129.4 (0.5C, CH_{Mes}), 129.3 (0.5C, CH_{Mes}), 128.9 (2C, 2 x CH_{Bn}), 128.9 (1C, CH_{Bn}), 128.4 (2C, 2 x CH_{Bn}), 128.3 (x CH_{Bn}), 128.2 (0.5C, CH_{Mes}), 128.1 (0.5C, CH_{Mes}), 124.1 (0.5C, $CH_{imid\ dia}$), 124.0 (0.5C, $CH_{imid\ dia}$), 122.4 (0.5C, $CH_{imid\ dia}$), 122.2 (0.5C, $CH_{imid\ dia}$), 96.9 (d, J_{Rh-C} = 5.6 Hz, 0.5 x $CH_{COD\ dia}$), 96.7 (d, J_{Rh-C} = 6.6 Hz, 0.5 x CH_{COD}

$_{dia}$), 96.5 (d, $J_{Rh\text{-}C}$ = 6.6 Hz, 0.5 x CH$_{COD\ dia}$), 96.3 (d, $J_{Rh\text{-}C}$ = 5.6 Hz, 0.5 x CH$_{COD\ dia}$), 69.2 (d, $J_{Rh\text{-}C}$ = 14.4 Hz, 0.5C, CH=CH$_{cod\ dia}$), 69.0 (d, $J_{Rh\text{-}C}$ = 13.8 Hz, 0.5C, CH=CH$_{cod\ dia}$), 68.4 (d, $J_{Rh\text{-}C}$ = 13.8 Hz, 0.5C, CH=CH$_{cod\ dia}$), 68.2 (d, $J_{Rh\text{-}C}$ = 14.4 Hz, 0.5C, CH=CH$_{cod\ dia}$), 67.7 (0.5C, 0.5 x CH_2Ph), 67.7 (0.5C, 0.5 x CH_2Ph), 63.2 (0.5C, $C_{MeVal\ dia}$), 63.2 (0.5C, $C_{MeVal\ dia}$), 57.6 (0.5C, $C_{Aib\ dia}$), 57.5 (0.5C, $C_{Aib\ dia}$), 57.1 (0.5C, $C_{Aib\ dia}$), 57.0 (0.5C, $C_{Aib\ dia}$), 56.9 (0.5C, $C_{Aib\ dia}$), 56.8 (0.5C, $C_{Aib\ dia}$), 56.8 (0.5C, $C_{Aib\ dia}$), 56.7 (0.5C, $C_{Aib\ dia}$), 51.4 (0.5C, CH_2-Imid $_{dia}$), 51.3 (0.5C, CH_2-Imid $_{dia}$), 41.3 (0.5C, CH_2NH_{dia}), 41.0 (0.5C, CH_2NH_{dia}), 35.7 (1C, CH(CH_3)$_2$), 34.2 (0.5C, $CH_{2\ COD\ dia}$), 34.0 (0.5C, $CH_{2\ COD\ dia}$), 31.9 (0.5C, $CH_{2\ COD\ dia}$), 31.8 (0.5C, $CH_{2\ COD\ dia}$), 29.9 (0.5C, $CH_{2\ COD\ dia}$), 29.5 – 28.3 ($CH_{2\ COD\ dia}$), 28.2 (0.5C, $CH_{2\ COD\ dia}$), 27.1 - 26.5 (4 X CH$_3$C $_{Aib\ dia}$), 24.2 – 23.0 (4 X CH$_3$C $_{Aib\ dia}$), 21.2 (CH$_3$Ph), 19.9 (0.5C, 0.5 x CH$_3$Ph$_{dia}$), 19.9 (0.5C, 0.5 x CH$_3$Ph$_{dia}$), 18.0 (1C, CH$_3$Ph), 17.7 (0.5C, CH$_3$CH $_{MeVal\ dia}$), 17.6 (0.5C, CH$_3$CH $_{MeVal\ dia}$), 17.4 (1C, CH$_3$CH $_{MeVal\ dia}$), 17.3 (1, CH$_3$CH $_{MeVal\ dia}$). **FTIR (neat)** ν_{max} = 3306, 2932, 1651, 1526, 1382, 1228, 698 cm^{-1}. **HR – MS** (ESI, positive ion mode) – *m/z* for $[C_{52}H_{76}N_8O_7Rh]^+$ 1027.4892, found 1027.4853. **MP** 152-154 °C.

3.38 Precursor 11, [(Cbz-(L-αMeVal)$_2$Aib$_4$NH(CH$_2$)$_2$(Im-Ph)]$^+$Br$^-$

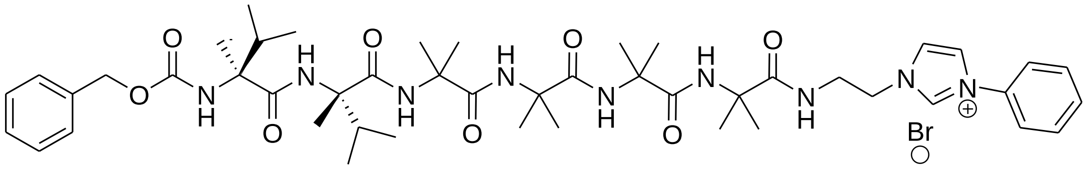

Following general procedure 4, to a solution of Cbz-(L-αMeVal)$_2$Aib$_4$OH (0.059 g, 0.098 mmol) in dry dichloromethane (3 mL) was added *N*-(3-dimethylaminopropyl)-*N'*-ethylcarbodiimide EDC.HCl (0.019 g, 0.098 mmol). The mixture was stirred at 20 °C for 10 minutes then was cooled to 0°C and triethylamine (0.015 mL, 0.098 mmol) was added. The mixture was stirred for 2 hours at 20 °C, then was concentrated, then dissolved in acetonitrile (3 mL). 1-(2-Aminoethyl)-3-(phenyl)-1H-imidazol-3-ium bromide, hydrobromide salt **5** (0.046 g, 0.17 mmol) was added to the mixture, followed with triethylamine (0.015 mL, 0.098 mmol) and the suspension was heated at 80 °C for 3 days. The mixture was concentrated under vacuum; the residue was dissolved in ethyl acetate and washed with water, the organic phase was dried (Na_2SO_4), concentrated and purified by chromatography on silica (eluent petroleum ether: ethyl acetate 1:1) to afford the compound as colorless oil (0.07 g, 0.072 mmol, 73 %). **^{1}H NMR** (500 MHz, $CDCl_3$) δ 9.94 (s, 1H, NCH_{imid}N), 8.15 (s, 1H,CH_{imid}), 7.84 (s, 1H, NH), 7.79 (s, 1H, NH), 7.72 (dd, J = 8.3, 3.9 Hz, 1H, NHCH$_2$), 7.68 (dd, J = 7.6, 2.1 Hz, 2H, 2 x C$H_{Ph\ ortho}$), 7.53 – 7.45 (m, 4H, NH, CH_{im}, 2 x C$H_{Ph\ meta}$), 7.45 – 7.41 (m, 1H, C$H_{Ph\ para}$), 7.37 – 7.32 (dd, J = 7.17, 1.61 Hz, 2H, 2 x C$H_{Bn\ ortho}$), 7.30 – 7.19 (m, 4H, NH, 3 x CH_{Bn}), 6.90 (s, 1H, NH), 6.28 (s, 1H, NH), 5.09 (d, J = 12.4 Hz, 1H, CH_2Ph), 4.97 (d, J = 12.4 Hz, 1H, CH_2Ph), 4.93 – 4.80 (m, 1H, CH_2N$_{imid}$), 4.59 (ddd, J = 12.1, 9.5, 4.7 Hz, 1H, CH_2N$_{imid}$), 4.14 – 4.01 (m, 1H, CH_2NH), 3.36 – 3.24 (m, 1H, CH_2NH), 2.15 (hept, J = 6.6 Hz, 1H, CH(CH$_3$)$_2$), 1.60 (hept, J = 6.7 Hz, 1H, CH(CH$_3$)$_2$), 1.45 (s, 3H, CH_{3Aib}), 1.40 (s, 3H, CH_{3Aib}), 1.38 (s, 3H, CH_{3Aib}), 1.36 (s, 6H, 2 x CH_{3Aib}), 1.34 – 1.31 (m, 9H, 3 x CH_{3Aib}), 1.30 (s, 3H, CH_{3Aib}), 1.24 (s, 3H, CH_{3Aib}), 0.92 (d, J = 6.9 Hz, 3H, (CH_3)$_2$CH), 0.84 (d, J = 6.9 Hz, 3H, (CH_3)$_2$CH), 0.77 (d, J = 6.9 Hz, 3H, (CH_3)$_2$CH), 0.73 (d, J = 6.7 Hz, 3H, (CH_3)$_2$CH). **^{13}C NMR** (126 MHz, $CDCl_3$) δ 176.8 (C=O$_{Aib}$), 176.5 (C=O$_{Aib}$), 175.9 (C=O$_{Aib}$), 175.7 (C=O$_{Aib}$), 173.9 (C=O$_{aMeVal}$), 172.9 (C=O$_{aMeVal}$), 156.8 (C=O$_{Cbz}$), 136.4 (C_{Bn}), 135.7 (NCH$_{imid}$N), 135.1 (C_{Ph}), 130.4 (2C, 2 x CH$_{Ph\ meta}$), 130.1 (CH$_{Ph\ para}$), 128.6 (2C, 2 x CH$_{Bn\ meta}$), 128.3 (CH$_{Bn\ para}$),, 128.3 (2C, 2 x CH$_{Bn\ ortho}$), 125.2 (CH$_{imid}$), 122.5 (2C, 2 x CH$_{Ph\ ortho}$), 120.4 (CH$_{imid}$), 67.2 (CH$_2$Ph), 63.5 (C_{aMeVal}), 62.5 (C_{aMeVal}), 57.2 (C_{Aib}), 56.8 (C_{Aib}), 56.7 (C_{Aib}), 56.5 (C_{Aib}), 50.5 (CH$_2$N$_{imid}$), 40.1 (CH$_2$NH), 35.6 (CH(CH$_3$)$_2$), 35.1 (CH(CH$_3$)$_2$), 27.9 (CH$_{3Aib}$), 27.2 (CH$_{3Aib}$), 27.2 (CH$_{3Aib}$), 27.0 (CH$_{3Aib}$), 22.9 (CH$_{3Aib}$), 22.8 (CH$_{3Aib}$), 22.8 (CH$_{3Aib}$), 22.7 (CH$_{3Aib}$), 18.1 (CH$_3$C$_{aMeVal}$), 17.4 (1C, (CH$_3$)$_2$CH), 17.3 (1C, (CH$_3$)$_2$CH), 17.3 (1C, (CH$_3$)$_2$CH), 17.2 (1C, (CH$_3$)$_2$CH), 17.0 (CH$_3$C$_{aMeVal}$). **FTIR (neat)** ν_{max} = 3300, 2990, 1656, 1530, 1454, 1390, 1260, 1225 cm^{-1}. **HR – MS** (ESI, positive ion mode) – *m/z* for $[C_{47}H_{70}N_9O_8]^+$ 888.5342, found 888.5316.

3.39 Foldamer 17 [(Cbz-(L-αMeVal)$_2$Aib$_4$NH(CH$_2$)$_2$-NHC-Ph)Rh(Cl)(COD)]

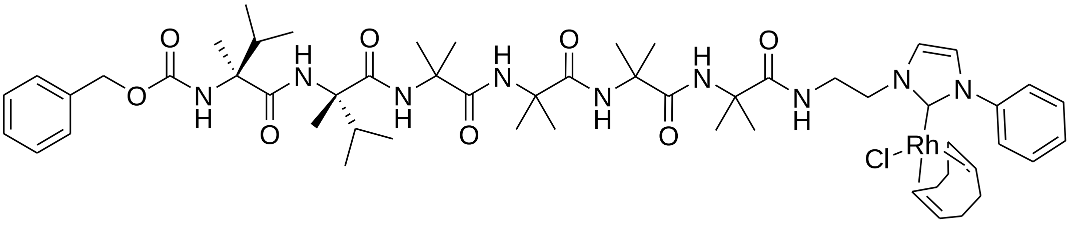

Following general procedure 6 with [Cbz-(L-αMeVal)$_2$Aib$_4$NH(CH$_2$)$_2$(Im-Ph)]$^+$Br$^-$ **11** (0.041 g, 0.042 mmol), dichloromethane (2 mL), silver oxide (0.01 g, 0.042 mmol), [Rh(COD)Cl]$_2$ (0.011 g, 0.021 mmol), the compound was obtained as a yellow solid (0.024 g, 0.020 mmol, 48 %) as a mixture of diastereomers (ratio 58.2 : 41.8 determined by line fitting of ^{1}H signal of C*H*$_{imid}$ confirmed by integration of other ^{1}H and ^{13}C signals). **^{1}H NMR** (500 MHz, CDCl$_3$) δ 8.18 (dd, *J* = 7.6, 2.1 Hz, 1.16H, 2 x C*H*$_{Ph\ ortho\ dia\ maj}$), 8.16 (dd, *J* = 7.6, 2.1 Hz, 0.84H, 2 x C*H*$_{Ph\ ortho\ dia\ min}$), 7.95 (dd, *J* = 7.6, 3.7 Hz, 0.58H, 0.58 x N*H*CH$_{2\ dia\ maj}$), 7.85 (t, *J* = 5.9 Hz, 0.42H, 0.42 x N*H*CH$_2$ $_{dia\ min}$), 7.80 (s, 0.5H, 0.5 NH$_{dia}$), 7.77 (s, 1H, 2 x 0.5 NH$_{dia}$), 7.72 (s, 0.5H, 0.5 x NH$_{dia}$), 7.69 (d, *J* = 2.0 Hz, 0.58H, C*H*$_{imid\ dia\ maj}$), 7.57 (s, 0.5H, 0.58 NH), 7.56 (s, 0.5H, 0.42 NH), 7.53 – 7.47 (m, 2H, 2 x C*H*$_{Ph\ meta}$), 7.42 – 7.38 (m, 2.42H, N*H*, C*H*$_{Ph\ para}$, 0.42 C*H*$_{imid\ dia\ min}$), 7.38 – 7.31 (m, 5H, 5 x C*H*$_{Bn}$), 7.06 (d, *J* = 2.0 Hz, 0.58H, C*H*$_{imid\ dia\ maj}$), 7.03 (d, *J* = 2.0 Hz, 0.42H, C*H*$_{imid\ dia\ min}$), 6.40 – 6.37 (m, 1H, N*H*), 5.43 (dd, *J* = 5.7, 3.0 Hz, 0.29H, C*H*$_2$-Imid$_{dia\ maj}$), 5.39 (dd, *J* = 5.7, 3.0 Hz, 0.29H, C*H*$_2$-Imid$_{dia\ maj}$), 5.39 – 5.36 (m, 1H, N*H*Cbz), 5.22 – 5.19 (m, 1H, 0.42H, C*H*$_2$-Imid$_{dia\ min}$), 5.19- 5.15 (m, 1.16H, C*H*$_2$Ph$_{dia\ maj}$), 5.06 – 4.98 (m, 1.68H, 0.84 C*H*$_2$Ph$_{dia\ min,}$ 0.84 x CH=CH$_{COD\ dia\ min}$), 4.95 – 4.86 (m, 1.16H, 1.16 x CH=CH$_{COD\ dia\ maj}$), 4.63 (ddd, *J* = 14.2, 9.0, 2.9 Hz, 0.58H, C*H*$_2$-Imid$_{dia\ maj}$), 4.52 (ddd, *J* = 13.8, 8.5, 5.3 Hz, 0.42H, C*H*$_2$-Imid$_{dia\ min}$), 4.19 – 4.08 (m, 0.58H, C*H*$_2$NH$_{dia\ maj}$), 4.06 – 3.97 (m, 0.42 C*H*$_2$NH$_{dia}$ $_{min}$), 3.81 (q, *J* = 4.3, 3.4 Hz, 0.21H, C*H*$_2$NH$_{dia\ min}$), 3.78 (q, *J* = 5.4, 4.3 Hz, 0.21H, C*H*$_2$NH$_{dia\ min}$), 3.59 – 3.55 (m, 0.29H, C*H*$_2$NH$_{dia\ maj}$), 3.55 – 3.53 (m, 0.29H, C*H*$_2$NH$_{dia\ maj}$), 3.46 – 3.39 (m, 1H, C*H*=CH$_{COD}$), 2.59 – 2.51 (m, 1H, C*H*=CH$_{COD}$), 2.44 – 2.33 (m, 1H, C*H*$_{2COD\ t}$), 2.34 – 2.20 (m, 1H, C*H*$_{2COD\ q}$), 2.11 (dtd, J = 13.2, 7.1, 3.8 Hz, 1H, C*H*$_{2COD\ v}$), 1.91 – 1.84 (m, 1H, C*H*(CH$_3$)$_2$), 1.86 – 1.77 (m, 1H, C*H*$_{2COD\ t}$), 1.79 – 1.69 (m, 2H, C*H*$_{2COD\ q}$, C*H*$_{2COD\ v}$), 1.64 (s, 1.74H, C*H*$_3$C $_{dia\ maj}$), 1.62 (s, 1.26H, C*H*$_3$C$_{dia\ min}$), 1.58 – 1.44 (m, 1H, C*H*(CH$_3$)$_2$), 1.55 – 1.43 (m, 2H, C*H*$_{2COD}$ $_u$), 1.53 (s, 1.74H, C*H*$_3$C$_{Aib\ dia\ maj}$), 1.51 (s, 1.26H, C*H*$_3$C$_{Aibdia\ min}$), 1.50 (s, 1.74H, C*H*$_3$C$_{Aib\ dia\ maj}$), 1.48 (s, 3H, C*H*$_3$C$_{Aib}$), 1.47 (s, 3H, C*H*$_3$C$_{Aib}$), 1.46 (s, 4.26H, C*H*$_3$C$_{Aib}$), 1.44 – 1.41 (9H, 2 x C*H*$_3$C$_{Aib}$, C*H*$_3$C$_{aMeVal}$), 1.38 (C*H*$_3$C$_{aMeVal}$), 0.97 (d, *J* = 6.9 Hz, 3H, C*H*$_3$CH$_{aMeVal}$), 0.94 (d, *J* = 6.6 Hz, 3H, C*H*$_3$CH$_{aMeVal}$), 0.80 – 0.74 (m, 6H, 2 x C*H*$_3$CH$_{aMeVal}$). **^{13}C NMR** (126 MHz, CDCl$_3$) δ 182.33 (d, *J* = 50.9 Hz, *C*Rh $_{dia\ min}$) 181.7 (d, *J* = 50.9 Hz, *C*Rh $_{dia\ maj}$), 176.3 (0.42C, *C*=O $_{Aib\ dia\ min}$), 176.3 (0.58C, *C*=O $_{Aib\ dia\ maj}$), 176.0 (0.58C, *C*=O $_{Aib\ dia\ maj}$), 176.0 (0.42C, *C*=O $_{Aib\ dia\ min}$), 175.3 (0.42C, *C*=O $_{Aib\ dia\ maj}$), 175.2 (0.42C, *C*=O $_{Aib\ dia\ min}$), 175.2 (0.42C, *C*=O $_{Aib\ dia\ maj}$), 174.9 (0.42C, *C*=O $_{Aib\ dia\ min}$), 172.5 (*C*=O $_{aMeVal\ dia}$), 172.5 (*C*=O $_{aMeVal\ dia}$), 156.4 (*C*=O $_{Cbz}$), 140.8 (*C*$_{Ph}$), 135.9 (*C*$_{Bn}$), 128.8 (4C, 2 x *C*H$_{Bn,}$ 2 x *C*H$_{Ph\ meta}$), 128.8 (*C*H$_{Bn}$), 128.6 (2C, 2 x *C*H$_{Bn}$), 127.6 (0.42C, *C*H$_{Ph\ para\ dia\ min}$), 127.5 (0.58C, *C*H$_{Ph\ para\ dia\ maj}$), 124.9 (0.84C, 2 x *C*H$_{Ph}$ $_{ortho\ dia\ min}$), 124.8 (1.16C, 2x *C*H$_{Ph\ ortho\ dia\ maj}$), 124.0 (*C*H$_{Imid\ dia\ maj}$), 123.7 (*C*H$_{Imid\ dia\ min}$), 120.9 (*C*H$_{Imid\ dia\ maj}$), 120.7 (*C*H$_{Imid\ dia\ min}$), 97.6 (d, $J_{Rh\text{-}C}$ = 6.5 Hz, 0.5C, *C*H=CH$_{cod\ dia}$), 97.4 (d, $J_{Rh\text{-}C}$ = 6.8 Hz, 0.5C, *C*H=CH$_{cod\ dia}$), 97.1 (d, $J_{Rh\text{-}C}$ = 6.5 Hz, 0.5C, *C*H=CH$_{cod\ dia}$), 97.0 (d, $J_{Rh\text{-}C}$ = 6.8 Hz, 0.5C, *C*H=CH$_{cod\ dia}$), 69.3 (d, $J_{Rh\text{-}C}$ = 14.5 Hz, 0.42C, *C*H=CH$_{cod\ dia}$ $_{min}$), 69.2 (d, $J_{Rh\text{-}C}$ = 14.5 Hz, 0.58C, *C*H=CH$_{cod\ dia\ maj}$), 68.7 (d, $J_{Rh\text{-}C}$ = 12.35 Hz, 0.58C, *C*H=CH$_{cod\ dia\ maj}$), 68.6 (d, $J_{Rh\text{-}C}$ = 12.3 Hz, 0.42C, *C*H=CH$_{cod\ dia\ min}$), 67.8 (*C*H$_2$Ph), 63.6 (*C*$_{MeVal}$), 62.5 (*C*$_{MeVal}$), 57.5 (0.5C, *C*$_{Aib\ dia\ maj}$), 57.4 (0.5C, *C*$_{Aib\ dia\ min}$), 57.0 (0.5C, *C*$_{Aib\ dia\ maj}$), 57.0 (0.5C, *C*$_{Aib\ dia\ min}$), 57.0 (0.5C, *C*$_{Aib\ dia\ maj}$), 57.0 (0.5C, *C*$_{Aib\ dia\ min}$), 56.8 (0.5C, *C*$_{Aib\ dia\ min}$), 56.8 (0.5C, *C*$_{Aib\ dia\ maj}$), 50.8 (0.58C, *C*H$_2$-Imid$_{dia\ maj}$), 50.8 (0.42C, *C*H$_2$-Imid$_{dia\ min}$), 41.3 (0.58C, *C*H$_2$NH$_{dia}$ $_{maj}$), 40.6 (0.42C, *C*H$_2$NH$_{dia\ min}$), 36.1 (*C*H(CH$_3$)$_2$), 35.8 (1C, *C*H(CH$_3$)$_2$), 33.5 (0.58C, *C*H$_{2\ COD\ t\ dia\ maj}$), 33.3 (0.42C, *C*H$_{2\ COD\ t\ dia\ min}$), 31.9 (0.42C, *C*H$_{2\ COD\ u\ dia\ min}$), 31.9 (0.58C, *C*H$_{2\ COD\ dia\ maj}$), 29.3 (0.58C, *C*H$_{2\ COD\ dia\ maj}$), 29.1 (0.42C, *C*H$_{2\ COD\ u\ dia\ min}$), 29.0 (0.42C, *C*H$_{2\ COD\ u\ dia\ min}$), 28.6 (0.58C, *C*H$_{2\ COD\ dia\ maj}$), 29.0 - 26.6 (4 X *C*H$_3$C $_{Aib\ dia}$), 23.8 – 22.6 (4 X *C*H$_3$C $_{Aib\ dia}$), 18.1 (*C*H$_3$C$_{aMeVal}$), 18.1 (*C*H$_3$C$_{aMeVal}$), 17.4 (*C*H$_3$CH$_{aMeVal}$), 17.3 (*C*H$_3$CH$_{aMeVal}$), 17.2 (*C*H$_3$CH$_{aMeVal}$), 17.1 (*C*H$_3$CH$_{aMeVal}$). **FTIR (neat)** ν_{max} = 3304, 2984, 2939, 1653, 1526, 1499, 1260, 1229, 909, 728, 695, 645

cm^{-1}. **HR – MS** (ESI, positive ion mode) – *m/z* for $[C_{55}H_{81}N_9O_8Rh]^+$ 1098.5258, found 1098.5221. **MP** 145-150 °C

3.40 Precursor 12, [Cbz-(L-αMeVal)$_2$Aib$_4$NH(CH$_2$)$_2$(Im-Mes)]$^+$Br$^-$

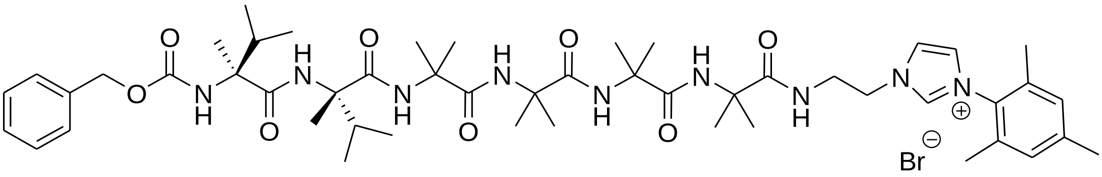

Following general procedure 4, to a solution of Cbz-(L-aMeVal)$_2$Aib$_4$OH (0.040 g, 0.066 mmol) in dry dichloromethane (3 mL) was added *N*-(3-dimethylaminopropyl)-*N′*-ethylcarbodiimide EDC.HCl (0.013 g, 0.066 mmol). The mixture was stirred at 20 °C for 10 minutes then was cooled to 0°C and triethylamine (0.01 mL, 0.066 mmol) was added. The mixture was stirred for 2 hours at 20 °C, then was concentrated, then dissolved in acetonitrile (3 mL). 1-(2-Aminoethyl)-3-(mesityl)-1H-imidazol-3-ium bromide, hydrobromide salt **6** (0.043 g, 0.112 mmol, 1.7 equiv.) was added to the mixture, followed with triethylamine (0.010 mL, 0.066 mmol) and the suspension was heated at 80 °C for 3 days. The mixture was concentrated under reduced pressure; the residue was purified by chromatography on silica (eluent CH_2Cl_2:MeOH 90:10) to afford the compound as colorless oil (0.038 g, 0.034 mmol, 53 %). **^{1}H NMR** (400 MHz, $CDCl_3$) δ 9.64 (s, 1H, NC*H*N), 8.19 (d, *J* = 2.1 Hz, 1H C*H$_{imid}$*), 7.86 (s, 1H, N*H*), 7.79 (s, 1H, N*H*), 7.71 (dd, *J* = 8.1, 4.1 Hz, 1H, N*H*CH$_2$), 7.45 (s, 1H, N*H*), 7.34 (d, *J* = 7.1 Hz, 2H, 2 x C*H*$_{Bn\ ortho}$), 7.27 (t, J = 7.2 Hz, 2H, 2 x C*H*$_{Bn\ meta}$), 7.22 (t, *J* = 3.6 Hz, 1H, C*H*$_{Bn\ para}$), 7.20 (1H, N*H*), 7.12 (s, 1H, N*H*), 6.99 (d, *J* = 1.9 Hz, 1H, C*H$_{imid}$*), 6.92 (s, 1H, C*H*$_{Mes}$), 6.90 (s, 1H, C*H*$_{Mes}$), 6.61 (s, 1H, N*H*), 5.08 (d, *J* = 12.4 Hz, 1H, C*H*$_2$Ph), 4.96 (d, *J* = 12.4 Hz, 1H, C*H*$_2$Ph), 4.85 (dd, *J* = 13.5, 5.2 Hz, 1H, C*H*$_2$N$_{imid}$), 4.62 (dd, *J* = 13.5, 8.6 Hz, 1H, C*H*$_2$N$_{imid}$), 4.10 – 4.0 (m, 1H, C*H*$_2$NH), 3.29 – 3.20 (m, 1H, C*H*$_2$NH), 2.27 (s, 3H, C*H*$_3$Ph), 2.20 – 2.09 (m, 1H, C*H*(CH$_3$)$_2$), 2.06 (s, 3H, C*H*$_3$Ph), 2.00 (s, 3H, C*H*$_3$Ph), 1.63 (hept, *J* = 6.8 Hz, 1H, C*H*(CH$_3$)$_2$), 1.42 (s, 3H, C*H*$_3$), 1.39 (s, 3H, C*H*$_3$), 1.37 – 1.33 (m, 9H, 3 x C*H*$_3$), 1.33 – 1.30 (s, 9H, C*H*$_3$CH, 2 x C*H*$_3$), 1.29 (s, 3H, C*H*$_3$CH), 1.15 (s, 3H, C*H*$_3$), 0.92 (d, *J* = 6.7 Hz, 3H, (C*H*$_3$)$_2$CH), 0.82 (d, *J* = 6.8 Hz, 3H, (C*H*$_3$)$_2$CH), 0.77 (d, *J* = 6.7 Hz, 3H, (C*H*$_3$)$_2$CH), 0.71 (d, *J* = 6.8 Hz, 3H, (C*H*$_3$)$_2$CH). **^{13}C NMR** (101 MHz, $CDCl_3$) δ 176.8 (*C*=O$_{Aib}$), 176.4 (*C*=O$_{Aib}$), 176.0 (*C*=O$_{Aib}$), 175.7 (*C*=O$_{Aib}$), 174.3 (*C*=O$_{aMeVal}$), 173.1 (*C*=O$_{aMeVal}$), 156.9 (*C*=O$_{Cbz}$), 140.9 (*C*$_{Mes}$-CH$_{3para}$), 137.8 (N*C*HN$_{imid}$), 136.6 (*C*$_{Bn}$), 134.9 (*C*$_{Mes}$-CH$_{3orhto}$), 134.4 (*C*$_{Mesl}$-CH$_{3ortho}$), 131.3 (*C*$_{Mes}$), 129.8 (*C*H$_{Mes}$), 129.6 (*C*H$_{Mes}$), 128.6 (2C, 2 x *C*H$_{Bn\ meta}$), 128.2 (*C*H$_{Bn\ para}$), 128.1 (2C, 2 x *C*H$_{Bn\ ortho}$), 125.3 (*C*H$_{imid}$), 122.5 (*C*H$_{imid}$), 67.1 (*C*H$_2$Ph), 63.5 (*C*$_{aMeVal}$), 62.6 (*C*$_{aMeVal}$), 57.2 (*C*$_{Aib}$), 56.8 (*C*$_{Aib}$), 56.7 (*C*$_{Aib}$), 56.5 (*C*$_{Aib}$), 50.7 (*C*H$_2$N$_{imid}$), 40.7 (*C*H$_2$NH), 35.5 (*C*H(CH$_3$)$_2$), 35.1 (*C*H(CH$_3$)$_2$), 28.0 (*C*H$_{3Aib}$), 27.2 (*C*H$_{3Aib}$), 27.2 (*C*H$_{3Aib}$), 27.0 (*C*H$_{3Aib}$), 22.8 (2C, 2 x *C*H$_{3Aib}$), 22.8 (*C*H$_{3Aib}$), 22.3 (*C*H$_{3Aib}$), 21.2 (*C*H$_{3Mespara}$), 18.0 (*C*H$_3$C$_{aMeVal}$), 17.7 (*C*H$_{3Mesortho}$), 17.6 (*C*H$_{3Mesortho}$), 17.4 (1C, (*C*H$_3$)$_2$CH), 17.3 (1C, (*C*H$_3$)$_2$CH), 17.3 (1C, (*C*H$_3$)$_2$CH), 17.2 (1C, (*C*H$_3$)$_2$CH), 16.7 (*C*H$_3$C$_{aMeVal}$). **FTIR (neat)** ν_{max} = 3290, 2980, 1653, 1526, 1450, 1380, 1360, 1225, 1212, 1070, 1026, 734, 697 cm^{-1}. **HR – MS** (ESI, positive ion mode) – *m/z* for $[C_{50}H_{76}N_9O_8]^+$ 930.5811, found 930.5803.

3.41 Foldamer 18, [(Cbz-(L-αMeVal)$_2$Aib$_4$NH(CH$_2$)$_2$-NHC-Mes)Rh(Cl)(COD)]

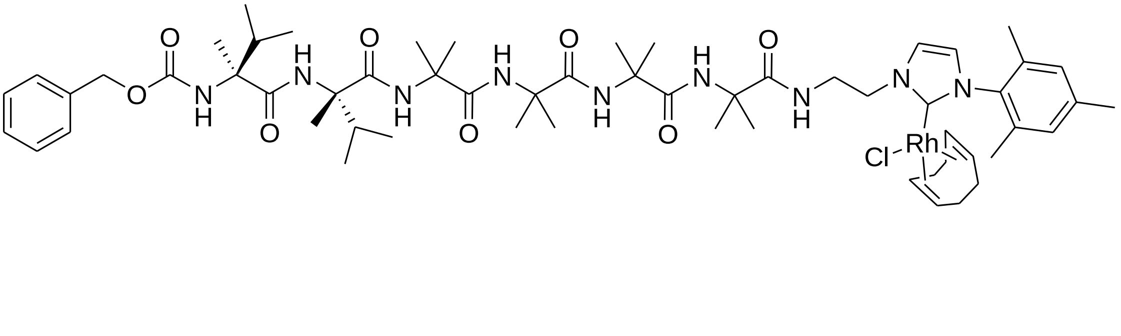

Following general procedure 6 with [Cbz-(L-αMeVal)$_2$Aib$_4$NH(CH$_2$)$_2$(Im-Mes)]$^+$Br$^-$ (precursor **12**) (0.038 g, 0.038 mmol), dichloromethane (5 mL), silver oxide (0.008 g, 0.038 mmol), [Rh(COD)Cl]$_2$ (0.01 g, 0.018 mmol), the compound was obtained as a yellow solid (0.017 g, 0.015 mmol, 39 %) as a mixture of diastereomers (ratio 52:48 determined by integration of ^{1}H NMR signals). **^{1}H NMR** (400 MHz, CDCl$_3$) δ 7.88 – 7.82 (m, 1H, N*H*CH$_{2\ dias}$), 7.77 (s, 0.5H, N*H*$_{dia}$), 7.75 (s, 0.5H, N*H*$_{dia}$), 7.74 (s, 0.5H, N*H*$_{dia}$), 7.67 (s, 0.5H, N*H*$_{dia}$), 7.60 (d, *J* = 1.8 Hz, 0.5H, C*H*$_{Imiddia}$), 7.56 (s, 0.5H, N*H*$_{dia}$), 7.55 (s, 0.5H, N*H*$_{dia}$), 7.39 – 7.34 (m, 5H, 5 x C*H*$_{Ph}$), 7.37 (d, *J* = 1.9 Hz, 0.5H, C*H*$_{Imid\ dia}$), 7. 30 (s, 0.5H, N*H*$_{dia}$), 7.27 (s, 0.5H, N*H*$_{dia}$), 7.06 (s, 1H, C*H*$_{Mesdias}$), 6.90 (s, 0.5H, C*H*$_{Mes\ dia}$), 6.87 (s, 0.5H, C*H*$_{Mes\ dia}$), 6.66 (d, *J* = 1.9 Hz, 0.5H, C*H*$_{Imid\ dia}$), 6.60 (d, *J* = 1.8 Hz, 0.5H, C*H*$_{Imid\ dia}$), 6.37 (s, 0.5H, N*H*$_{dia}$), 6.36 (s, 0.5H, N*H*$_{dia}$), 5.61 (ddd, *J* = 17.1, 8.8, 3.7 Hz, 1H, C*H*$_2$Imid$_{dias}$), 5.24 (s, 0.5H, N*H*Cbz $_{dia}$), 5.23 (s, 0.5H, N*H*Cbz $_{dia}$), 5.19 (d, *J* = 12.1, 0.5H, C*H*$_2$Ph$_{dia}$), 5.19 (d, *J* = 12.1, 0.5H, C*H*$_2$Ph$_{dia}$), 5.03 (d, *J* = 12.1, 1H, C*H*$_2$Ph$_{dias}$), 4.91 – 4.79 (m, 1H, C*H*=CH$_{COD\ dias}$), 4.77 – 4.68 (m, 1H, C*H*=CH$_{COD\ dias}$), 4.41 (q, *J* = 5.0, 3.3 Hz, 0.5H, C*H*$_2$Imid$_{dia}$), 4.31 – 4.23 (m, 0.5H, C*H*$_2$NH$_{dia}$), 4.27 – 4.22 (m, 0.5H, C*H*$_2$Imid$_{dia}$), 4.23 – 4.11 (m, 0.5H, C*H*$_2$NH$_{dia}$), 3.93 – 3.83 (m, 1H, C*H*=CH$_{COD\ dia}$), 3.90 – 3.72 (m, 0.5H, C*H*$_2$NH$_{dia}$), 3.64 – 3.45 (m, 0.5H, C*H*$_2$NH$_{dia}$), 3.06 – 2.98 (m, 1H, C*H*=CH$_{COD\ dias}$), 2.44 (s, 1.5H, C*H*$_{3Mes\ dia}$), 2.42 (s, 1.5H, C*H*$_{3Mes\ dia}$), 2.36 (s, 3H, C*H*$_{3Mes\ dia}$) 1.63 (s, 1.5H, C*H*$_{3Mes\ dia}$), 1.59 (s, 1.5H, C*H*$_{3Mes\ dia}$), 2.49 – 2.41 (m, 1H, C*H*$_2$CH$_{COD}$), 2.41 – 2.33 (m, 1H, C*H*$_2$CH$_{COD}$), 2.22 – 2.12 (m, 1H, C*H*$_2$CH$_{COD}$), 2.05 – 1.93 (m, 1H, C*H*$_2$CH$_{COD}$), 1.90 – 1.81 (m, 1H, C*H*(CH$_3$)$_2$), 1.83 – 1.66 (m, 1H, C*H*$_2$CH$_{COD}$), 1.72 – 1.61 (m, 1H, C*H*$_2$CH$_{COD}$), 1.70 – 1.64 (m, 1H, C*H*$_2$CH$_{COD}$), 1.63 (s, 1.5H, C*H*$_3$C$_{dia}$), 1.59 (s, 1.5H, C*H*$_3$C$_{dia}$), 1.57 – 1.48 (m, 1H, C*H*(CH$_3$)$_2$), 1.56 – 1.41 (m, 1H, C*H*$_2$CH$_{COD}$), 1.52 – 1.38 (m, 24H, 8 x C*H*$_3$C), 1.25 (s, 3H, C*H*$_3$C), 0.98 (d, *J* = 6.77 Hz, 3H, (C*H*$_3$)$_2$CH), 0.96 (d, *J* = 6.77 Hz, 3H, (C*H*$_3$)$_2$CH), 0.79 (d, *J* = 6.77 Hz, 3H, (C*H*$_3$)$_2$CH), 0.78 (d, *J* = 6.77 Hz, 3H, (C*H*$_3$)$_2$CH). **^{13}C NMR** (126 MHz, CDCl$_3$) δ 180.9 (d, *J* = 50.4 Hz, 0.5C, *C*=Rh $_{dia}$), 180.3 (d, *J* = 50.1 Hz, 0.5C, *C*=Rh $_{dia}$), 176.0 (0.5C, *C*=O$_{Aib\ dia}$), 176.0 (0.5C, *C*=O$_{Aib\ dia}$), 175.9 (0.5C, *C*=O$_{Aib\ dia}$), 175.8 (0.5C, *C*=O$_{Aib\ dia}$), 175.2 (0.5C, *C*=O$_{Aib\ dia}$), 175.2 (0.5C, *C*=O$_{Aib\ dia}$), 175.1 (0.5C, *C*=O$_{Aib\ dia}$), 174.8 (0.5C, *C*=O$_{Aib\ dia}$), 172.5 (1.5C, *C*=O $_{aMeVal\ dia}$), 172.4 (0.5C, *C*=O $_{aMeVal\ dia}$), 156.3 (0.5C, *C*=O$_{Cbz\ dia}$), 156.3 (0.5C, *C*=O$_{Cbz\ dia}$), 138.3 (0.5C, *C*$_{Mes\ dia}$), 138.2 (0.5C, *C*$_{Mes\ dia}$), 137.3 (0.5C, *C*$_{Mes\ dia}$), 137.2 (0.5C, *C*$_{Mes\ dia}$), 136.8 (0.5C, *C*$_{Mes\ dia}$), 136.8 (0.5C, *C*$_{Mes\ dia}$), 135.8 (*C*$_{Bn}$), 135.3 (0.5C, *C*$_{Mes\ dia}$), 135.0 (0.5C, *C*$_{Mes\ dia}$), 129.4 (0.5C, *C*H$_{Mes\ dia}$), 129.3 (0.5C, *C*H$_{Mes\ dia}$), 128.9 (1C, *C*H$_{Bn\ dia}$), 128.9 (2C, 2 x *C*H$_{Bn\ dia}$), 128.7 (1C, *C*H$_{Bn\ dia}$), 128.7 (1C, *C*H$_{Bn\ dia}$), 128.2 (0.5C, *C*H$_{Mes\ dia}$), 128.1 (0.5C, *C*H$_{Mes\ dia}$), 124.2 (1C, *C*H$_{imid}$), 122.4 (0.5C, *C*H$_{imid\ dia}$), 122.1 (0.5C, *C*H$_{imid\ dia}$), 96.9 (d, J_{Rh-C} = 7.2 Hz, 0.5C, *C*H=CH$_{cod\ dia}$), 96.6 (d, J_{Rh-C} = 7.2 Hz, 0.5C, *C*H=CH$_{cod\ dia}$), 96.4 (d, J_{Rh-C} = 6.4 Hz, 0.5C, *C*H=CH$_{cod\ dia}$), 96.3 (d, J_{Rh-C} = 6.4 Hz, 0.5C, *C*H=CH$_{cod\ dia}$), 69.2 (d, J_{Rh-C} = 14.5 Hz, 0.5C, *C*H=CH$_{cod\ dia}$), 69.0 (d, J_{Rh-C} = 14.5 Hz, 0.5C, *C*H=CH$_{cod\ dia}$), 68.4 (d, J_{Rh-C} = 13.8 Hz, 0.5C, *C*H=CH$_{cod\ dia}$), 68.2 (d, J_{Rh-C} = 13.8 Hz, 0.5C, *C*H=CH$_{cod\ dia}$), 67.9 (*C*H$_2$Ph), 63.6 (0.5C, *C*$_{MeVal\ dia}$), 63.6 (0.5C, *C*$_{MeVal\ dia}$), 62.5 (0.5C, *C*$_{MeVal\ dia}$), 62.5 (0.5C, *C*$_{MeVal\ dia}$), 57.7 (0.5C, *C*$_{Aib\ dia}$), 57.5 (0.5C, *C*$_{Aib\ dia}$), 57.1 (0.5C, *C*$_{Aib\ dia}$), 57.0 (0.5C, *C*$_{Aib\ dia}$), 57.0 (0.5C, *C*$_{Aib\ dia}$), 57.0 (0.5C, *C*$_{Aib\ dia}$), 56.8 (0.5C, *C*$_{Aib\ dia}$), 56.8 (0.5C, *C*$_{Aib\ dia}$), 51.5 (0.5C, *C*H$_2$-Imid$_{dia}$), 51.3 (0.5C, *C*H$_2$-Imid$_{dia}$), 41.4 (0.5C, *C*H$_2$NH$_{dia}$), 41.0 (0.5C, *C*H$_2$NH$_{dia}$), 36.2 (1C, *C*H(CH$_3$)$_2$), 35.9 (1C, *C*H(CH$_3$)$_2$), 34.2 (0.5C, *C*H$_{2\ COD\ dia}$), 33.9 (0.5C, *C*H$_{2\ COD\ dia}$), 31.9 (0.5C, *C*H$_{2\ COD\ dia}$), 31.8 (0.5C, *C*H$_{2\ COD\ dia}$), 29.3 (0.5C, *C*H$_{2\ COD\ dia}$), 29.2 (0.5C, *C*H$_{2\ COD\ dia}$), 28.3 (0.5C, *C*H$_{2\ COD\ dia}$), 28.2 (0.5C, *C*H$_{2\ COD\ dia}$), 28.6 - 27.0 (4 X *C*H$_3$C $_{Aib\ dia}$), 23.8 – 22.6 (4 X *C*H$_3$C $_{Aib\ dia}$), 21.2 (1C, *C*H$_3$Ph), 20.0 (0.5C, 0.5 x *C*H$_3$Ph$_{dia}$), 19.9 (0.5C, 0.5 x *C*H$_3$Ph$_{dia}$), 18.2 (0.5C, *C*H$_3$C $_{MeVal\ dia}$), 18.1 (1C, *C*H$_3$Ph), 18.1 (0.5C, *C*H$_3$C $_{MeVal\ dia}$), 18.0 (0.5C, *C*H$_3$C $_{MeVal\ dia}$), 18.0 (0.5C, *C*H$_3$C $_{MeVal\ dia}$), 17.4 (1C, *C*H$_3$CH $_{MeVal\ dia}$), 17.3 (1C, *C*H$_3$CH $_{MeVal\ dia}$), 17.2 (1C, *C*H$_3$CH $_{MeVal\ dia}$), 17.1 (1C, *C*H$_3$CH $_{MeVal\ dia}$). **FTIR (neat)** ν_{max} = 3305, 3297, 2979, 2927, 2871, 1658, 1531, 1454, 1383, 1261, 1232, 1069, 697 cm^{-1}. **HR – MS** (ESI, positive ion mode) – *m/z* for [C$_{58}$H$_{87}$N$_9$O$_8$Rh]$^+$ 1140.5727, found 1140.5696. **MP** 136-140 °C

3.42 Precursor 13, [(Cbz-(L-αMeVal)Aib$_5$NH(CH$_2$)$_2$(Im-Mes)]$^+$Br$^-$

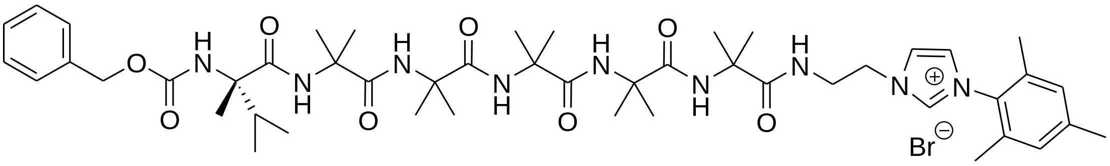

Following general procedure 4, to a solution of Cbz-(L-αMeVal)Aib_5OH (0.079 g, 0.114 mmol) in dry dichloromethane (6 mL) was added triethylamine (0.017 mL, 0.114 mmol) then *N*-(3-dimethylaminopropyl)-*N'*-ethylcarbodiimide EDC.HCl (0.022 g, 0.114 mmol). The mixture was stirred at 20 °C for 4 hours at 20 °C then was concentrated, and the residue was dissolved in dry acetonitrile (6 mL). 1-(2-Aminoethyl)-3-(mesityl)-1H-imidazol-3-ium bromide, hydrobromide salt **6** (0.075 g, 0.194 mmol, 1.7 equiv.) was added to the mixture, followed with triethylamine (0.018 mL, 0.114 mmol) and the suspension was heated at 80 °C for 3 days. The mixture was concentrated, the residue was purified by chromatography on silica (eluent CH_2Cl_2 / MeOH 90:10) to afford the compound as a white solid (0.06 g, 0.061 mmol, 54 %). **^{1}H NMR** (400 MHz, $CDCl_3$) δ 9.63 (s, 1H, $NCHN_{imid}$), 8.23 (d, *J* = 2.2 Hz, 1H, CH_{imid}), 7.84 (s, 1H, NH), 7.80 – 7.71 (m, 3H, 2 x NH_{Aib}, $NHCH_2$), 7.49 (d, *J* = 7.5 Hz, 2H, 2 x $CH_{Ph\ ortho}$), 7.36 (t, *J* = 7.5 Hz, 2H, 2 x $CH_{Ph\ meta}$), 7.31 – 7.25 (m, 3H, 2 x NH, $CH_{Ph\ para}$), 7.24 (s, 1H, NH), 7.10 (d, *J* = 2.2 Hz, 1H, CH_{imid}), 7.01 (s, 1H, CH_{Mes}), 7.00 (s, 1H, CH_{Mes}), 6.45 – 6.42 (m, 1H, NH_{CBz}), 5.22 (d, *J* = 12.8 Hz, 1H, CH_2Ph), 5.05 (d, *J* = 12.8 Hz, 1H, CH_2Ph), 4.90 (dd, *J* = 13.5, 5.3 Hz, 1H, CH_2N_{imid}), 4.74 (dd, *J* = 13.5, 8.6 Hz, 1H, CH_2N_{imid}), 4.09 – 4.02 (m, 1H, CH_2NH), 3.47 – 3.35 (m, 1H, CH_2NH), 2.48 – 2.40 (m, 1H, $CH(CH_3)_2$), 2.35 (s, 3H, CH_3Ph_{para}), 2.15 (s, 3H, CH_3Ph_{ortho}), 2.09 (s, 3H, CH_3Ph_{ortho}), 1.48 (s, 3H, CH_{3Aib}), 1.46 (s, 3H, CH_{3Aib}), 1.44 (s, 3H, CH_{3Aib}), 1.42 (s, 3H, CH_{3Aib}), 1.40 (s, 3H, CH_{3Aib}), 1.38 (s, 3H, CH_3C_{aMeVal}), 1.35 (s, 3H, CH_{3Aib}), 1.30 (s, 3H, CH_{3Aib}), 1.27 (s, 3H, CH_{3Aib}), 1.25 (s, 3H, CH_{3Aib}), 1.23 (s, 3H, CH_{3Aib}), 1.01 (d, *J* = 6.7 Hz, 3H, $(CH_3)_2CH$), 0.89 (d, *J* = 6.7 Hz, 3H, $(CH_3)_2CH$). **^{13}C NMR** (101 MHz, $CDCl_3$) δ 176.8 ($C{=}O_{Aib}$), 176.3 ($C{=}O_{Aib}$), 176.1 ($C{=}O_{Aib}$), 175.5 ($C{=}O_{Aib}$), 175.3 ($C{=}O_{Aib}$), 175.0 ($C{=}O_{aMeVal}$), 156.6 ($C{=}O_{Cbz}$), 140.9 (C_{Mes}), 137.6 (CH_{imid}), 137.1 (C_{Ph}), 134.8 (C_{Mes}), 134.3 (C_{Mes}), 131.1 (C_{Mes}), 129.8 (CH_{Mes}), 129.5 (CH_{Mes}), 128.6 (2C, 2 x $CH_{Ph\ meta}$), 127.9 ($CH_{Ph\ para}$), 127.4 (2C, 2 x $CH_{Ph\ ortho}$), 124.9 (CH_{imid}), 122.5 (CH_{imid}), 66.7 (CH_2Ph), 63.0 (C_{aMeVal}), 57.1 (C_{Aib}), 56.7 (C_{Aib}), 56.5 (C_{Aib}), 56.4 (C_{Aib}), 56.3 (C_{Aib}), 50.7 (CH_2N_{imid}), 40.4 (CH_2NH), 34.3 ($CH(CH_3)_2$), 27.7 – 26.0 (5C, 5 x CH_3C_{Aib}), 23.5 – 22.2 (5C, 5 x CH_3C_{Aib}), 21.1 (CH_3C_{Mes}), 17.6 ($CH_3C_{Meortho}$), 17.6 ($CH_3C_{Meortho}$), 17.3 (1C, $(CH_3)_2CH$), 17.2 (1C, $(CH_3)_2CH$), 16.0 ($CH_{3\ aMeVal}$). **FTIR (neat)** ν_{max} = 3294, 2981, 2935, 1653, 1526, 1453, 1383, 1361, 1260, 1225, 1212, 1166, 1071, 1026, 734, 697, 670 cm^{--1}. **HR – MS** (ESI, positive ion mode) – *m/z* for $[C_{48}H_{72}N_9O_8]^+$ 902.5498, found 902.5465. **MP** 134-136 °C

3.43 Foldamer 19, [(Cbz-(L-αMeVal)Aib$_5$NH(CH$_2$)$_2$-NHC-Mes)Rh(Cl)(COD)]

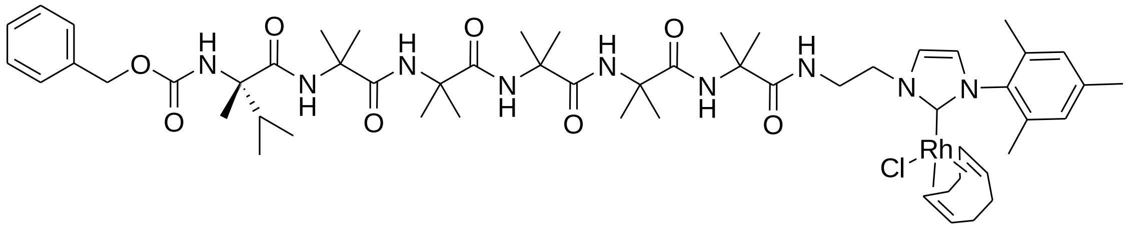

Synthesised following general procedure 6 with [Cbz-(L-αMeVal)$Aib_5NH(CH_2)_2$(Im-Mes)]$^+Br^-$ **13** (0.035 g, 0.0354 mmol), dichloromethane (5 mL), silver oxide (0.008 g, 0.036 mmol), $[Rh(COD)Cl]_2$ (0.009 g, 0.018 mmol). The compound was obtained as a yellow solid (0.032 g, 0.0276 mmol, 78 %, R_f 0.31 in eluent dichloromethane: methanol 9:1) as a mixture of diastereomers (ratio 47:53 determined by integration of the ^{1}H NMR signals of the foldamer e.g. the CH_{imid} signal). **^{1}H NMR** (500 MHz, $CDCl_3$) δ 7.89 – 7.81 (m, 1H, $NHCH_{2dias}$), 7.74 (s, 0.5H, NH_{dia1}), 7.73 (s, 0.5H, NH_{dia2}), 7.66 (s, 0.5H, NH_{dia2}), 7.59 (s, 0.5H, NH_{dia1}), 7.54 (s, 0.5H, $CH_{Imid\ dia1}$), 7.50 (bs, 1H, NH_{dias}), 7.40 (d, *J* = 1.8 Hz, 0.5H, $CH_{Imid\ dia2}$), 7.39 – 7.30 (m, 5H, 5 x CH_{Ph}), 7.29 (s, 0.5H, NH_{dia}), 7.27 (s, 0.5H, NH_{dia}), 7.03 (s, 1H, $CH_{Mes\ dias}$), 6.88 (s, 0.5H, 0.5 x $CH_{Mes\ dia2}$), 6.86 (s, 0.5H, 0.5 x $CH_{Mes\ dia1}$), 6.65 (s, 0.5H, NH_{dia}), 6.64 (d, *J* = 1.8 Hz, 0.5H, $CH_{Imid\ dia1}$), 6.62 (s, 0.5H, NH_{dia}), 6.59 (d, *J* = 1.8 Hz, 0.5H, $CH_{Imid\ dia2}$), 5.74 (s, 0.5H, $NHCbz_{dia1}$) 5.71 (s, 0.5H, $NHCbz_{dia2}$), 5.54 (ddd, *J* = 14.5, 7.4, 3.8 Hz, 1H, $CH_2Imid_{\ dia}$), 5.17 (d, *J* = 12.4 Hz, 0.5H, CH_2Ph_{dia1}), 5.16 (d, *J* = 12.4 Hz, 0.5H, CH_2Ph_{dia2}), 5.03 (d, *J* = 12.4 Hz, 0.5H, CH_2Ph_{dia2}), 5.02 (d, *J* = 12.4 Hz, 0.5H, CH_2Ph_{dia1}), 4.87 – 4.76 (m, 1H, $CH{=}CH_{COD\ dias}$), 4.70 (td, *J* = 7.9, 3.6 Hz, 1H, $CH{=}CH_{COD\ dias}$), 4.38 (td, *J* = 10.2, 5.0 Hz, 0.5H, CH_2Imid_{dia}), 4.30 – 4.16 (m, 1H, 0.5H of CH_2Imid_{dia}, 0.5H of CH_2NH_{dia}), 4.15 – 4.05 (m, 0.5H, CH_2NH_{dia}), 3.90 – 3.75 (m, 1.5H, 0.5H of CH_2NH_{dia}, $CH{=}CH_{COD\ dias}$), 3.62 – 3.53 (m, 0.5H, CH_2NH_{dia}),

3.02 (m, 1H, CH=CH$_{COD\ dias}$), 2.49 – 2.34 (m, 1 H, CH_{2a}CH$_{COD}$), 2.41 (s, 1.5H, C$H_{3Mes\ dia}$), 2.40 (s, 1.5H, C$H_{3Mes\ dia}$), 2.35 (s, 3H, CH_{3Mes}), 2.20 – 2.10 (m, 1H, CH_{2d}CH$_{COD}$), 2.05 – 1.91 (m, 2H, CH_{2c}CH$_{COD}$, CH(CH$_3$)$_2$), 1.93 – 1.80 (m, 1H, CH_{2a}CH$_{COD}$), 1.86 (s, 1.5H, C$H_{3Mes\ dia}$), 1.82 (s, 1.5H, C$H_{3Mes\ dia}$), 1.76 – 1.69 (m, 1 H, CH_{2c}CH$_{COD}$), 1.69 – 1.62 (m, 1 H, CH_{2d}CH$_{COD}$), 1.62 (s, 1.5H, CH_3C$_{dia}$), 1.57 (s, 1.5H, CH_3C$_{dia}$), 1.53 – 1.43 (m, 2H, CH_{2b}CH$_{COD}$), 1.52 (s, 1.5H, CH_3C$_{dia}$), 1.50 – 1.39 (m, 27H, 9 x CH_3C), 1.39 (s, 1.5H, CH_3C$_{dia}$), 0.97 (dd, J = 6.7 Hz, 3H, (CH_3)$_2$CH), 0.92 (dd, J = 6.9 Hz, 3H, (CH_3)$_2$CH). **^{13}C NMR** (101 MHz, CDCl$_3$) δ 181.2 (d, J = 50.4 Hz, 0.5C, C=Rh $_{dia}$), 181.1 (d, J = 51.3 Hz, 0.5C, C=Rh $_{dia}$), 176.1 (0.5C, C=O$_{Aib\ dia}$), 176.1 (0.5C, C=O$_{Aib\ dia}$), 176.0 (0.5C, C=O$_{Aib\ dia}$), 175.9 (0.5C, C=O$_{Aib\ dia}$), 175.5 (0.5C, C=O$_{Aib\ dia}$), 175.4 (0.5C, C=O$_{Aib\ dia}$), 175.0 (0.5C, C=O$_{Aib\ dia}$), 174.9 (0.5C, C=O$_{Aib\ dia}$), 174.4 (1C, C=O$_{Aib\ dias}$), 173.4 (1C, C=O$_{aMeVal\ dias}$), 156.4 (0.5C, C=O$_{Cbz\ dia1}$), 156.3 (0.5C, C=O$_{Cbz\ dia2}$), 138.3 (0.5C, $C_{Mes\ dia1}$), 138.2 (0.5C, $C_{Mes\ dia2}$), 137.1 (0.5C, $C_{Mes\ dia1}$), 137.1 (0.5C, $C_{Mes\ dia2}$), 136.7 (0.5C, $C_{Mes\ dia2}$), 136.7 (0.5C, $C_{Mesdia1}$), 136.3 (0.5C, $C_{Bn\ dia1}$), 136.3 (0.5C, $C_{Bn\ dia\ 2}$), 135.2 (0.5C, $C_{Mes\ dia2}$), 135.0 (0.5C, $C_{Mes\ dia1}$), 129.4 (0.5C, CH$_{Mes\ dia1}$), 129.3 (0.5C, CH$_{Mes\ dia2}$), 128.8 (2C, 2 x CH$_{Bn\ dias}$), 128.6 (1C, CH$_{Bn\ dias}$), 128.2 (0.5C, CH$_{Mesdia}$), 128.1 (0.5C, CH$_{Mes\ dia}$), 128.1 (1C, CH$_{Bn\ dias}$), 128.1 (1C, CH$_{Bn\ dias}$), 124.2 (0.5C, CH$_{imid\ dia1}$), 124.1 (0.5C, CH$_{imid\ dia2}$), 122.4 (0.5C, CH$_{imid\ dia2}$), 122.2 (0.5C, CH$_{imid\ dia1}$), 96.8 (d, $J_{Rh\text{-}C}$ = 6.1 Hz, 0.5C, CH=CH$_{cod\ dia}$), 96.6 (d, $J_{Rh\text{-}C}$ = 6.1 Hz, 0.5C, CH=CH$_{cod\ dia}$), 96.4 (d, $J_{Rh\text{-}C}$ = 6.7 Hz, 0.5C, CH=CH$_{cod\ dia}$), 96.2 (d, $J_{Rh\text{-}C}$ = 6.7 Hz, 0.5C, CH=CH$_{cod\ dia}$), 69.1 (d, $J_{Rh\text{-}C}$ = 13.4 Hz, 0.5C, CH=CH$_{cod\ dia}$), 69.0 (d, $J_{Rh\text{-}C}$ = 13.8 Hz, 0.5C, CH=CH$_{cod\ dia}$), 68.4 (d, $J_{Rh\text{-}C}$ = 14.6 Hz, 0.5C, CH=CH$_{cod\ dia}$), 68.2 (d, $J_{Rh\text{-}C}$ = 14.6 Hz, 0.5C, CH=CH$_{cod\ dia}$), 67.4 (CH$_2$Ph), 63.1 (0.5C, $C_{MeVal\ dia1}$), 63.1 (0.5C, $C_{MeVal\ dia2}$), 57.5 (0.5C, $C_{Aib\ dia2}$), 57.4 (0.5C, $C_{Aib\ dia1}$), 57.0 (0.5C, $C_{Aib\ dia2}$), 56.9 (0.5C, $C_{Aib\ dia1}$), 56.8 (0.5C, $C_{Aib\ dia}$), 56.7 (0.5C, $C_{Aib\ dia}$), 56.7 (1.5C, 1.5 x $C_{Aib\ dia}$), 56.6 (0.5C, $C_{Aib\ dia}$), 51.4 (0.5C, CH$_2$-Imid $_{dia1}$), 51.3 (0.5C, CH$_2$-Imid $_{dia2}$), 41.2 (0.5C, CH$_2$NH$_{dia2}$), 41.1 (0.5C, CH$_2$NH$_{dia1}$), 35.4 (0.5C, 0.5 x CH(CH$_3$)$_{2\ dia1}$), 35.3 (0.5C, 0.5 x CH(CH$_3$)$_{2\ dia2}$), 34.1 (0.5C, CH$_{2a\ COD\ dia2}$), 33.9 (0.5C, CH$_{2a\ COD\ dia1}$), 31.8 (0.5C, CH$_{2b\ COD\ dia1}$), 31.7 (0.5C, CH$_{2b\ COD\ dia2}$), 29.2 (0.5C, CH$_{2c\ COD\ dia2}$), 29.2 (0.5C, CH$_{2c\ COD\ dia1}$), 28.2 (0.5C, CH$_{2d\ COD\ dia}$), 28.2 (0.5C, CH$_{2d\ COD\ dia}$), 27.3 – 26.2 (m, 5 X CH$_3$C $_{Aib\ dia}$), 24.5 – 22.9 (m, 5 X CH$_3$C $_{Aib\ dia}$), 21.2 (CH$_3$Ph), 19.9 (0.5C, 0.5 x CH$_3$Ph$_{dia\ 2}$), 19.9 (0.5C, 0.5 x CH$_3$Ph$_{dia\ 1}$), 18.0 (0.5C, 0.5 x CH$_3$Ph$_{dia\ 1}$), 18.0 (0.5C, 0.5 x CH$_3$Ph$_{dia\ 2}$), 17.5 (0.5C, 0.5 x CH$_3$C$_{aMeVal\ dia}$), 17.5 (0.5C, 0.5 x CH$_3$CH $_{aMeVal\ dia}$), 17.4 (0.5C, 0.5 x CH$_3$CH $_{aMeVal\ dia}$), 17.3 (0.5C, 0.5 x CH$_3$C $_{aMeVal\ dia}$), 17.3 (0.5C, 0.5 x CH$_3$CH $_{aMeVal\ dia}$), 17.2 (0.5C, 0.5 x CH$_3$CH $_{aMeVal\ dia}$). **FTIR (neat)** ν_{max} = 3300, 2930, 1650, 1526, 1381, 1215, 695 cm^{-1}. **HR – MS** (ESI, positive ion mode) – m/z for [C$_{56}$H$_{83}$O$_8$N$_9$Rh]$^+$ 1112.5414, found 1112.5370. **MP** 138-142 °C

3.44 Intermediate [(+)-Mosher-NH(CH$_2$)$_2$(Im-Mes)]$^+$Br$^-$

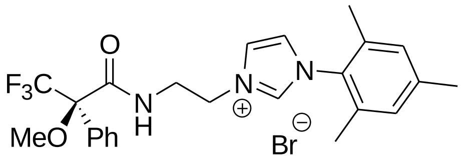

To a solution of (2R)-(+)-2-methoxy-2-phenyl-3,3,3-trifluoropropanoic acid (0.135 g, 0.58 mmol) in dry 1,2-dichloroethane (2 mL) and dry dimethylformamide (0.4 mL) was added DIPEA (0.05 mL, 0.58 mmol), diisopropylcarbodiimide (0.09 mL, 0.58 mmol), HOBt (13 mg, 0.1 mmol) and the mixture was stirred under nitrogen at 20 C for an hour, after which 1-(2-aminoethyl)-3-(2,4,6-methylphenyl)-1H-imidazol-3-ium bromide **6** (0.16 g, 0.51 mmol, 0.9 equiv) was added along with a single crystal of N,N-dimethylaminopyridine. The resulting mixture was stirred at 30 C for 4 days. Dichloromethane was added and the organic phase was washed successively with saturated aqueous ammonium chloride solution, then with saturated aqueous NaHCO$_3$ solution, dried (Na$_2$SO$_4$), filtered and concentrated under reduced pressure. The residue was purified by chromatography on silica (eluent dichloromethane methanol 95:5 then 85:15, KMnO$_4$ stain for TLC) to afford the product as a glassy solid (0.087 g, 0.2mmol, 34 %). **^{1}H NMR** (400 MHz, CDCl$_3$) δ 9.81 (s, 1H, NCHN$_{imid}$), 8.63 (t, J = 5.7 Hz, 1H, NHCH$_2$), 7.81 - 7.78 (m, 1H, CH_{imid}), 7.51 (d, J = 7.3 Hz, 2H, 2 x C$H_{Ph\ ortho}$), 7.30 (m, 3H, 3 x CH_{Ph}), 6.99- 6.92 (m, 3H, CH_{imid}, 2 x CH_{Mes}), 4.99 – 4.75 (m, 2H, CH_2N), 4.04 – 3.88 (m, 2H, CH_2NH), 3.33 (s, 3H, OCH_3), 2.33 (s, 3H, CH_3Ph$_{Mes\ para}$), 1.99 (s, 3H, CH_3Ph$_{Mes\ ortho}$), 1.89 (s, 3H, CH_3Ph$_{Mes\ ortho}$). **^{13}C NMR**

(126 MHz, $CDCl_3$) *δ* 168.3 (*C*=O), 141.4 (*C*$CH_{3Mes\ para}$), 138.0 (*C*H_{Imid}), 134.5 (*C*$CH_{3Mes\ ortho}$), 132.2 (*C*$_{Ph}$), 130.7 (*C*$CH_{3Mes\ ortho}$), 129.9 (2C, 2 x *C*$H_{Mes\ meta}$), 129.5 (*C*$H_{Ph\ para}$), 128.7 (*C*$_{Mes}$), 127.8 (2C, 2 x *C*$H_{Ph\ ortho}$), 124.0 (q, *J* = 289.2 Hz, *C*F_3), 123.8 (*C*H_{Imid}), 122.8 (*C*H_{Imid}), 83.9 (d, *J* = 25.7 Hz, *C*CF_3), 55.1 (O*C*H_3), 49.2 (*C*H_2Imid), 39.3 (*C*H_2NH), 21.2 (*C*H_3Mes_{para}), 17.5 (*C*H_3Mes_{ortho}), 17.5 (*C*H_3Mes_{ortho}). **FTIR (neat)** ν_{max} = 3316, 2966, 1686, 1647, 1561, 1260, 1163, 1104, 801 cm^{-1}. **HR – MS** (ESI, positive ion mode) – m/z for $[C_{24}H_{27}N_3F_3O_2]^+$ 446.2050, found 446.2041.

3.45 Foldamer 20, [(+)-MosherNH(CH_2)$_2$-NHC-Mes)Rh(Cl)(COD)]

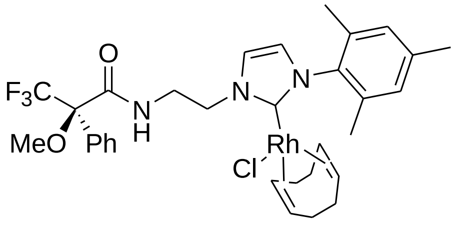

Following general procedure 5 with [(+)-MosherNH(CH_2)$_2$(Im-Mes)]$^+$, Cl^- (0.031 g, 0.062 mmol) in dry acetone (2 mL), potassium carbonate K_2CO_3 (0.018 g, 0.13 mmol), [RhCl(COD)]$_2$ (0.016 g, 0.0325 mmol), and purification by chromatography on silica (eluent petroleum spirit:ethyl acetate 1:1), the product was obtained as a yellow solid (0.031 g, 0.043 mmol, 69 %) as a mixture of diastereomers (ratio 49:51 determined by integration of ^{1}H NMR *e.g.* C*H*$_{imid}$). **^{1}H NMR** (500 MHz, $CDCl_3$) δ 8.01 -7.93 (m, 1H, N*H*$_{dia\ 1\ and\ 2}$), 7.54 (dd, *J* = 6.9, 2.7 Hz, 1H, C*H*$_{Ph\ ortho\ dia1}$), 7.45 (dd, *J* = 6.8, 2.9 Hz, 1H, 0.5 x 2 x C*H*$_{Ph\ adia1}$), 7.41 – 7.33 (m, 2H, C*H*$_{Ph\ para}$, 0.5 x 2 x C*H*$_{Ph\ bdia2}$), 7.30 (dd, *J* = 5.1, 1.9 Hz, 1H, 0.5 x 2 x C*H*$_{Ph\ adia2}$), 7.10 (d, *J* = 1.9 Hz, 0.5H, 0.5 x C*H*$_{Mes\ b\ dia2}$), 7.07 (d, *J* = 2.0 Hz, 0.5H, 0.5 x C*H*$_{Mes\ a\ dia2}$), 7.06 (d, *J* = 1.9 Hz, 0.5H, 0.5 x C*H*$_{imid\ b\ dia1}$), 6.93 (d, *J* = 1.9 Hz, 0.5H, 0.5 x C*H*$_{Mes\ b\ dia1}$), 6.91 (d, *J* = 2.0 Hz, 0.5H, 0.5 x C*H*$_{Mes\ a\ dia1}$) 6.87 (d, *J* = 2.0 Hz, 0.5H, 0.5 x C*H*$_{imid\ b\ dia2}$), 6.77 (d, *J* = 1.9 Hz, 1H, 0.5 x C*H*$_{imid\ a\ dia1}$), 6.63 (d, *J* = 1.9 Hz, 0.5H, 0.5 x C*H*$_{imid\ a\ dia2}$), 5.48 – 5.42 (m, 0.5H, 0.25 x C*H*$_{2b}$-Imid$_{dia1}$), 5.42 – 5.36 (m, 0.5H, 0.25 x C*H*$_{2a}$-Imid$_{dia2}$), 4.86 – 4.76 (m, 2H, C*H*=CH $_{COD\ dia}$), 4.60 (dt, *J* = 13.8, 5.5 Hz, 0.5H, 0.25 x C*H*$_{2a}$-Imid $_{dia1}$), 4.54 (ddd, *J* = 13.7, 6.0, 4.7 Hz, 0.5H, 0.25 x C*H*$_{2b}$ -Imid $_{dia2}$), 4.33 – 4.20 (m, 1H, 0.5 x C*H*$_2$NH $_{dia}$), 4.03 – 3.84 (m, 1H, 0.5 x C*H*$_2$NH $_{dia}$), 3.51 -3.44 (m, 1H, C*H*=CH$_{COD\ dia}$), 3.40 (s, 1.5H, 0.5 x OC*H*$_{3\ dia}$), 3.29 (s, 1.5H, 0.5 x OC*H*$_{3\ dia}$), 3.02 – 2.96 (m, 0.5H, 0.5 x C*H*=CH$_{COD\ dia}$), 2.95 – 2.90 (m, 0.5H, 0.5 x C*H*=CH$_{COD\ dia}$), 2.41 (s, 1.5H, 0.5 x C*H*$_{3Mes\ dia}$), 2.40 – 2.37 (m, 1H, 0.5 x C*H*$_{2aCOD\ dia}$), 2.38 (s, 1.5H, 0.5 x C*H*$_{3Mes\ dia}$), 2.37 (s, 1.5H, 0.5 x C*H*$_{3Mes\ dia}$), 2.27 (s, 1.5H, 0.5 x C*H*$_{3Mes\ dia}$), 2.15 – 2.09 (m, 1H, 0.5 x C*H*$_{2dCOD\ dia}$), 2.02 – 1.96 (m, 1H, 0.5 x C*H*$_{2c\ COD\ dia}$), 1.93 – 1.89 (m, 1H, 0.5 x C*H*$_{2aCOD}$), 1.83 (s, 1.5H, 0.5 x C*H*$_{3Mes\ dia}$), 1.80 – 1.76 (m, 1H, 0.5 x C*H*$_{2cCOD\ dia}$), 1.79 (s, 1.5H, 0.5 x C*H*$_{3Mes\ dia}$), 1.68 – 1.63 (m, 1H, 0.5 x C*H*$_{2COD\ dia}$), 1.51 – 1.42 (m, 2H, C*H*$_{2bCOD}$). **^{13}C NMR** (126 MHz, $CDCl_3$) *δ* 183.1 (d, *J* = 51.4 Hz, *C*=Rh $_{dia1}$), 182.7 (d, *J* = 51.4 Hz, *C*=Rh$_{dia2}$), 167.7 (*C*=O), 138.9 (*C*$_{Mes\ dia1}$), 138.9 (*C*$_{Mes\ dia2}$), 137.2 (*C*$_{Mes\ dia1}$), 137.1 (*C*$_{Mes\ dia2}$), 136.2 (*C*$_{Mes\ dia1}$), 136.1 (*C*$_{Mes\ dia2}$), 134.3 (*C*$_{Mes}$$CH_{3para}$), 132.8 (*C*$_{Ph\ dia1}$), 132.5 (*C*$_{Ph\ dia2}$), 129.8 (*C*$H_{Mes\ dia1}$), 129.7 (*C*$H_{Mes\ dia2}$), 129.5 (2 x *C*$H_{Ph\ dia1}$), 129.4 (2 x *C*$H_{Ph\ dia2}$), 128.7 (*C*$H_{Ph}$), 128.4 (*C*$H_{Mes\ dia1}$), 128.4 (*C*$H_{Mes\ dia2}$), 127.8 (2 x *C*$H_{Ph\ dia1}$), 127.7 (2 x *C*$H_{Ph\ dia2}$), 124.4 (q, *J* = 289.4 Hz, *C*$F_{3\ dia1}$), 124.4 (q, *J* = 289.4 Hz, *C*$F_{3\ dia2}$), 123.7 (*C*$H_{imid\ dia2}$), 123.6 (*C*$H_{imid\ dia1}$), 120.8 (*C*$H_{imid\ dia1}$), 120.3 (*C*$H_{imid\ dia2}$), 97.8 (d, *J* = 7.0 Hz, *C*H_{CODdia}), 97.8 (d, *J* = 7.0 Hz, *C*H_{CODdia}), 97.3 (d, *J* = 7.2 Hz, *C*H_{CODdia}), 69.0 (d, *J* = 14.1 Hz, *C*$H_{COD\ dia1}$), 69.0 (d, *J* = 14.1 Hz, *C*$H_{COD\ dia2}$), 67.6 (d, *J* = 14.1 Hz, *C*$H_{COD\ dia1}$), 67.6 (d, *J* = 14.1 Hz, *C*$H_{COD\ dia1}$), 55.1 (O*C*$H_{3\ dia1}$), 54.9 (O*C*$H_{3\ dia2}$), 49.1 (*C*H_2Imid $_{dia1}$), 49.0 (*C*H_2Imid $_{dia2}$), 39.1 (*C*H_2NH_{dia2}), 38.8 (*C*H_2NH_{dia2}), 34.7 (*C*$H_{2COD\ dia1}$), 34.6 (*C*$H_{2COD\ dia2}$), 31.3 (*C*$H_{2COD\ dia1}$), 31.2 (*C*$H_{2COD\ dia2}$), 28.2 (*C*$H_{2COD\ dia1\ and\ 2}$), 27.9 (*C*$H_{2COD\ dia1}$), 27.8 (*C*$H_{2COD\ dia2}$), 21.2 (*C*$H_{3Mes\ dia1\ and\ 2}$), 19.8 (*C*$H_{3Mes\ dia1}$), 19.7 (*C*$H_{3Mes\ dia2}$), 18.0 (*C*$H_{3Mes\ dia1}$), 17.9 (*C*$H_{3Mes\ dia2}$). **FTIR (neat)** ν_{max} = 3296, 2928, 1652, 1530, 1260, 1071, 1027, 733, 695 cm^{-1}. **HR – MS** (ESI, positive ion mode) – m/z for $[C_{32}H_{38}N_3F_3O_2Rh]^+$ 656.1966, found 656.1952. **MP** 135-140 °C.

4. ^{1}H and ^{13}C NMR spectra of synthesized compounds

4.1 Cbz-(L-αMeVal)-Aib$_5$O^{t}Bu

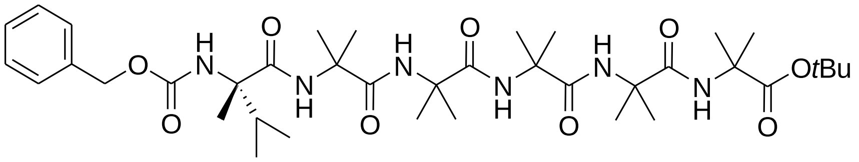

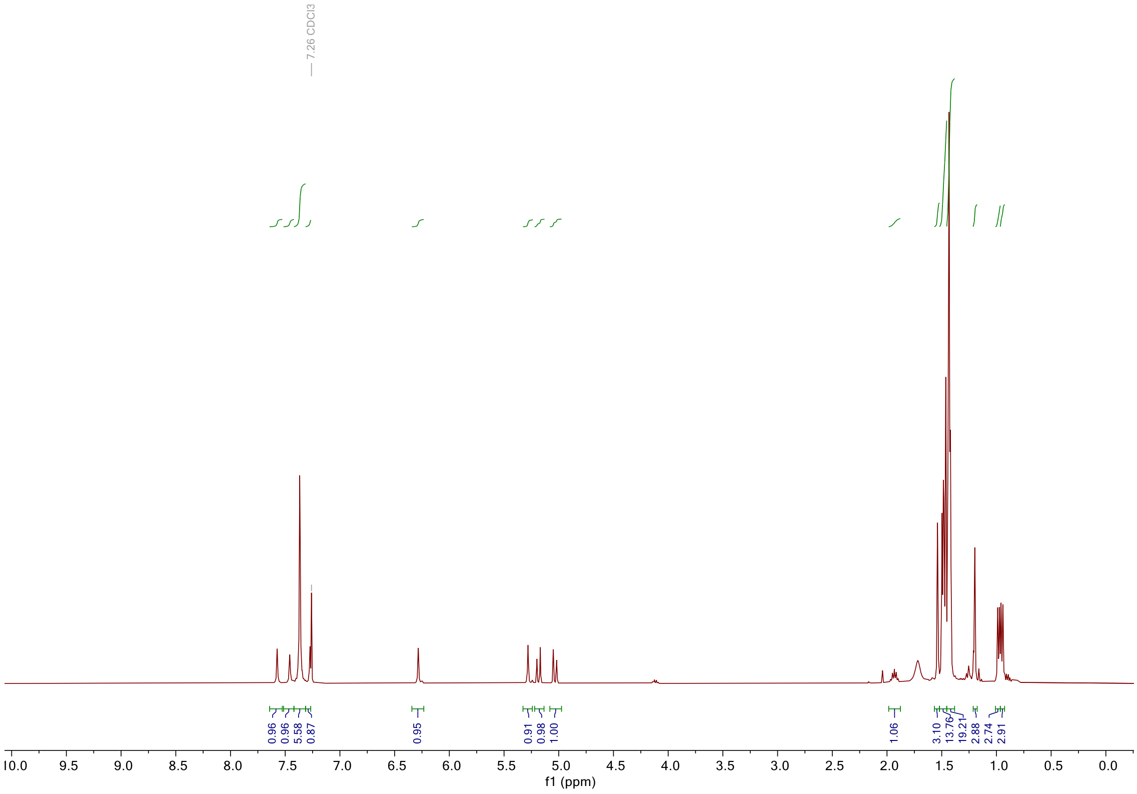

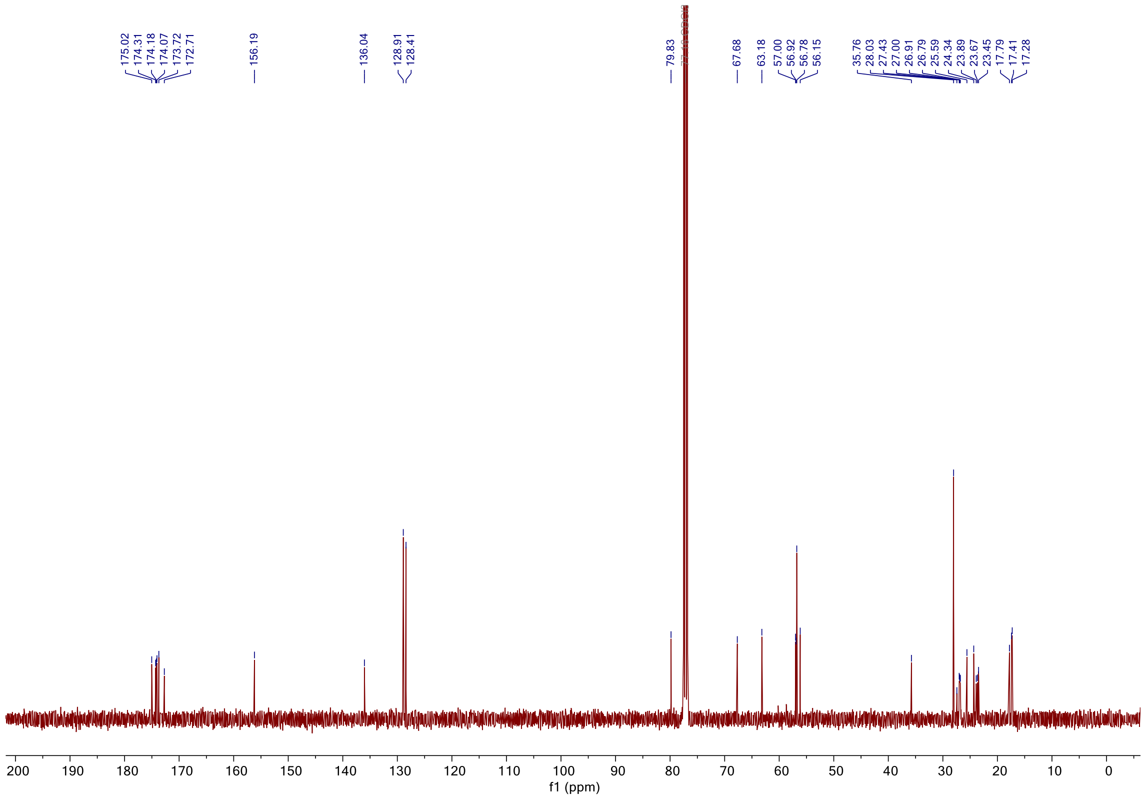

^{1}H ^{13}C HSQC

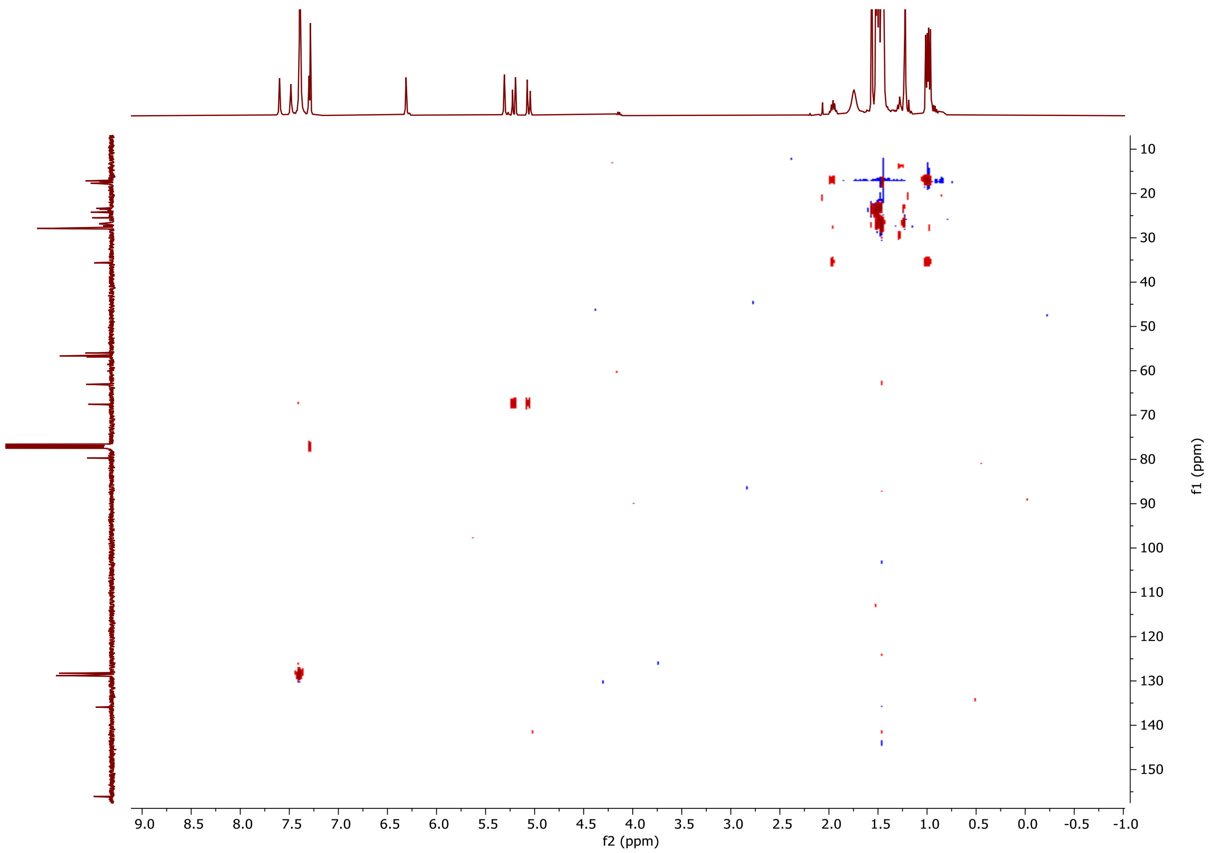

^{1}H ^{13}C HMBC

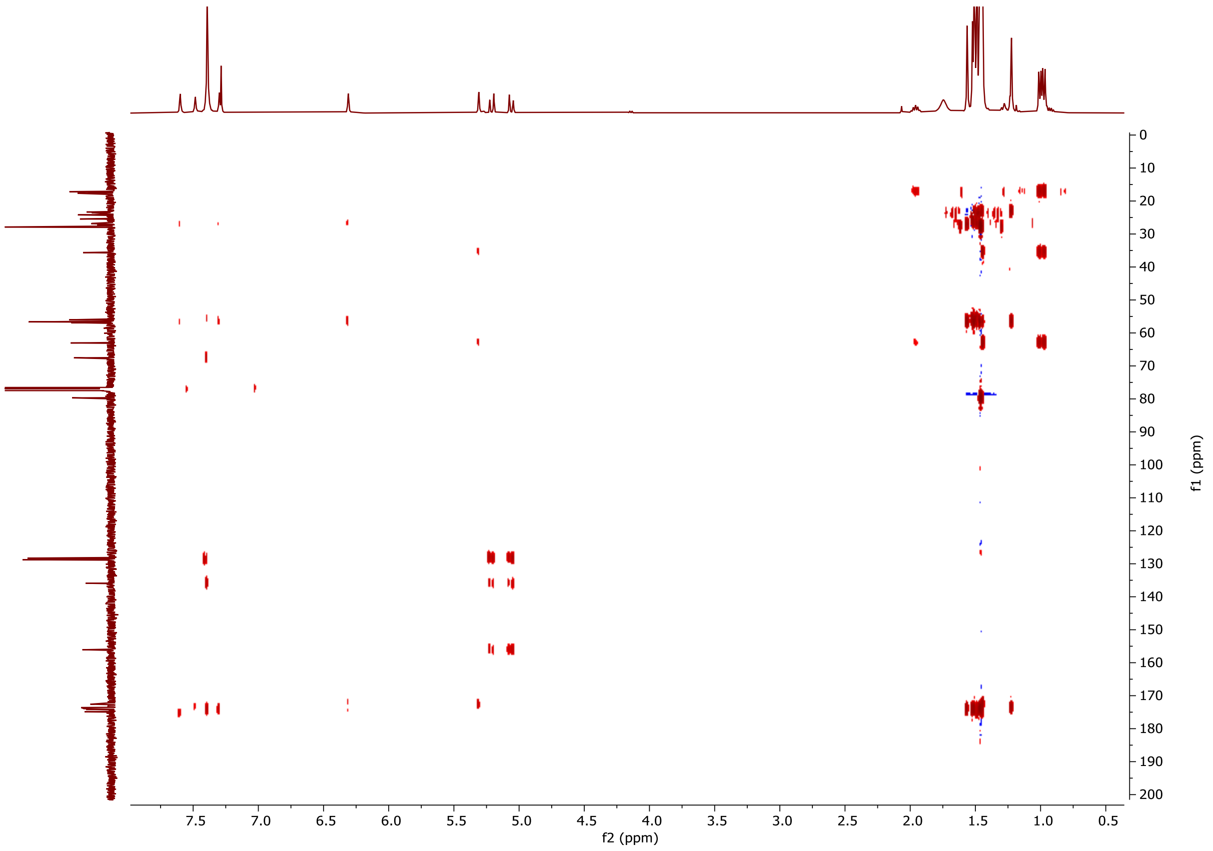

4.2 *Cbz-(L-αMeVal)-Aib$_5$OH*

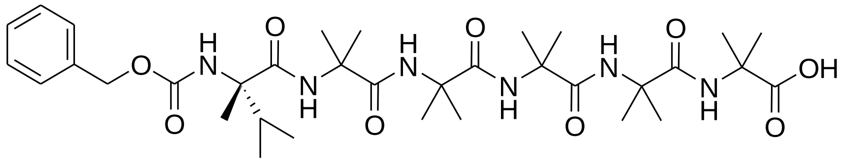

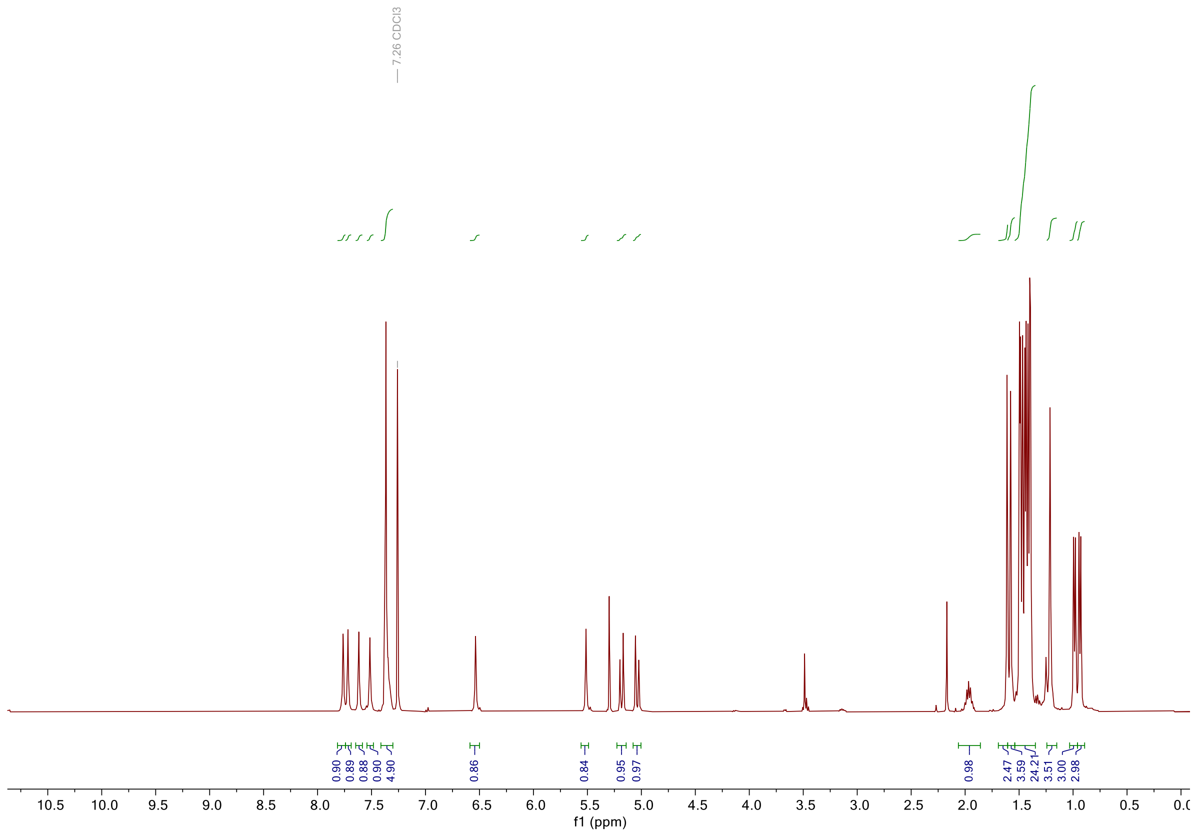

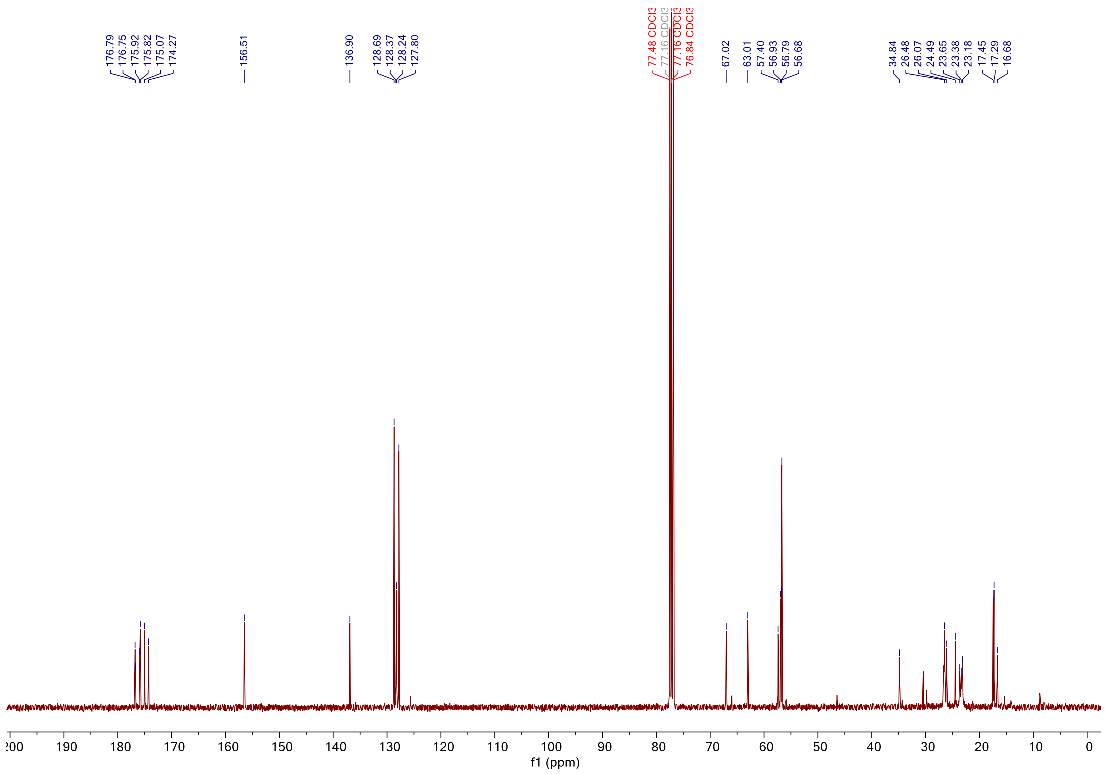

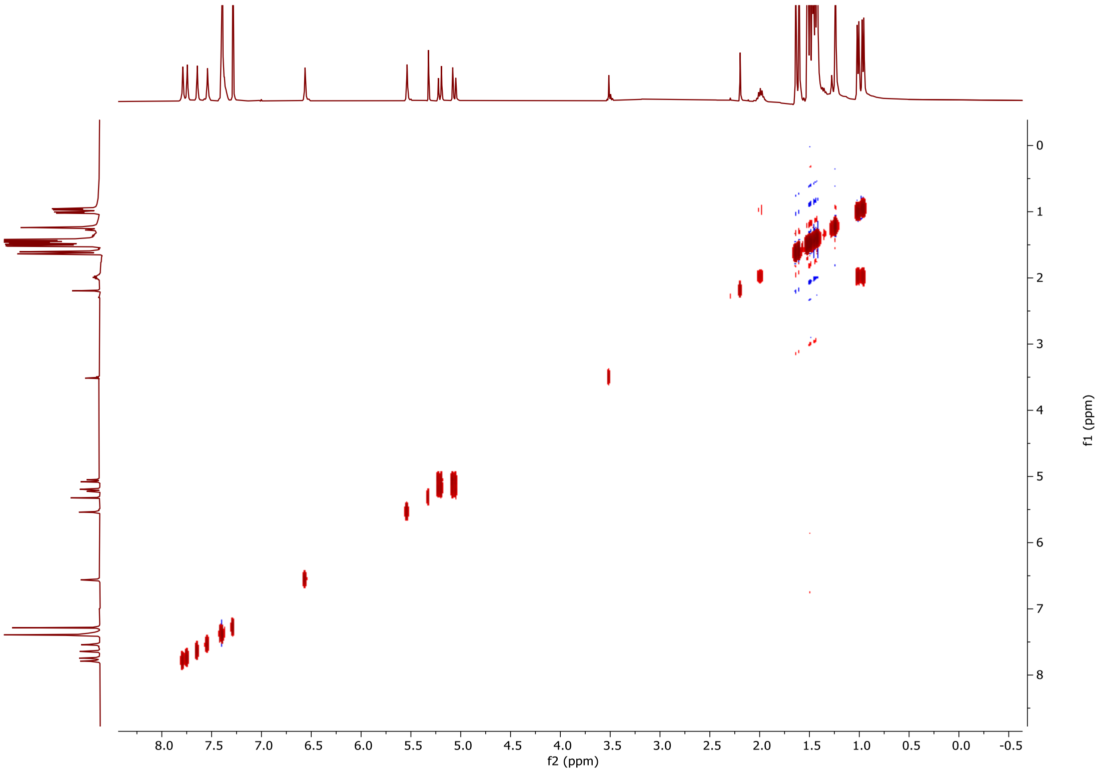

^{1}H ^{13}C HSQC

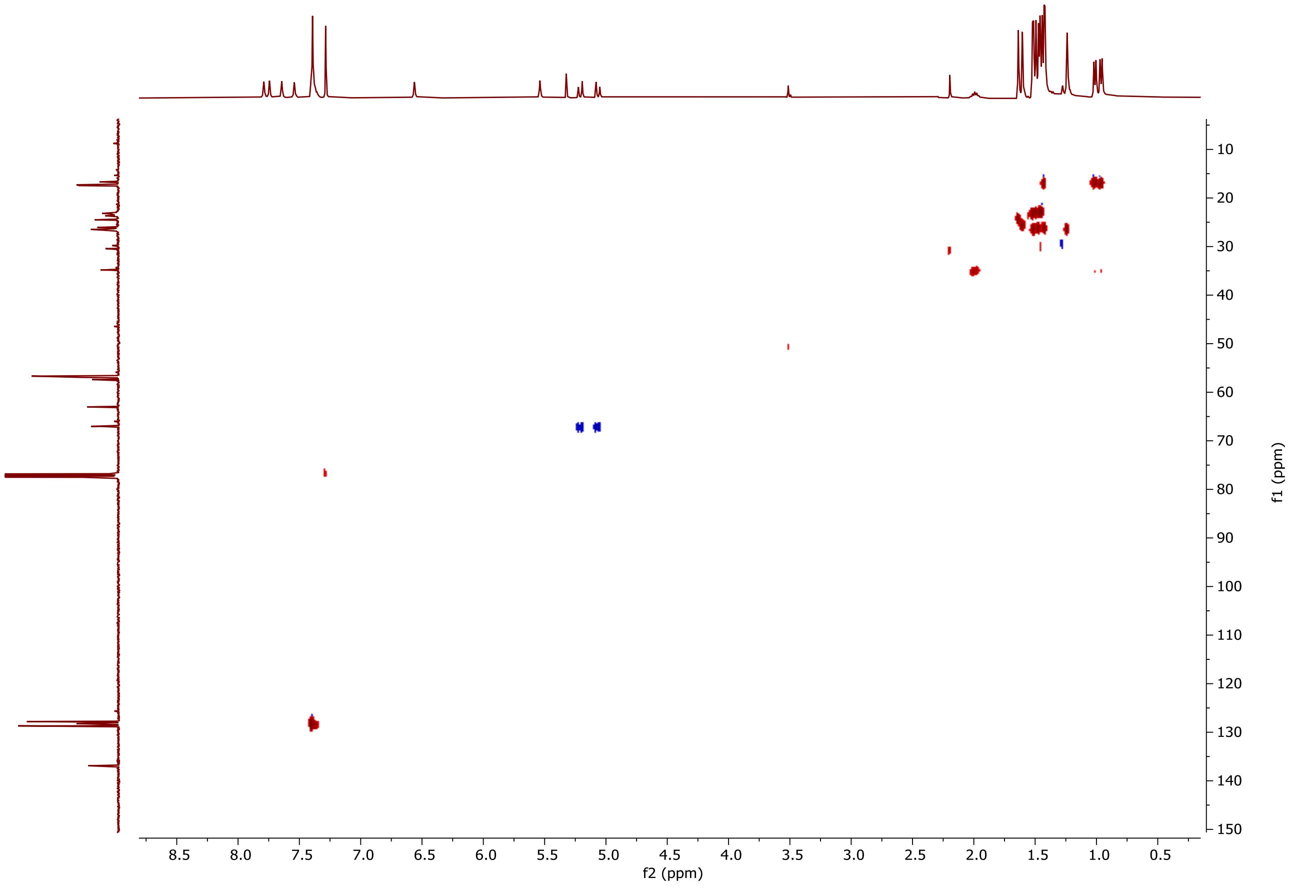

^{1}H ^{13}C HMBC

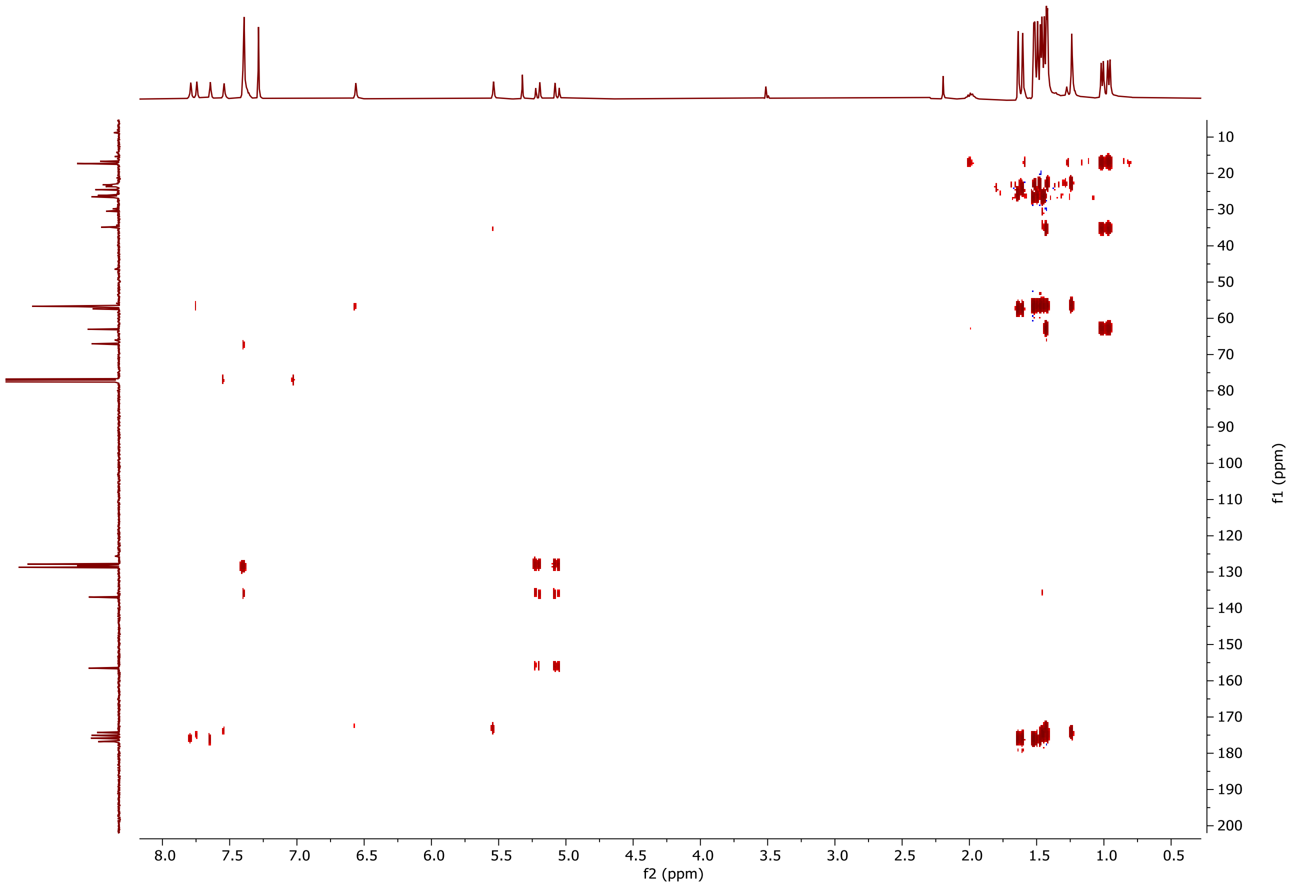

f2 (ppm)
f1 (ppm)
8.0
7.5
7.0
6.5
6.0
5.5
5.0
4.5
4.0
3.5
3.0
2.5
2.0
1.5
1.0
0.5
10
20
30
40
50
60
70
80
90
100
110
120
130
140
150
160
170
180
190
200

4.3 1-(2-Aminoethyl)-3-(phenyl)-1H-imidazol-3-ium bromide, hydrobromide salt 5

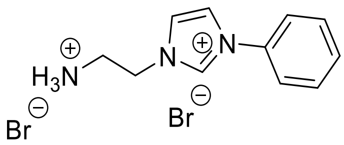

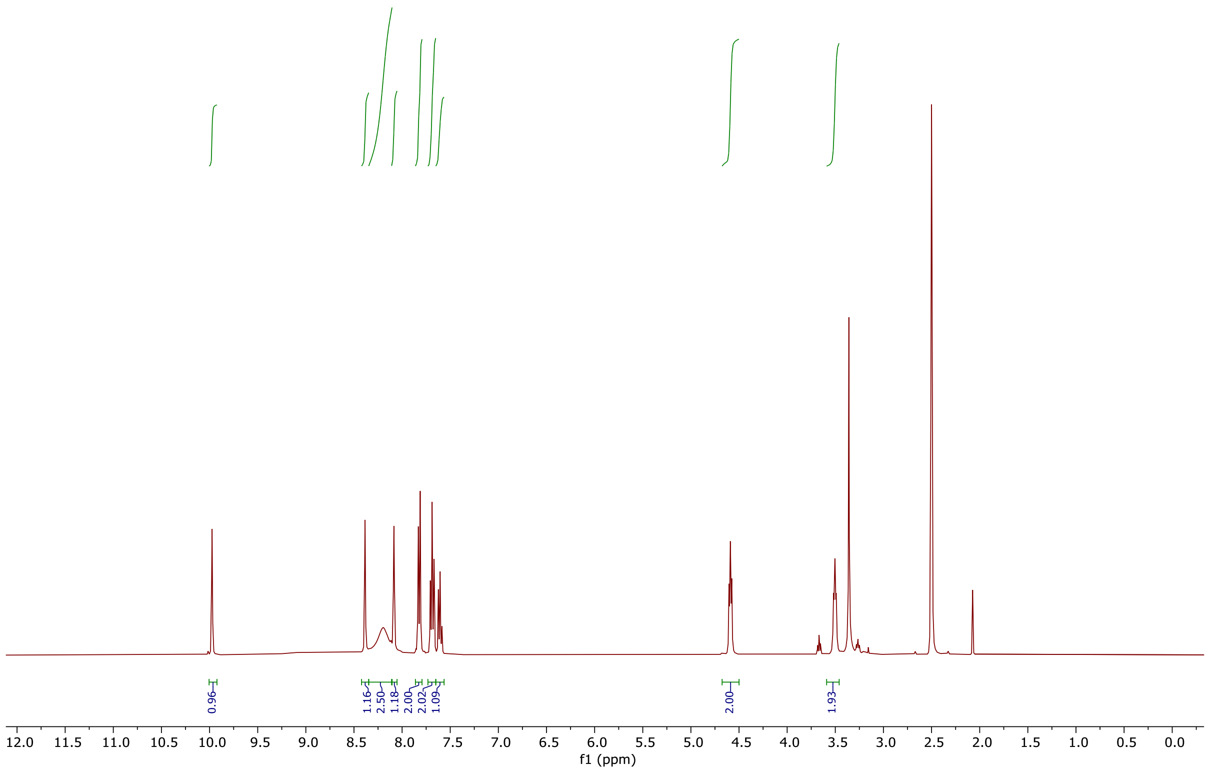

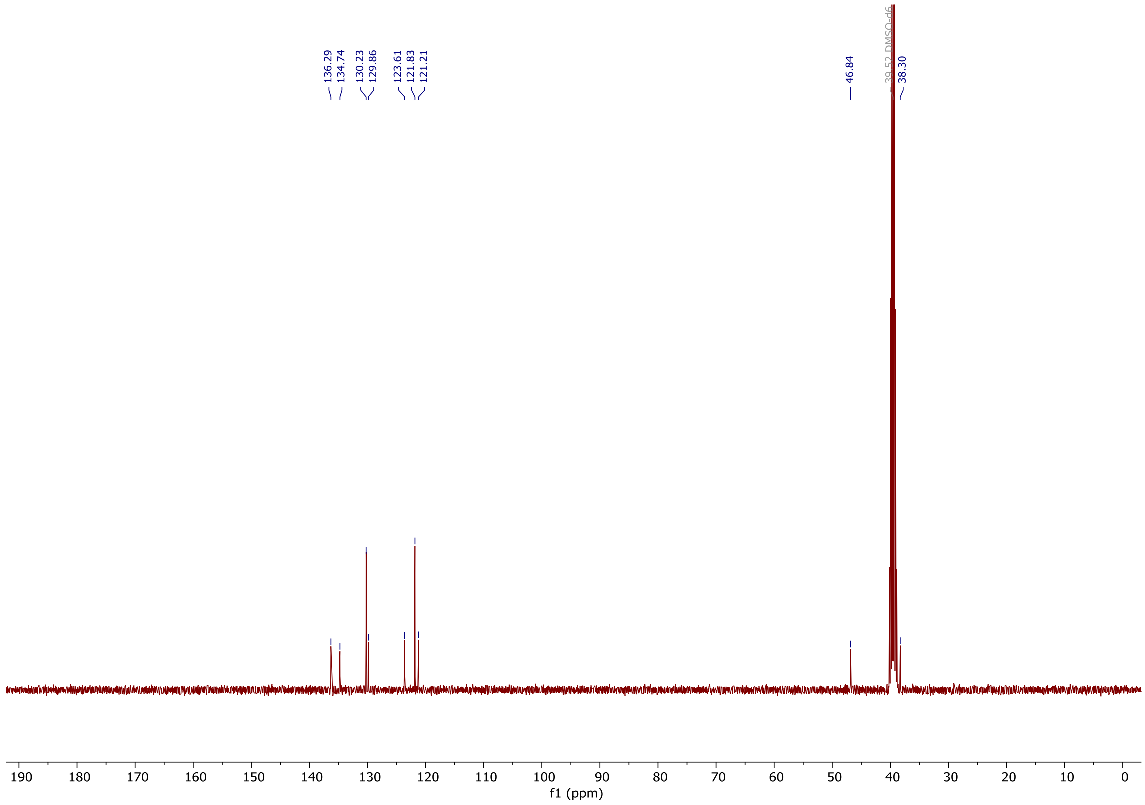

4.4 1-(2-Aminoethyl)-3-(2,4,6-methylphenyl)-1H-imidazol-3-ium bromide, HBr salt 6

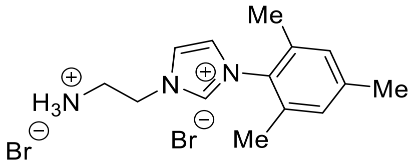

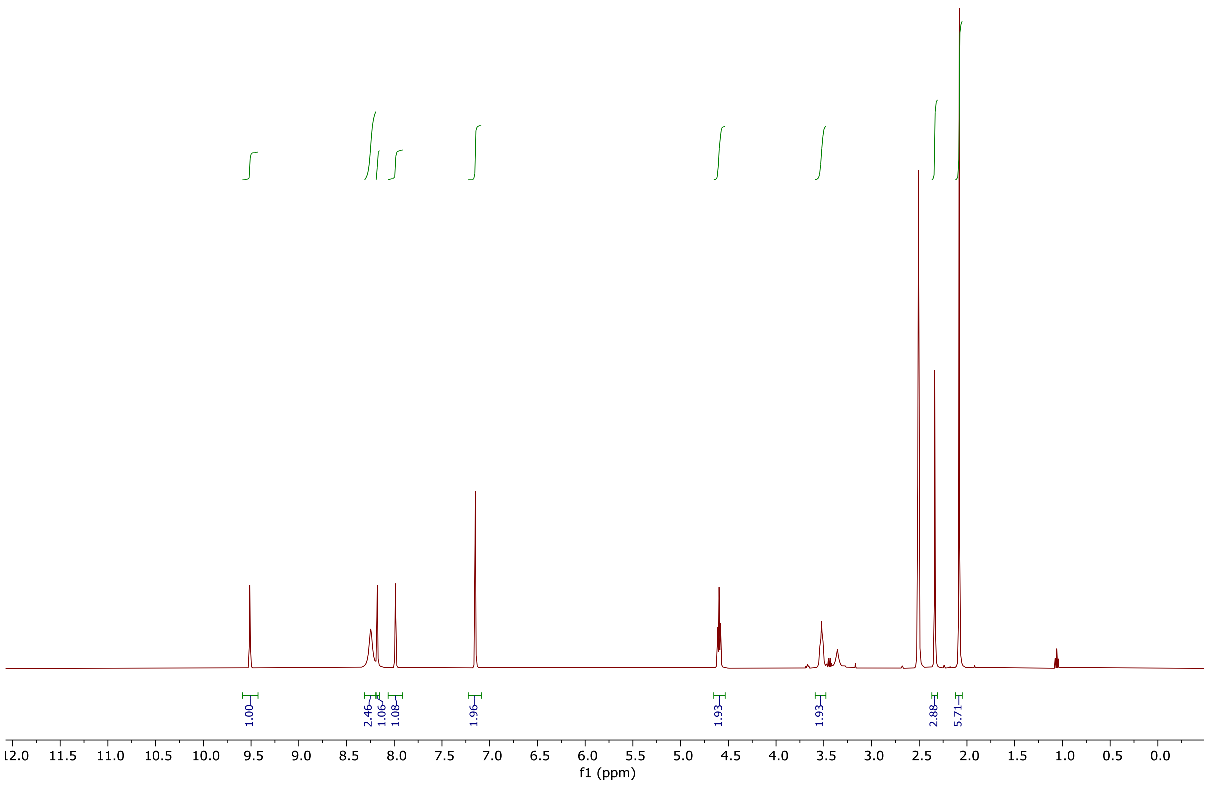

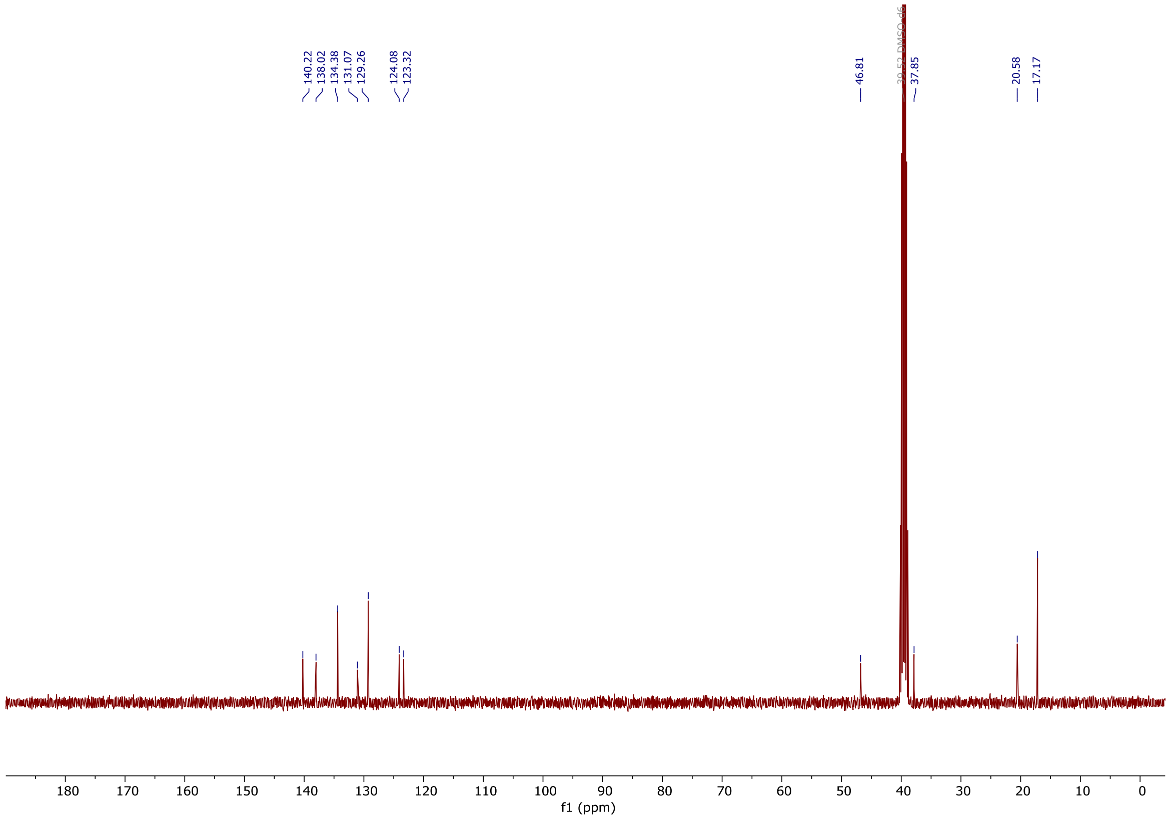

4.5 Precursor S1 1-(2-isobutyramidoethyl)-3-phenyl-1H-imidazol-3-ium bromide

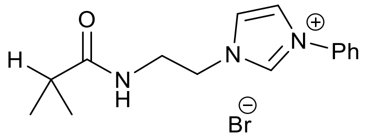

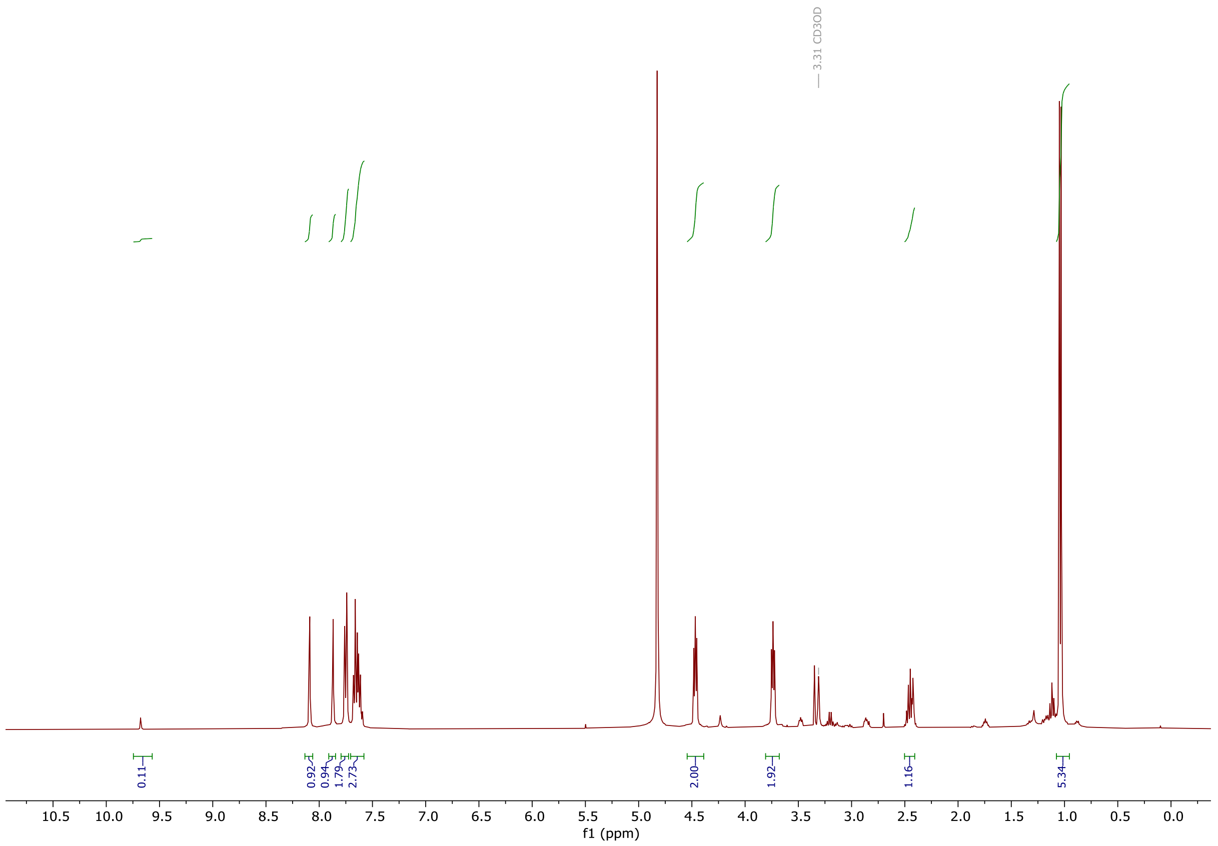

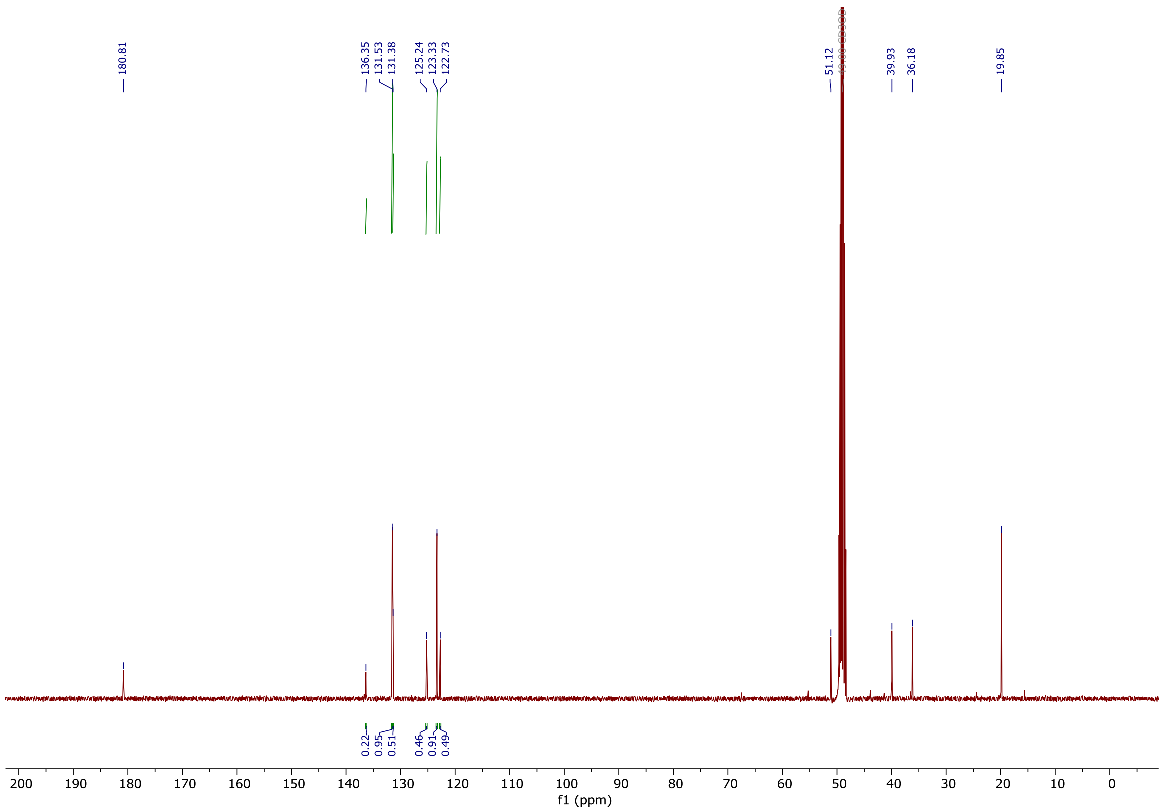

^{1}H ^{13}C HMBC

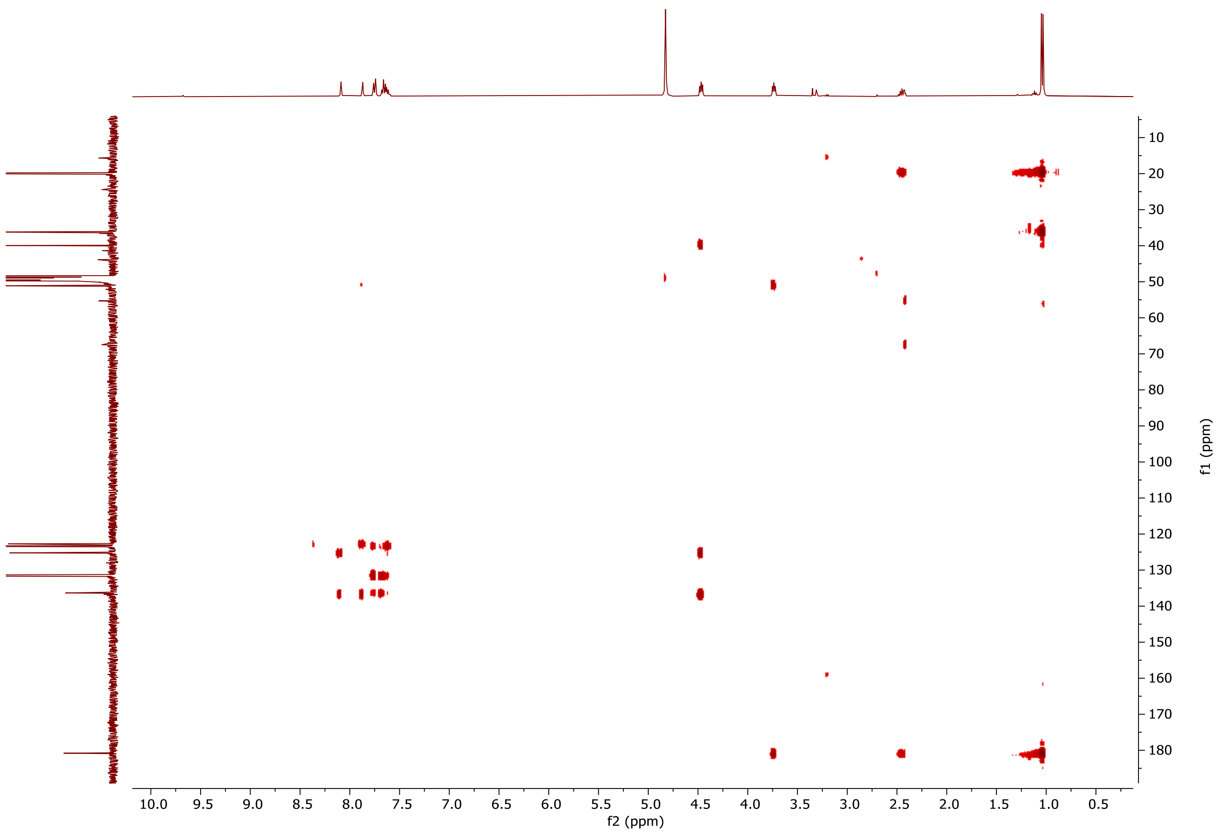

10.0 9.5 9.0 8.5 8.0 7.5 7.0 6.5 6.0 5.5 5.0 4.5 4.0 3.5 3.0 2.5 2.0 1.5 1.0 0.5
f2 (ppm)
10 20 30 40 50 60 70 80 90 100 110 120 130 140 150 160 170 180
f1 (ppm)

4.6 Compound 1

Bromide salt:

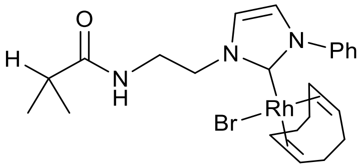

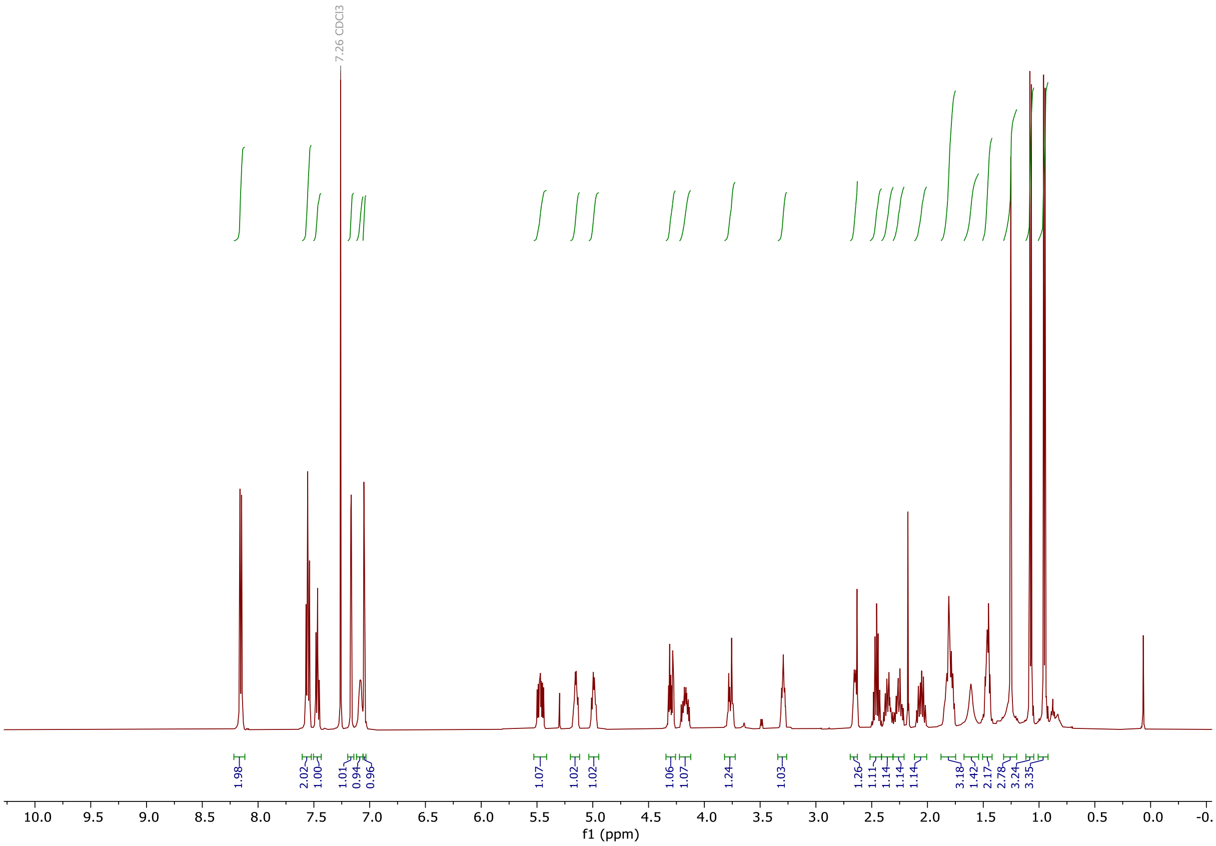

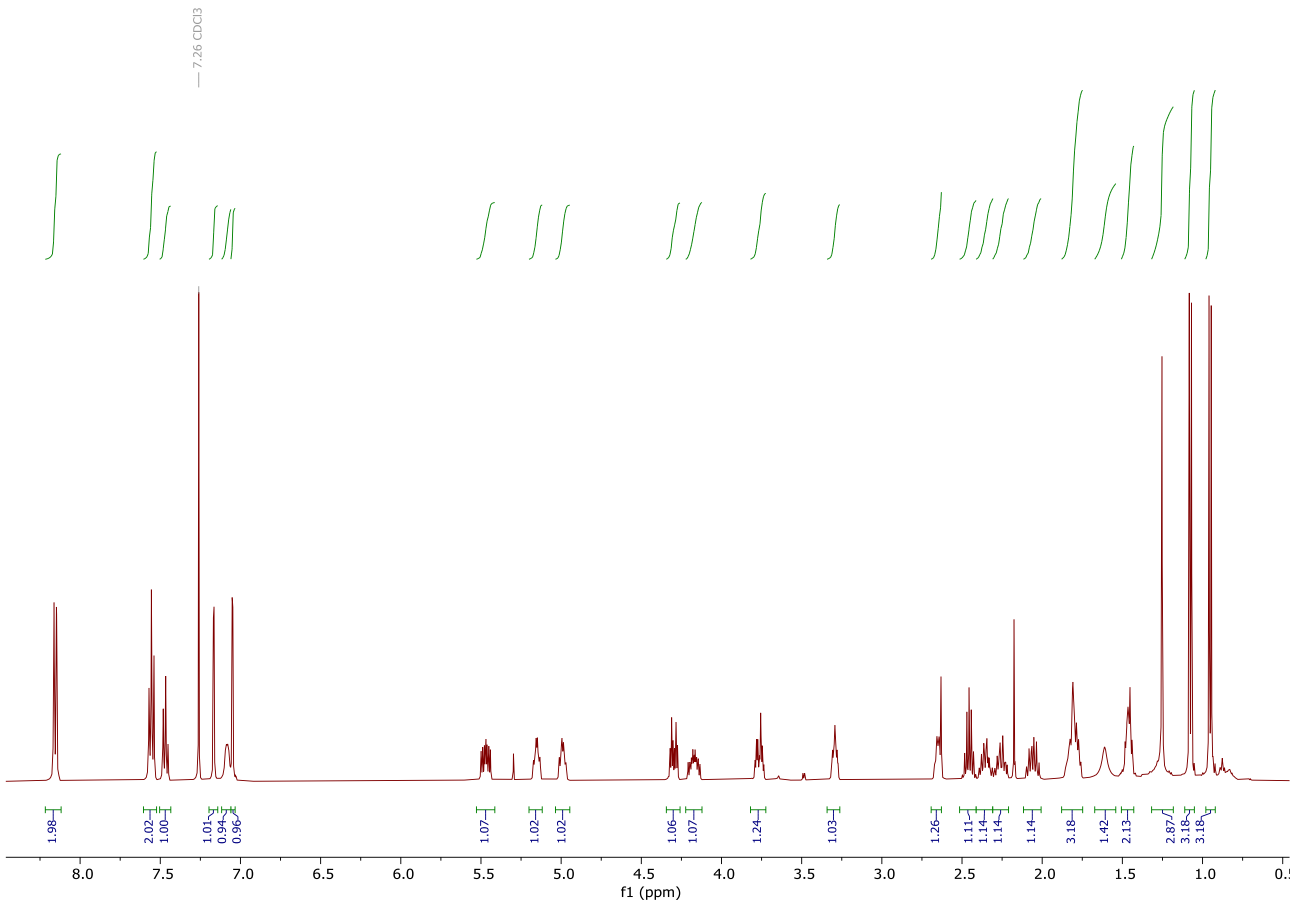
7.26 CDCl3
1.98
2.02
1.00
1.01
0.94
0.96
1.07
1.02
1.02
1.06
1.07
1.24
1.03
1.26
1.11
1.14
1.14
1.14
3.18
1.42
2.13
2.87
3.18
3.18
f1 (ppm)

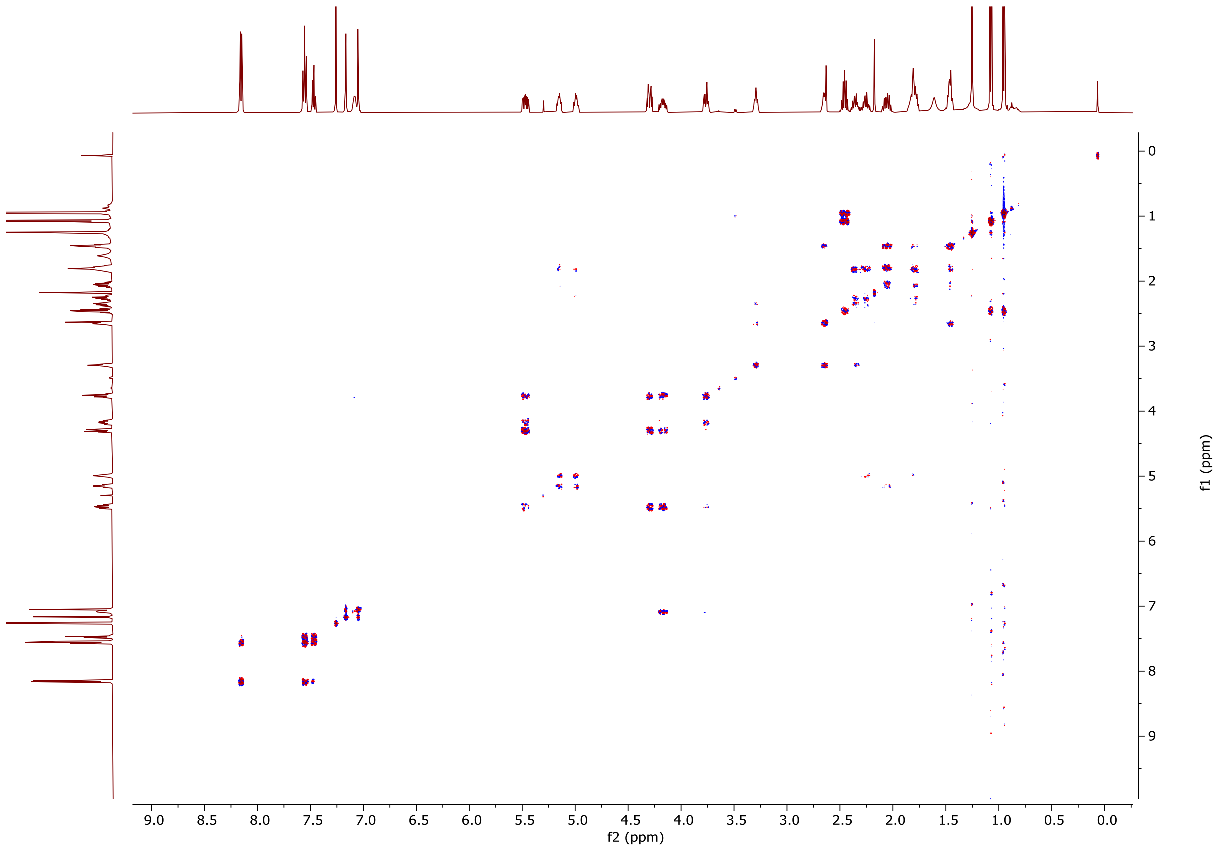
f2 (ppm)
f1 (ppm)

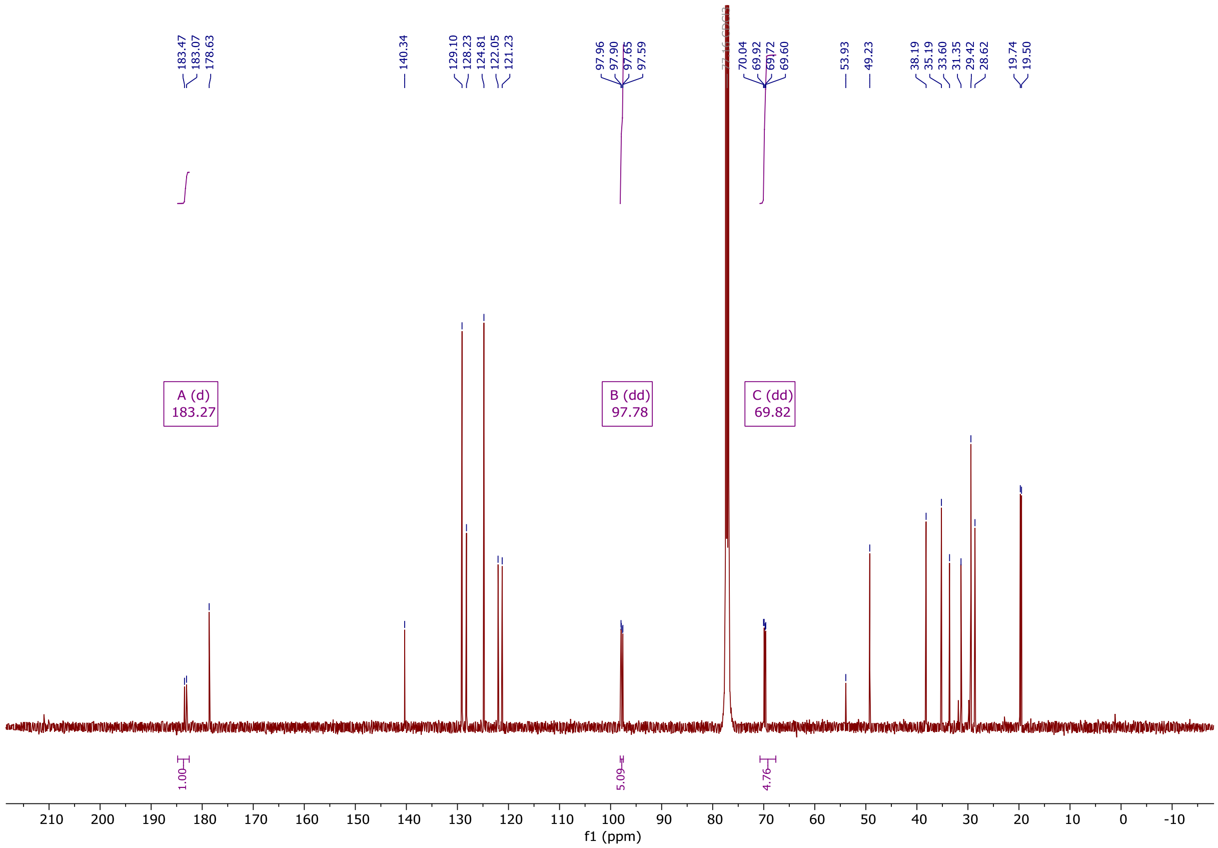

^{1}H ^{13}C HSQC

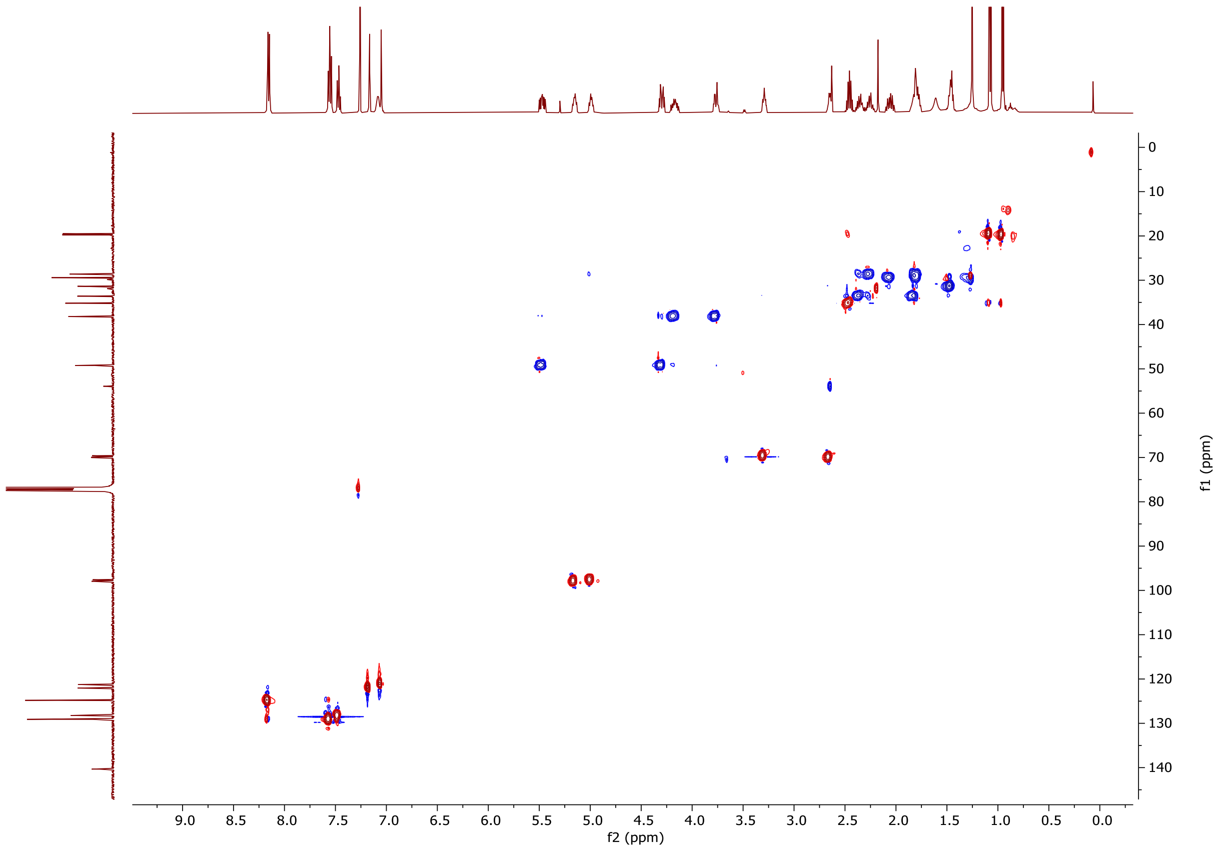

^{1}H ^{13}C HMBC

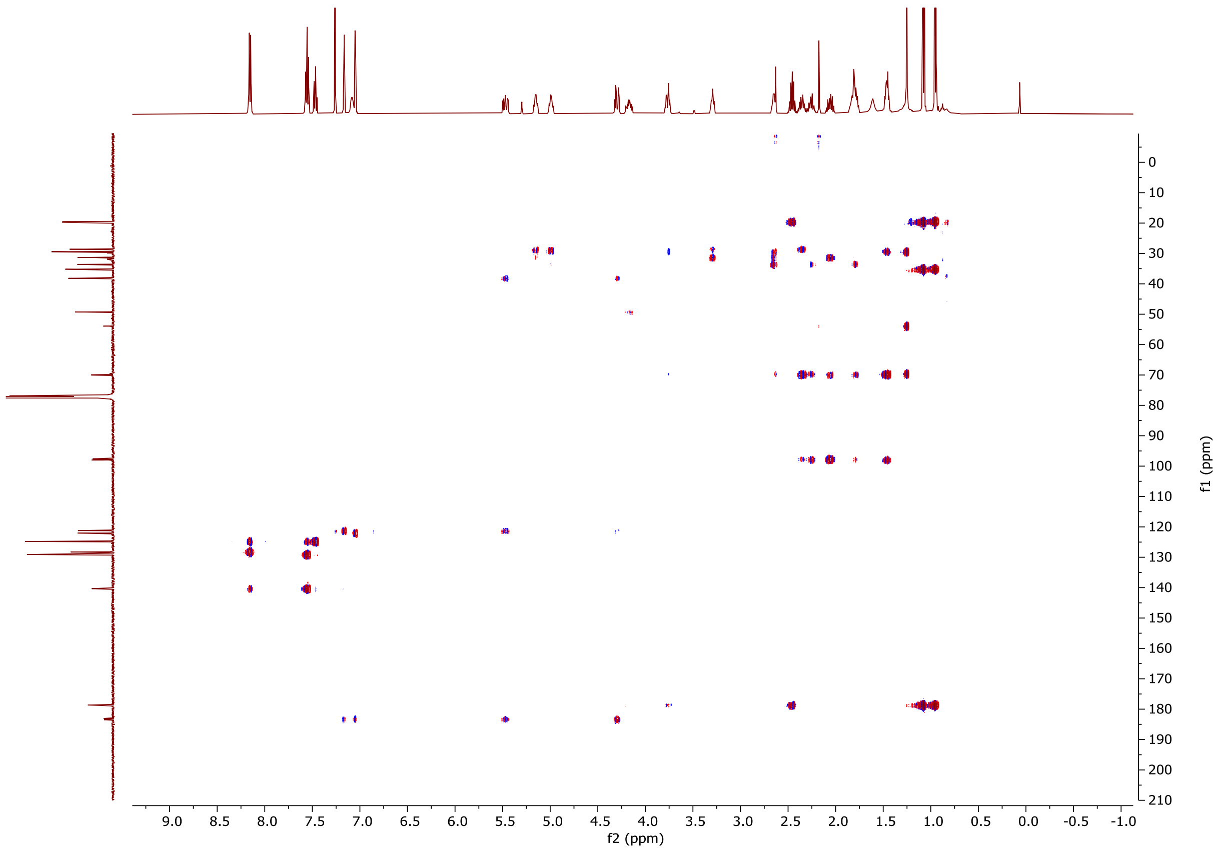

Chloride salt:

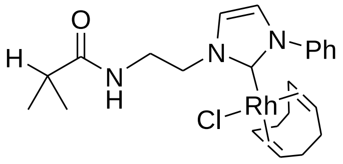

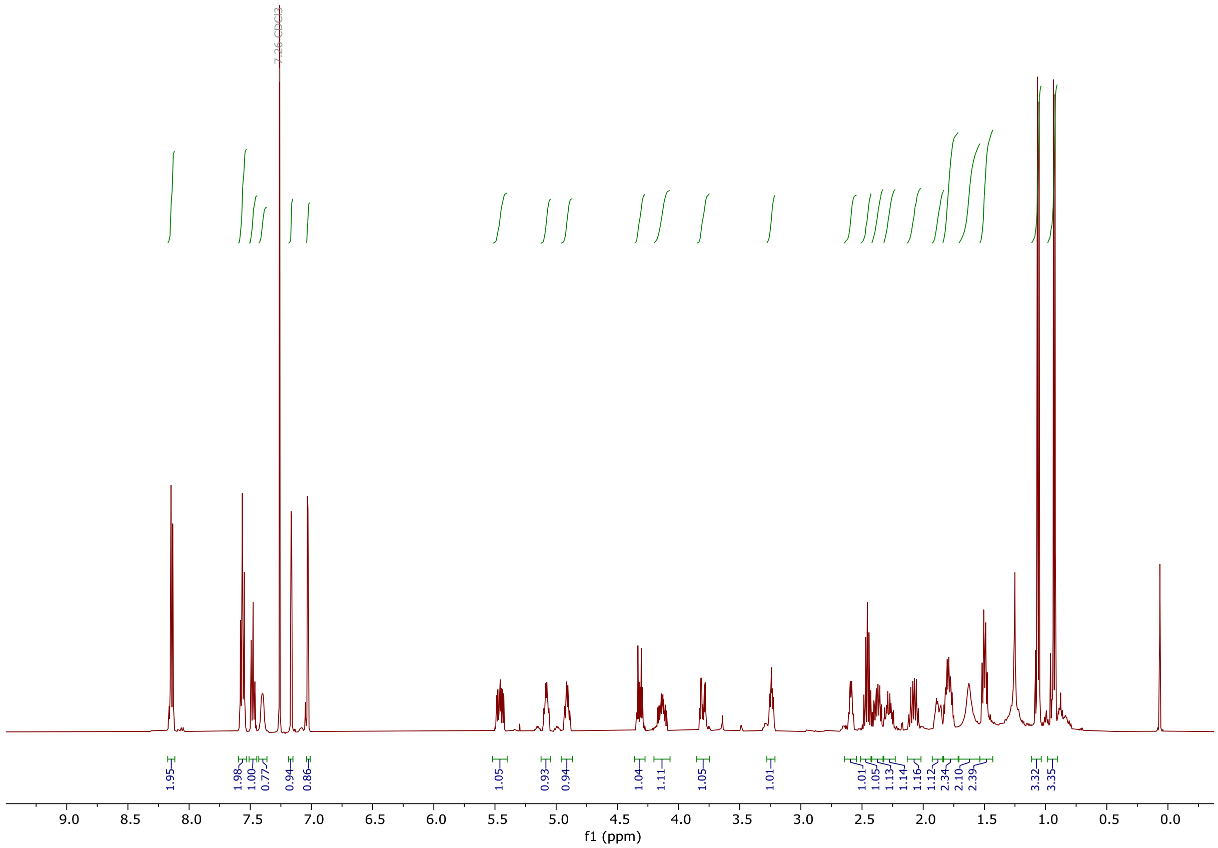

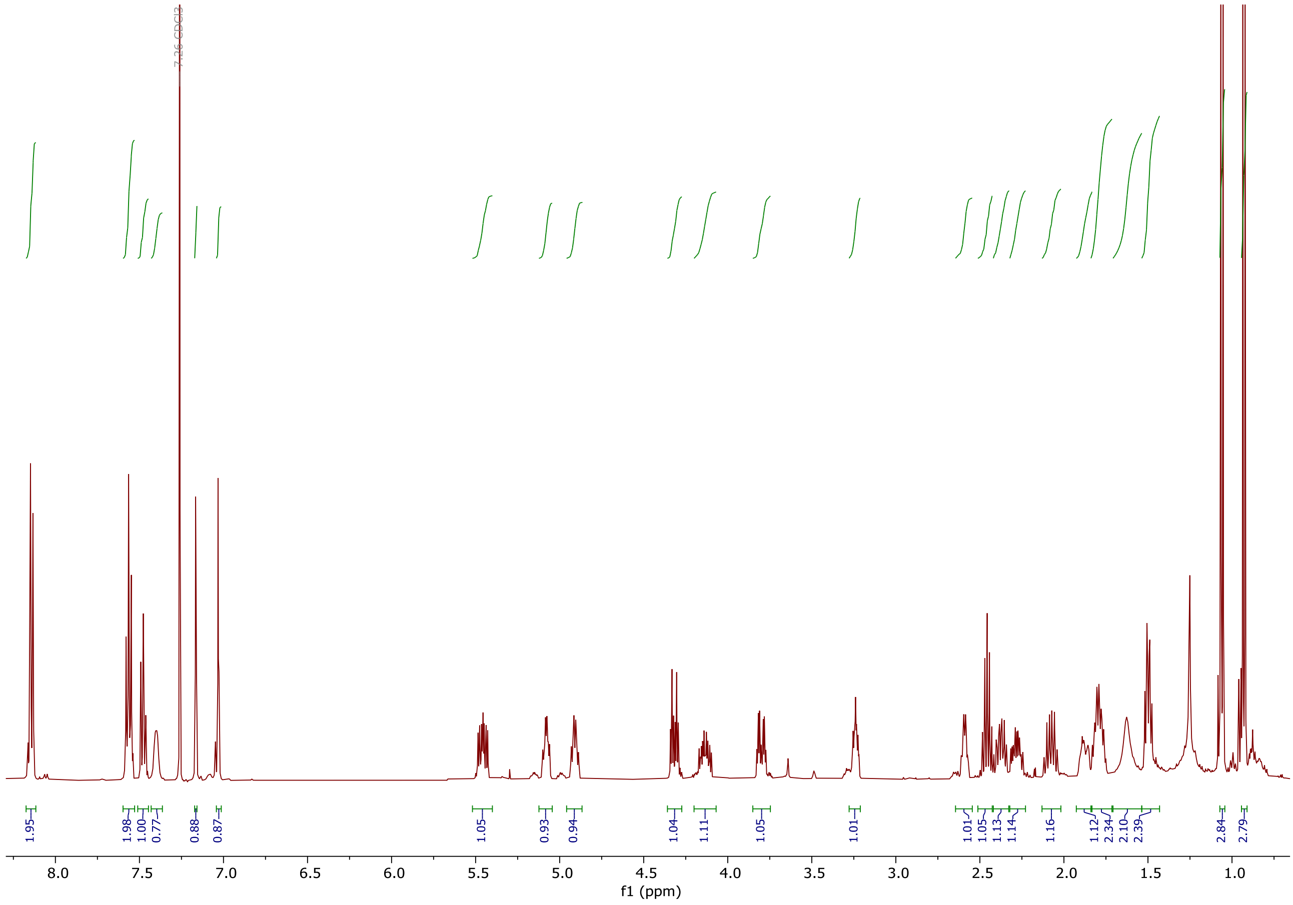

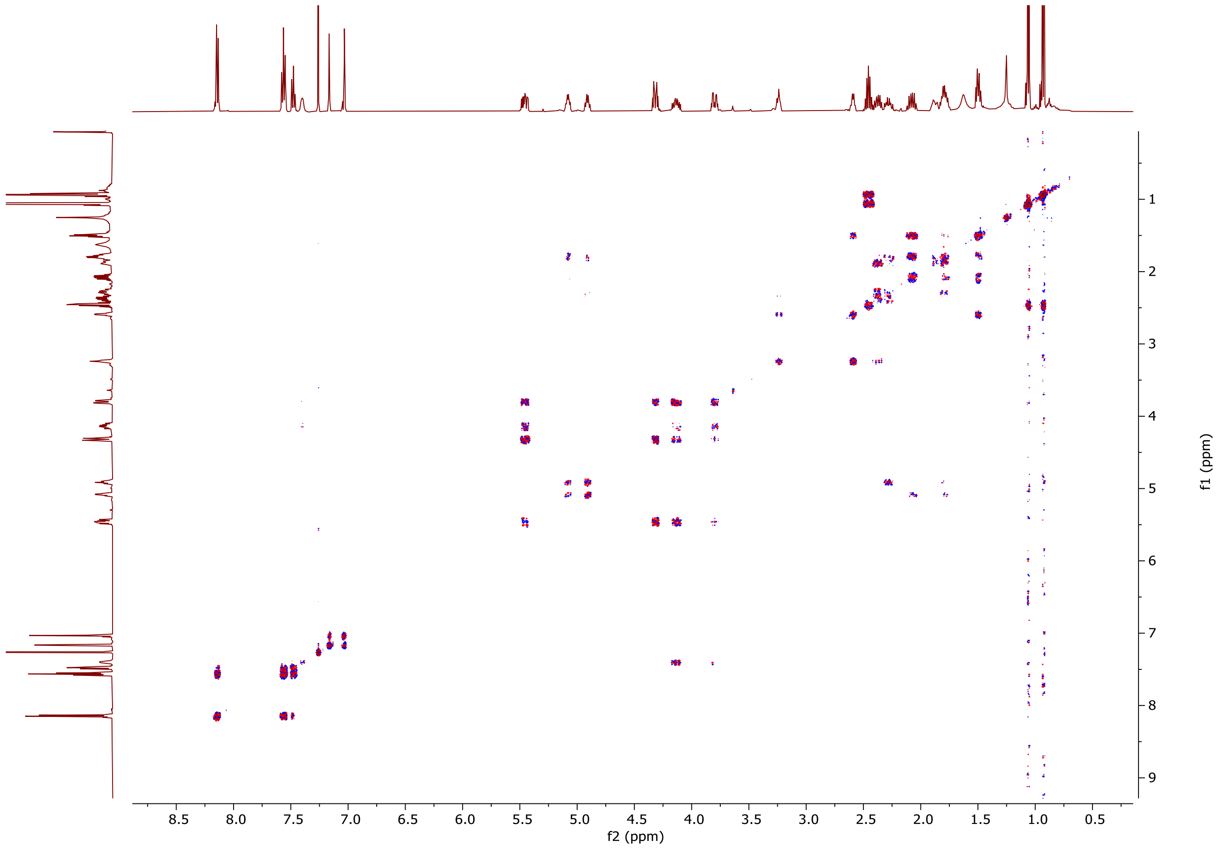
f2 (ppm)
f1 (ppm)

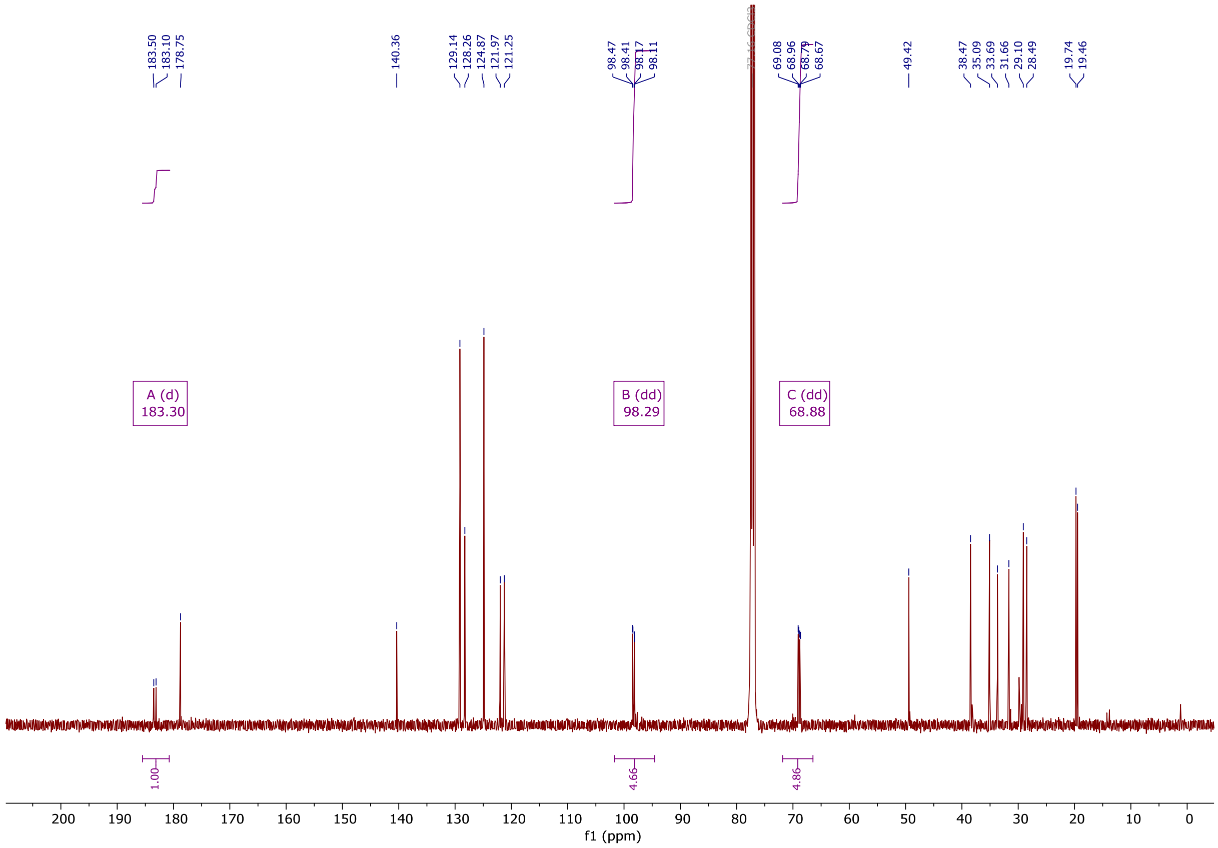
183.50
183.10
178.75
140.36
129.14
128.26
124.87
121.97
121.25
98.47
98.41
98.17
98.11
69.08
68.96
68.79
68.67
49.42
38.47
35.09
33.69
31.66
29.10
28.49
19.74
19.46
A (d)
183.30
B (dd)
98.29
C (dd)
68.88
1.00
4.66
4.86
f1 (ppm)

^{1}H ^{13}C HSQC

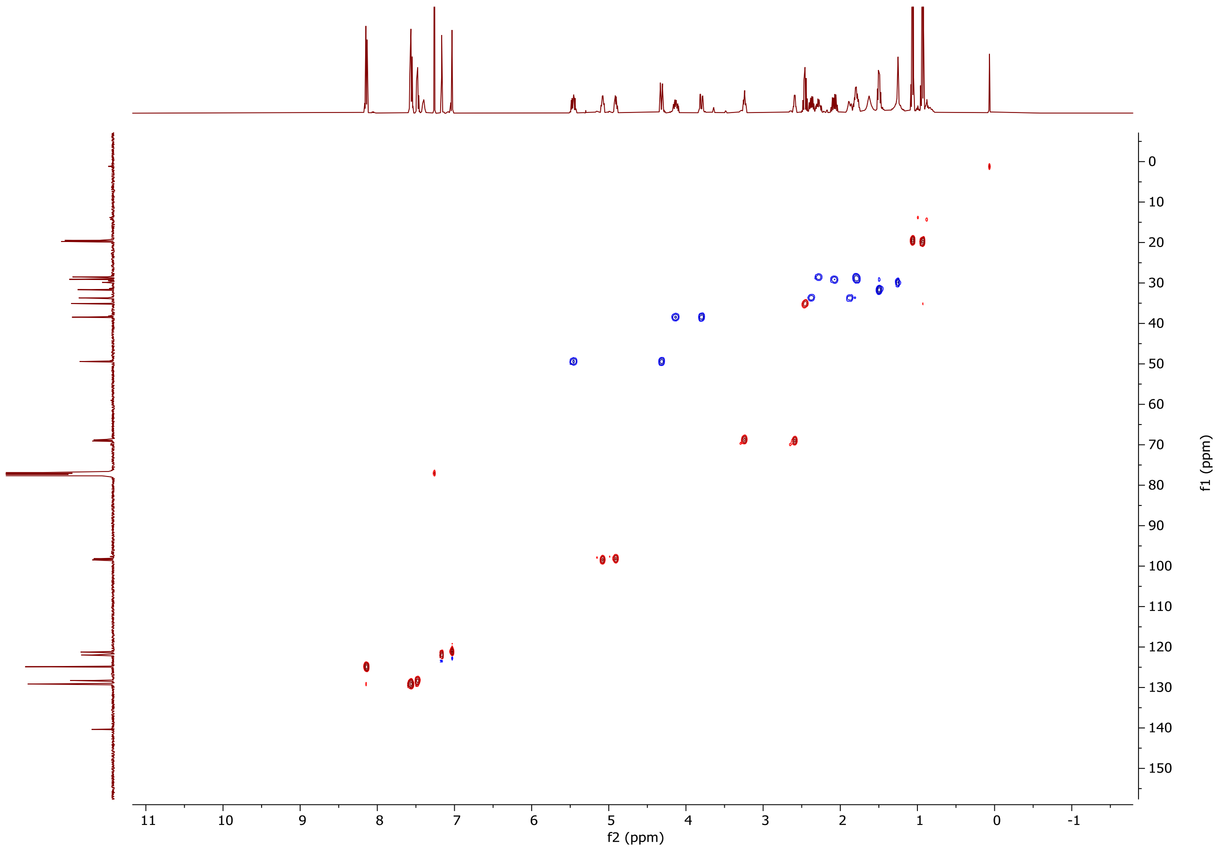

^{1}H ^{13}C HMBC

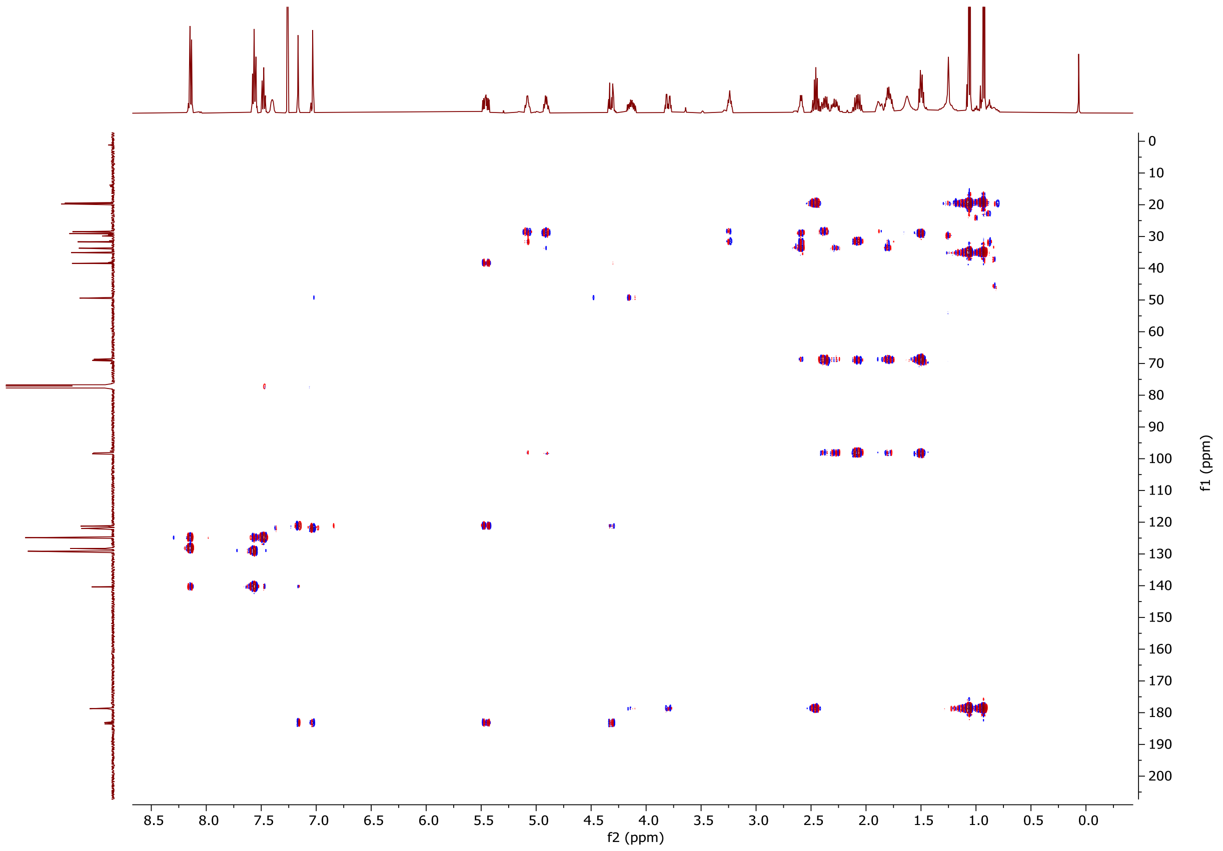

4.7 Precursor S2 [$N_3Aib_4(CH_2)_2$(Im-Ph)]$^+$Br$^-$

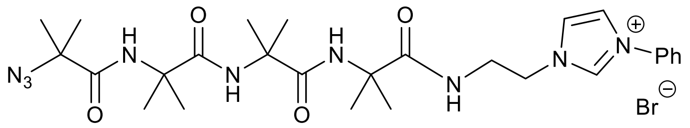

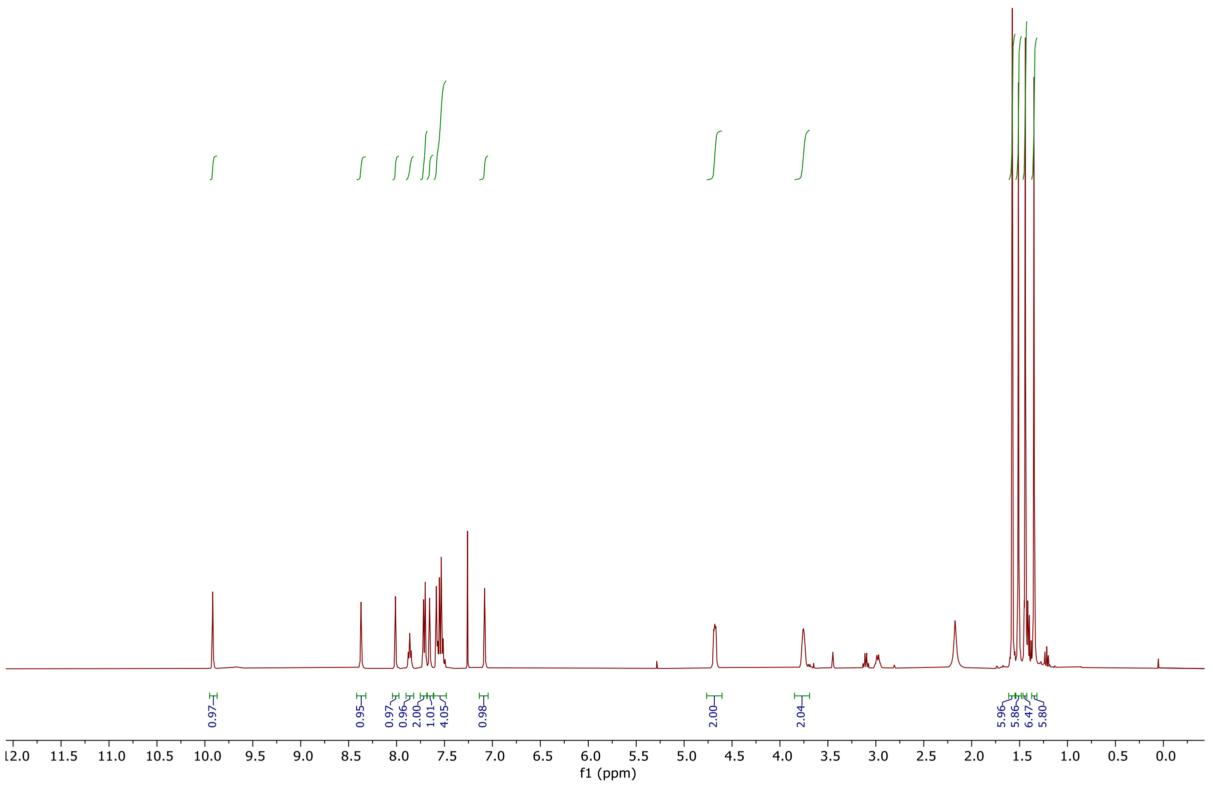

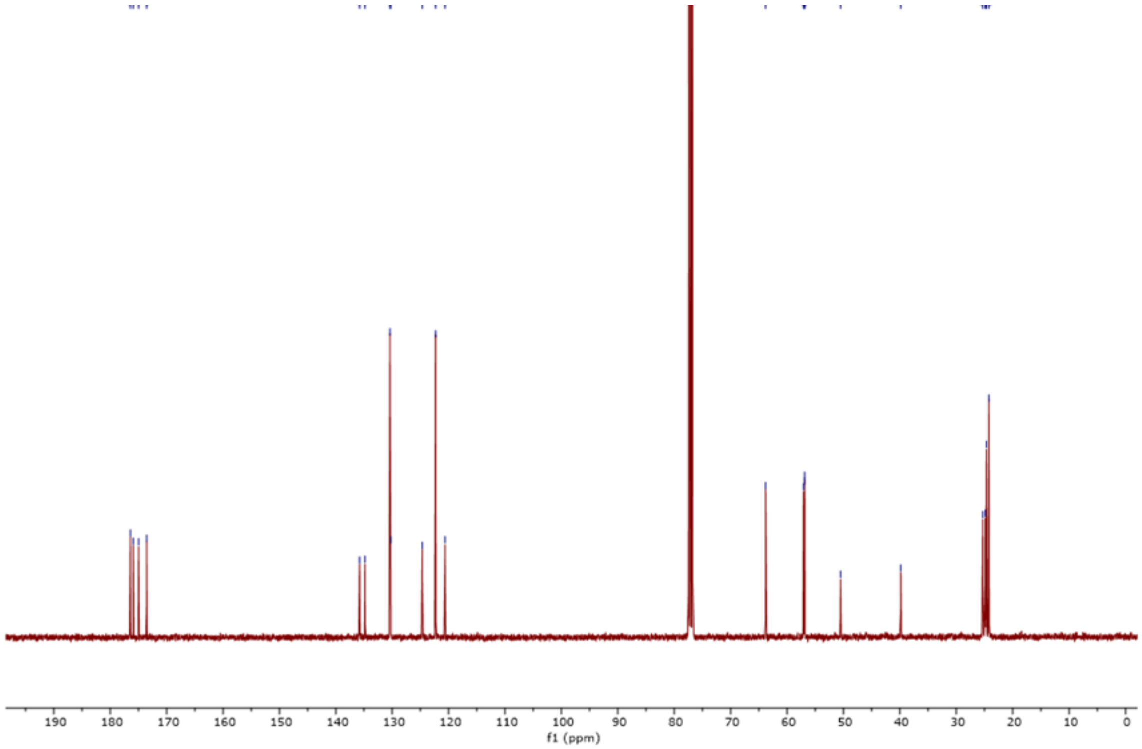

190
180
170
160
150
140
130
120
110
100
90
80
70
60
50
40
30
20
10
0
f1 (ppm)

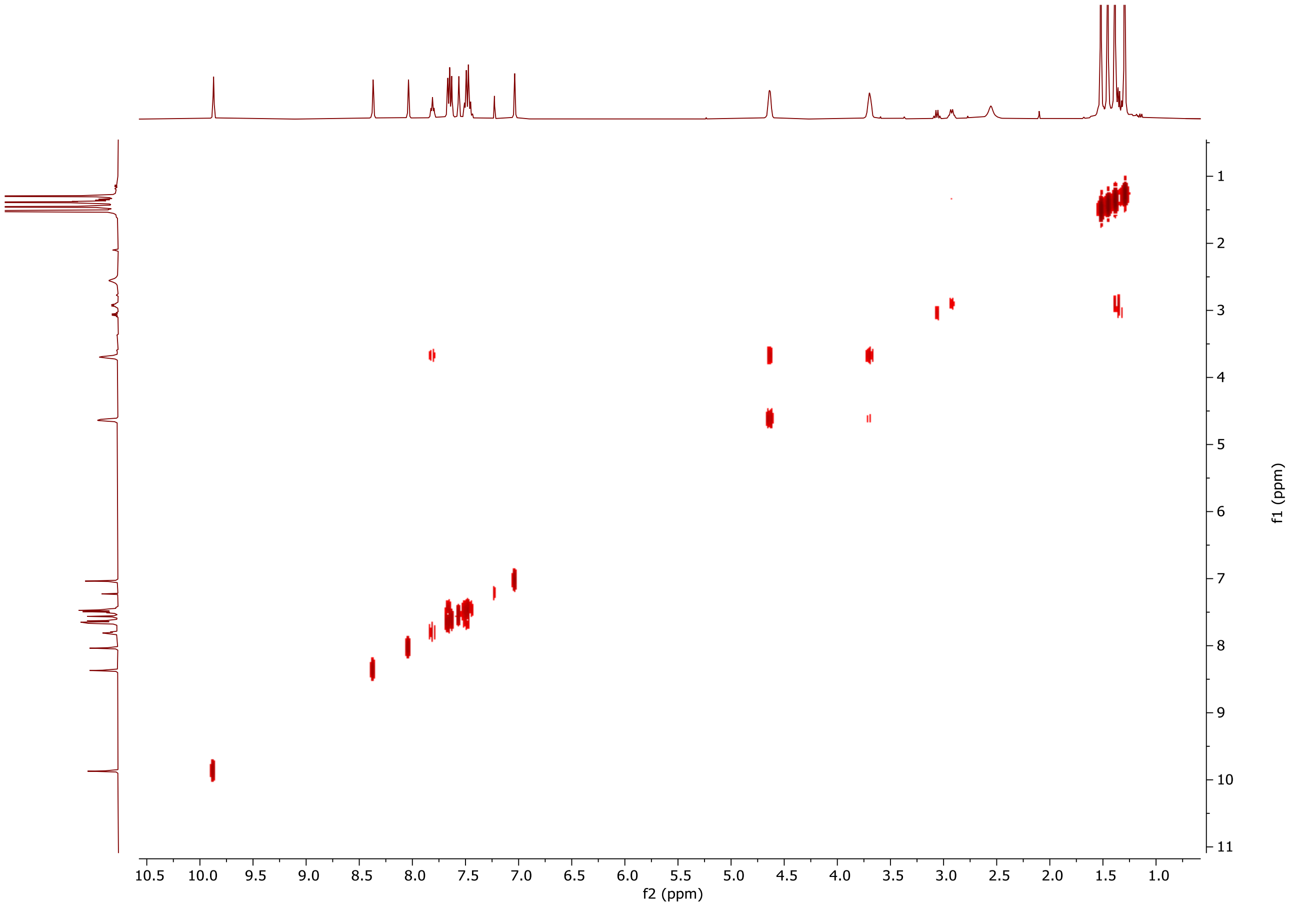

10.5
10.0
9.5
9.0
8.5
8.0
7.5
7.0
6.5
6.0
5.5
5.0
4.5
4.0
3.5
3.0
2.5
2.0
1.5
1.0
f2 (ppm)
f1 (ppm)

4.8 Foldamer 2 [(N_3Aib$_4$NH(CH_2)$_2$-NHC-Ph)Rh(Cl)COD)]

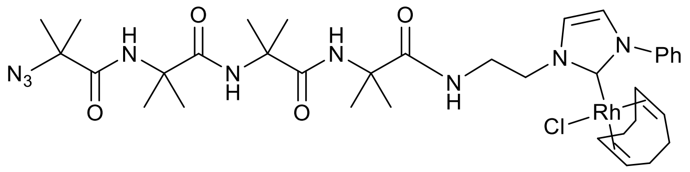

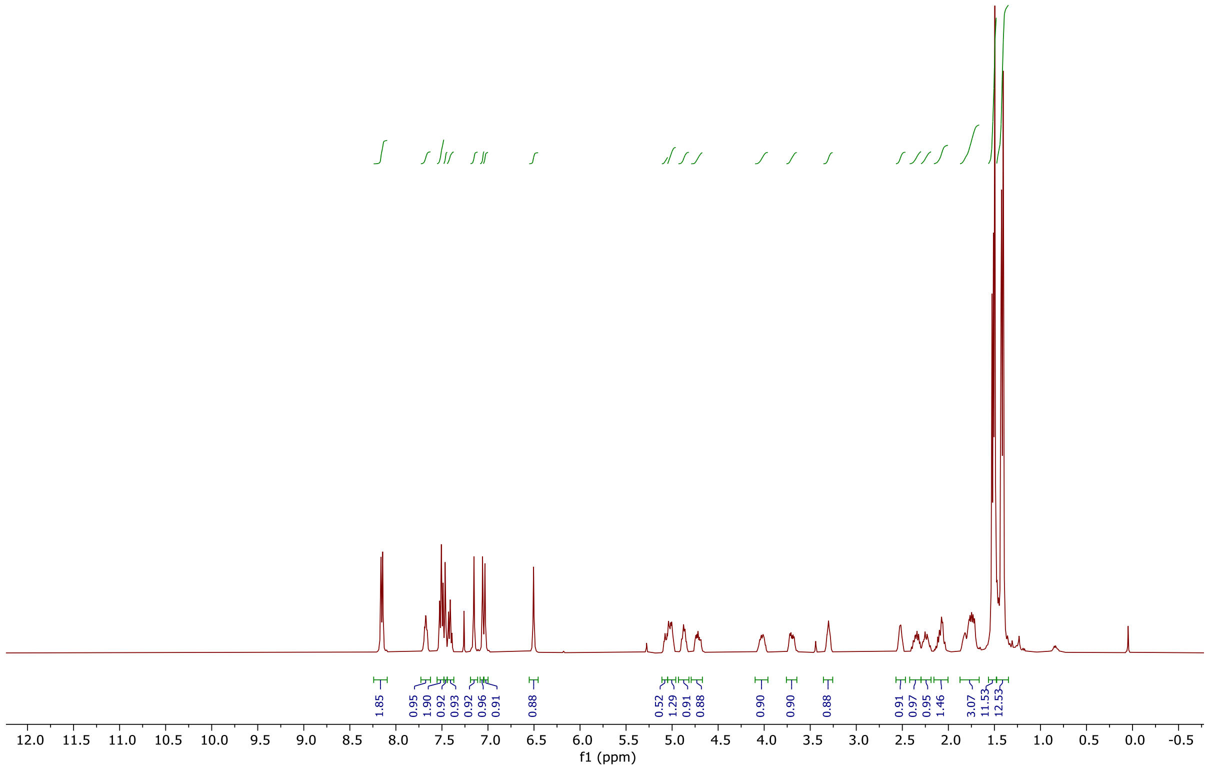

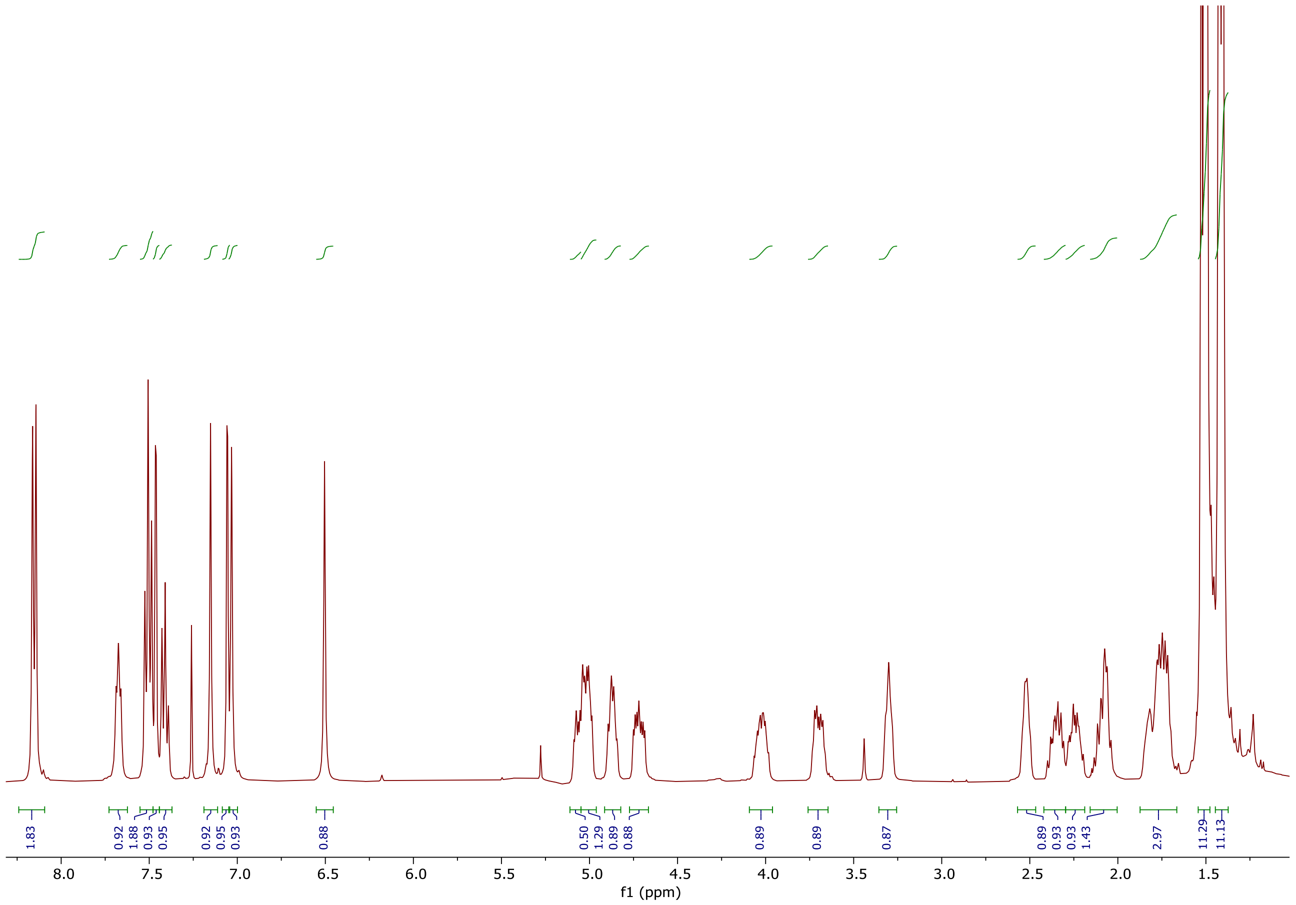
1.83
0.92
1.88
0.93
0.95
0.92
0.95
0.93
0.88
0.50
1.29
0.89
0.88
0.89
0.89
0.87
0.89
0.93
0.93
1.43
2.97
11.29
11.13
8.0 7.5 7.0 6.5 6.0 5.5 5.0 4.5 4.0 3.5 3.0 2.5 2.0 1.5
f1 (ppm)

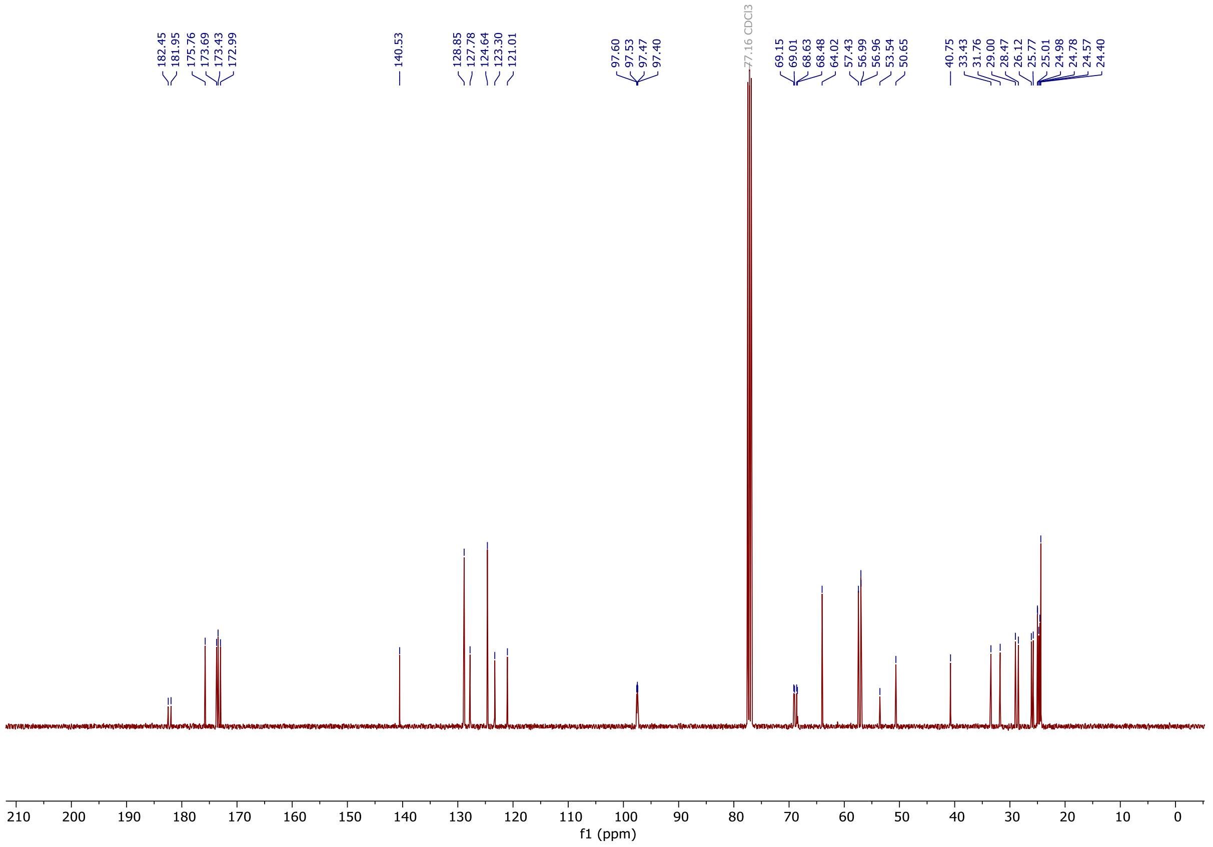
182.45
181.95
175.76
173.69
173.43
172.99
140.53
128.85
127.78
124.64
123.30
121.01
97.60
97.53
97.47
97.40
77.16 CDCl3
69.15
69.01
68.63
68.48
64.02
57.43
56.99
56.96
53.54
50.65
40.75
33.43
31.76
29.00
28.47
26.12
25.77
25.01
24.98
24.78
24.57
24.40
210 200 190 180 170 160 150 140 130 120 110 100 90 80 70 60 50 40 30 20 10 0
f1 (ppm)

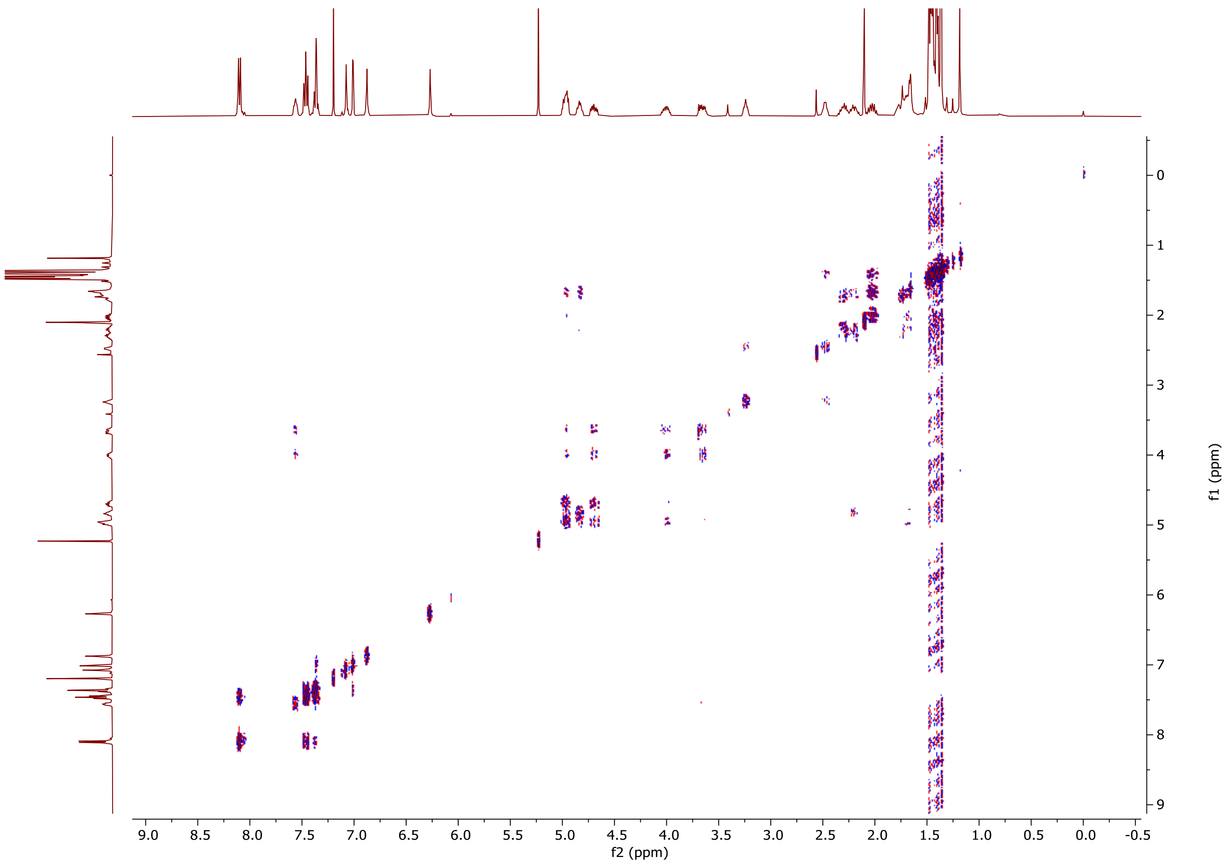

^{1}H ^{13}C HSQC

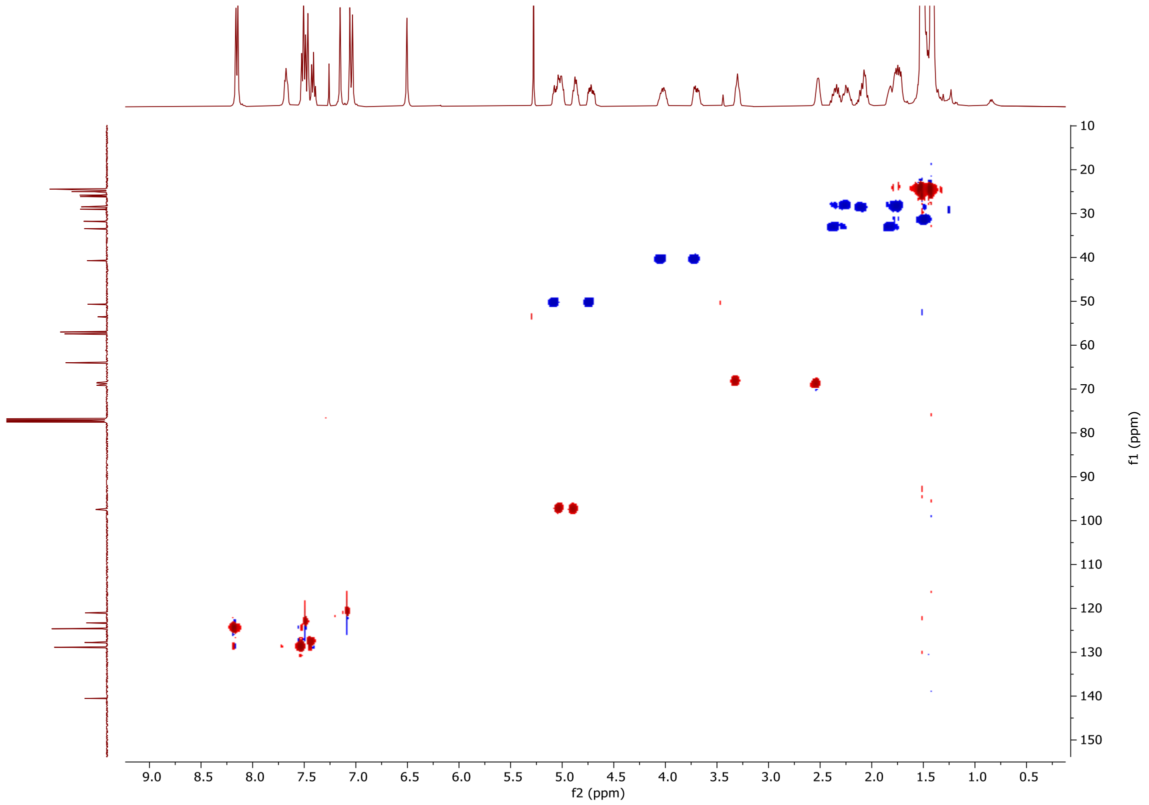

^{1}H ^{13}C HMBC

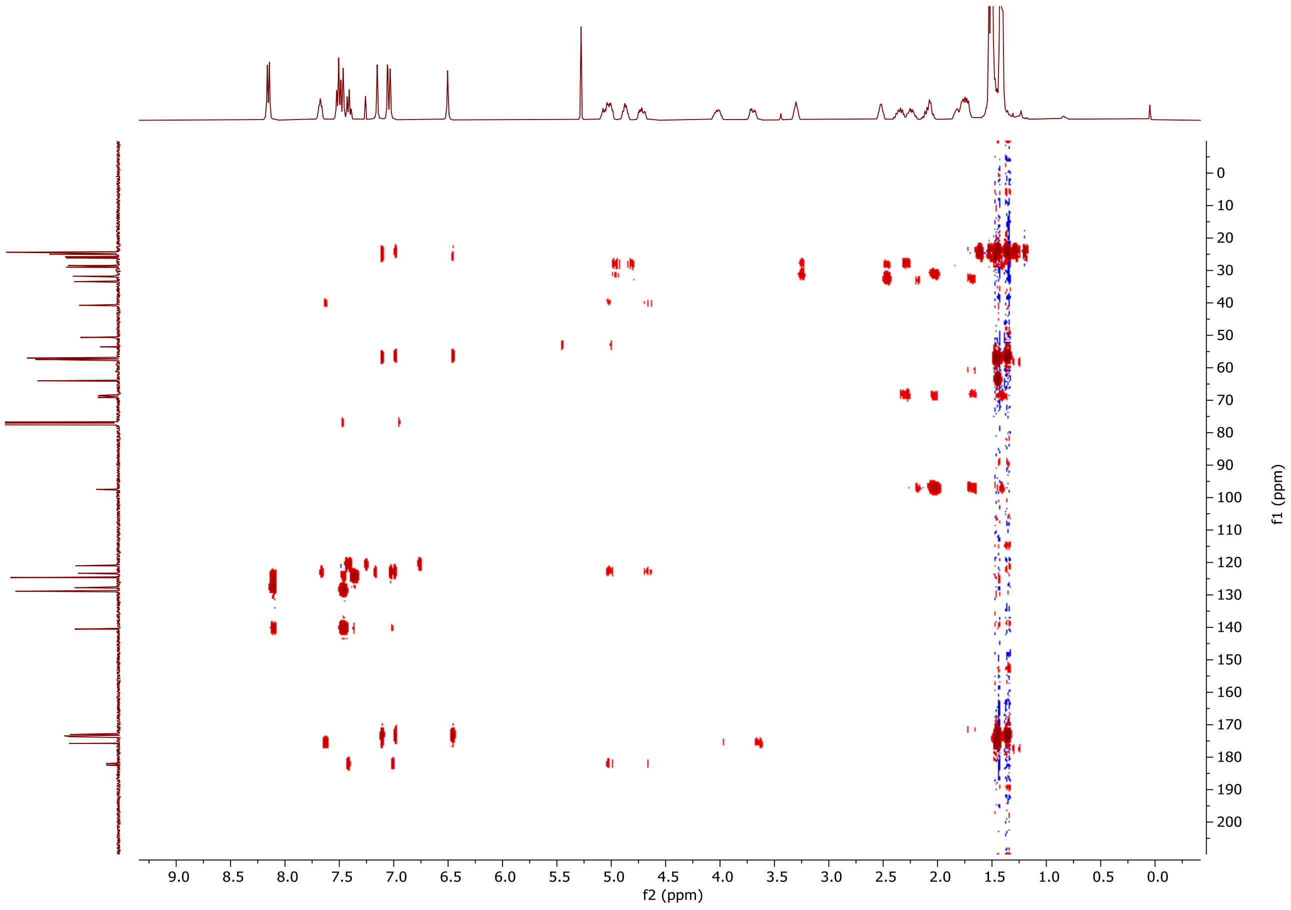

4.9 Foldamer 8, [(N_3Aib$_4$NH(CH_2)$_2$-NHC-Ph)Rh(Cl)(NBD)]

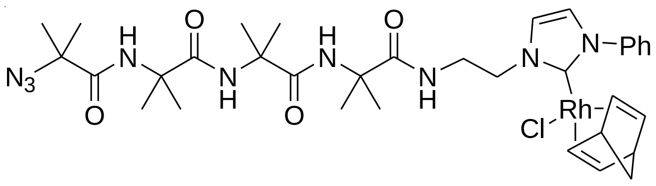

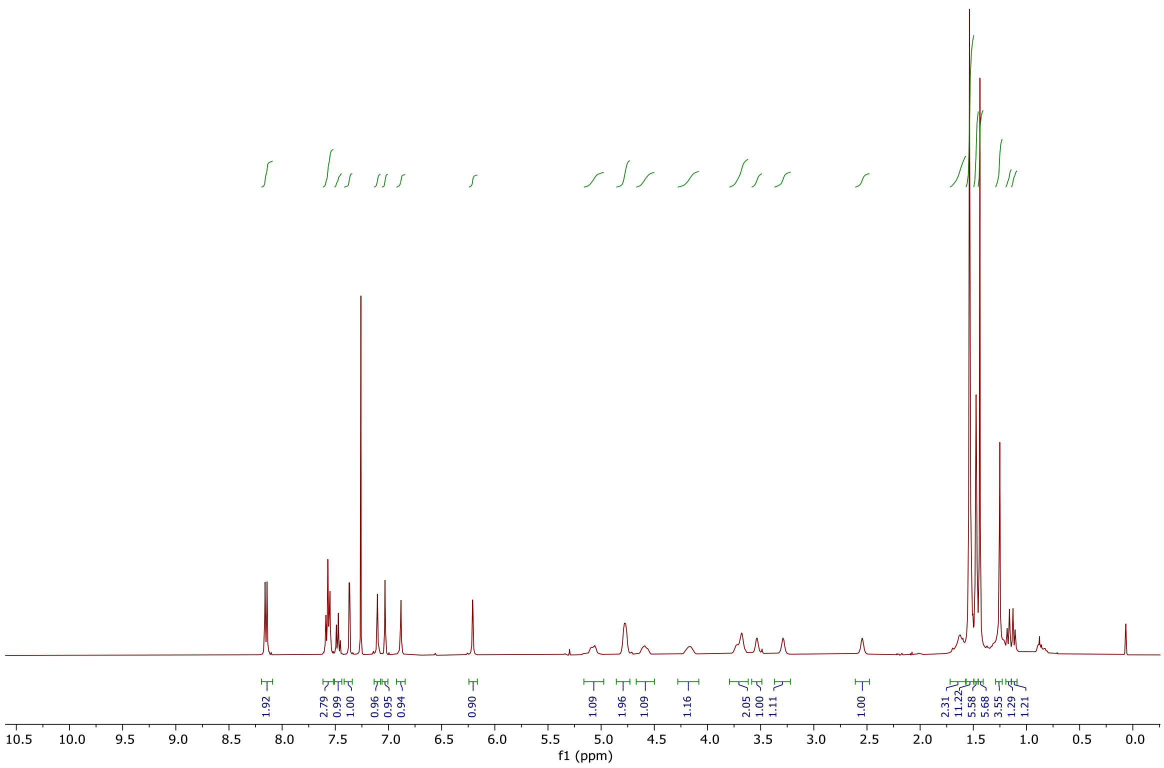

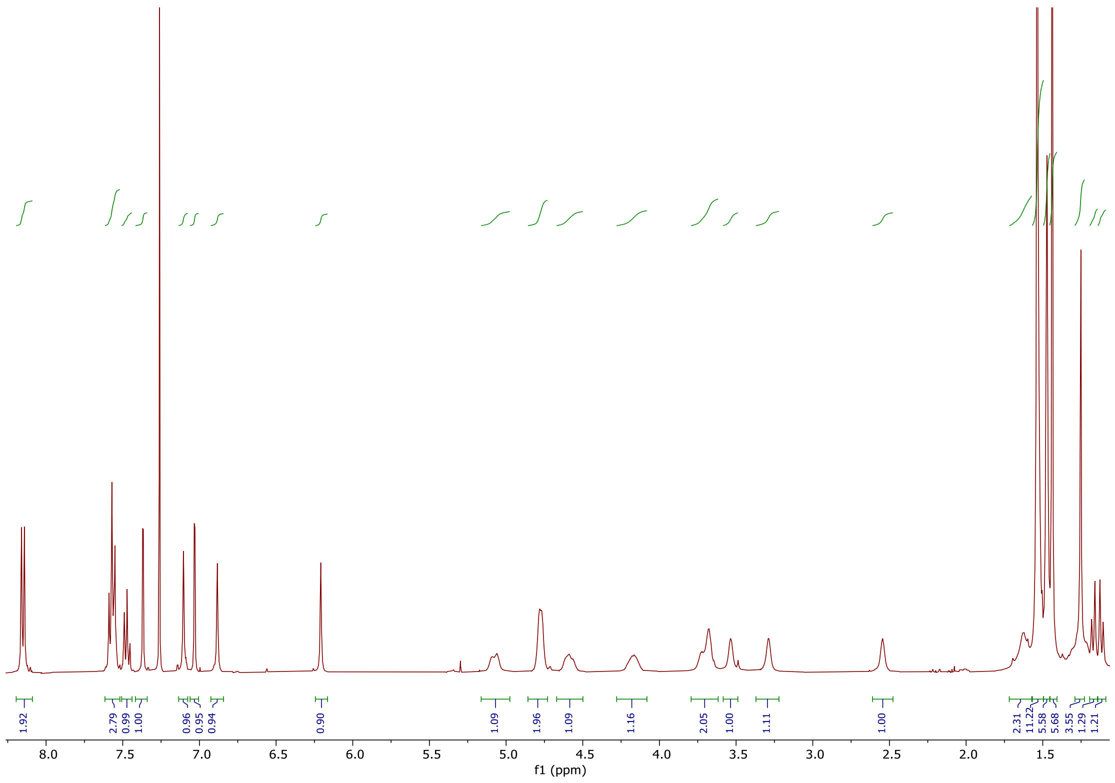

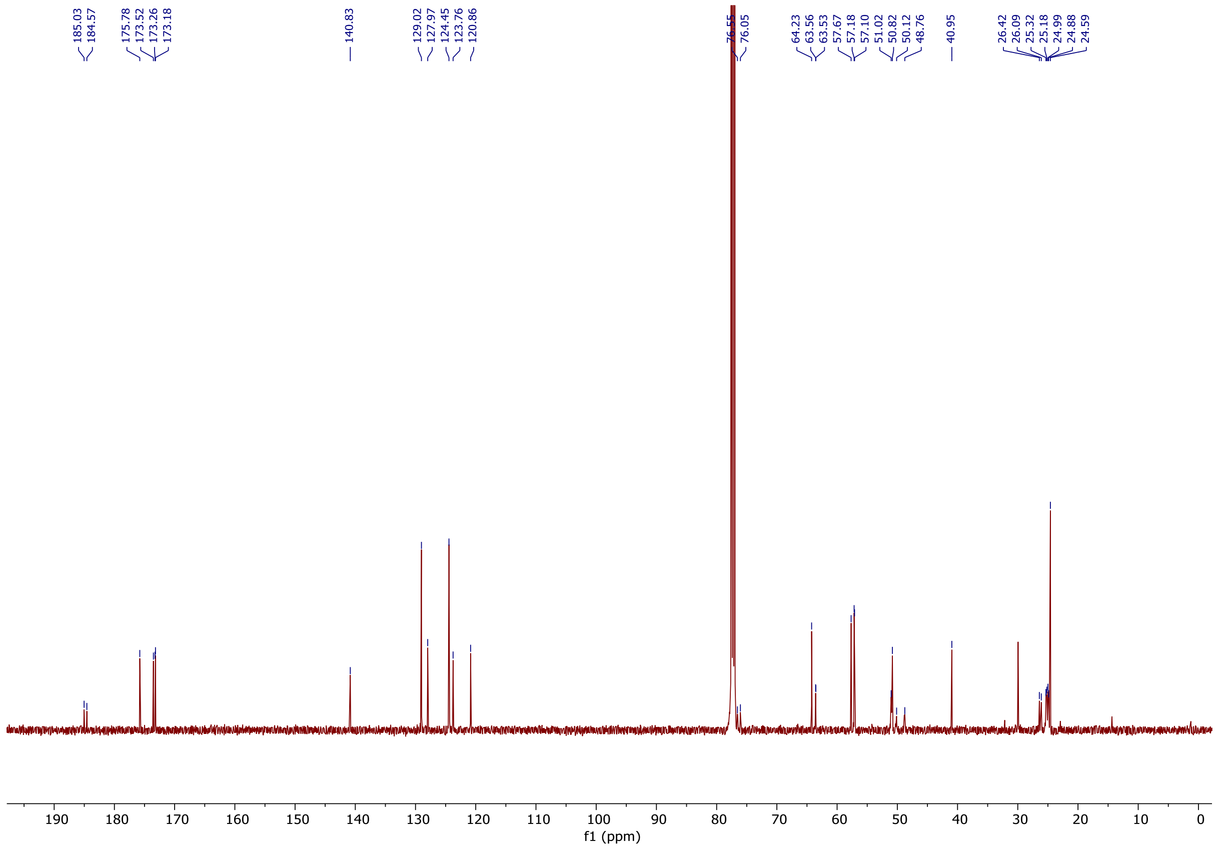
185.03
184.57
175.78
173.52
173.26
173.18
140.83
129.02
127.97
124.45
123.76
120.86
76.55
76.05
64.23
63.56
63.53
57.67
57.18
57.10
51.02
50.82
50.12
48.76
40.95
26.42
26.09
25.32
25.18
24.99
24.88
24.59
f1 (ppm)

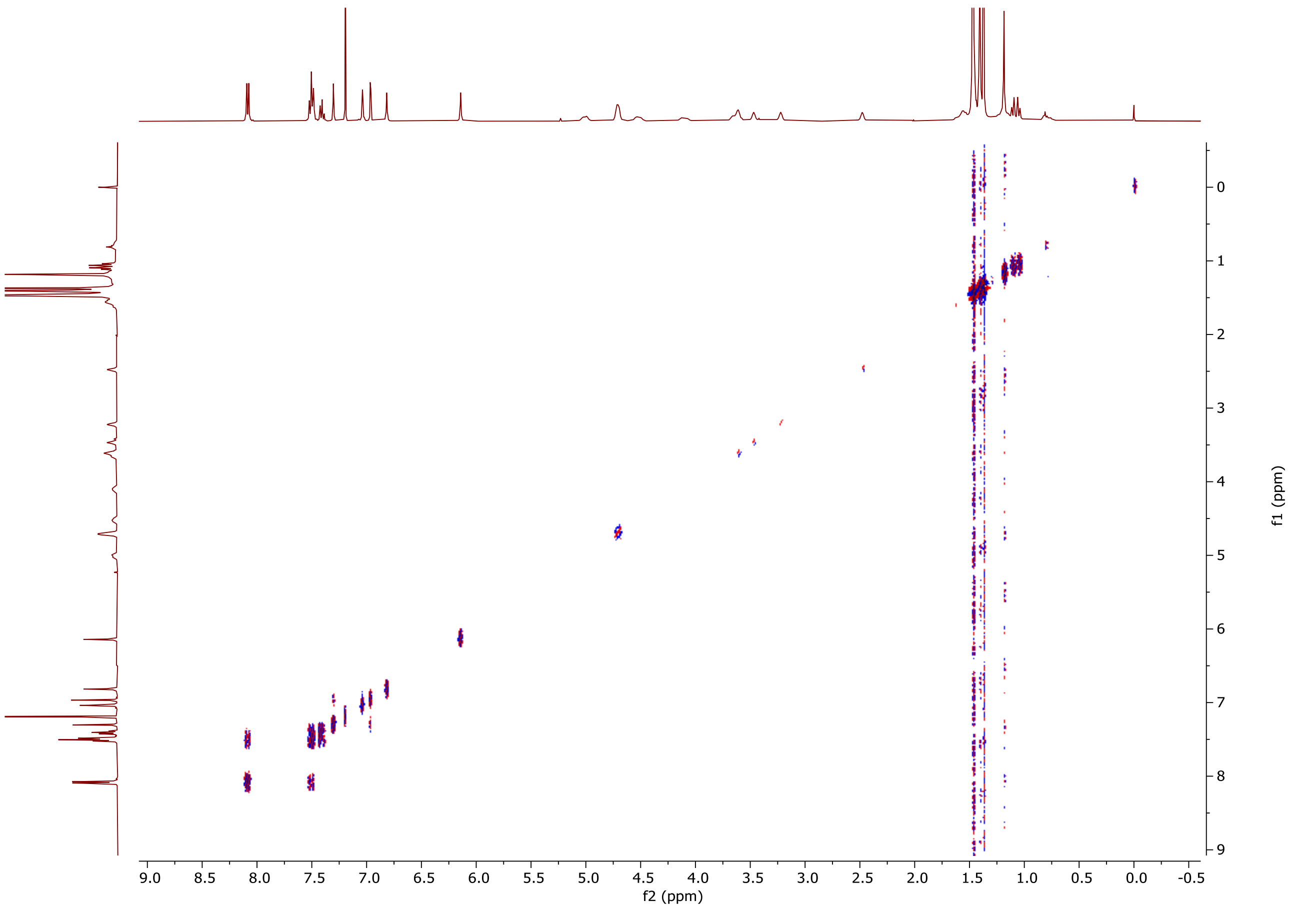
f2 (ppm)
f1 (ppm)

^1H ^{13}C HSQC

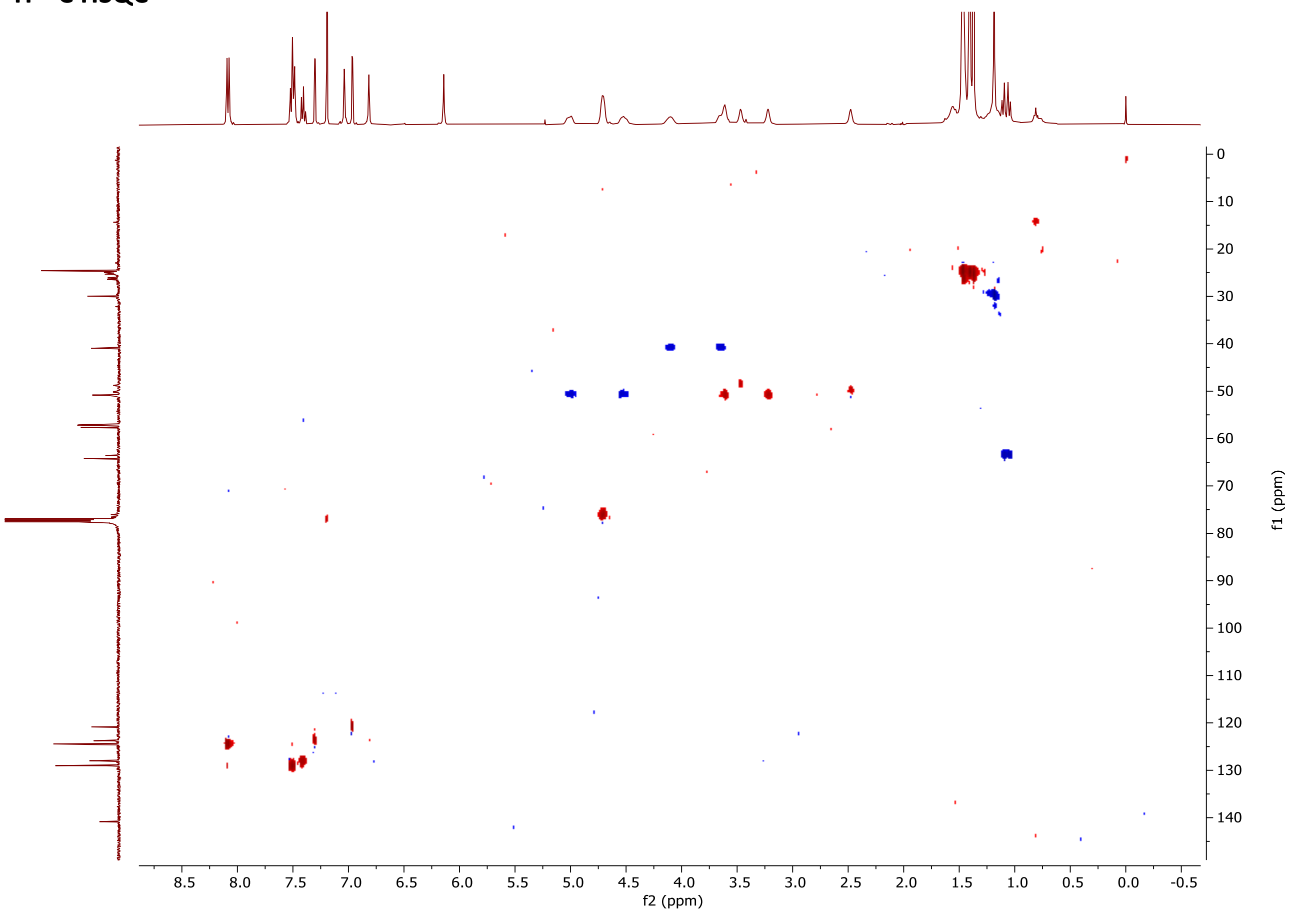

^1H ^{13}C HMBC

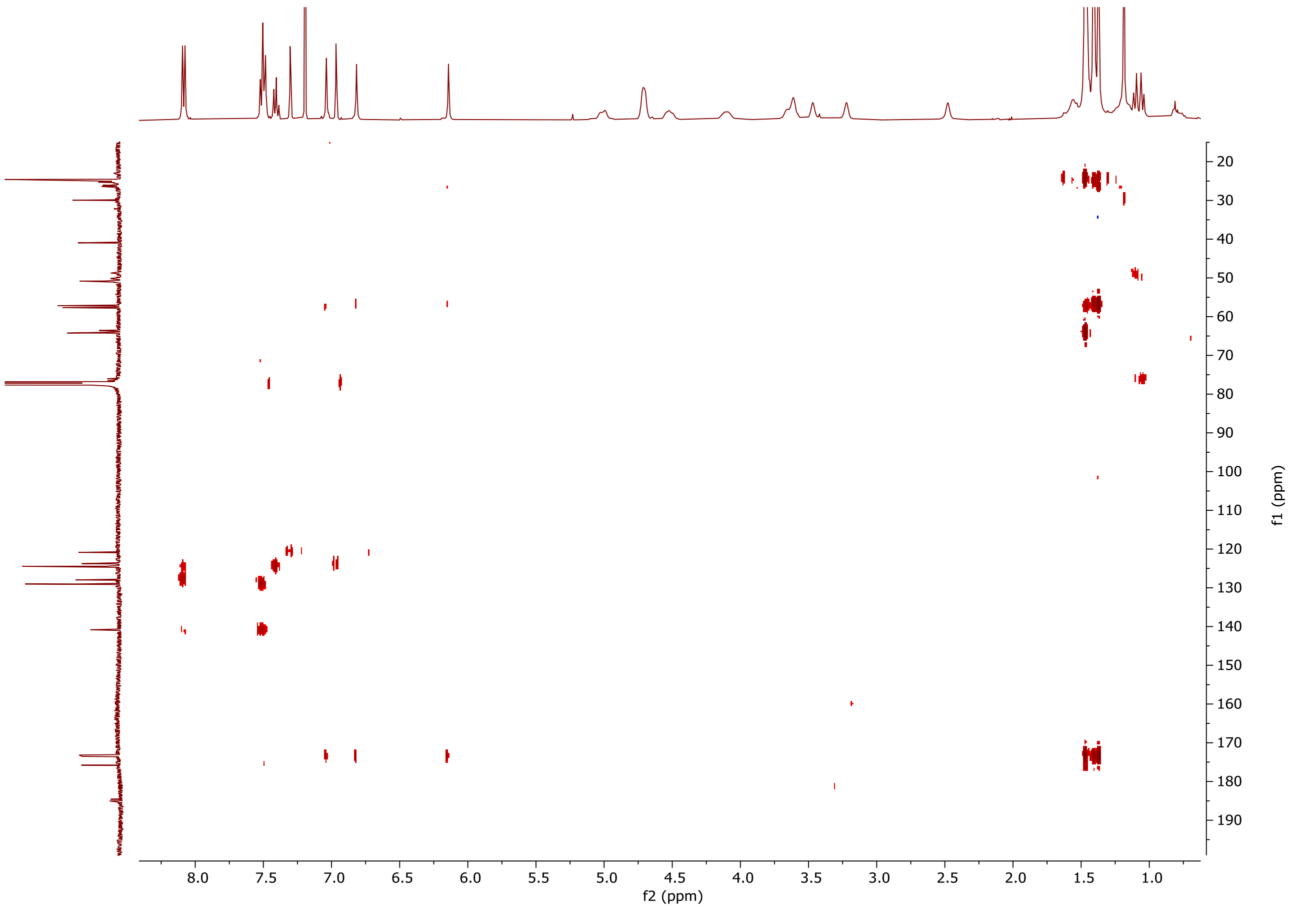

4.10 Precursor S3 [$N_3Aib_4(CH_2)_2$(Im-Mes)]$^+Br^-$

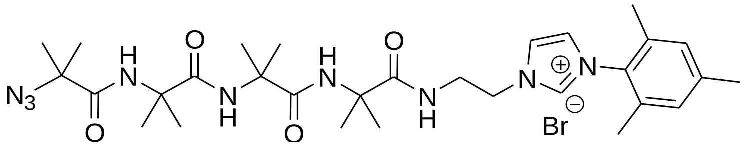

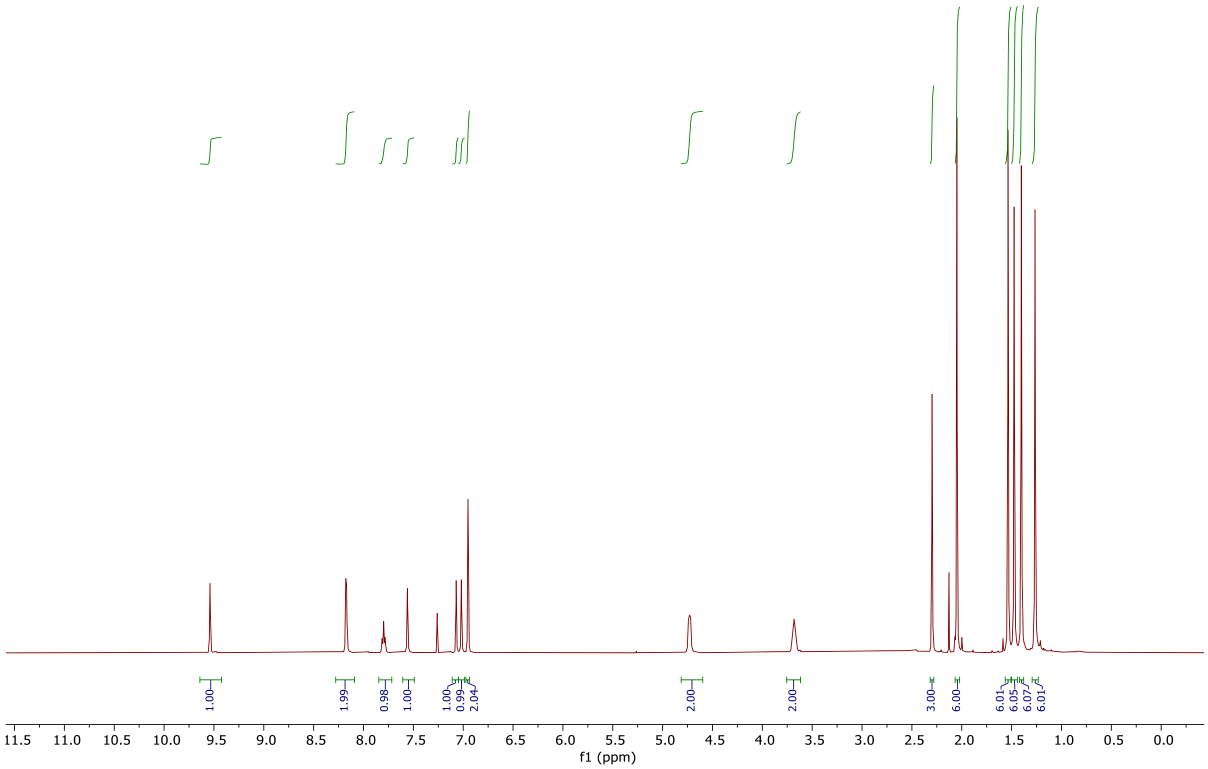

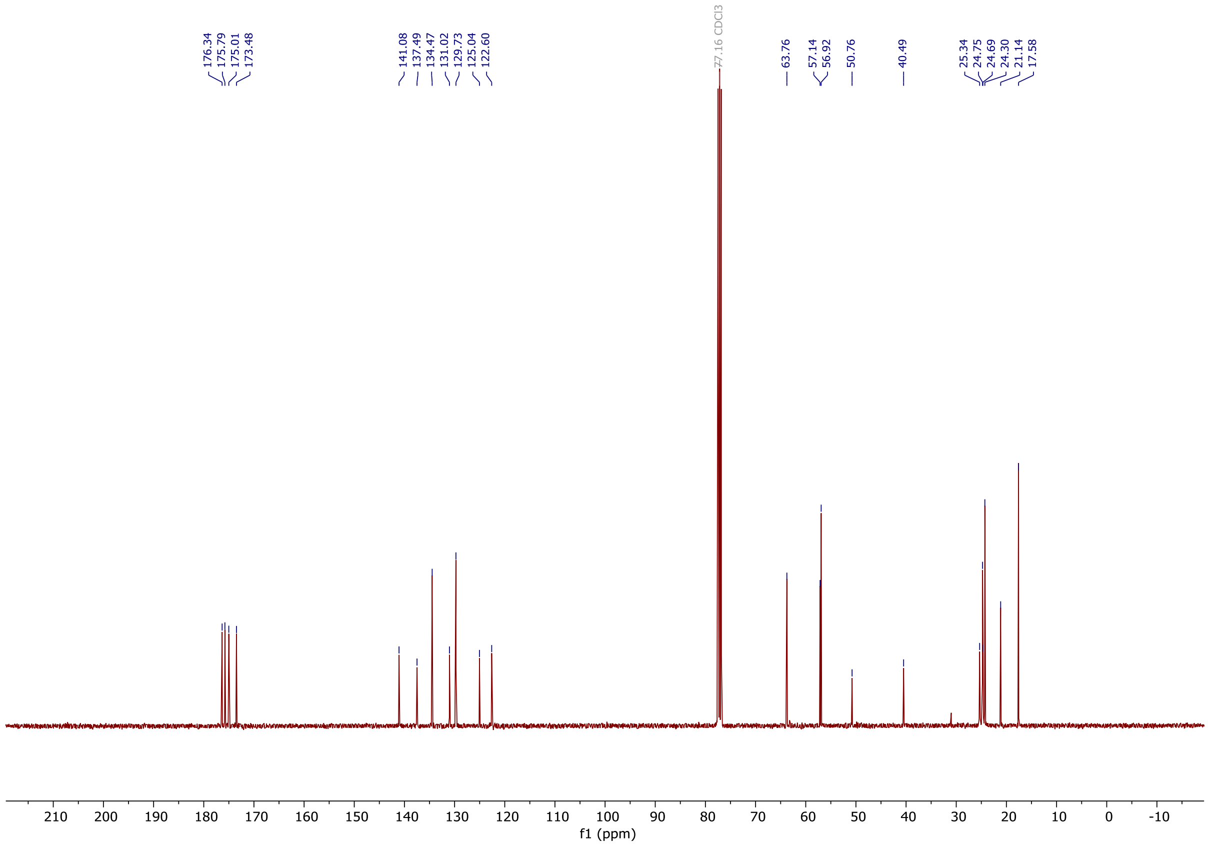

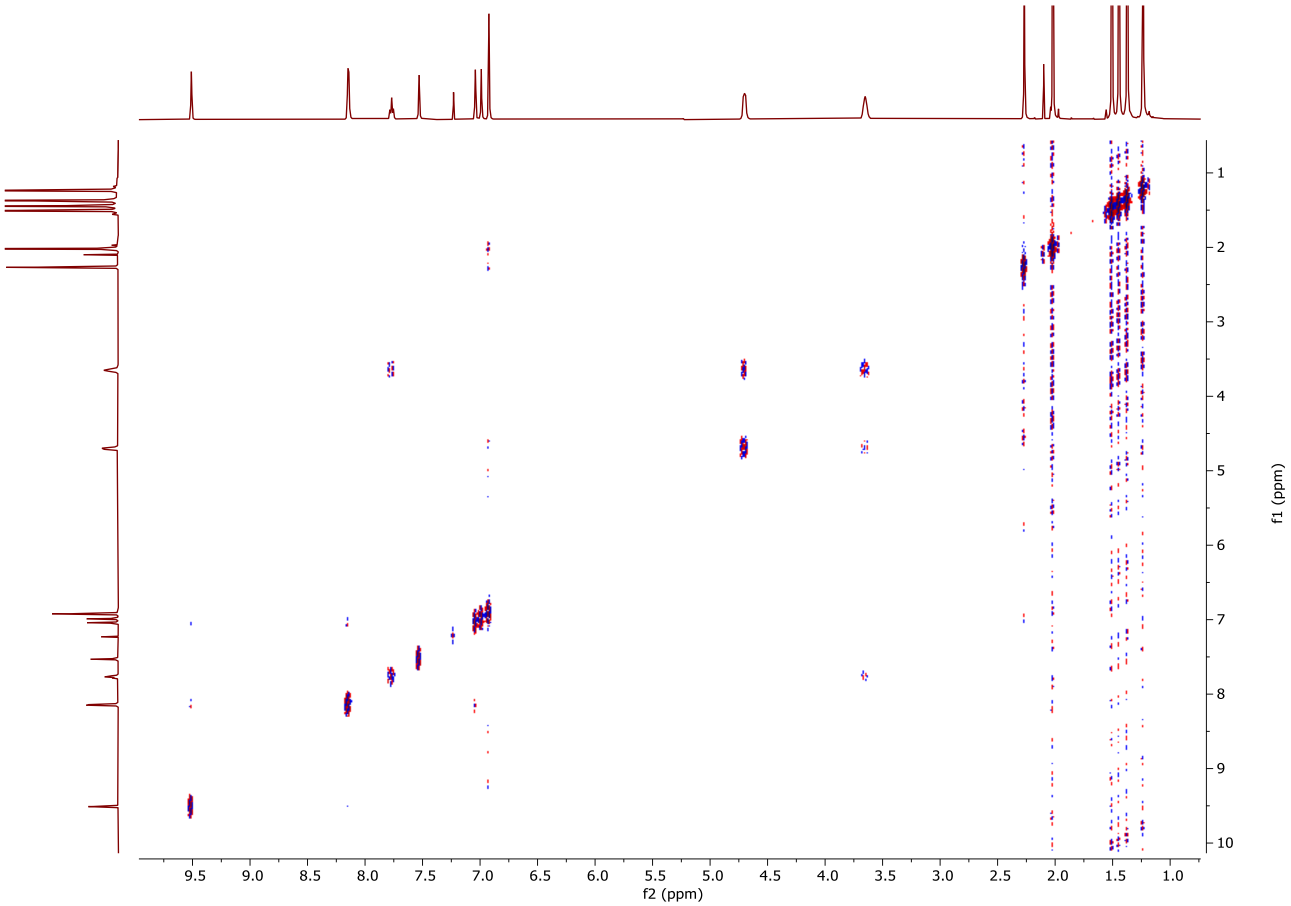
f2 (ppm)
f1 (ppm)

4.11 Foldamer 3 [(N_3Aib$_4$NH(CH_2)$_2$-NHC-Mes)Rh(Cl)COD)]

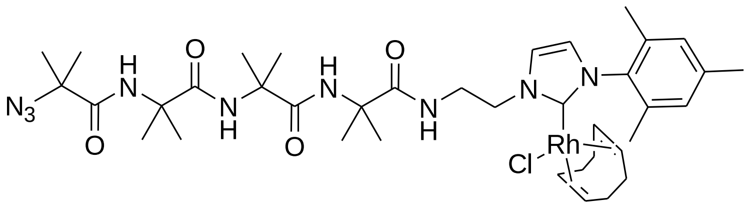

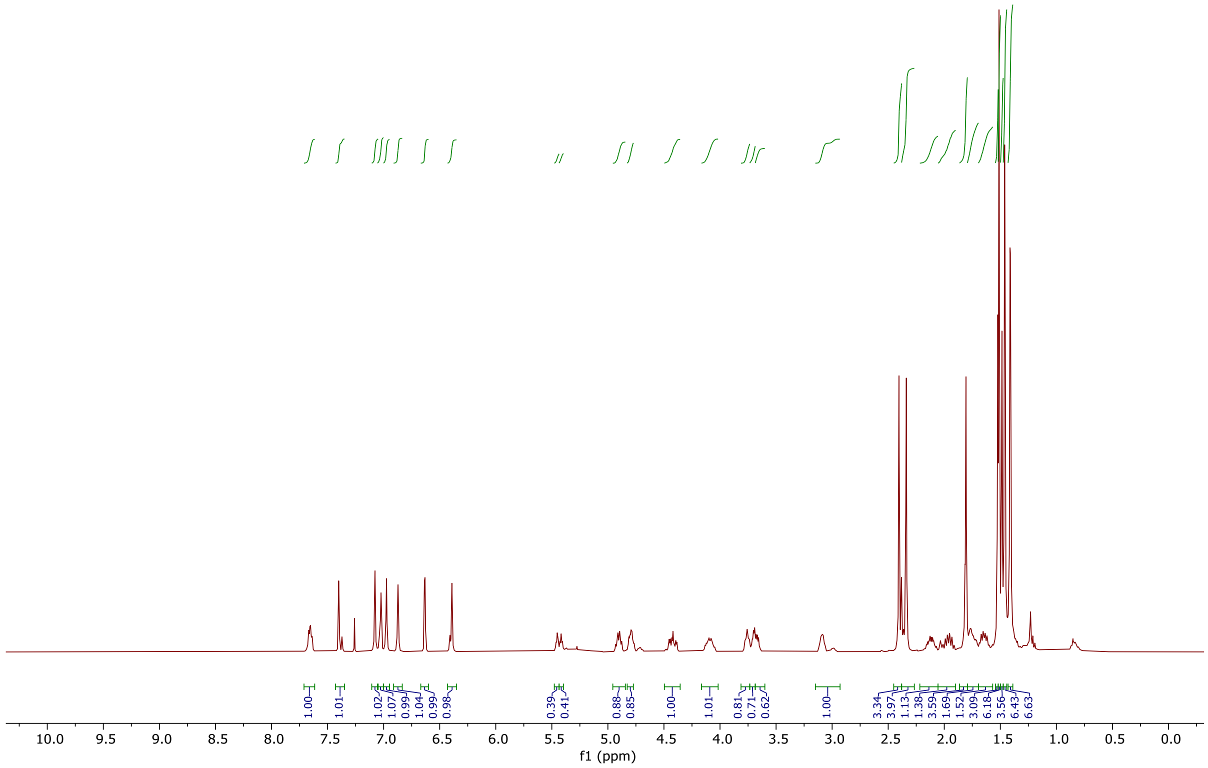

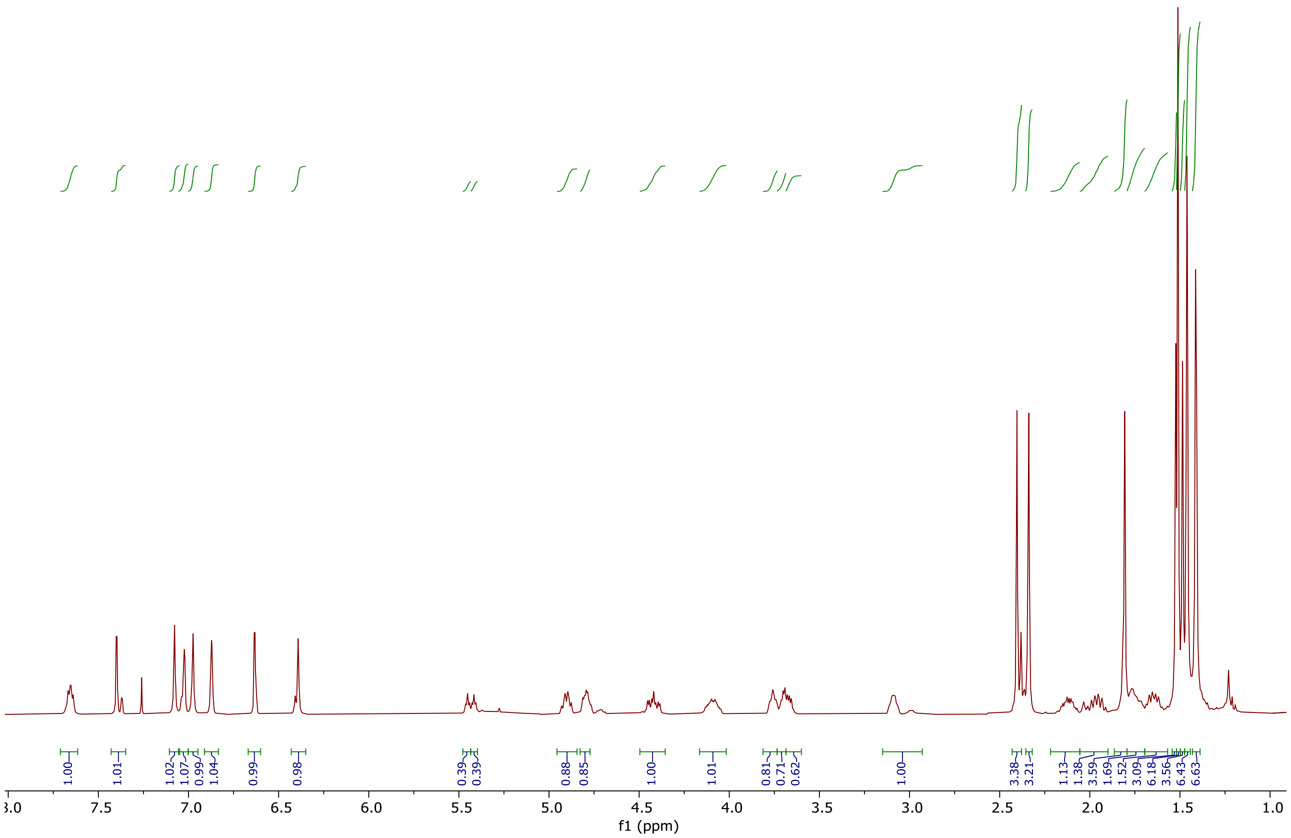

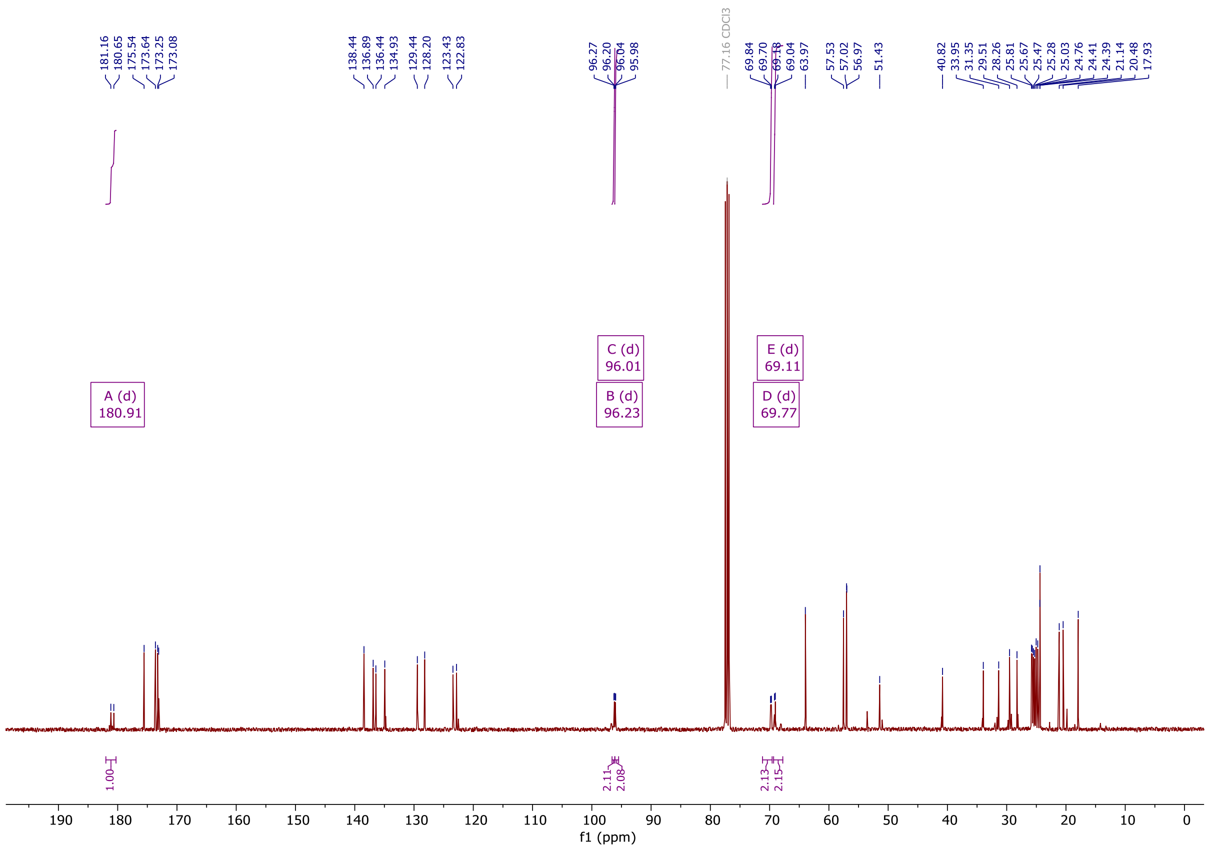

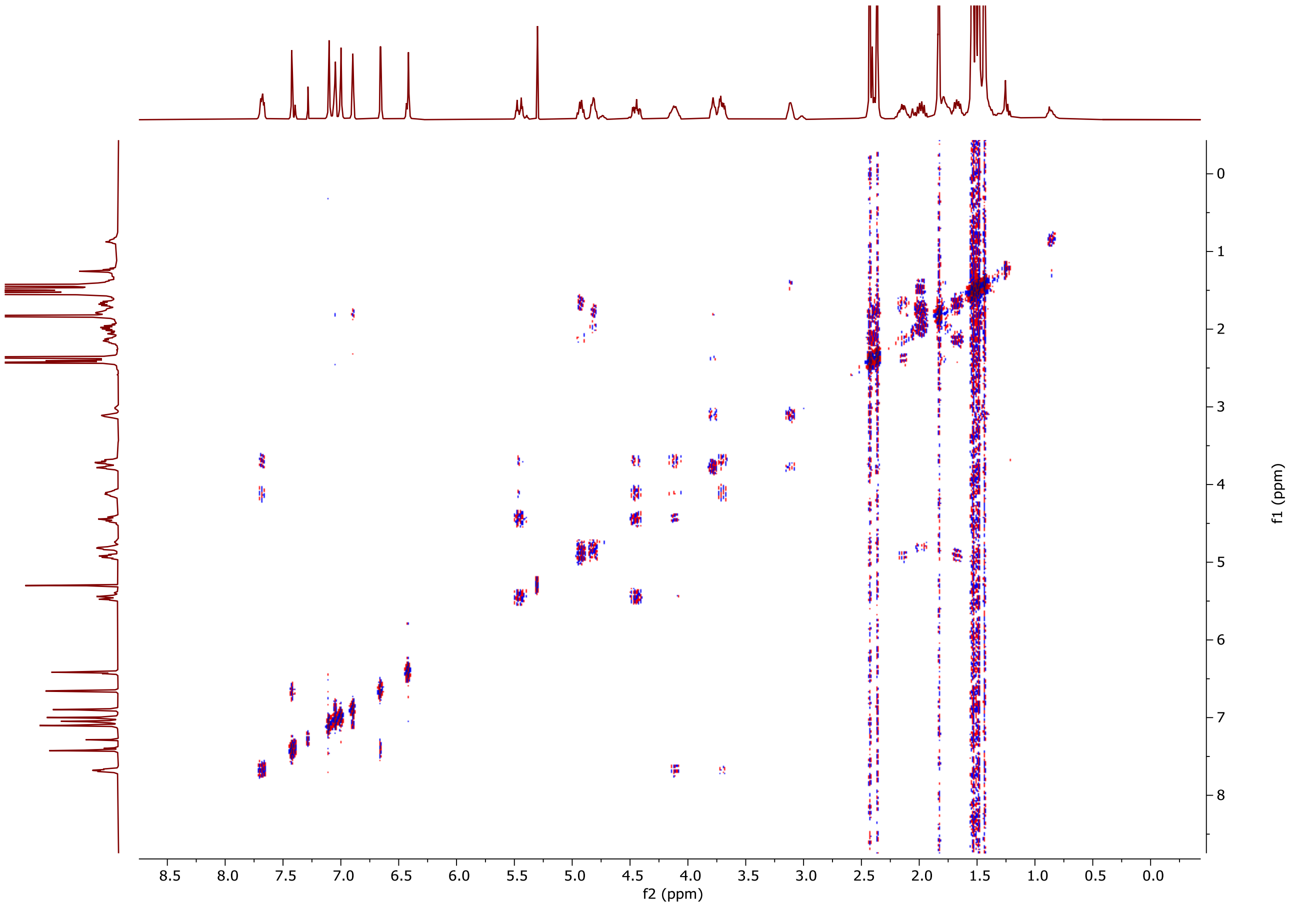

^{1}H ^{13}C HSQC

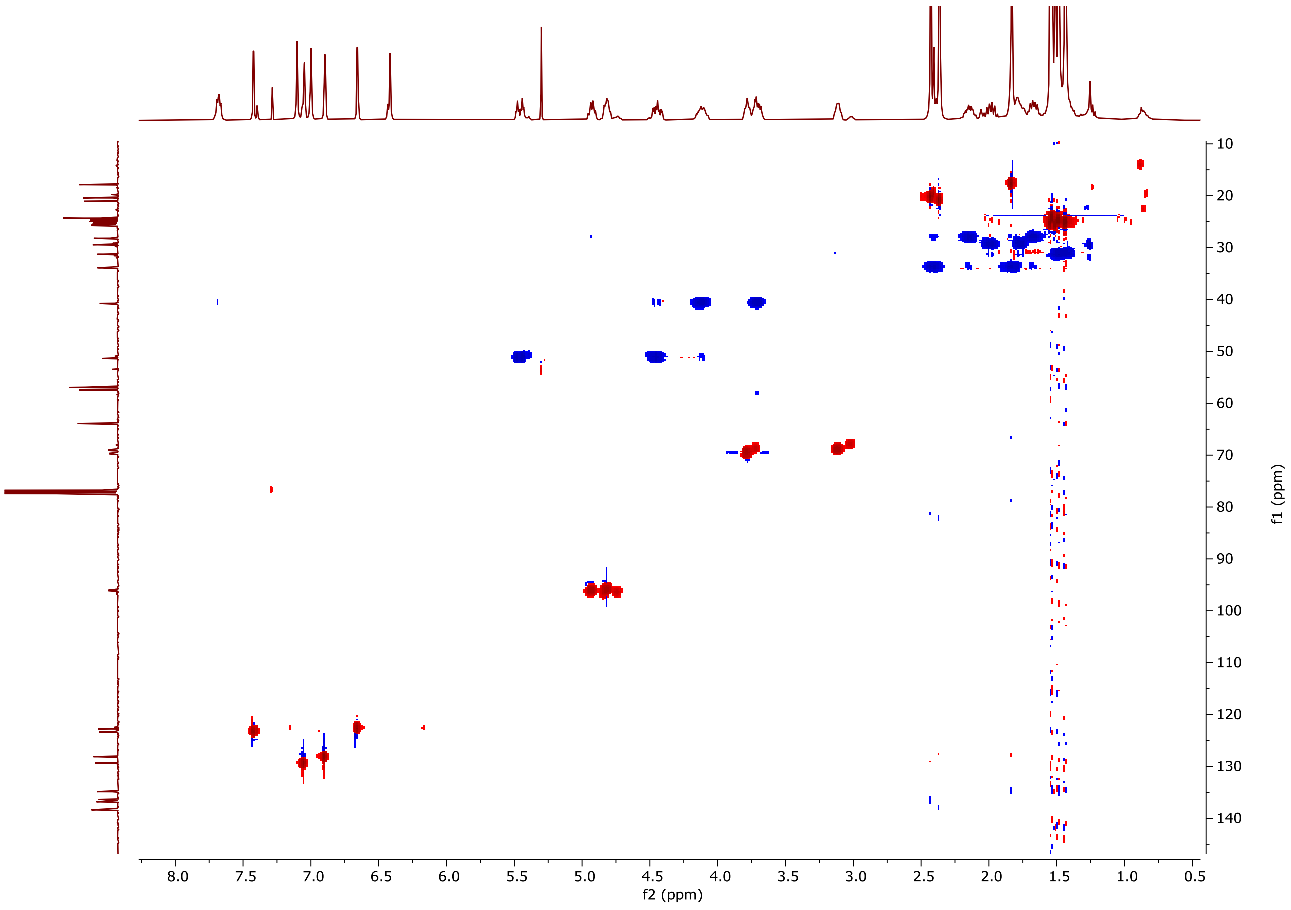

^{1}H ^{13}C HMBC

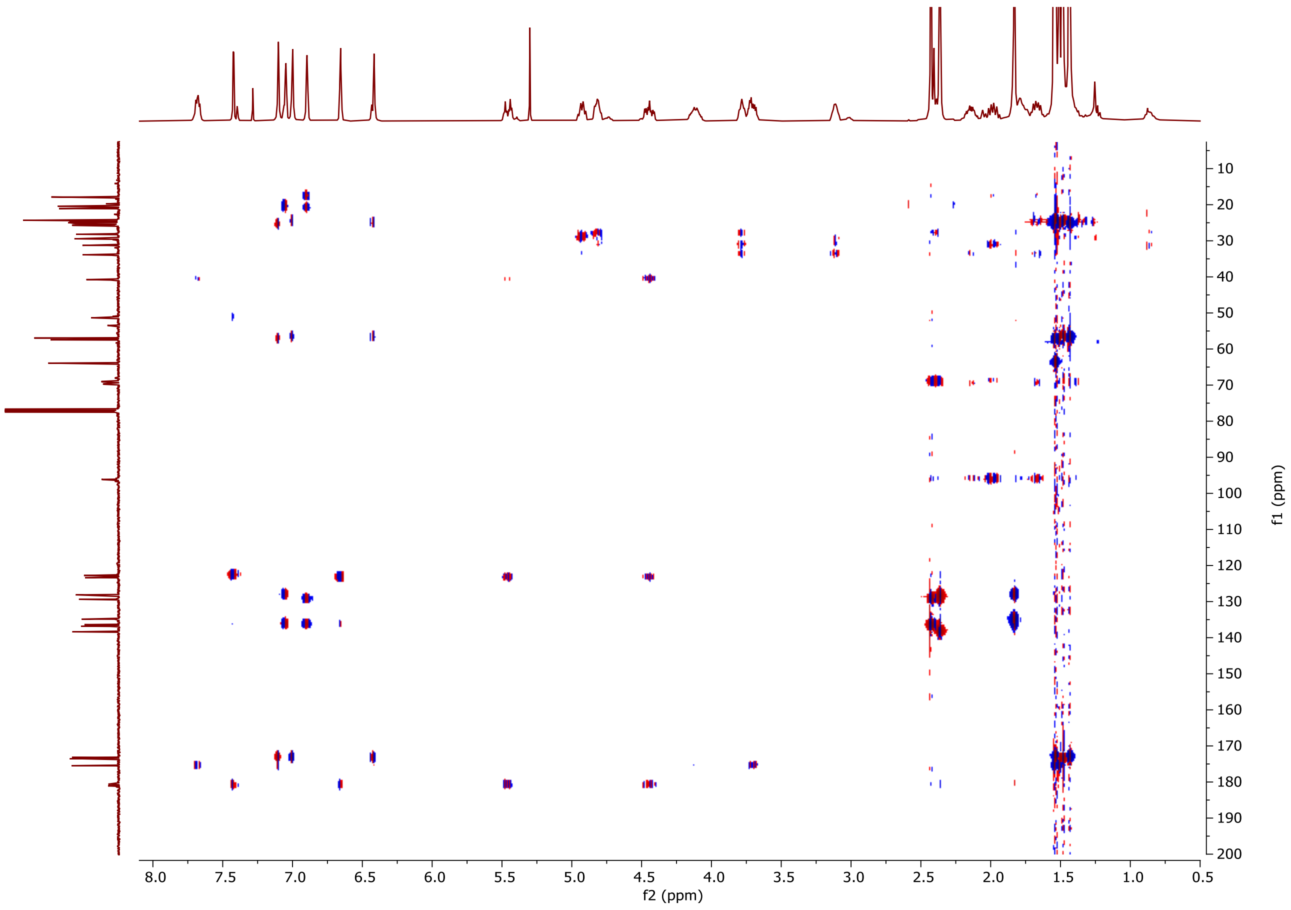

4.12 Precursor S4 [$N_3Aib_4(CH_2)_2(Im\text{-}Me)]^+Br^-$

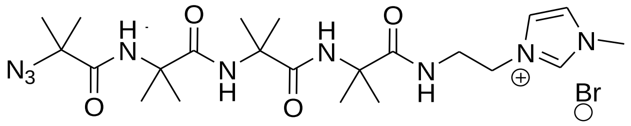

In $CDCl_3$

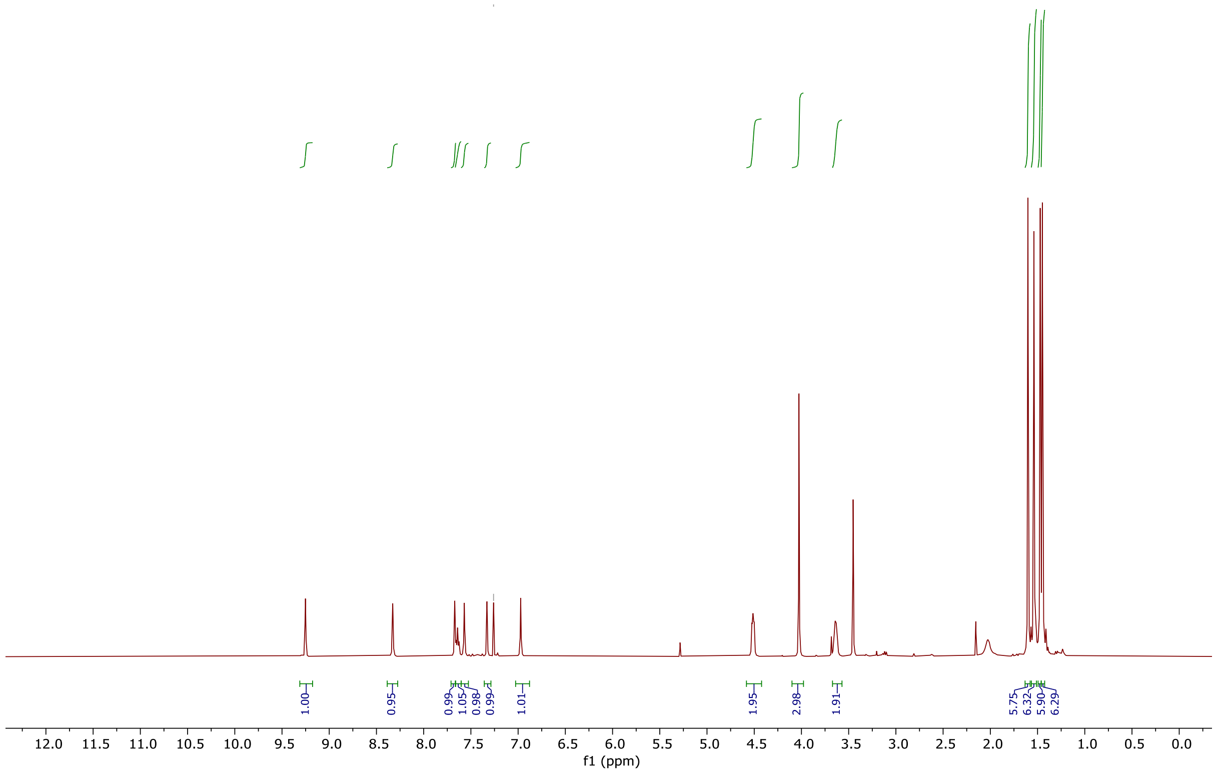

In MeOD (due to limited solubility in chloroform)

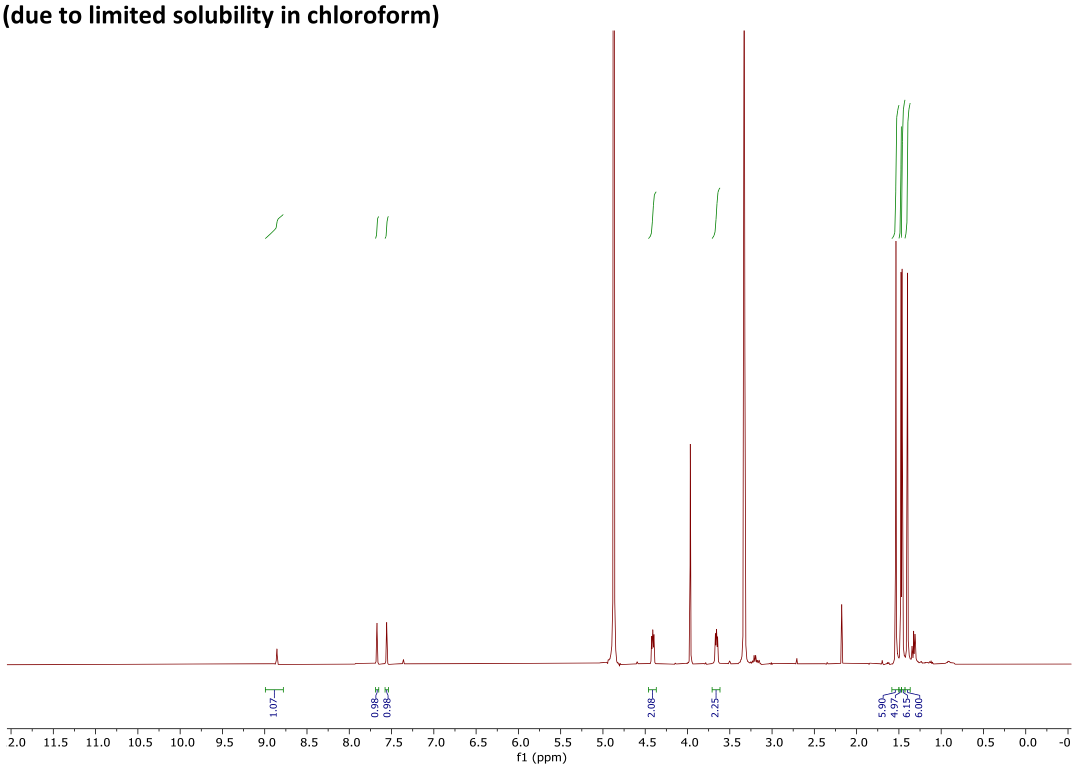

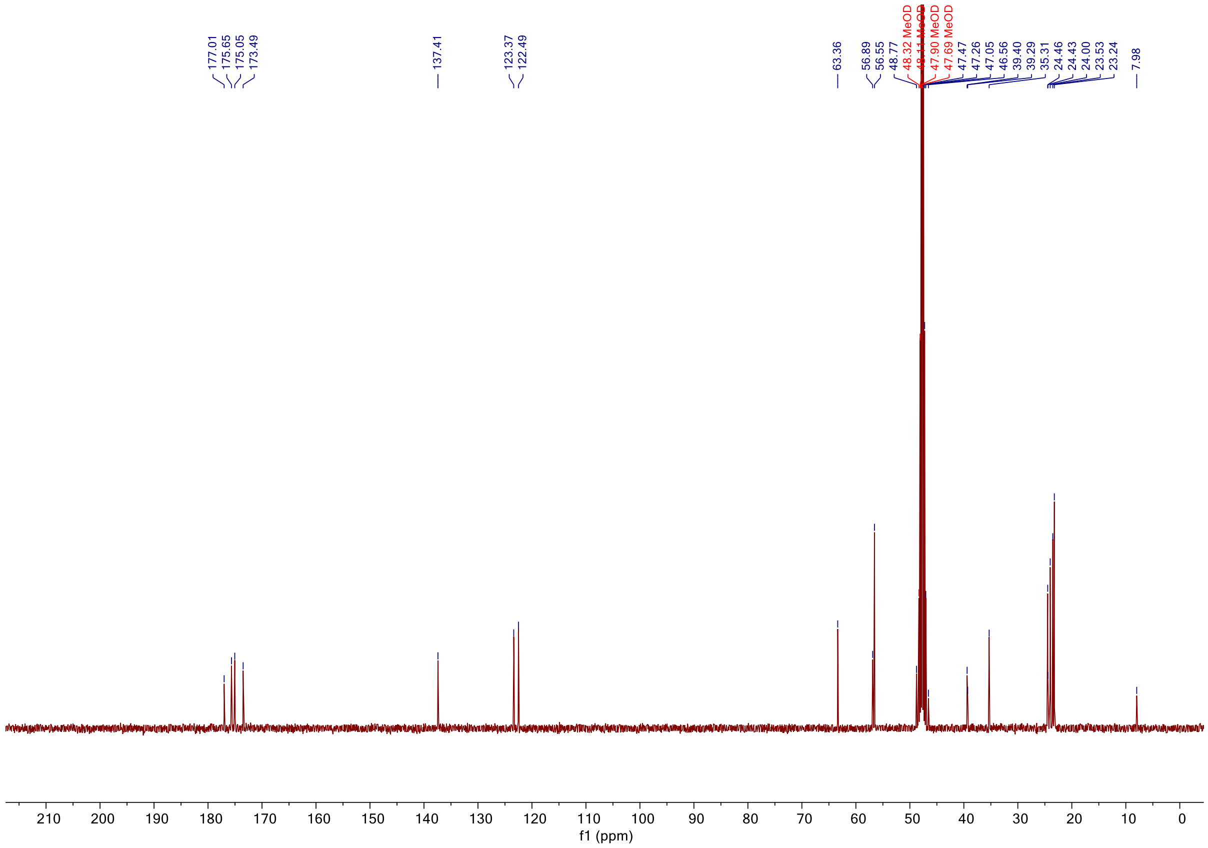
177.01
175.65
175.05
173.49
137.41
123.37
122.49
63.36
56.89
56.55
48.77
48.32 MeOD
47.90 MeOD
47.69 MeOD
47.47
47.26
47.05
46.56
39.40
39.29
35.31
24.46
24.43
24.00
23.53
23.24
7.98
210
200
190
180
170
160
150
140
130
120
110
100
90
80
70
60
50
40
30
20
10
0
f1 (ppm)

4.13 Foldamer 4 [(N₃Aib₄NH(CH₂)₂-NHC-Me)Rh(Cl)COD)]

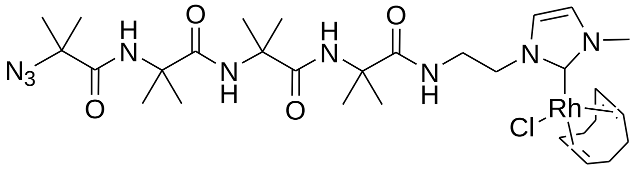

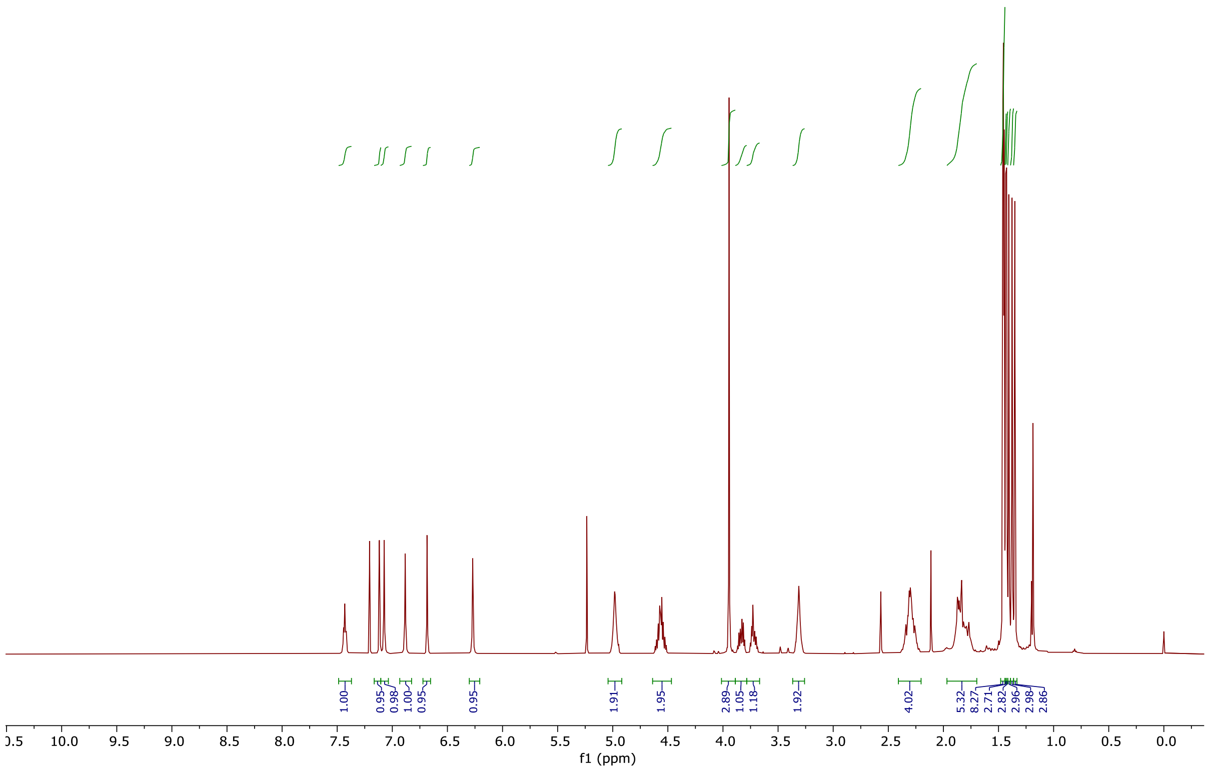

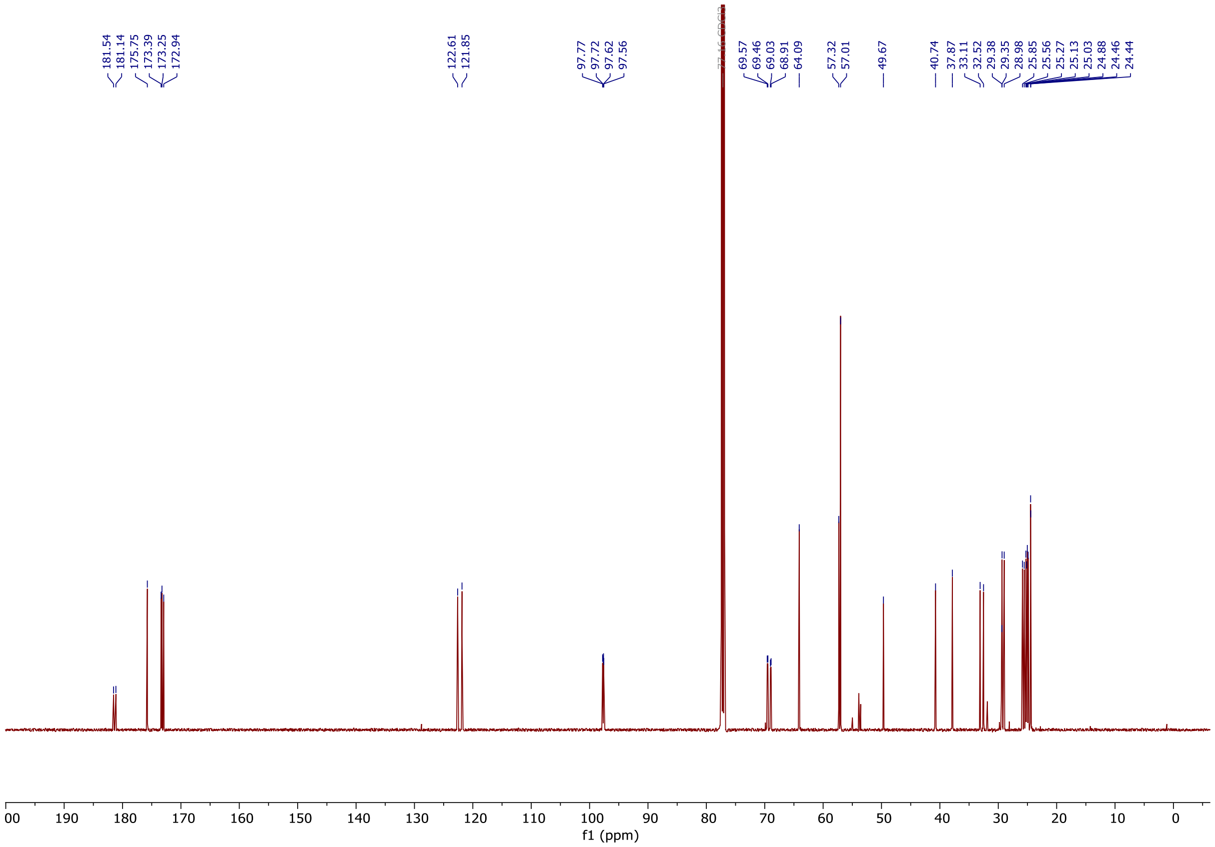

4.14 Precursor 9 [Cbz-(L-αMeVal)Aib$_4$NH(CH$_2$)$_2$(Im-Ph)]$^+$Br$^-$

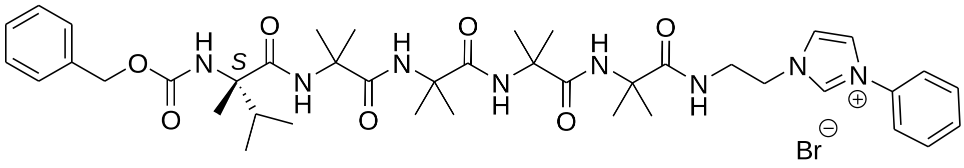

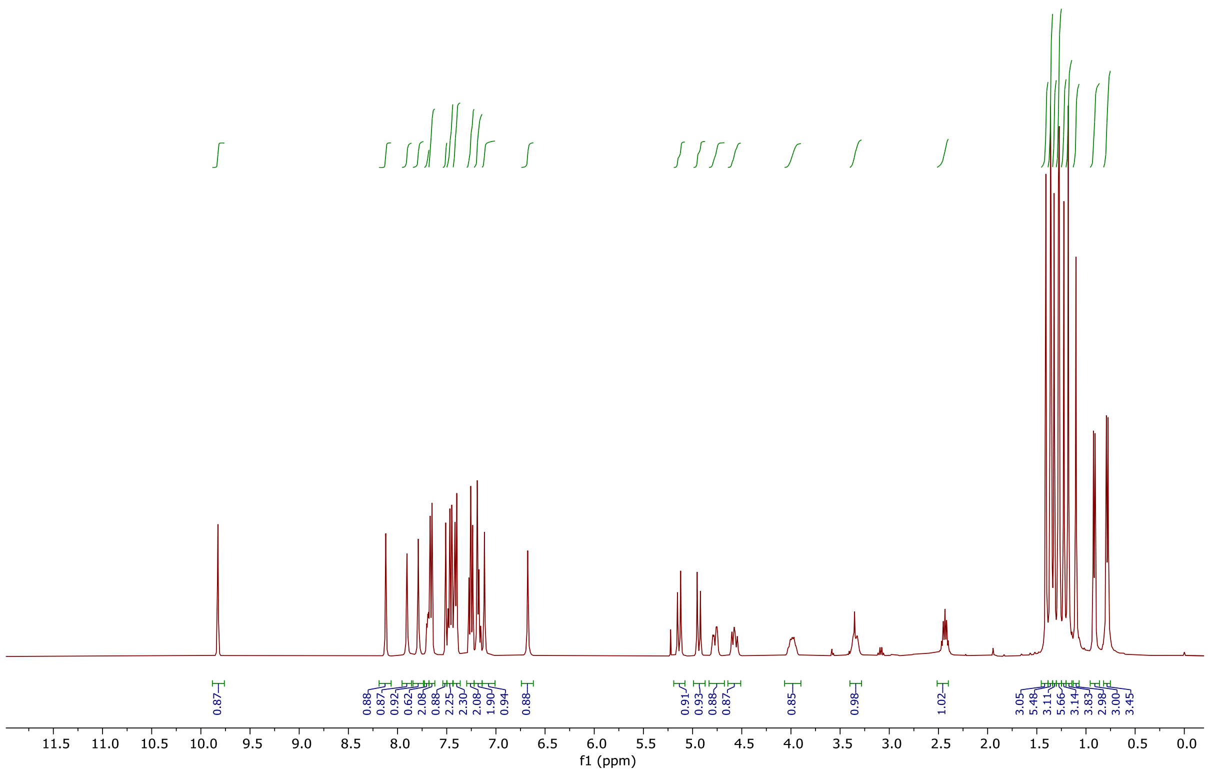

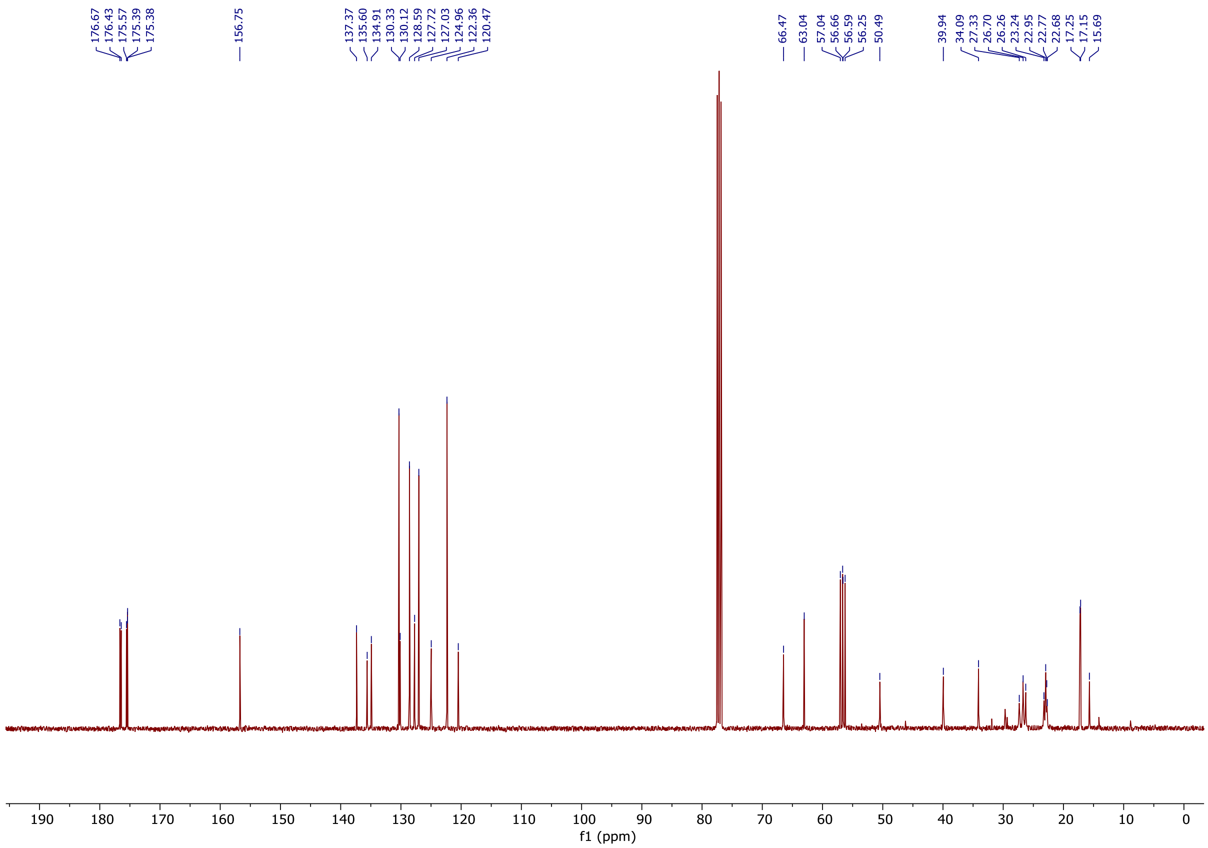
176.67
176.43
175.57
175.39
175.38
156.75
137.37
135.60
134.91
130.33
130.12
128.59
127.72
127.03
124.96
122.36
120.47
66.47
63.04
57.04
56.66
56.59
56.25
50.49
39.94
34.09
27.33
26.70
26.26
23.24
22.95
22.77
22.68
17.25
17.15
15.69
f1 (ppm)

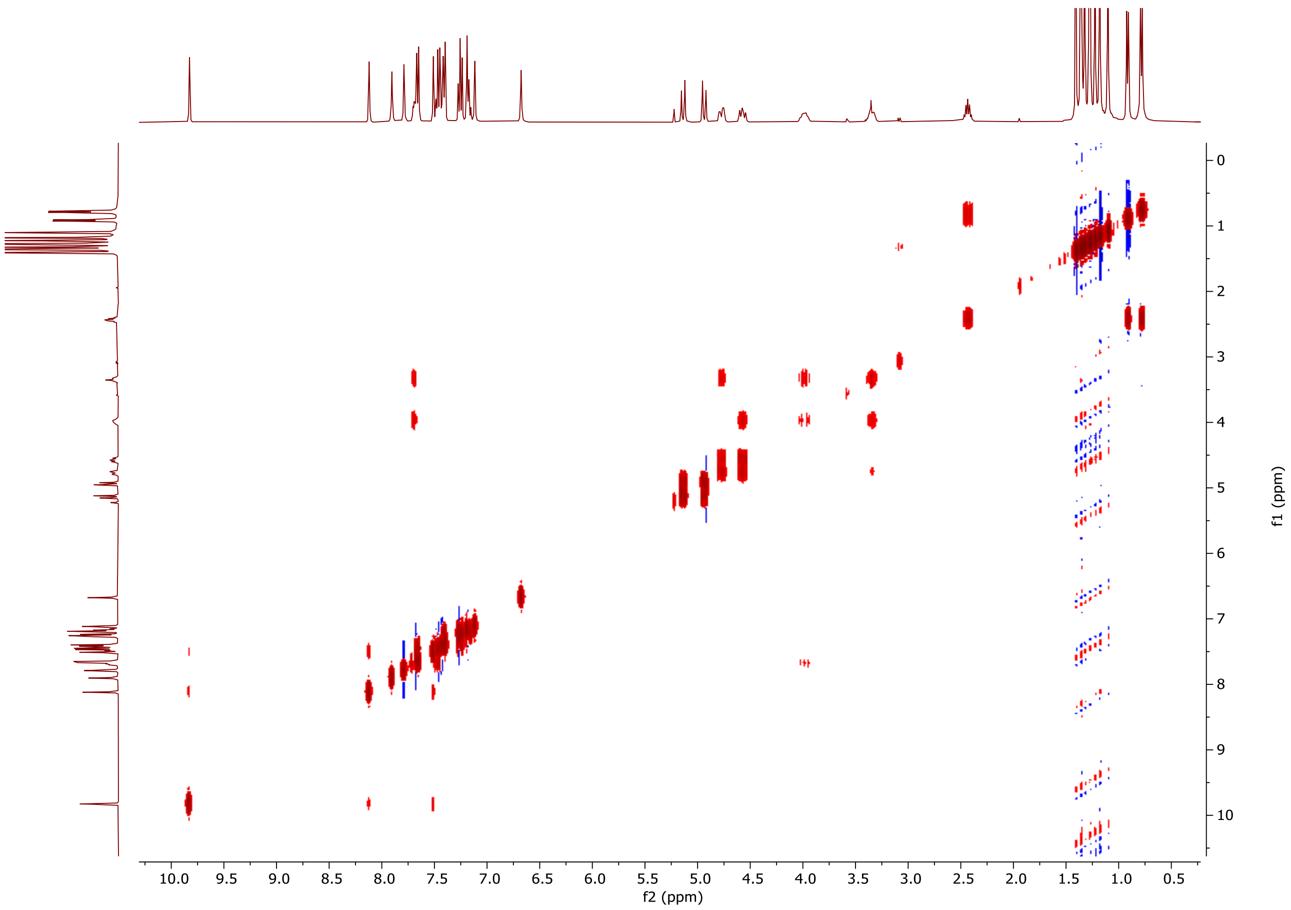
f2 (ppm)
f1 (ppm)

^{1}H ^{13}C HSQC

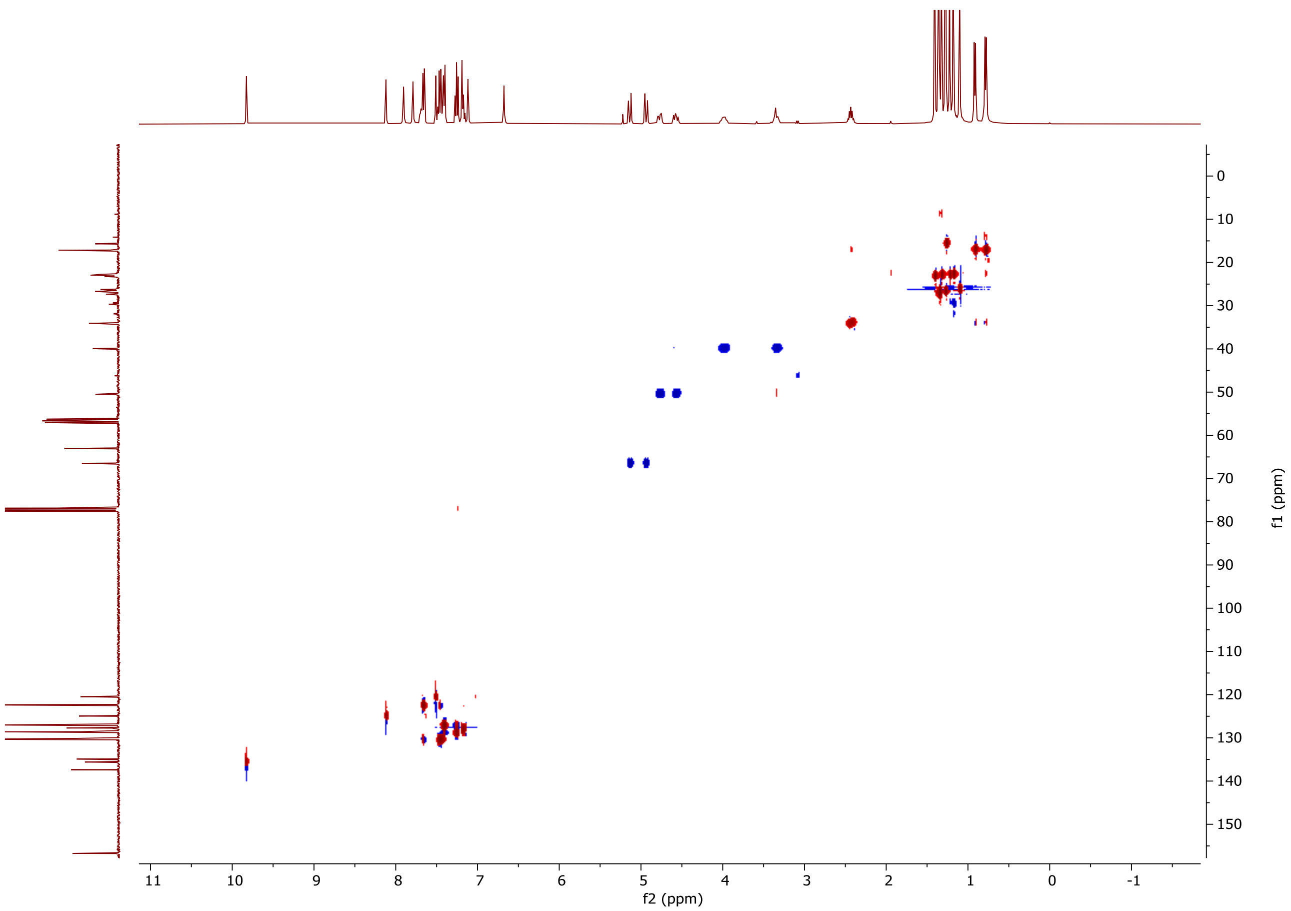

^{1}H ^{13}C HMBC

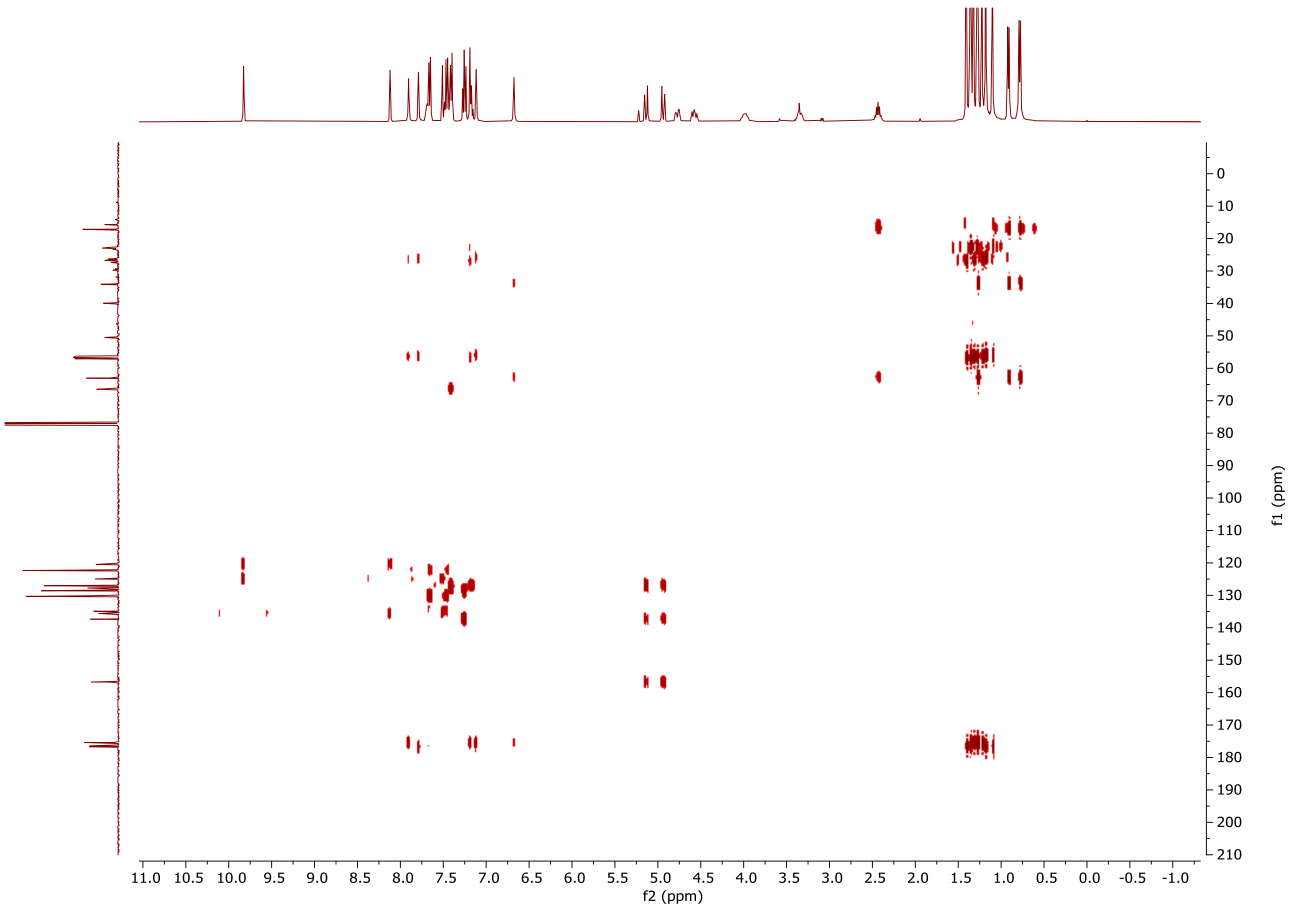

4.15 Foldamer 14 [(Cbz-(L-αMeVal)Aib4NH(CH2)2-NHC-Ph)Rh(Cl)(COD)]

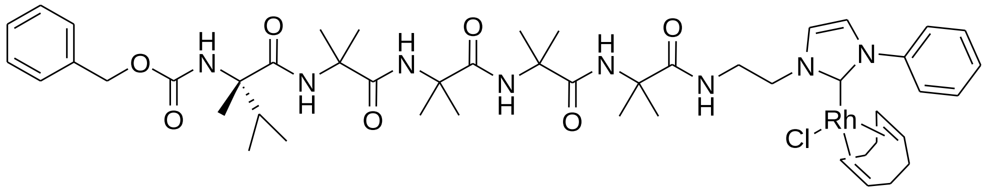

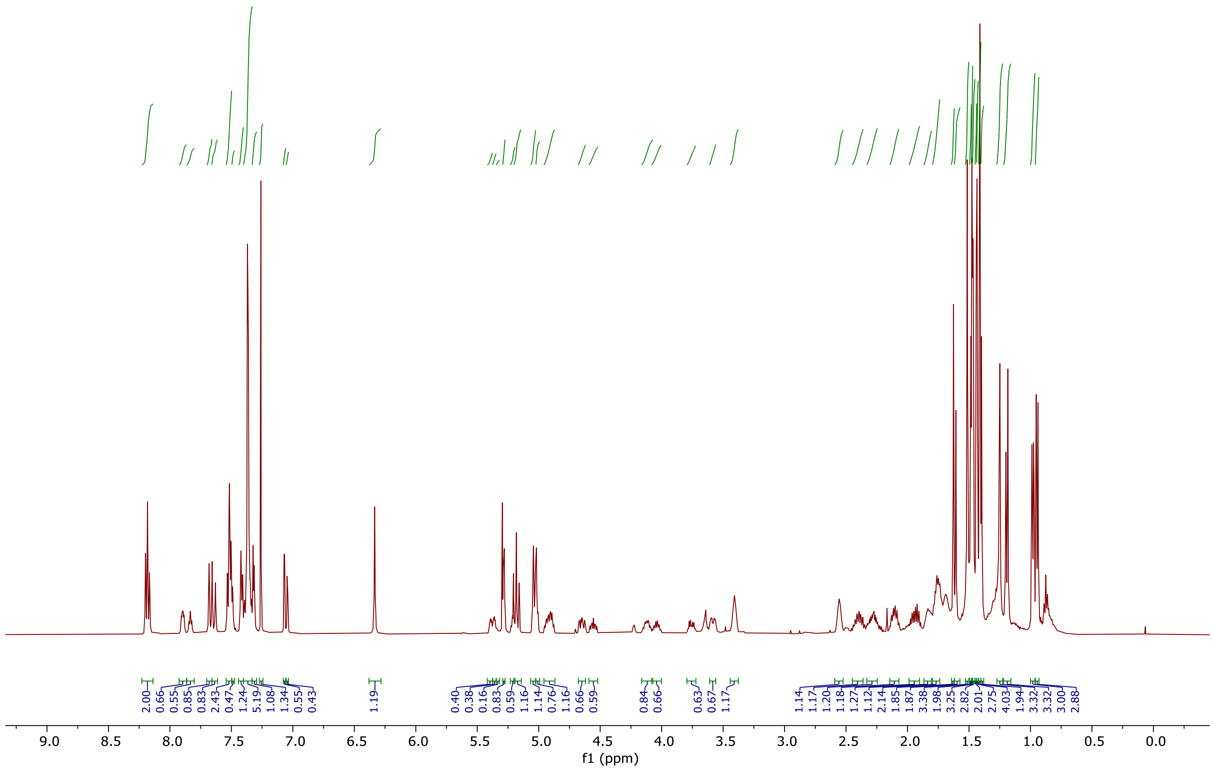

Line fitting of the CH_{Imid} and CH_2-imid signals in the ^{1}H NMR spectrum

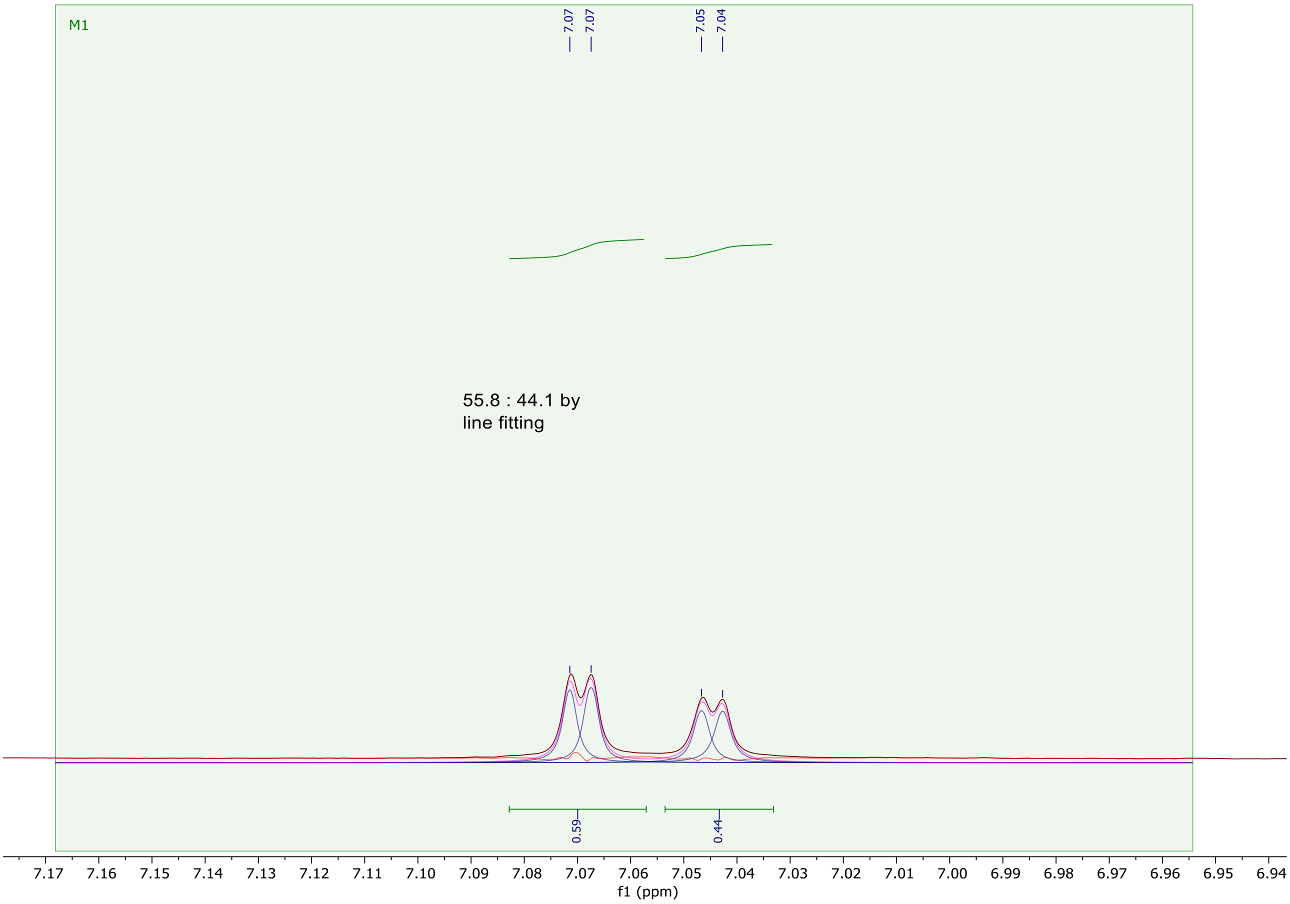

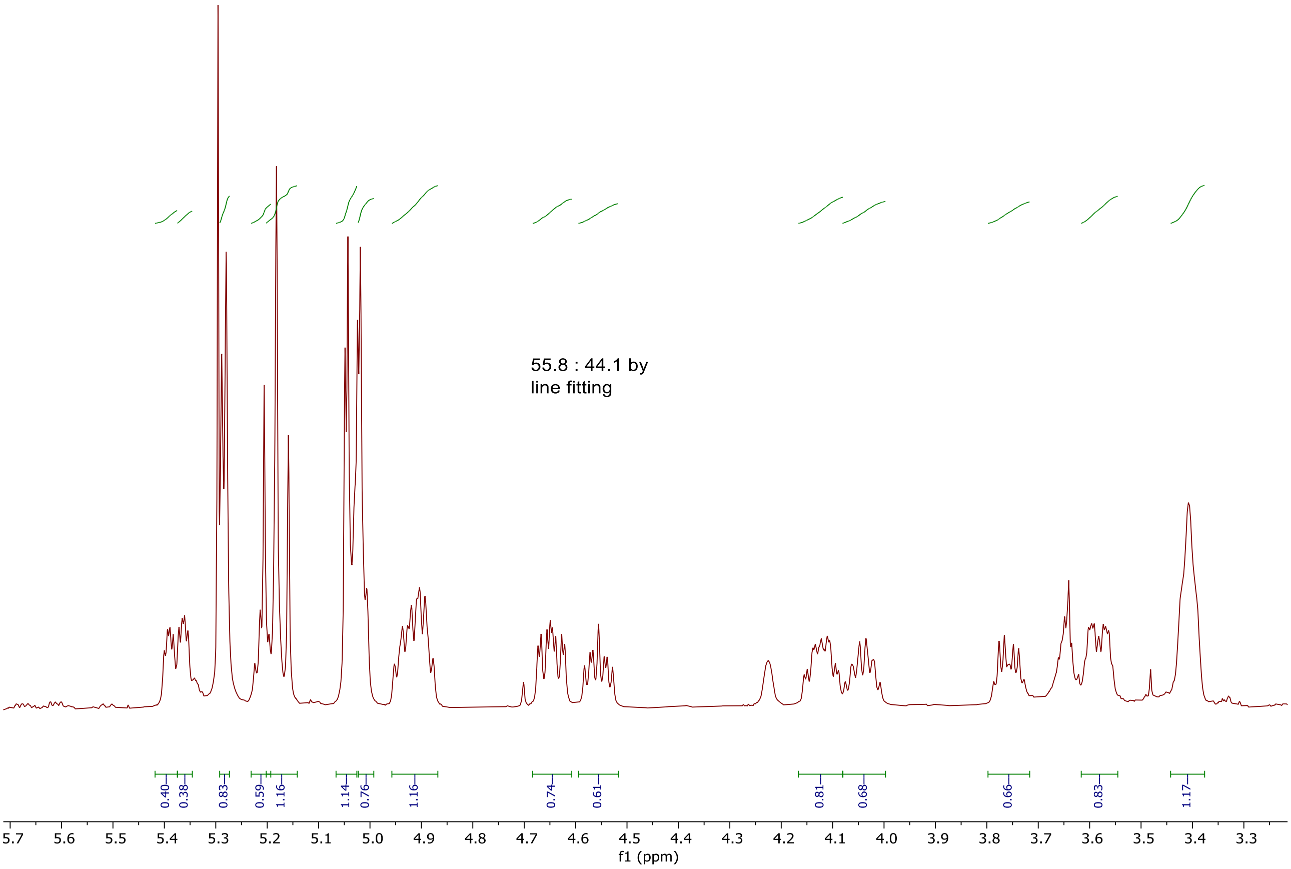

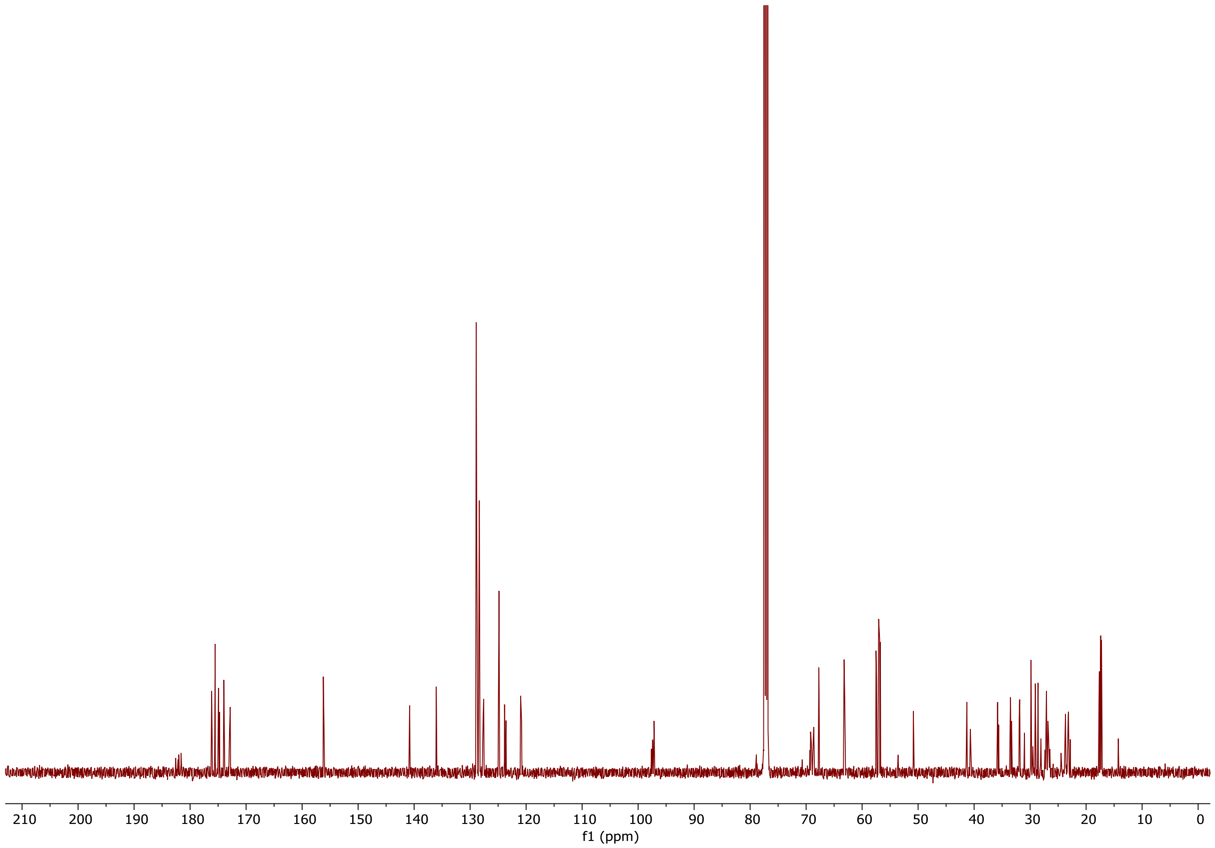
f1 (ppm)

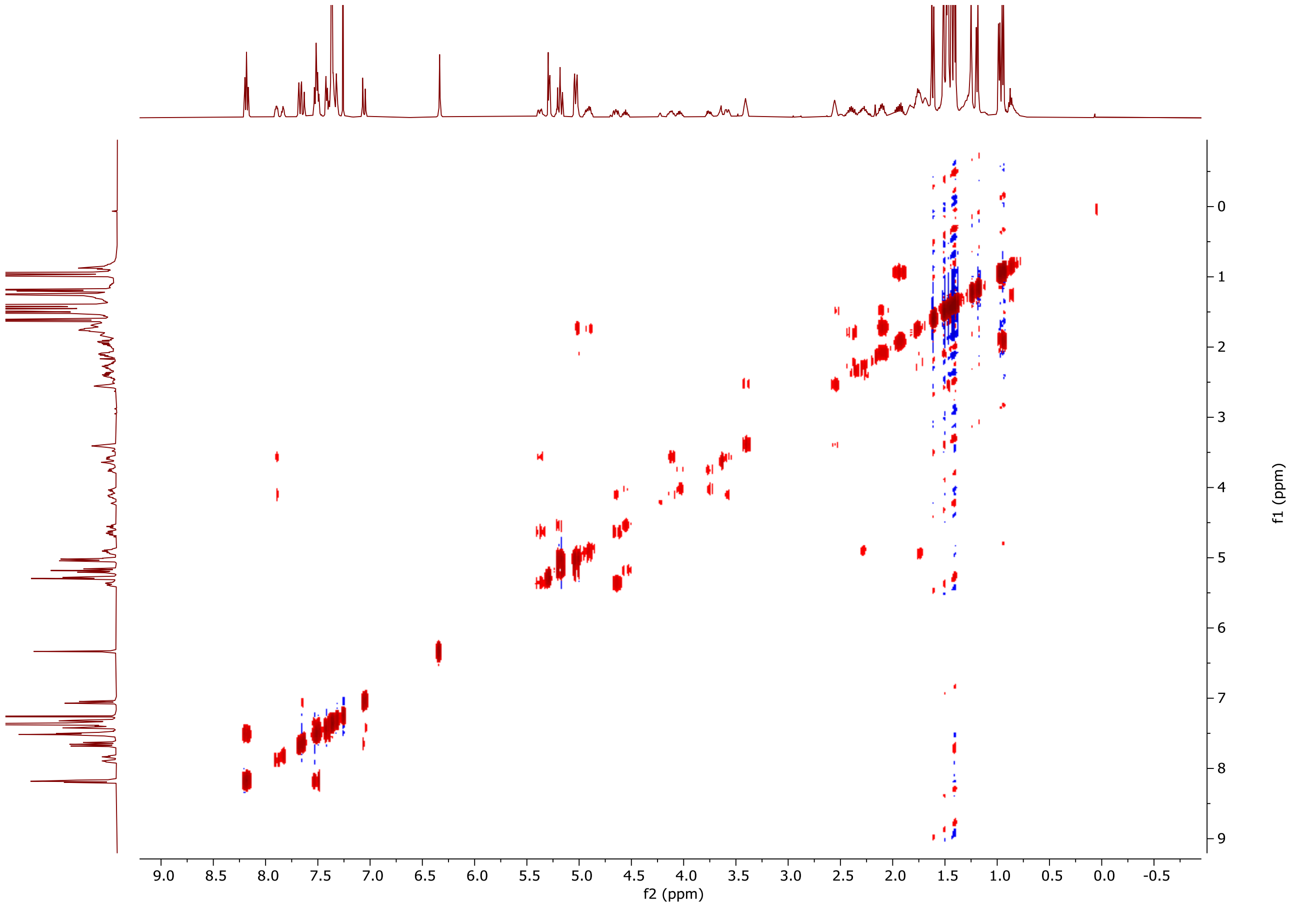
f2 (ppm)
f1 (ppm)

^{1}H ^{13}C HSQC

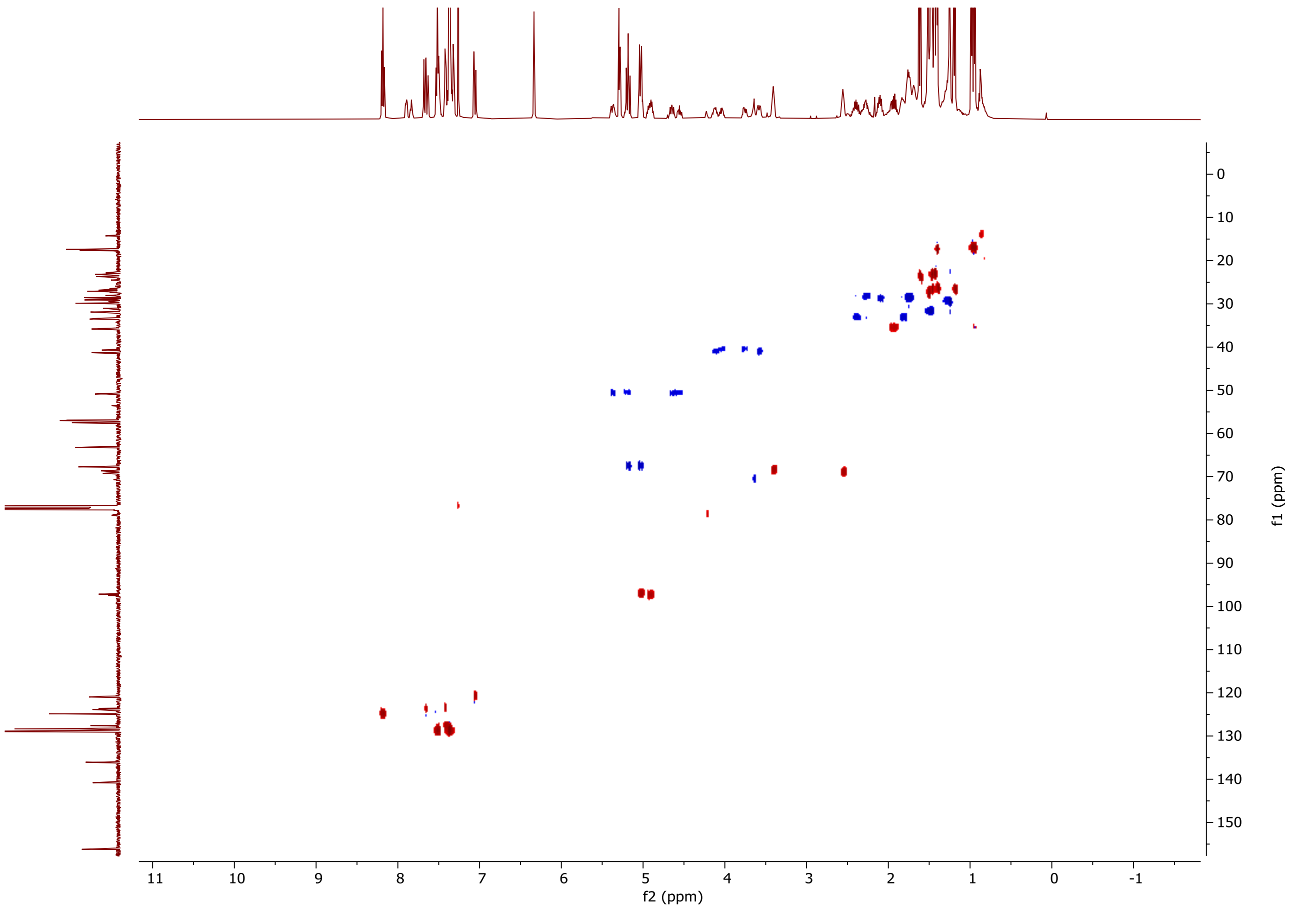

0
10
20
30
40
50
60
70
80
90
100
110
120
130
140
150
f1 (ppm)
11
10
9
8
7
6
5
4
3
2
1
0
-1
f2 (ppm)

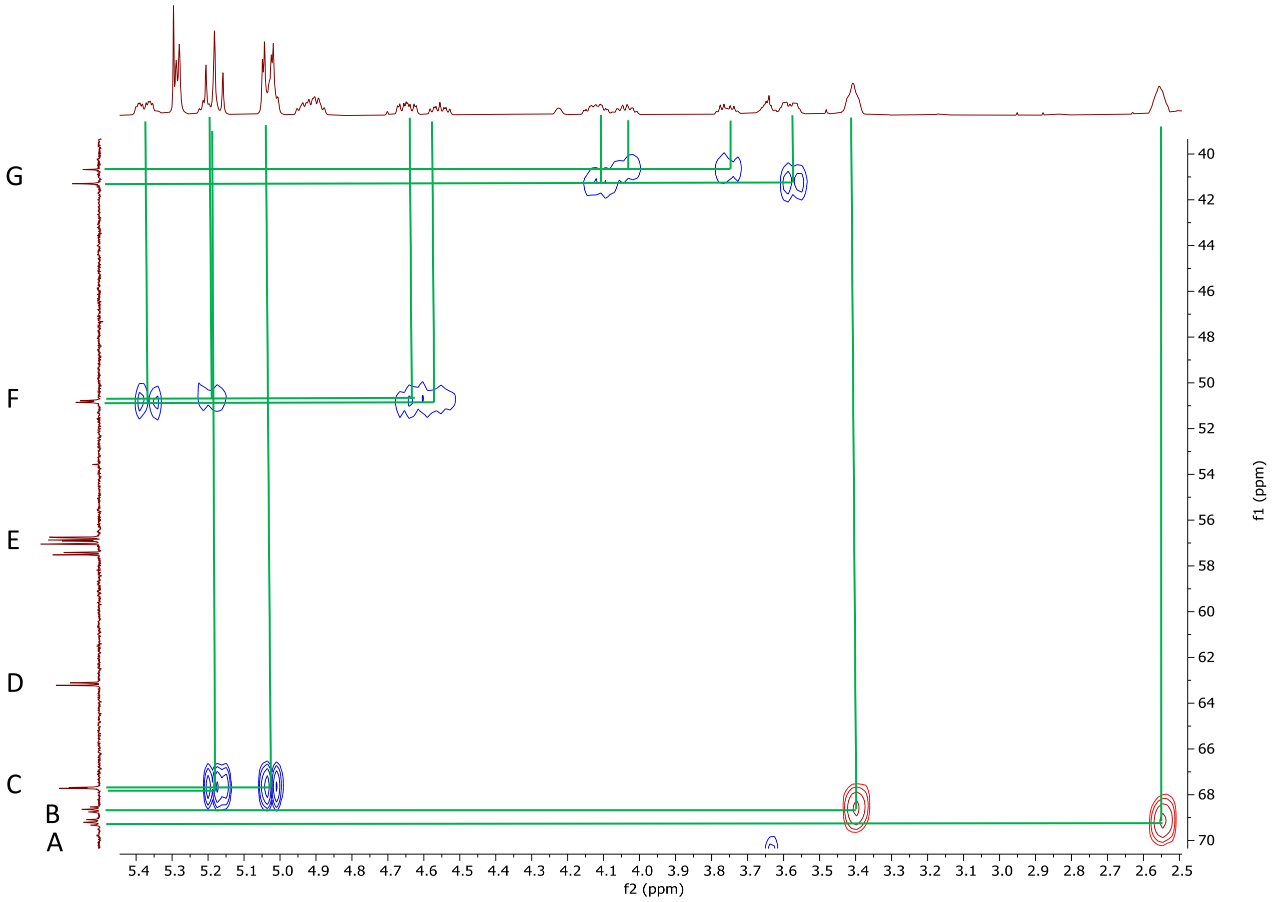

Expansion of the HSQC spectrum to show the distinct ^{1}H and ^{13}C resonances from the two diastereoisomers. Cross peaks highlighed with green lines. Assignments: A: 69.3 (d, *J* = 15.6 Hz, *C*H=CH$_{cod\ dia}$), 69.2 (d, *J* = 15.3 Hz, *C*H=CH$_{cod\ dia}$); B: 68.7 (d, *J* = 14.1 Hz, *C*H=CH$_{cod\ dia}$), 68.6 (d, *J* = 13.2 Hz, *C*H=CH$_{cod\ dia}$); C: 67.7 (*C*H$_2$Ph$_{dia}$), 67.7 (*C*H$_2$Ph$_{dia}$); D: 63.2 (*C*$_{aMeVal}$CH$_{3\ dia\ 1}$), 63.1 (*C*$_{aMeVal}$CH$_{3\ dia\ 2}$); E: 57.5 (*C*(CH$_3$)$_{2\ dia\ 1}$), 57.4 (*C*(CH$_3$)$_{2\ dia\ 2}$), 57.0 (*C*(CH$_3$)$_{2\ dia\ 1}$), 57.0 (*C*(CH$_3$)$_{2\ dia\ 2}$), 56.9 (*C*(CH$_3$)$_{2\ dia\ 2}$), 56.9 (*C*(CH$_3$)$_{2\ dia\ 1}$), 56.8 (*C*(CH$_3$)$_{2\ dia2}$), 56.7 (*C*(CH$_3$)$_{2\ dia\ 1}$); F: 50.9 (*C*H$_2$Imid$_{\ dia\ 1}$), 50.8 (*C*H$_2$Imid$_{\ dia\ 2}$); G: 41.3 (*C*H$_2$NH$_{\ dia1}$), 40.7 (*C*H$_2$NH$_{\ dia2}$).

^{1}H ^{13}C HMBC

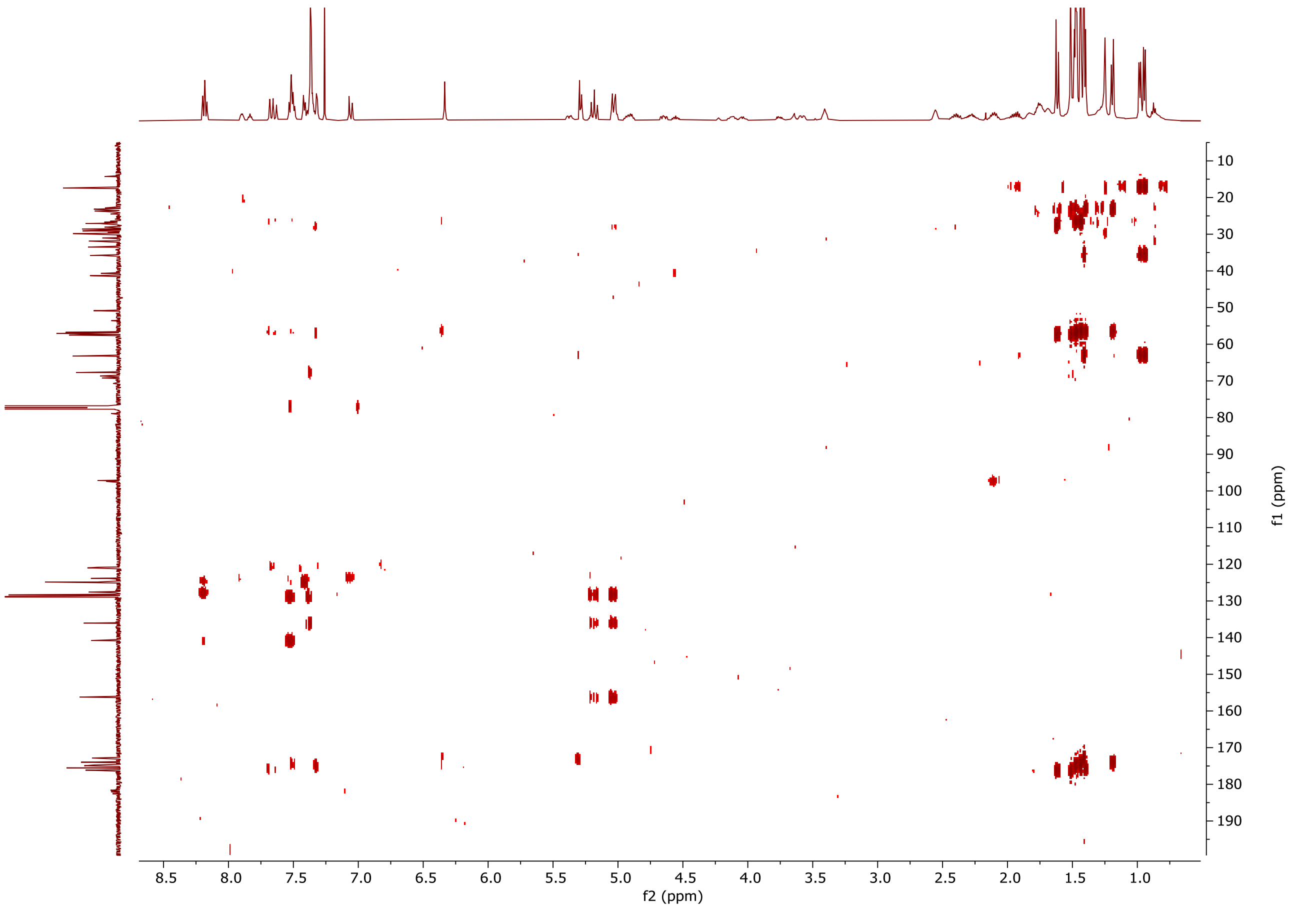

^{1}H ^{1}H NOESY

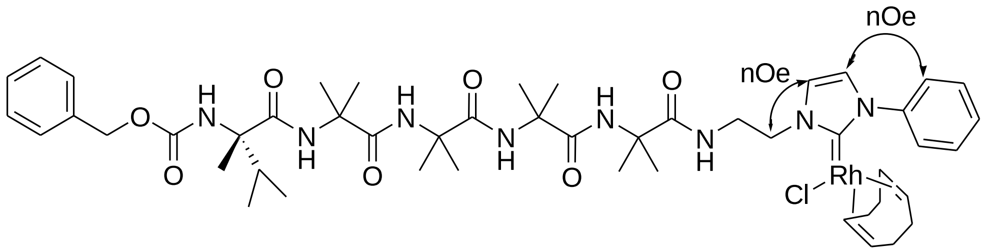

NMR assignments aided by: nOe signals between CH_{imid} (7.06 ppm) and $CH_{Ph\ ortho}$ (8.23 – 8.14 ppm), between CH_{imid} (7.64 ppm) and CH_2-Imid (4.56 ppm).

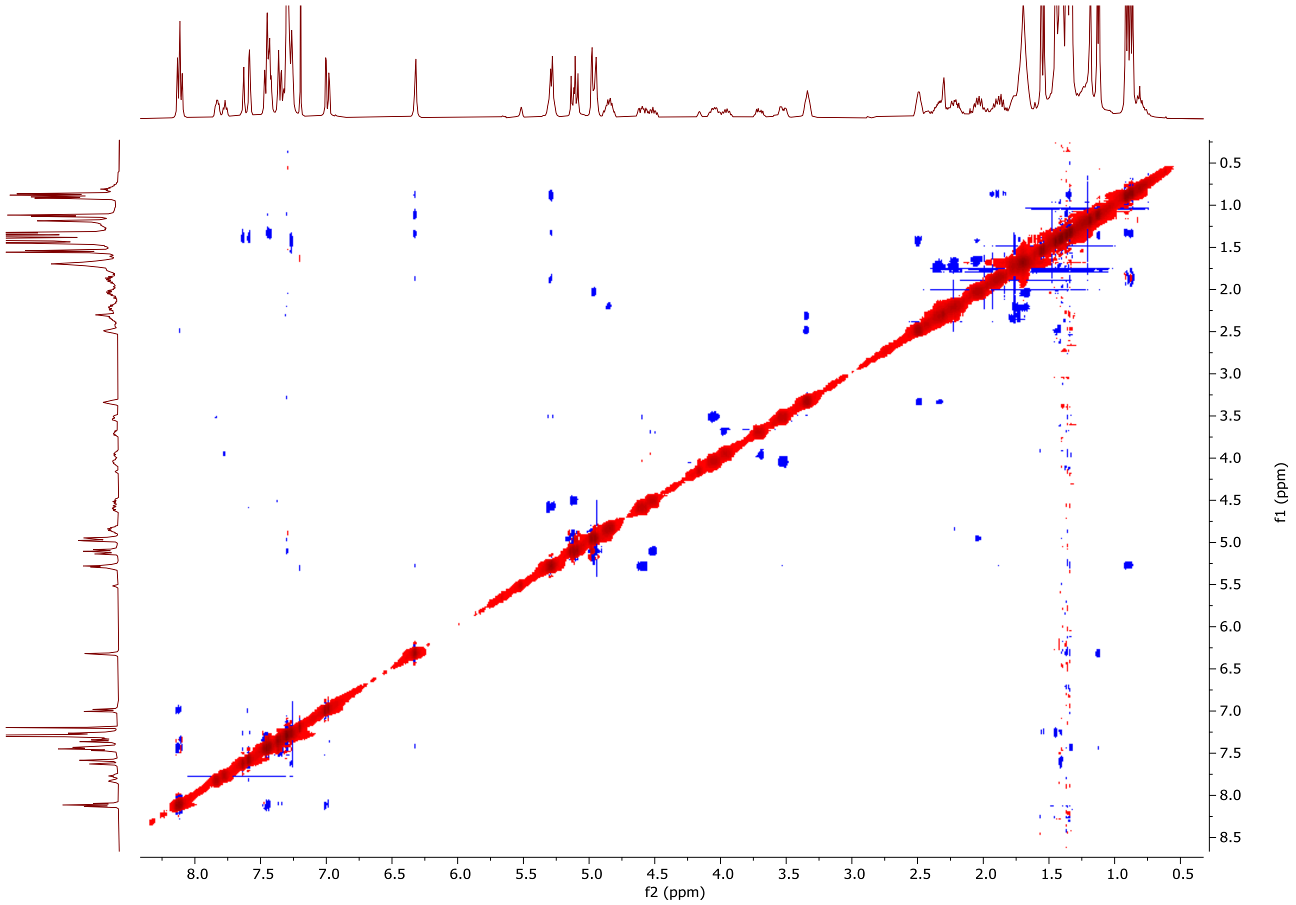
8.0
7.5
7.0
6.5
6.0
5.5
5.0
4.5
4.0
3.5
3.0
2.5
2.0
1.5
1.0
0.5
f2 (ppm)
f1 (ppm)

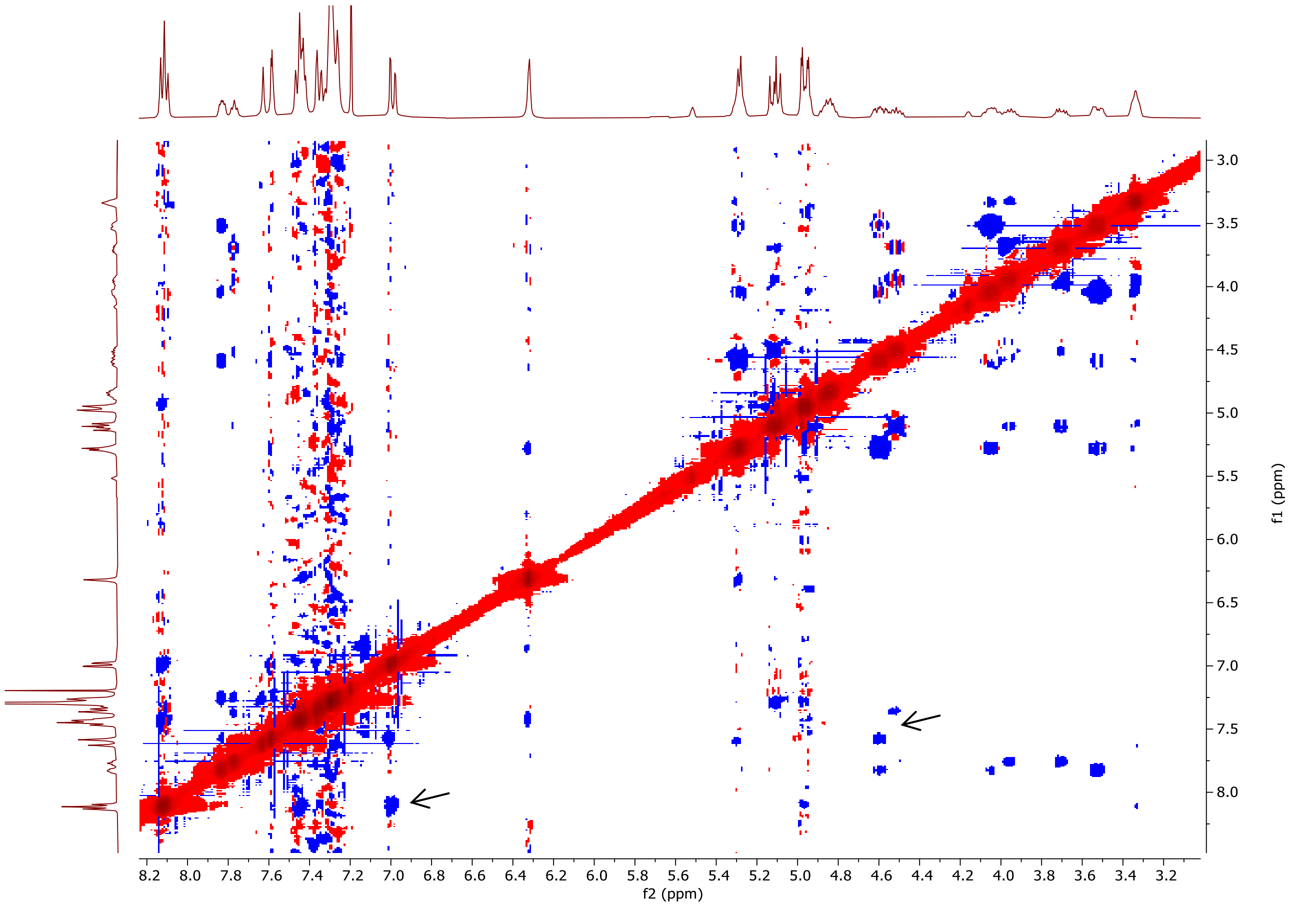
8.2
8.0
7.8
7.6
7.4
7.2
7.0
6.8
6.6
6.4
6.2
6.0
5.8
5.6
5.4
5.2
5.0
4.8
4.6
4.4
4.2
4.0
3.8
3.6
3.4
3.2
f2 (ppm)
3.0
3.5
4.0
4.5
5.0
5.5
6.0
6.5
7.0
7.5
8.0
f1 (ppm)

Irradiation of ^{1}H frequency of CH_{imid} revealing nOe correlation signal with ^{1}H signal of Ph_{ortho}

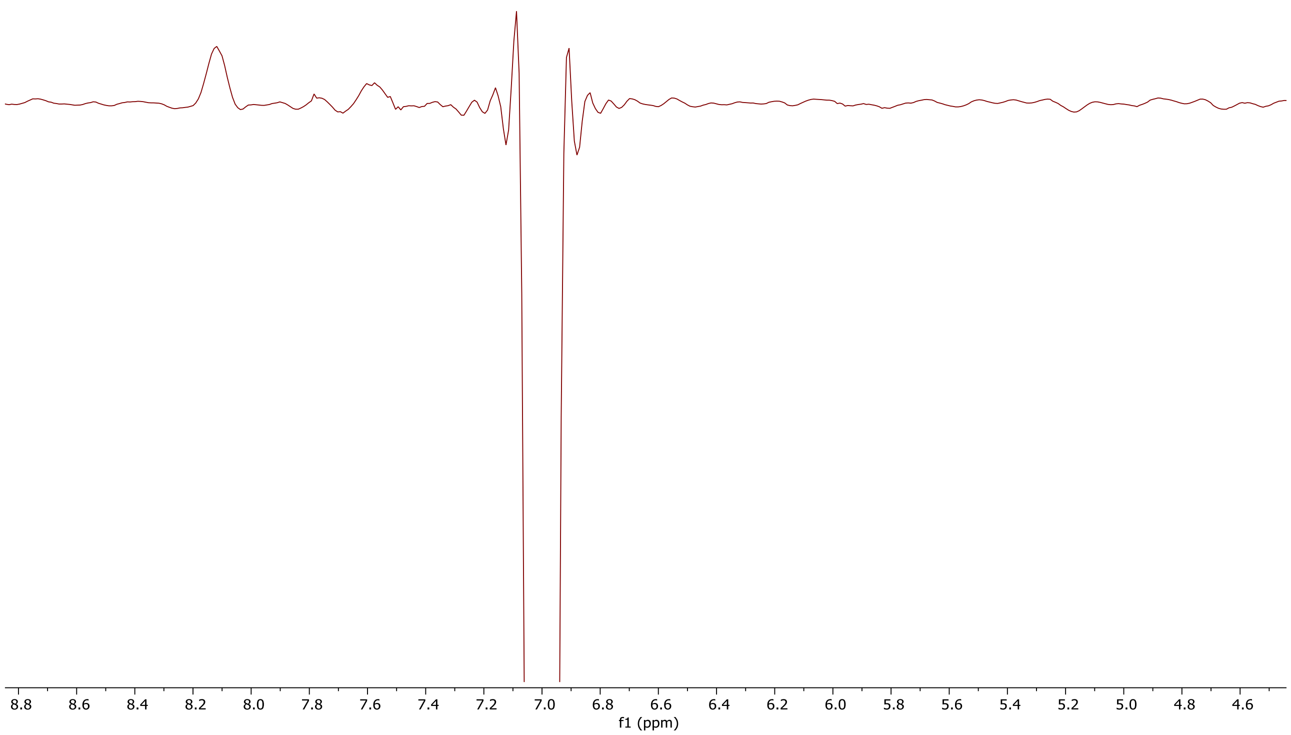

4.16 Foldamer 15 [(Cbz-(L-αMeVal)Aib$_4$NH(CH$_2$)$_2$-NHC-Ph)Rh(Cl)(NBD)]

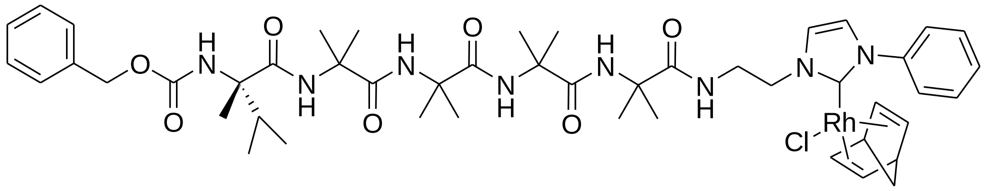

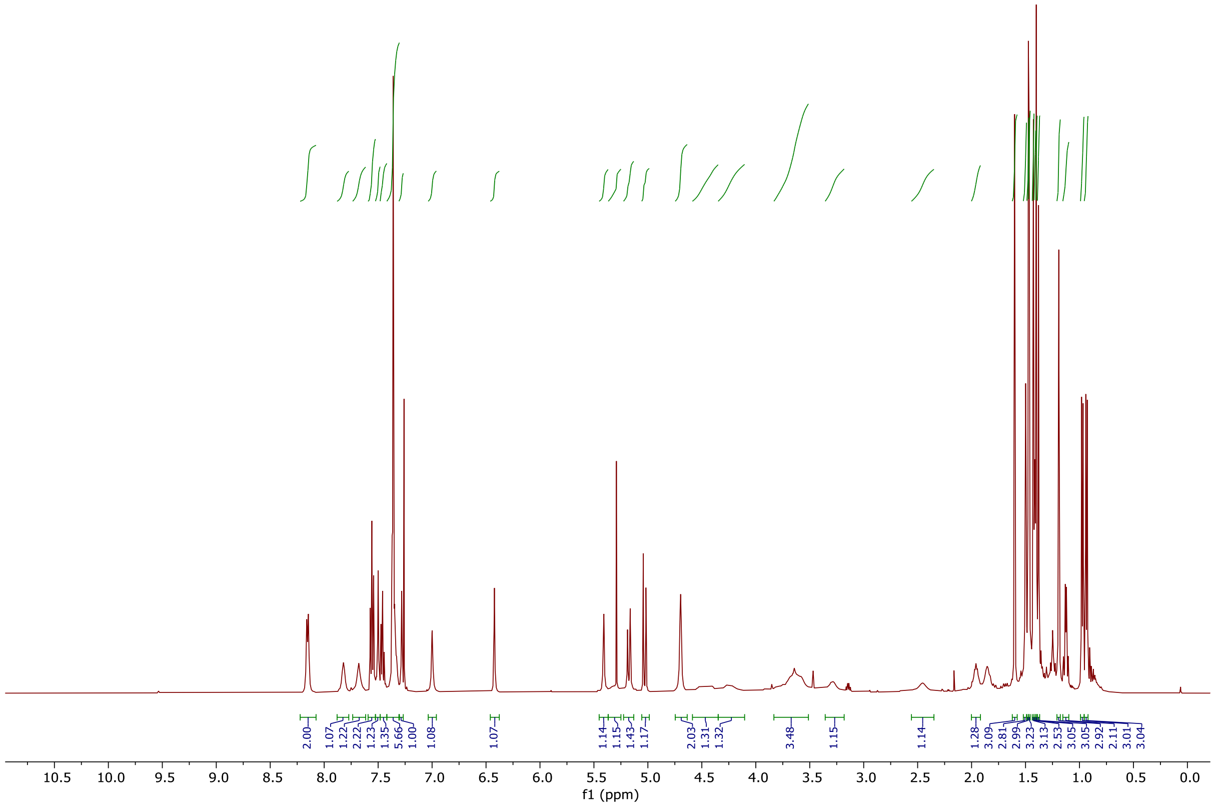

Expansion of the ^{1}H NMR spectrum showing the N*H*CH$_2$ and C*H*$_2$Ph signals.

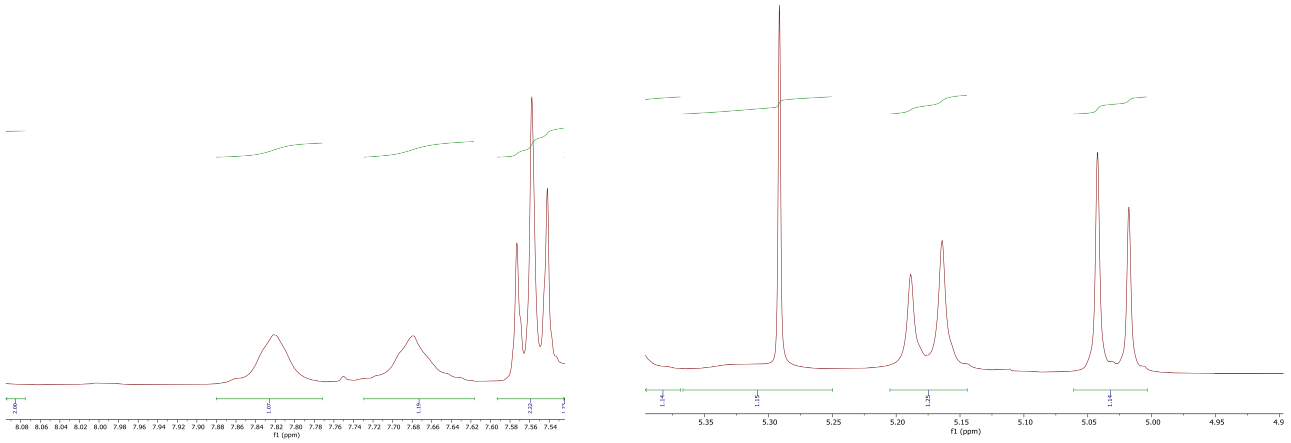

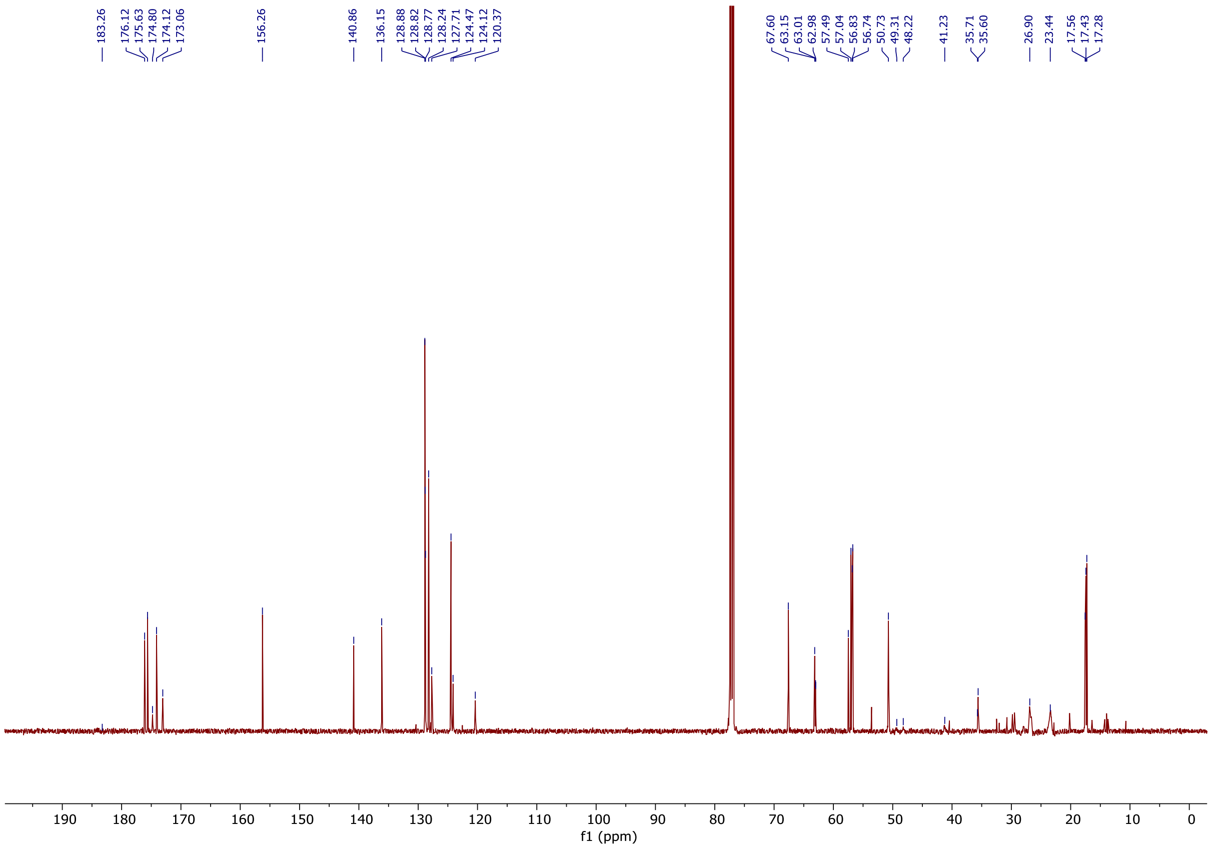
183.26
176.12
175.63
174.80
174.12
173.06
156.26
140.86
136.15
128.88
128.82
128.77
128.24
127.71
124.47
124.12
120.37
67.60
63.15
63.01
62.98
57.49
57.04
56.83
56.74
50.73
49.31
48.22
41.23
35.71
35.60
26.90
23.44
17.56
17.43
17.28
190 180 170 160 150 140 130 120 110 100 90 80 70 60 50 40 30 20 10 0
f1 (ppm)

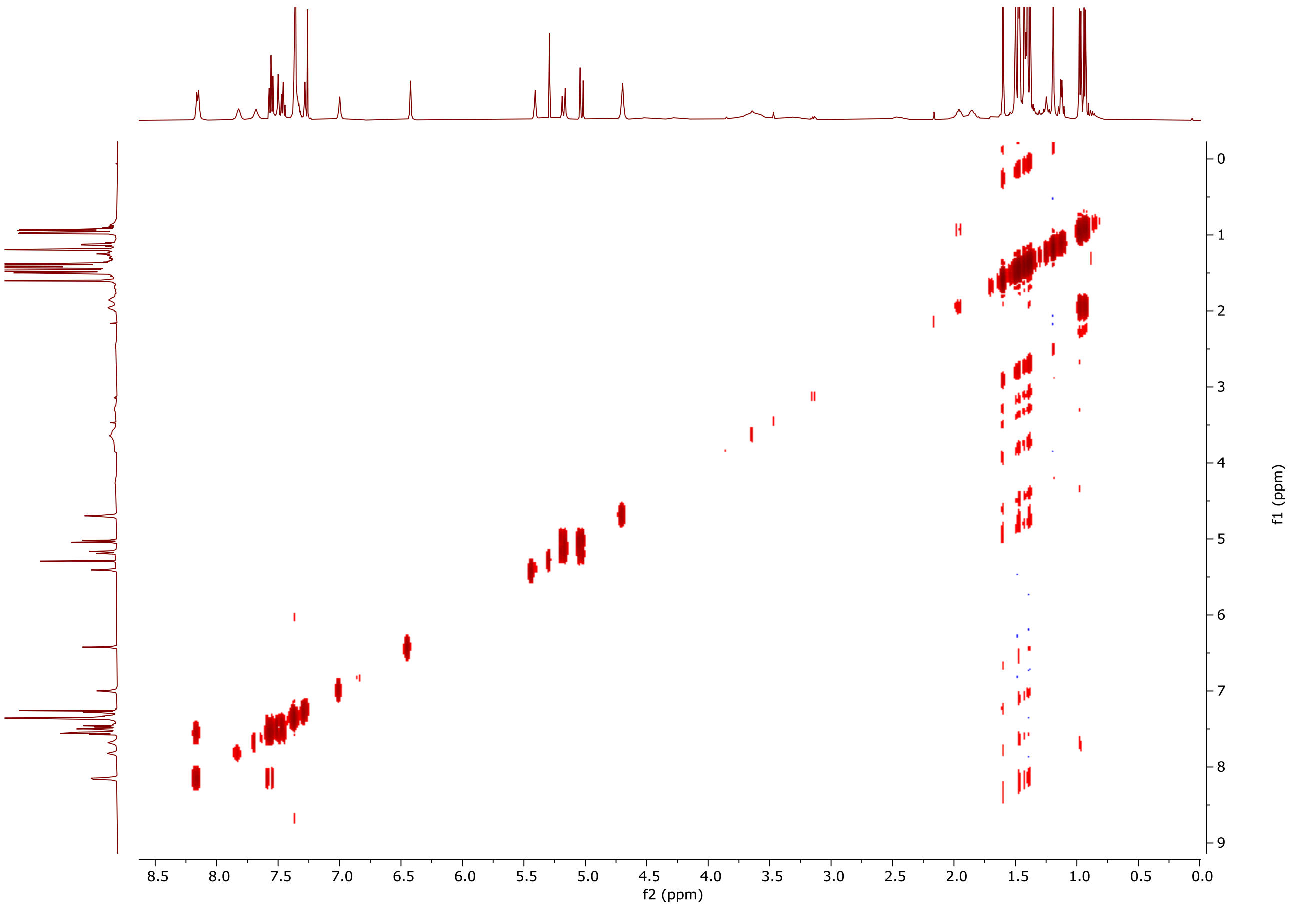
8.5 8.0 7.5 7.0 6.5 6.0 5.5 5.0 4.5 4.0 3.5 3.0 2.5 2.0 1.5 1.0 0.5 0.0
f2 (ppm)
0 1 2 3 4 5 6 7 8 9
f1 (ppm)

^{1}H ^{13}C HSQC

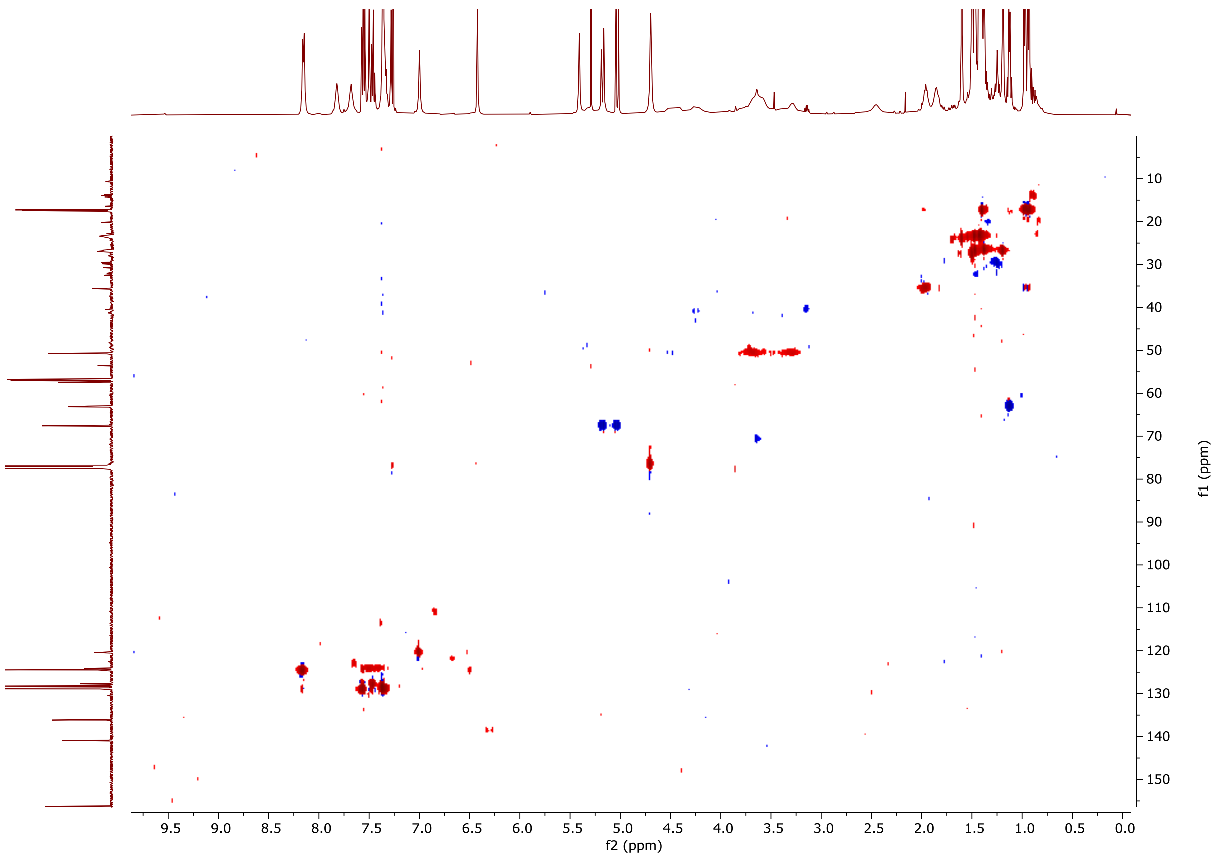

^{1}H ^{13}C HMBC

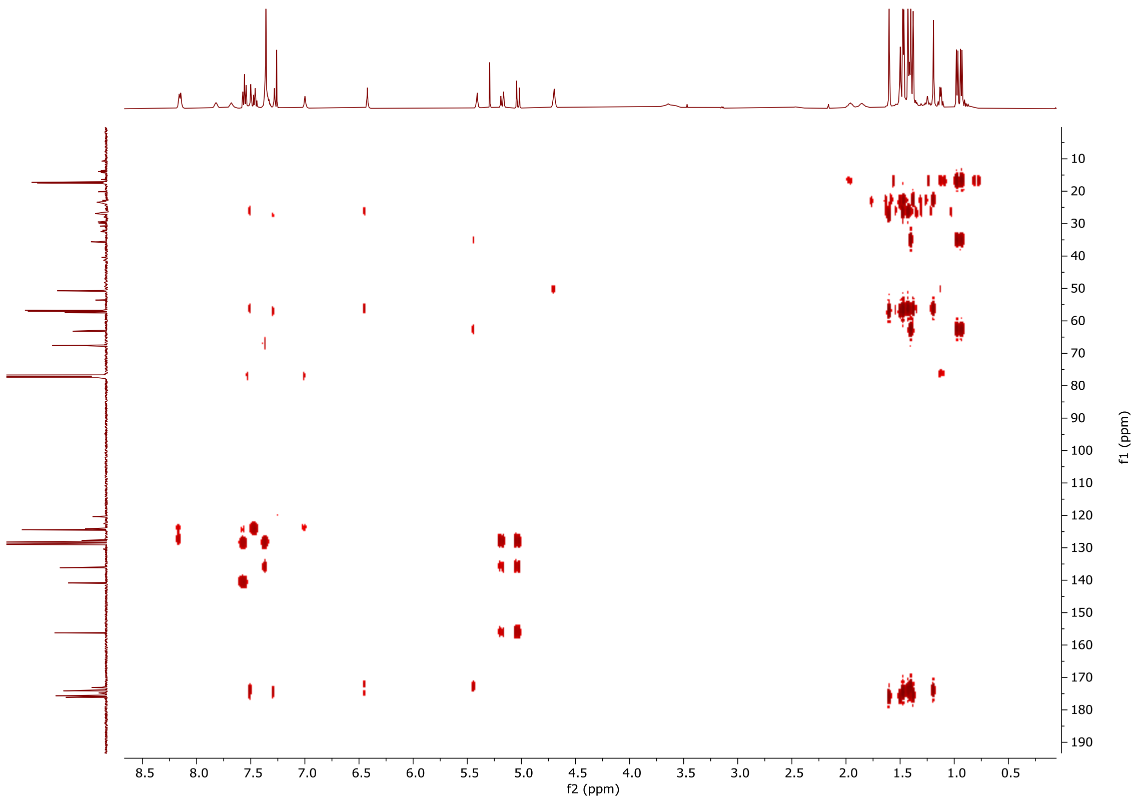

4.17 Precursor 10, [Cbz-(L-αMeVal)$Aib_4NH(CH_2)_2$(Im-Mes)]$^+$Br

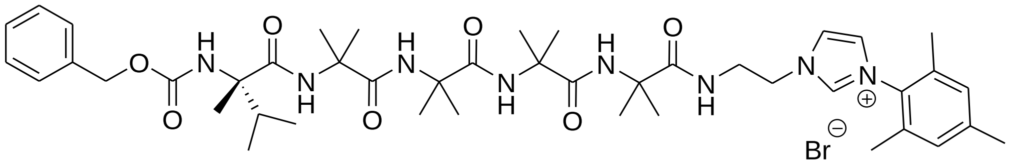

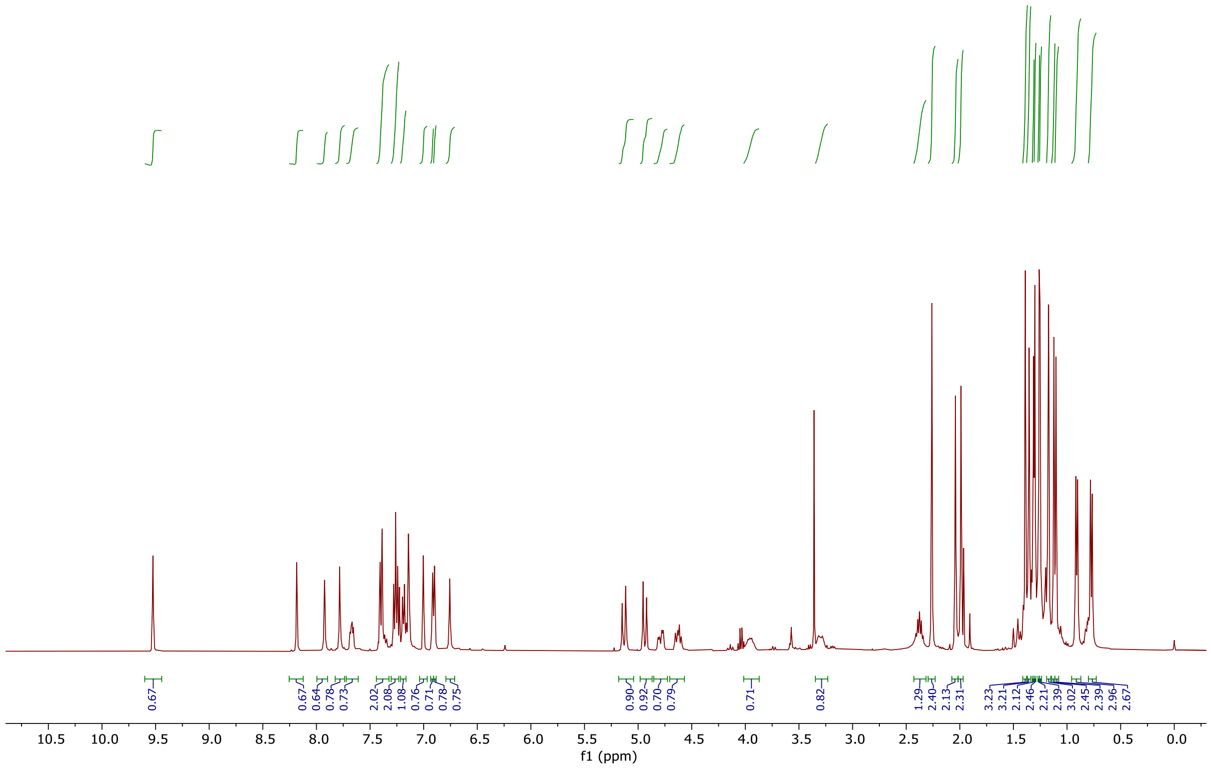

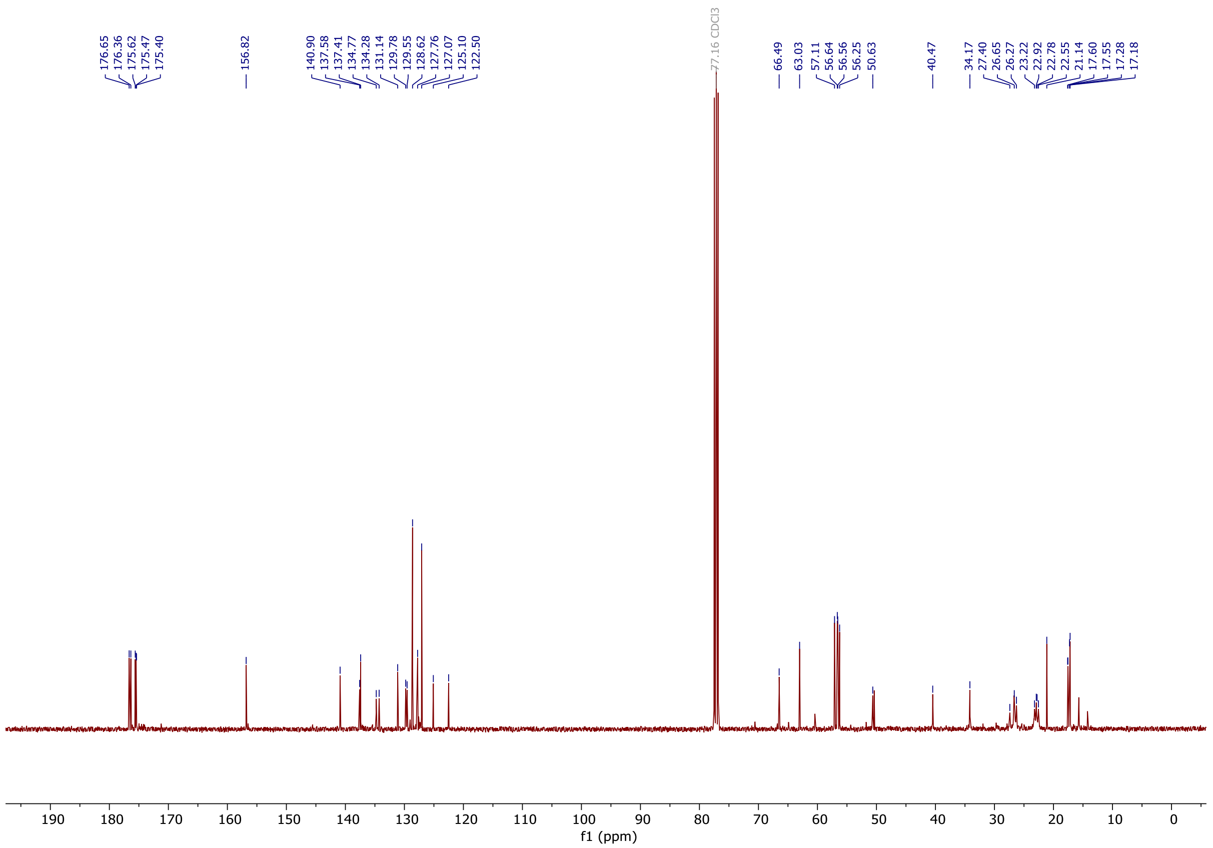
176.65
176.36
175.62
175.47
175.40
156.82
140.90
137.58
137.41
134.77
134.28
131.14
129.78
129.55
128.62
127.76
127.07
125.10
122.50
77.16 CDCl3
66.49
63.03
57.11
56.64
56.56
56.25
50.63
40.47
34.17
27.40
26.65
26.27
23.22
22.92
22.78
22.55
21.14
17.60
17.55
17.28
17.18
f1 (ppm)

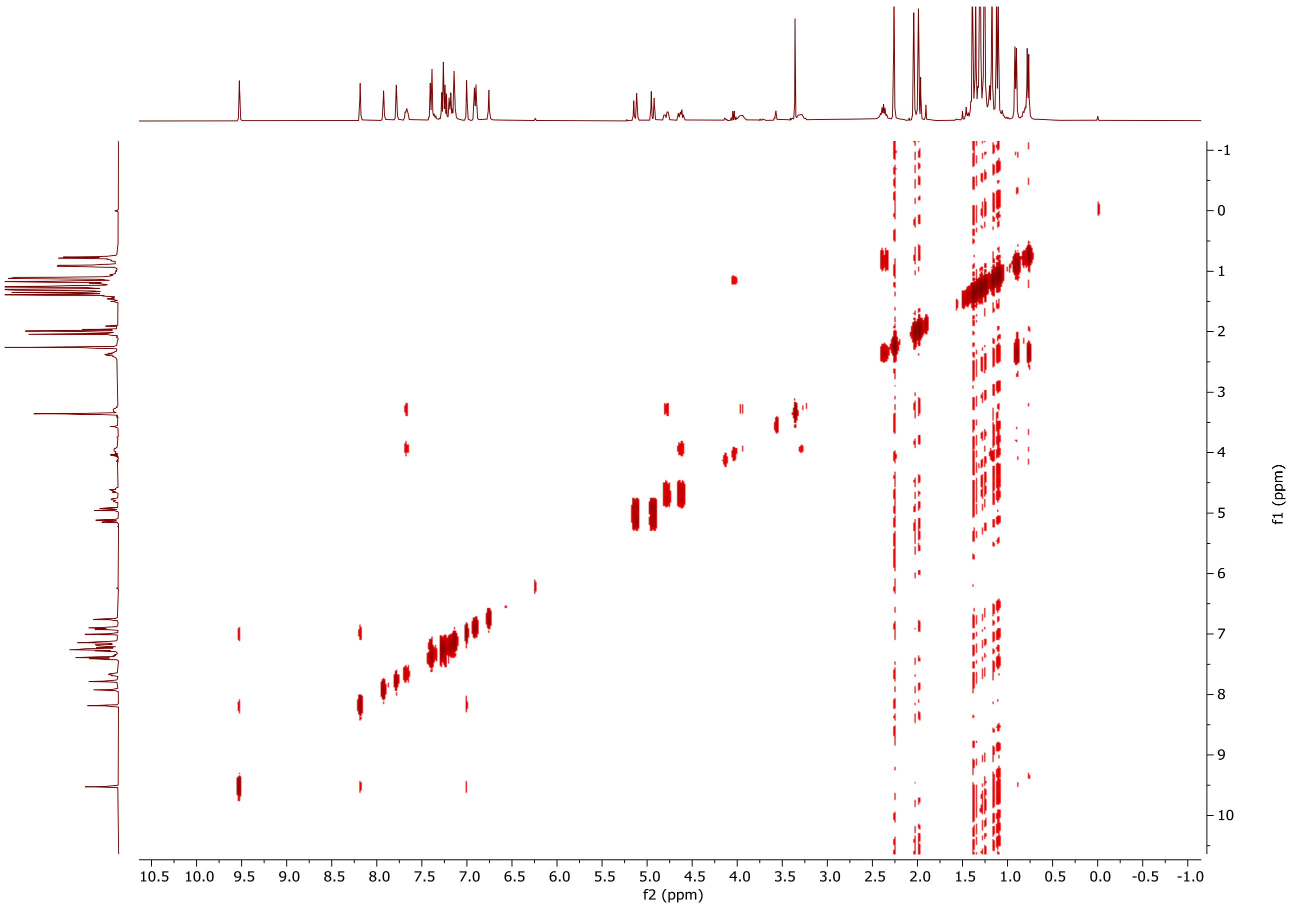
f2 (ppm)
f1 (ppm)

^{1}H ^{13}C HSQC

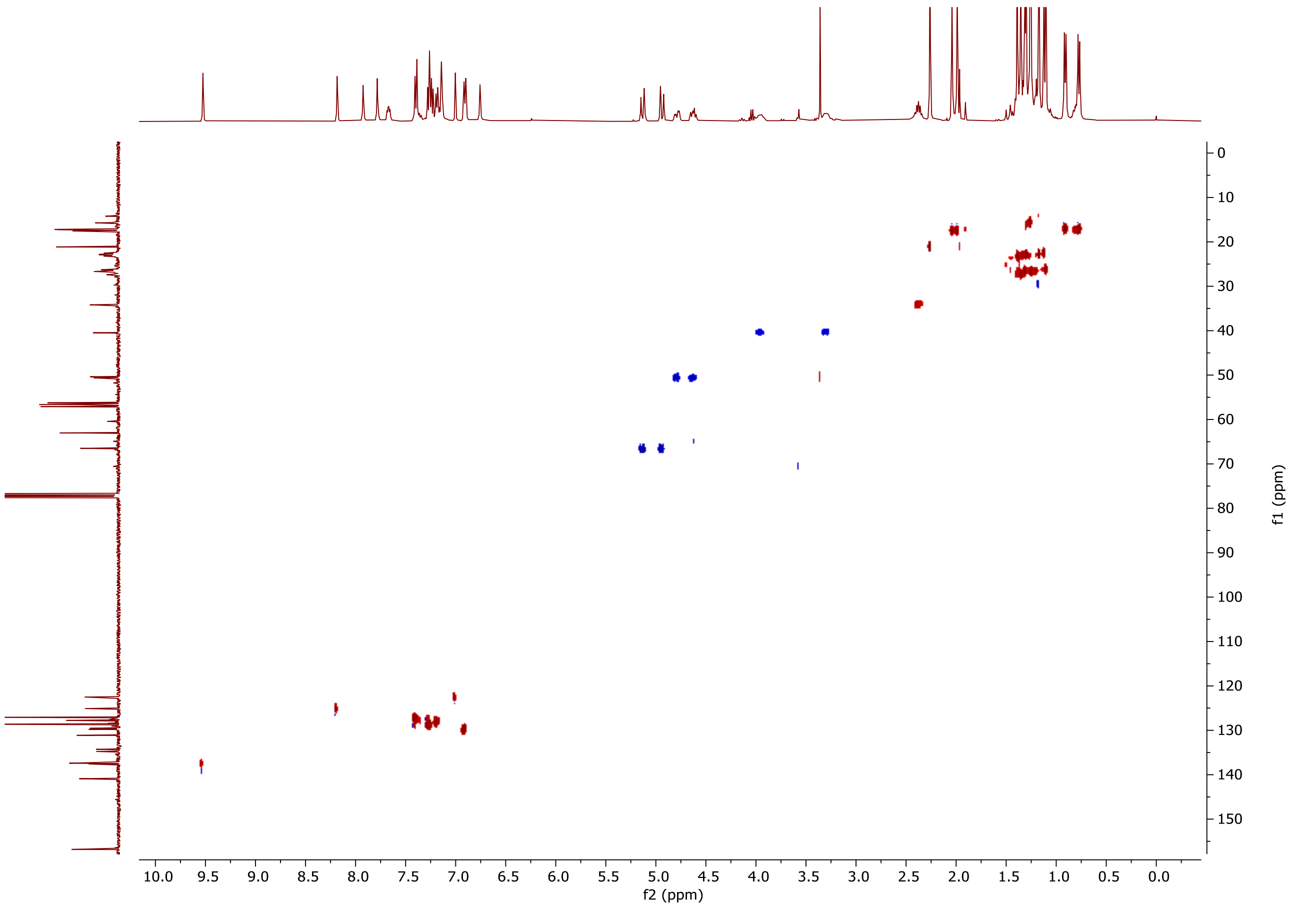

^{1}H ^{13}C HMBC

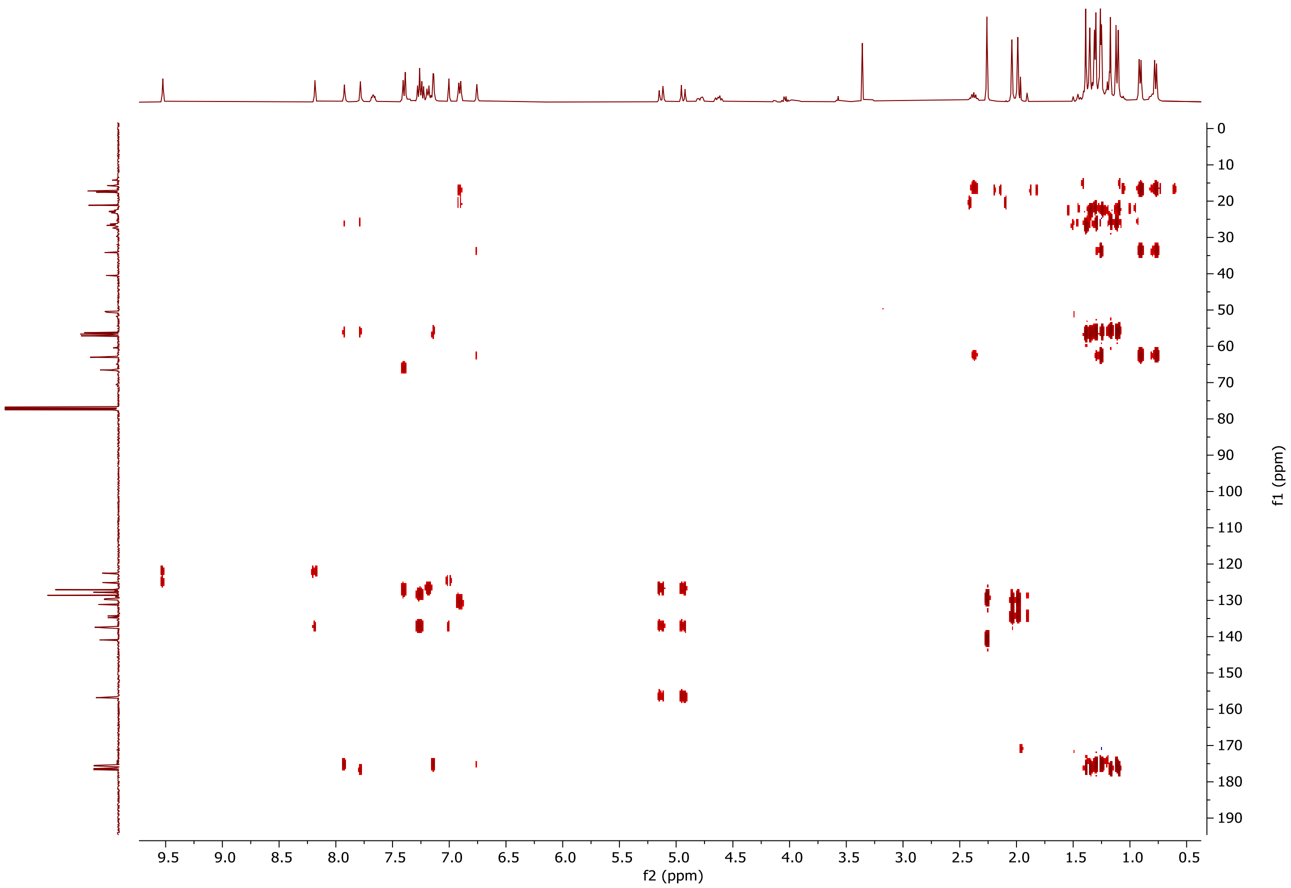

4.18 Foldamer 16, [(Cbz-(L-αMeVal)Aib$_4$NH(CH$_2$)$_2$-NHC-Mes)Rh(Cl)(COD)]

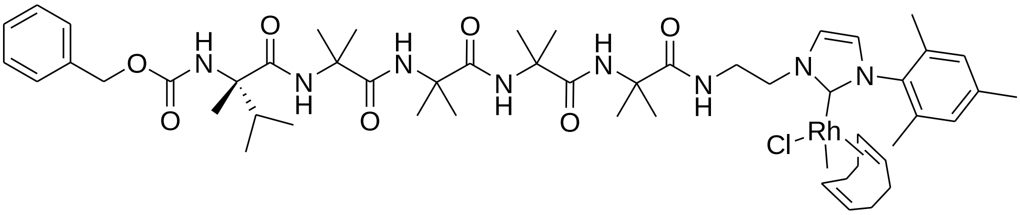

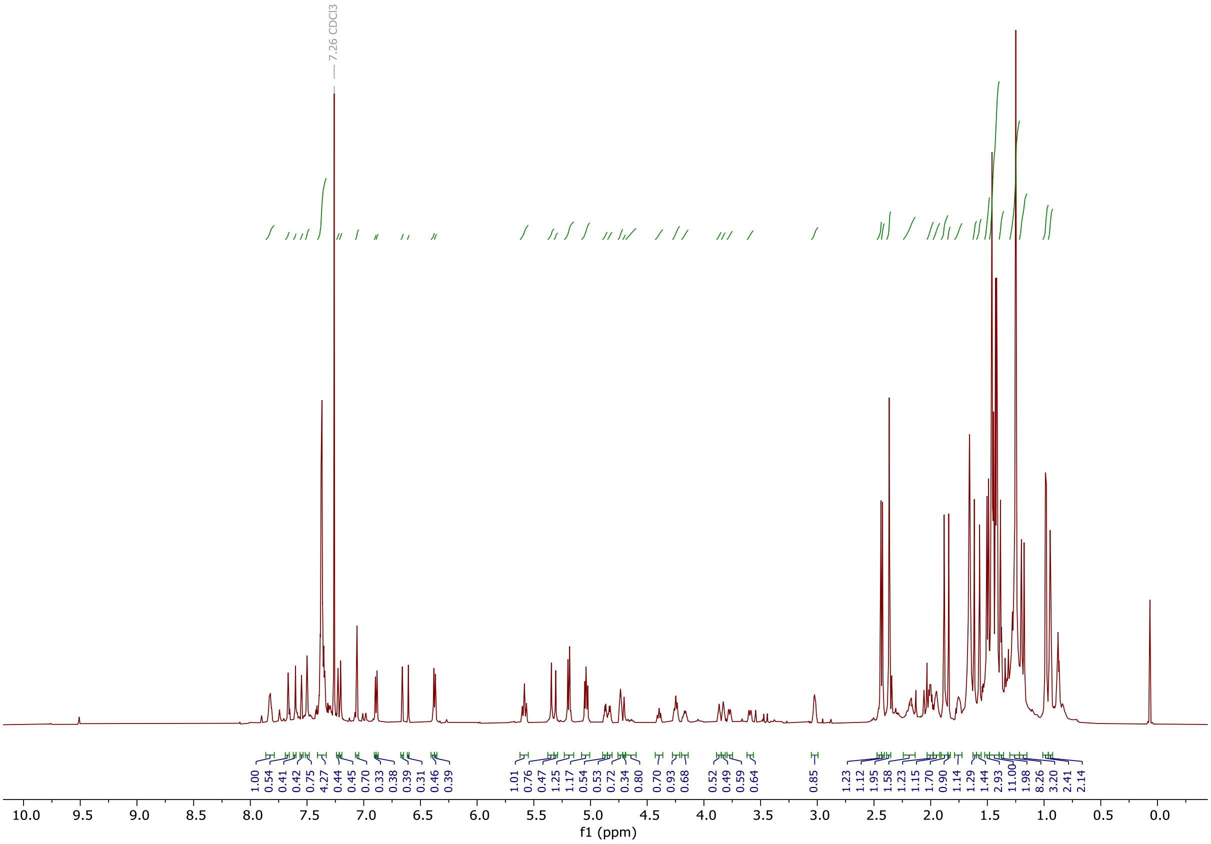

Expansion of ^{1}H NMR spectrum (C$H_{Mesityl}$, CH_{imid} , NH signals), showing determination of the diastereomeric ratio.

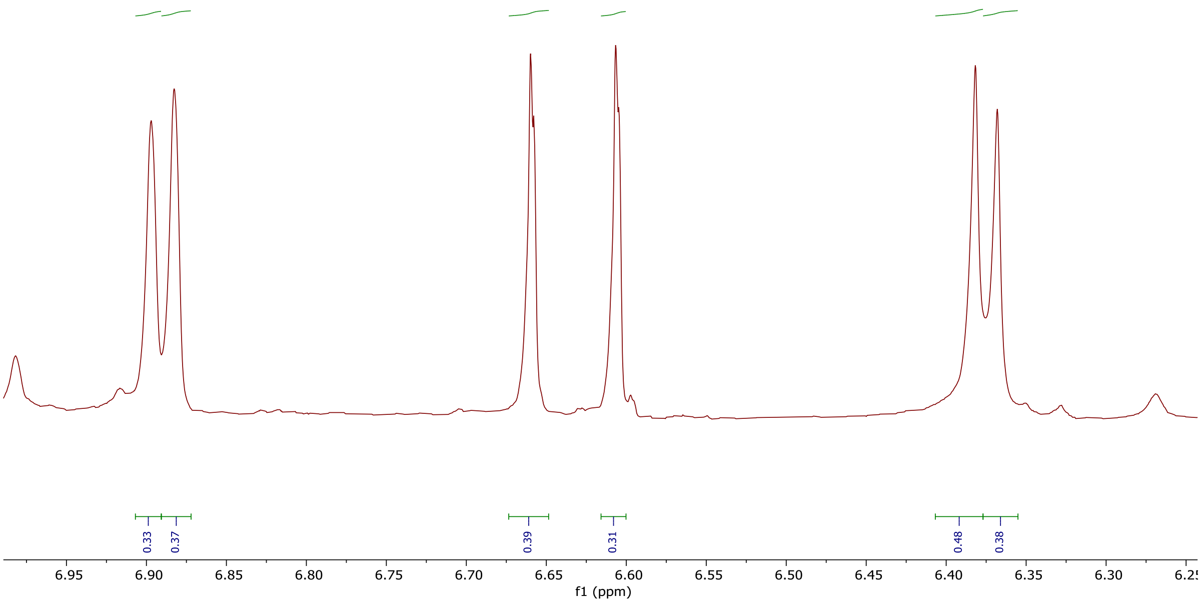

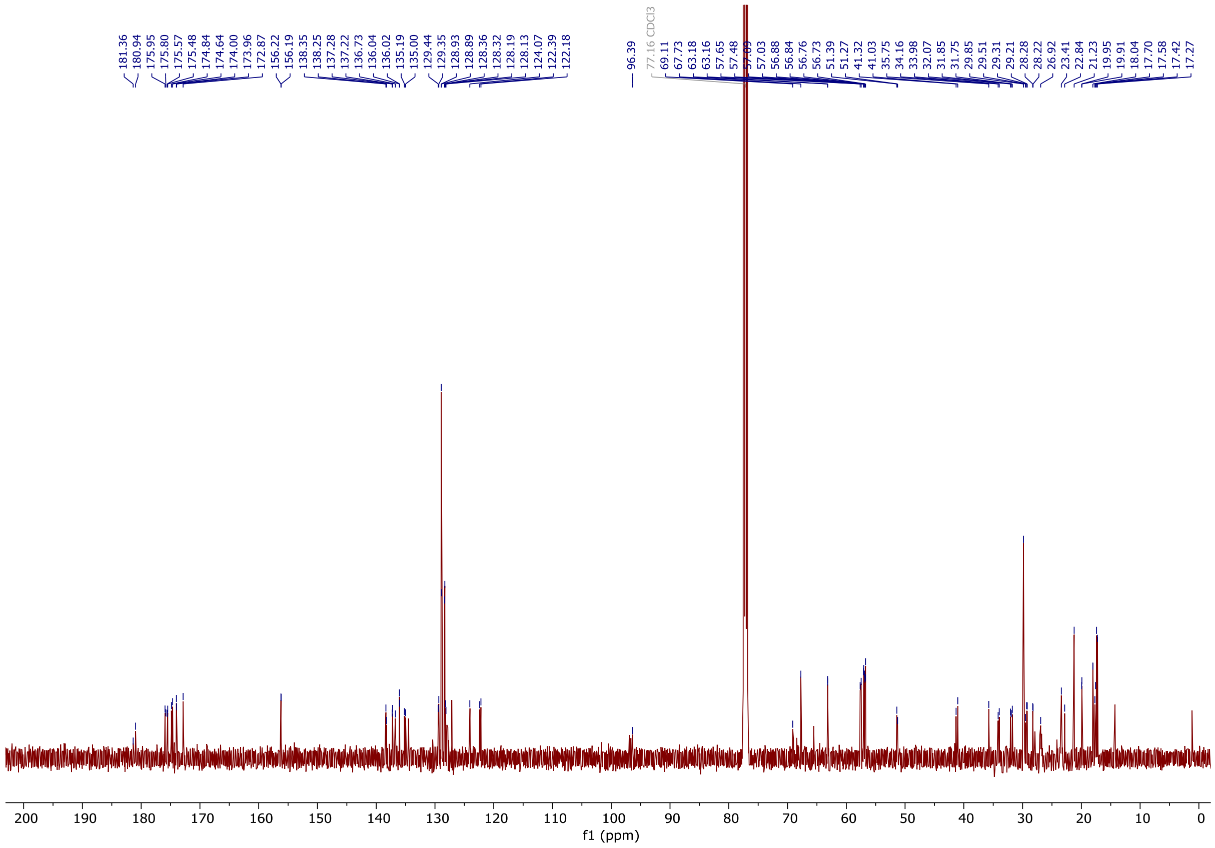
181.36
180.94
175.95
175.80
175.57
175.48
174.84
174.64
174.00
173.96
172.87
156.22
156.19
138.35
138.25
137.28
137.22
136.73
136.04
136.02
135.19
135.00
129.44
129.35
128.93
128.89
128.36
128.32
128.19
128.13
124.07
122.39
122.18
96.39
77.16 CDCl3
69.11
67.73
63.18
63.16
57.65
57.48
57.05
57.03
56.88
56.84
56.76
56.73
51.39
51.27
41.32
41.03
35.75
34.16
33.98
32.07
31.85
31.75
29.85
29.51
29.31
29.21
28.28
28.22
26.92
23.41
22.84
21.23
19.95
19.91
18.04
17.70
17.58
17.42
17.27
f1 (ppm)

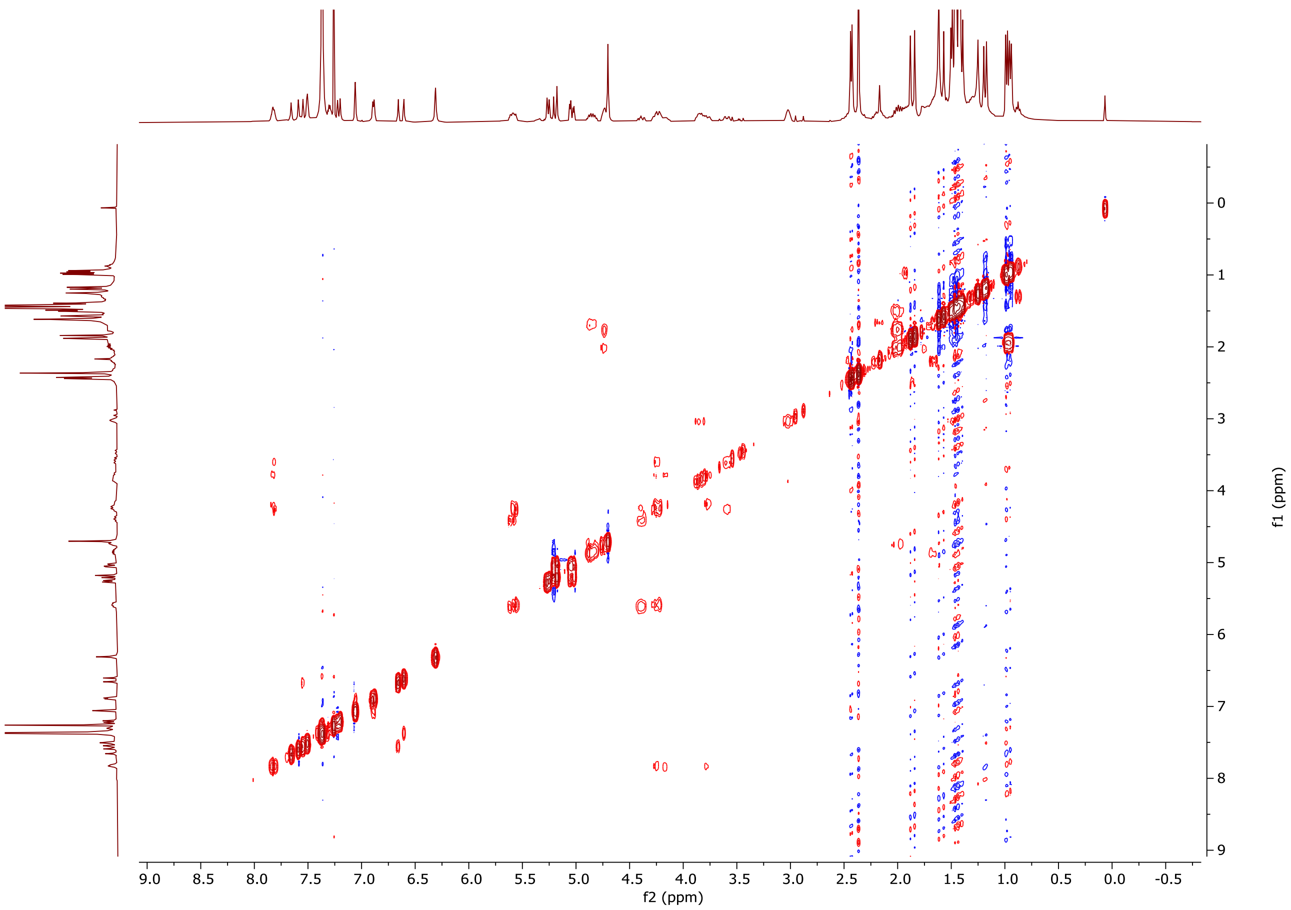
f2 (ppm)
f1 (ppm)

^1H ^{13}C HSQC

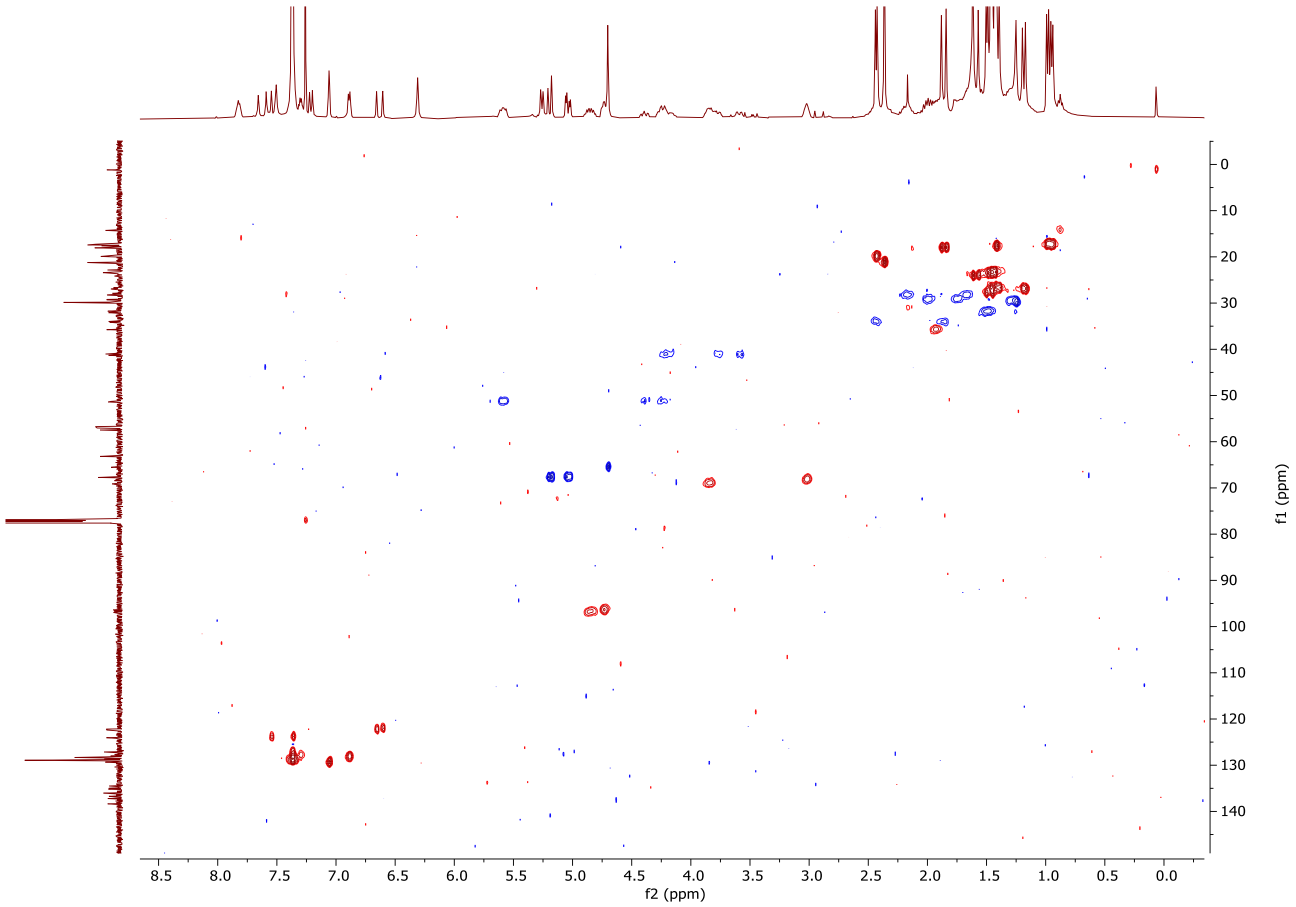

^{1}H ^{13}C HMBC

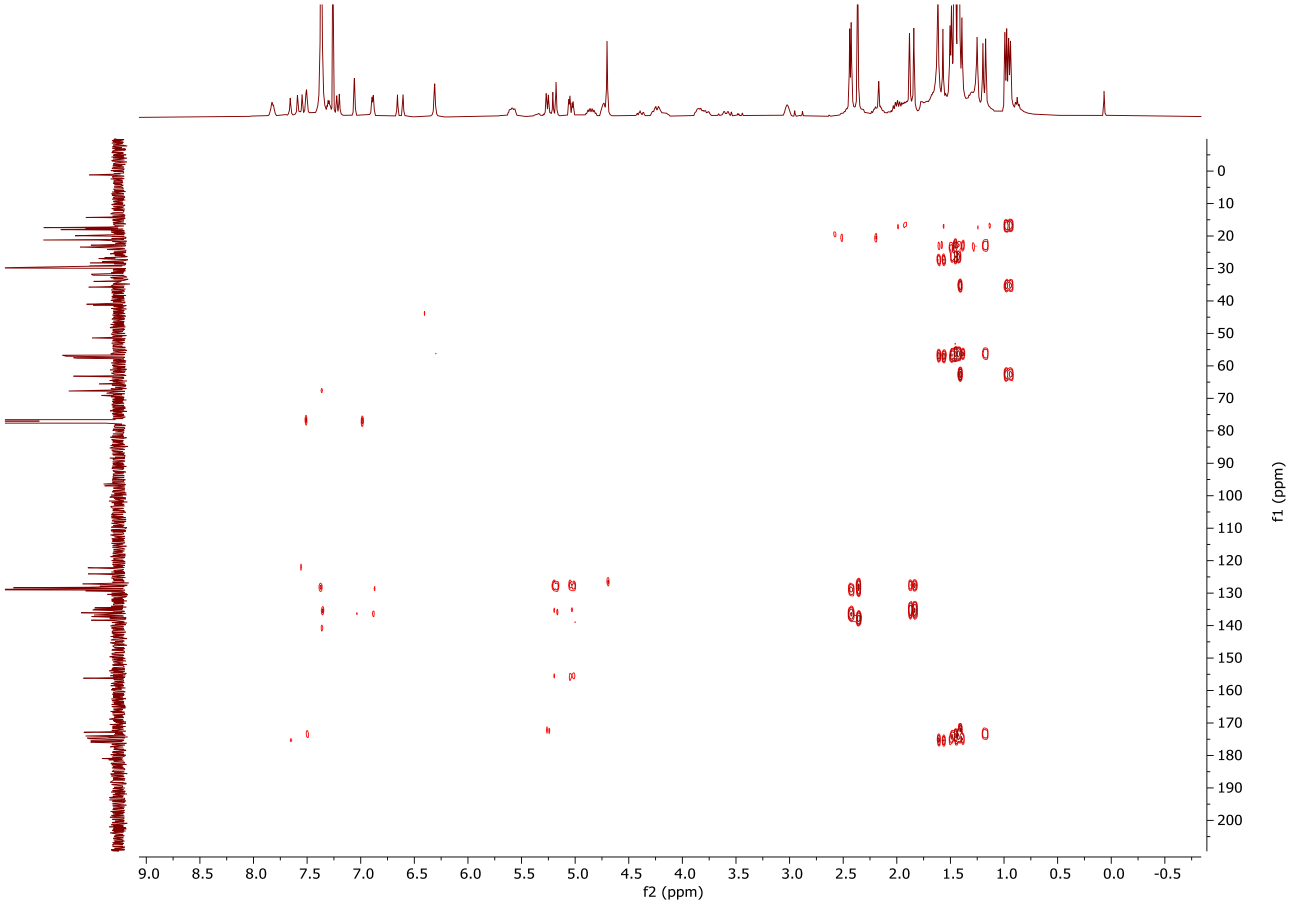

9.0
8.5
8.0
7.5
7.0
6.5
6.0
5.5
5.0
4.5
4.0
3.5
3.0
2.5
2.0
1.5
1.0
0.5
0.0
-0.5
f2 (ppm)
0
10
20
30
40
50
60
70
80
90
100
110
120
130
140
150
160
170
180
190
200
f1 (ppm)

4.19 Precursor 11 [Cbz-(L-αMeVal)$_2$Aib$_4$NH(CH$_2$)$_2$(Im-Ph)]$^+$Br$^-$

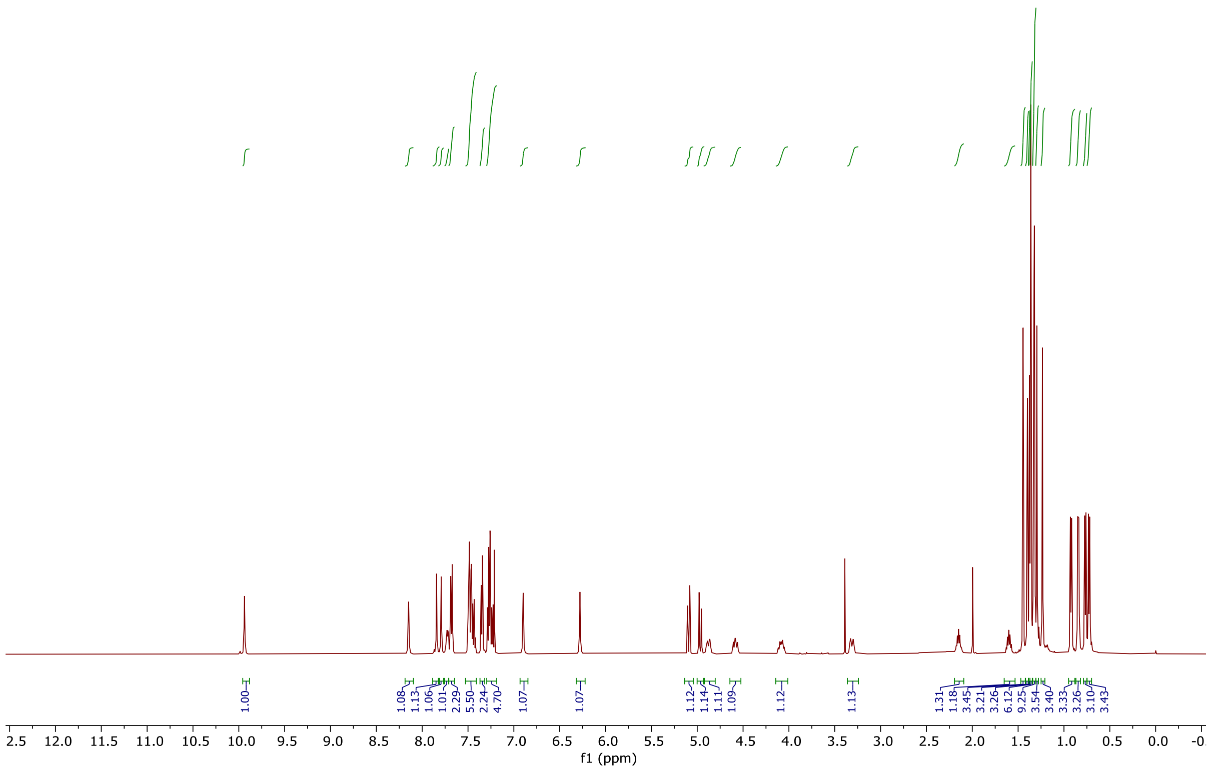

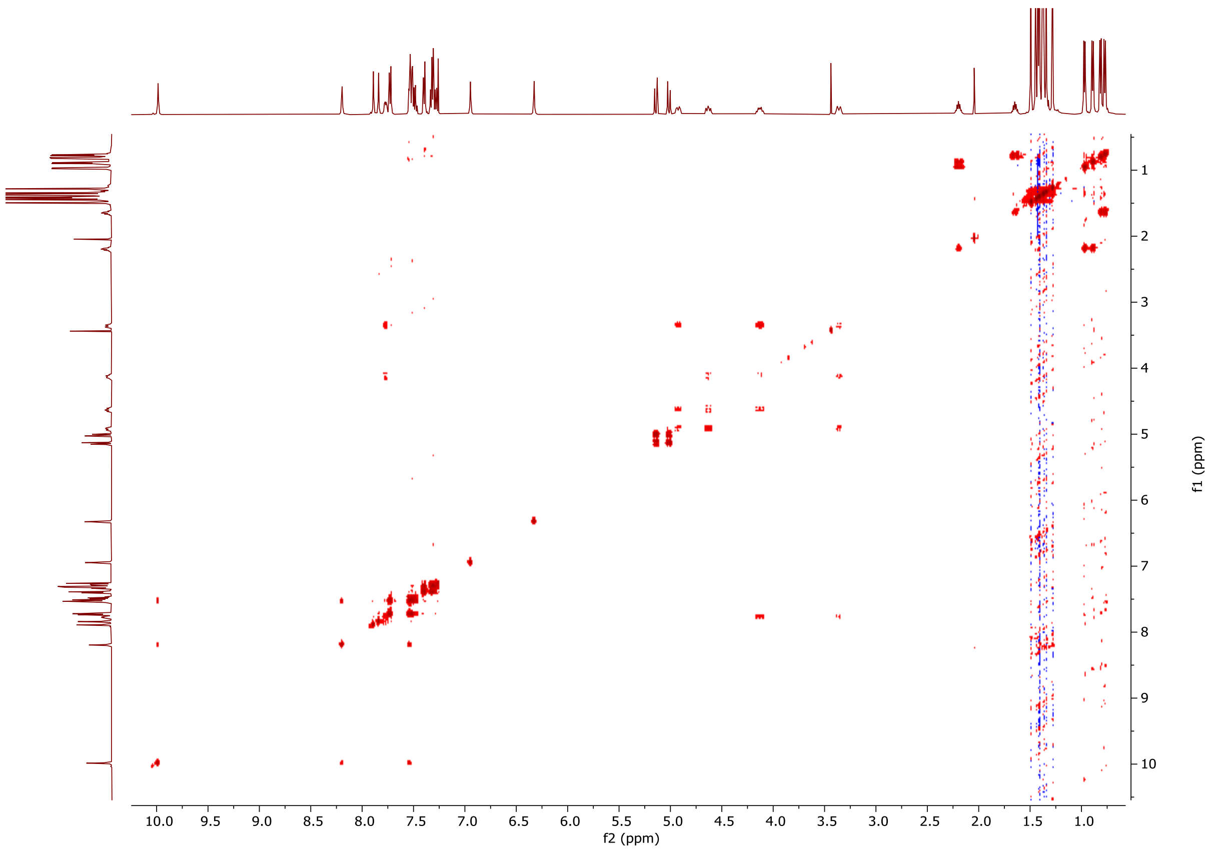

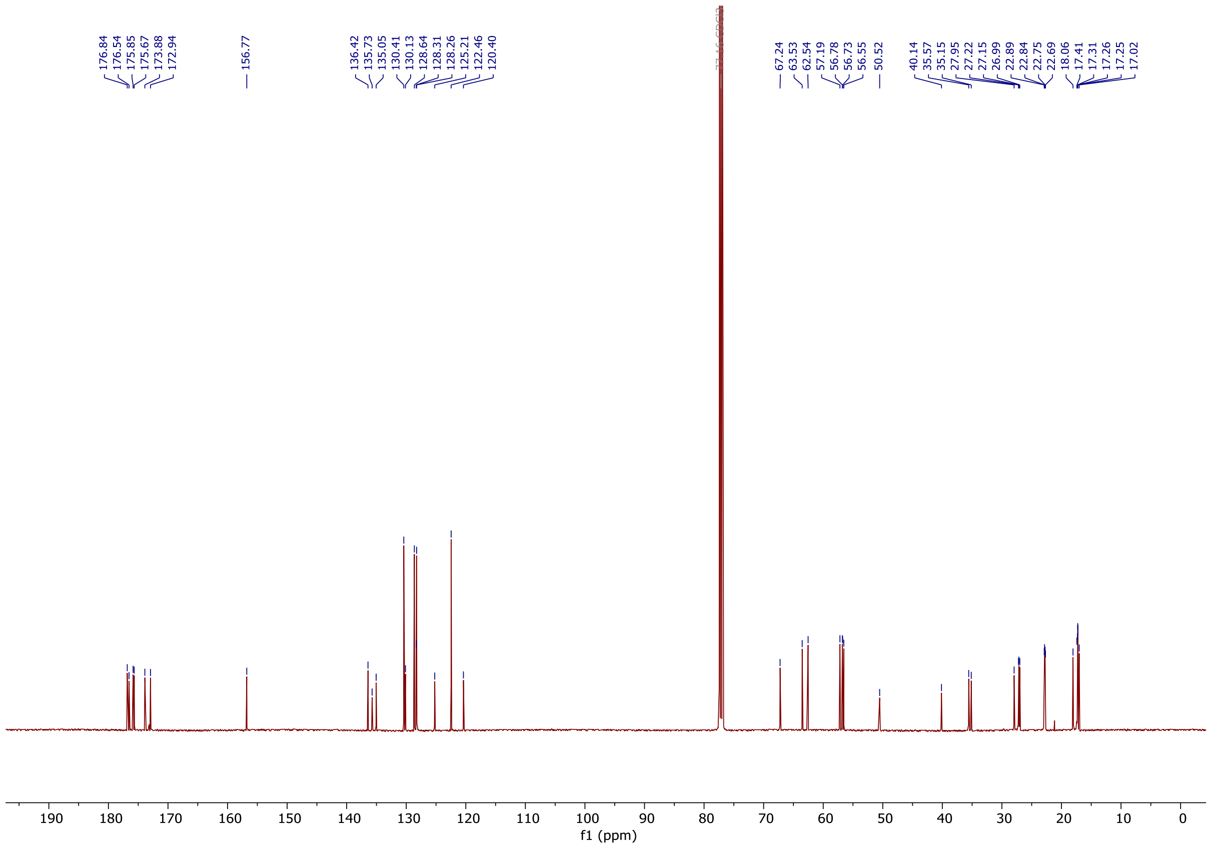
176.84
176.54
175.85
175.67
173.88
172.94
156.77
136.42
135.73
135.05
130.41
130.13
128.64
128.31
128.26
125.21
122.46
120.40
67.24
63.53
62.54
57.19
56.78
56.73
56.55
50.52
40.14
35.57
35.15
27.95
27.22
27.15
26.99
22.89
22.84
22.75
22.69
18.06
17.41
17.31
17.26
17.25
17.02
f1 (ppm)

^{1}H ^{13}C HSQC

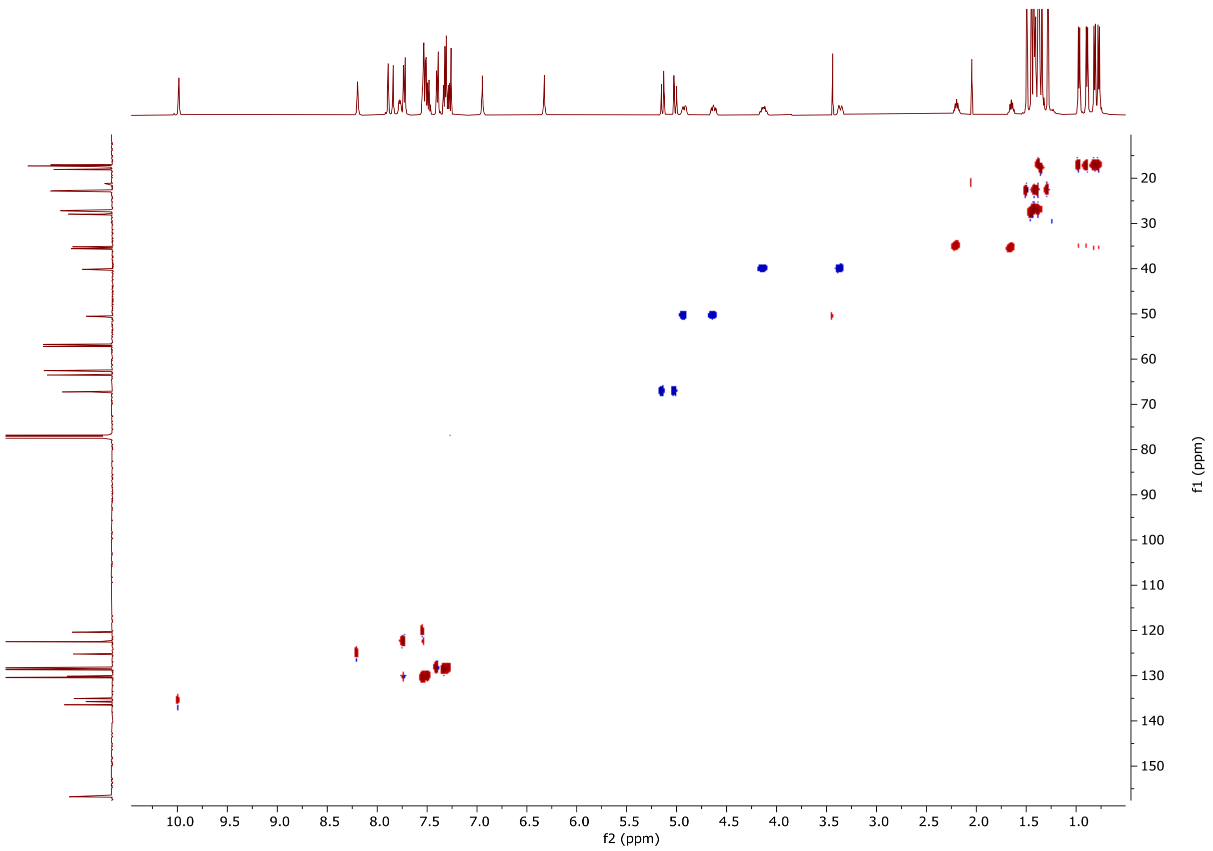
f2 (ppm)
f1 (ppm)

^{1}H ^{13}C HMBC

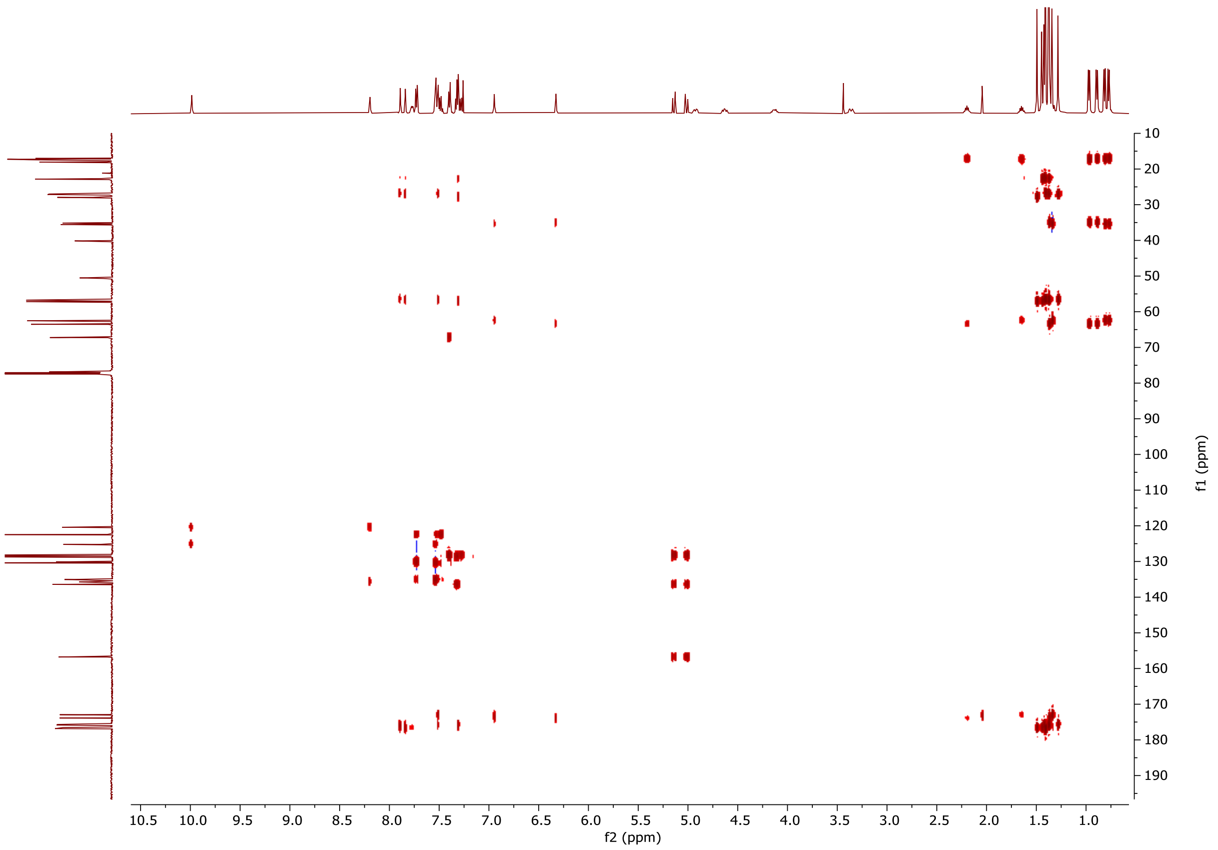

10.5
10.0
9.5
9.0
8.5
8.0
7.5
7.0
6.5
6.0
5.5
5.0
4.5
4.0
3.5
3.0
2.5
2.0
1.5
1.0
f2 (ppm)
10
20
30
40
50
60
70
80
90
100
110
120
130
140
150
160
170
180
190
f1 (ppm)

4.20 Foldamer 17 [(Cbz-(L-αMeVal)$_2$Aib$_4$NH(CH$_2$)$_2$-NHC-Ph)Rh(Cl)(COD)]

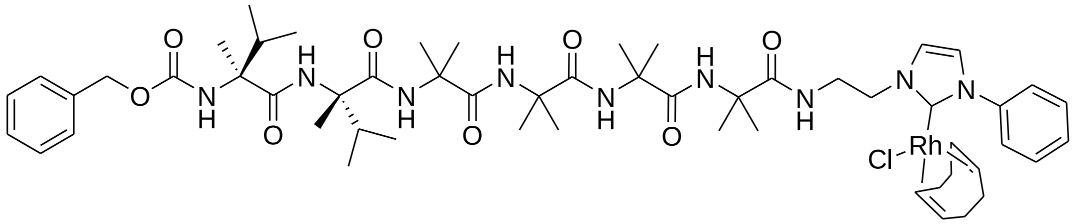

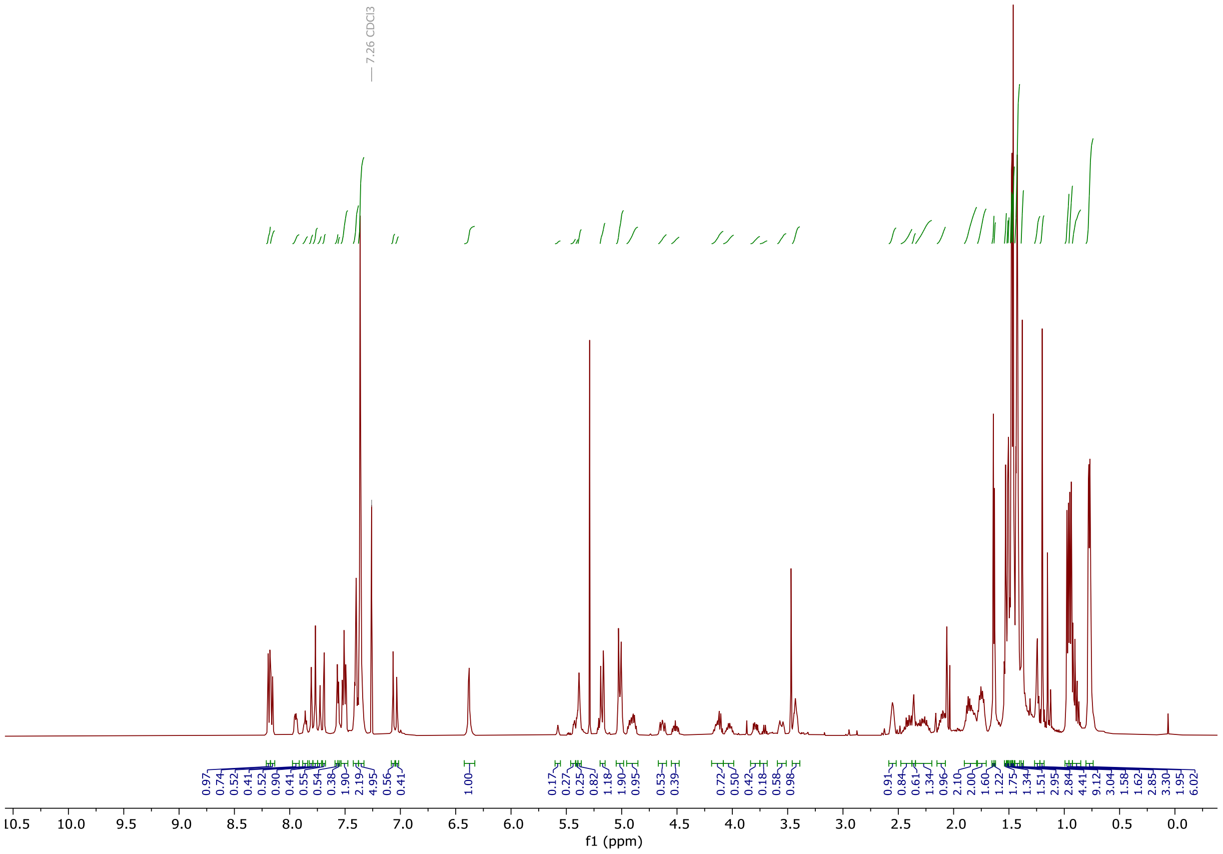

Line fitting of ^{1}H NMR signals to determine the diastereomeric ratio

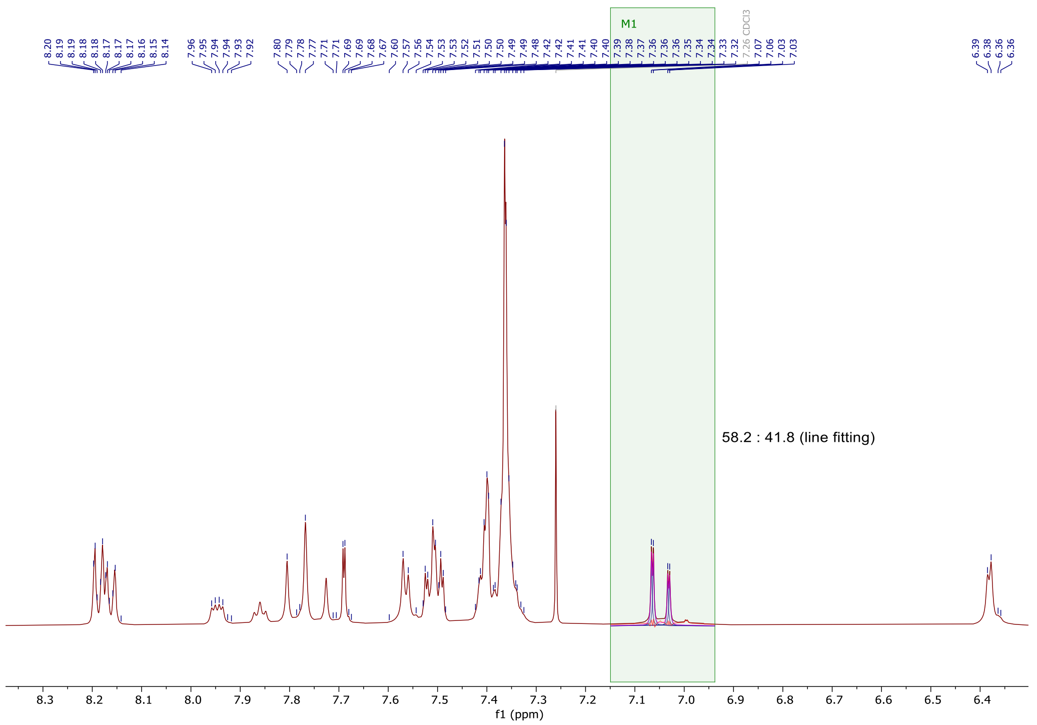

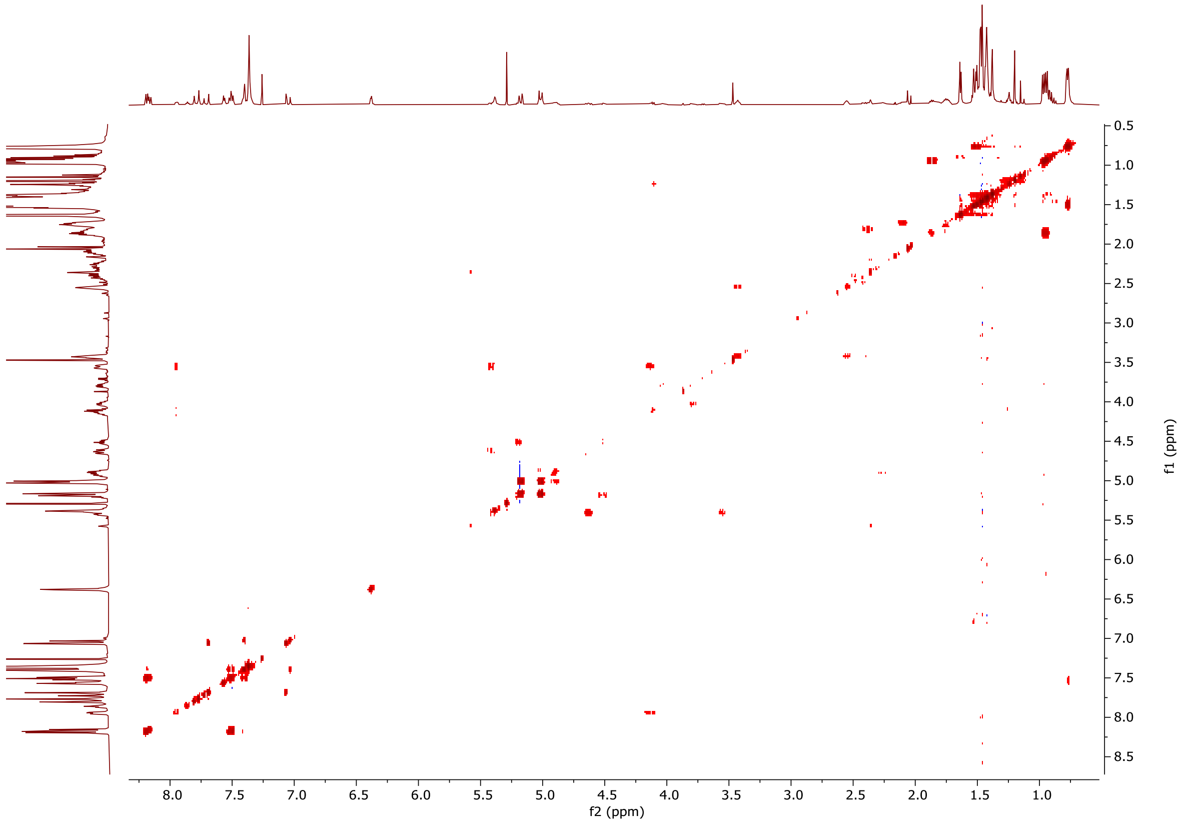

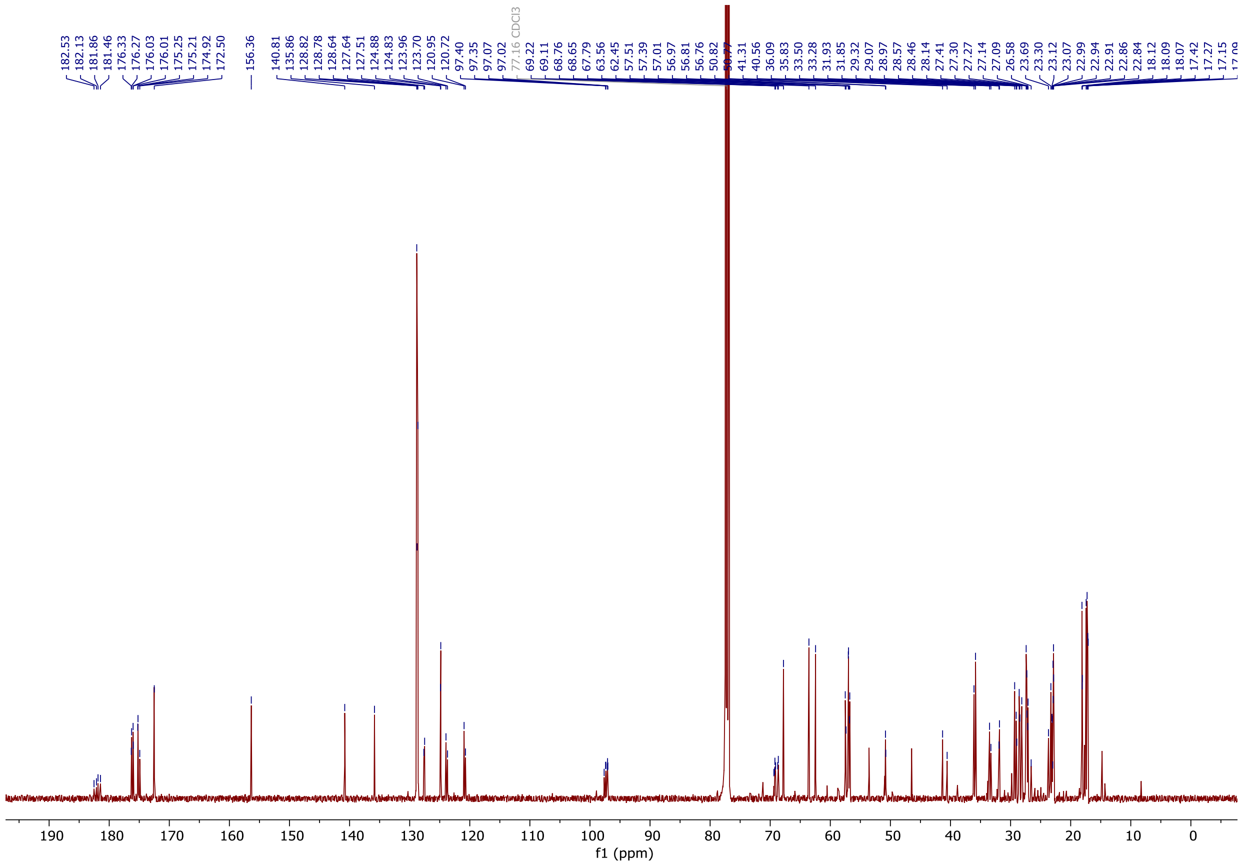
182.53
182.13
181.86
181.46
176.33
176.27
176.03
176.01
175.25
175.21
174.92
172.50
156.36
140.81
135.86
128.82
128.78
128.64
127.64
127.51
124.88
124.83
123.96
123.70
120.95
120.72
97.40
97.35
97.07
97.02
77.16 CDCl3
69.22
69.11
68.76
68.65
67.79
63.56
62.45
57.51
57.39
57.01
56.97
56.81
56.76
50.82
41.31
40.56
36.09
35.83
33.50
33.28
31.93
31.85
29.32
29.07
28.97
28.57
28.46
28.14
27.41
27.30
27.27
27.14
27.09
26.58
23.69
23.30
23.12
23.07
22.99
22.94
22.91
22.86
22.84
18.12
18.09
18.07
17.42
17.27
17.15
f1 (ppm)

^{1}H ^{13}C HSQC

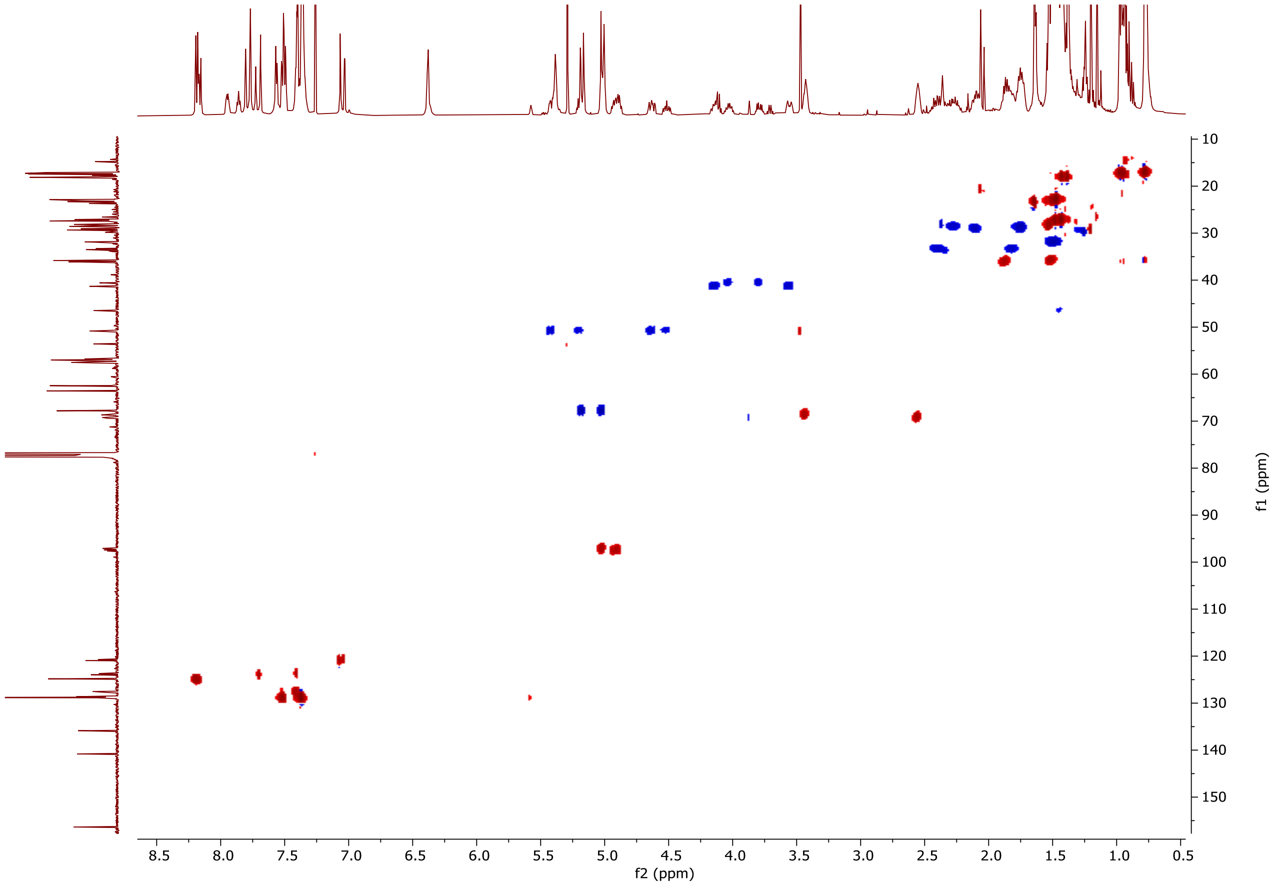
f2 (ppm)
f1 (ppm)

^{1}H ^{13}C HMBC

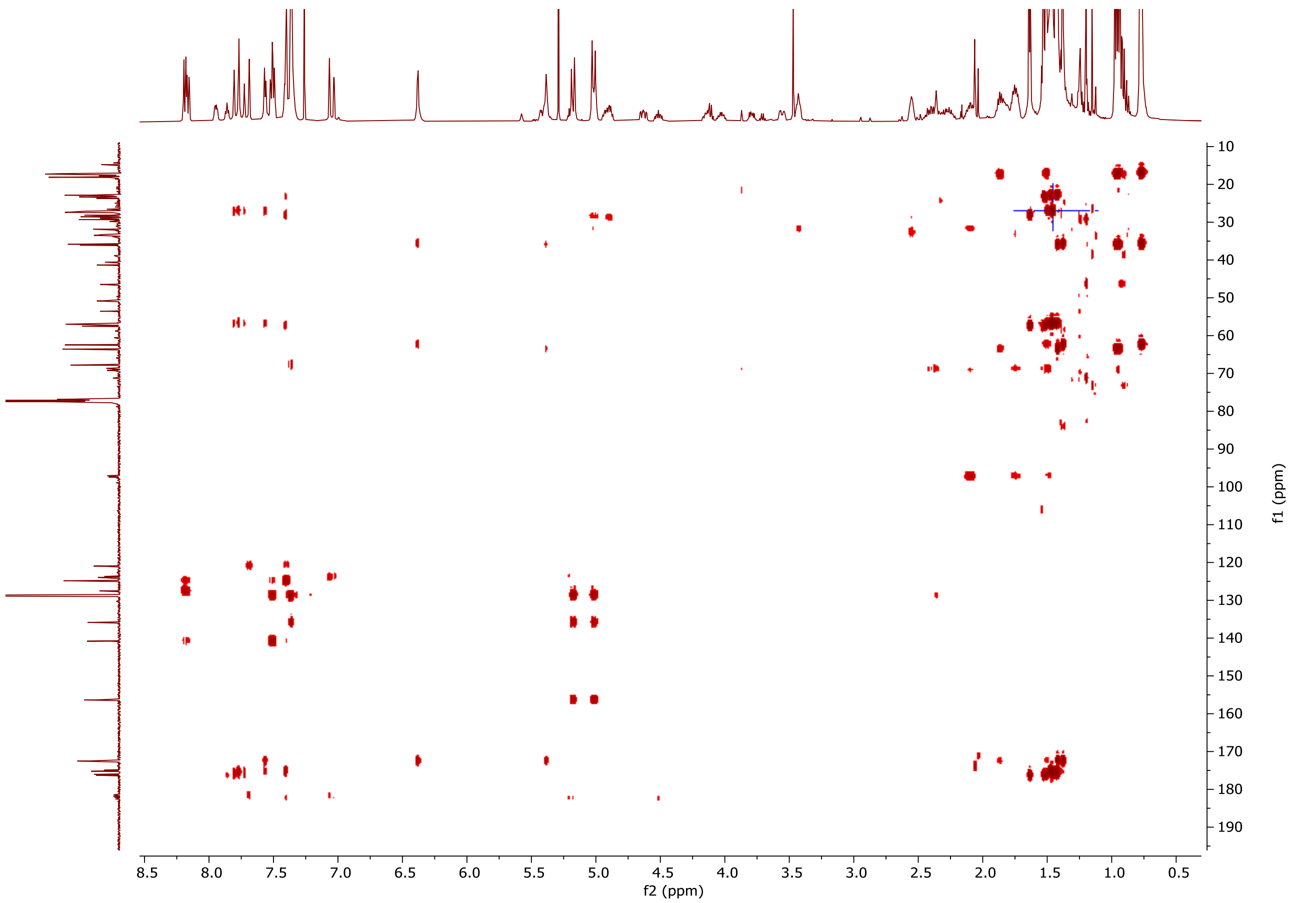

f1 (ppm)
f2 (ppm)
10
20
30
40
50
60
70
80
90
100
110
120
130
140
150
160
170
180
190
8.5
8.0
7.5
7.0
6.5
6.0
5.5
5.0
4.5
4.0
3.5
3.0
2.5
2.0
1.5
1.0
0.5

4.21 Precursor 12 [Cbz-(L-αMeVal)$_2$Aib$_4$NH(CH$_2$)$_2$(Im-Mes)]$^+$Br$^−$

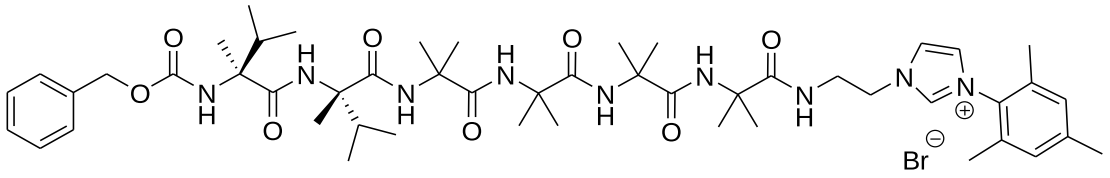

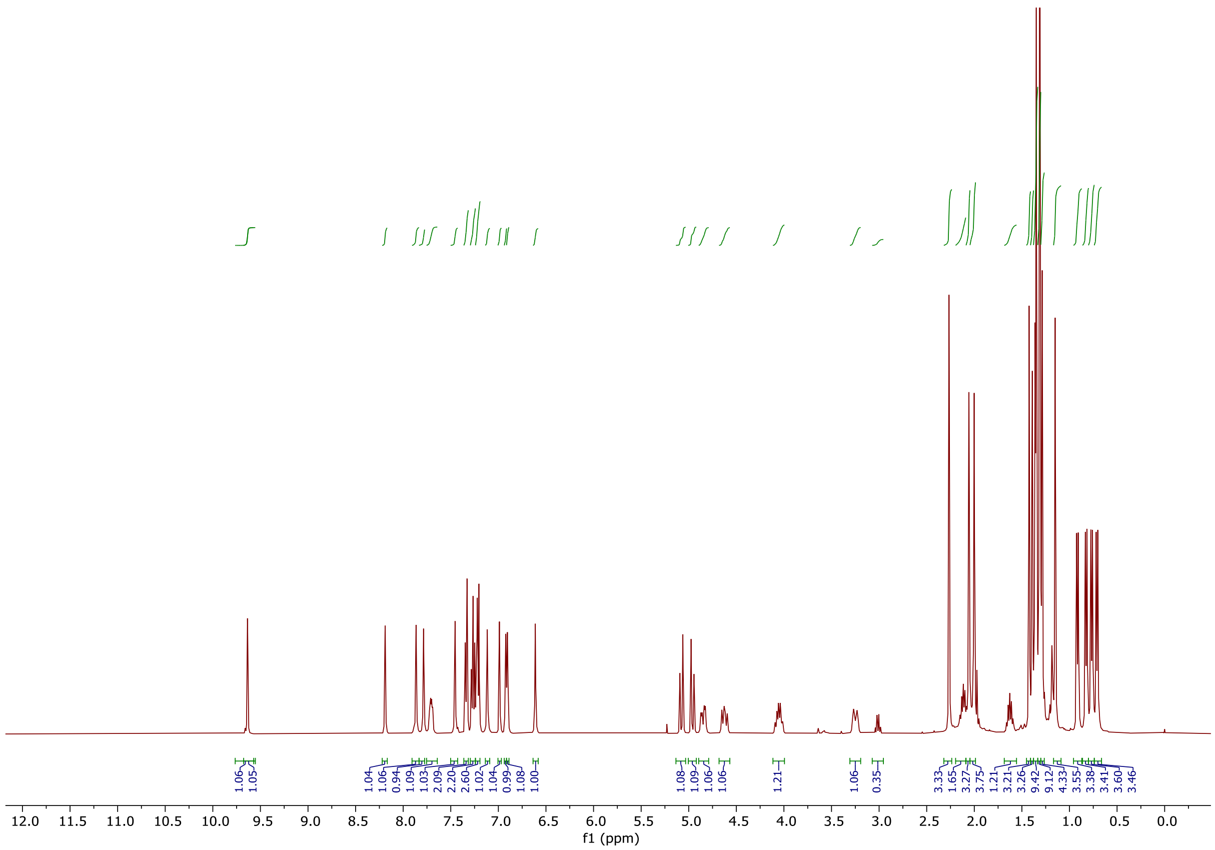

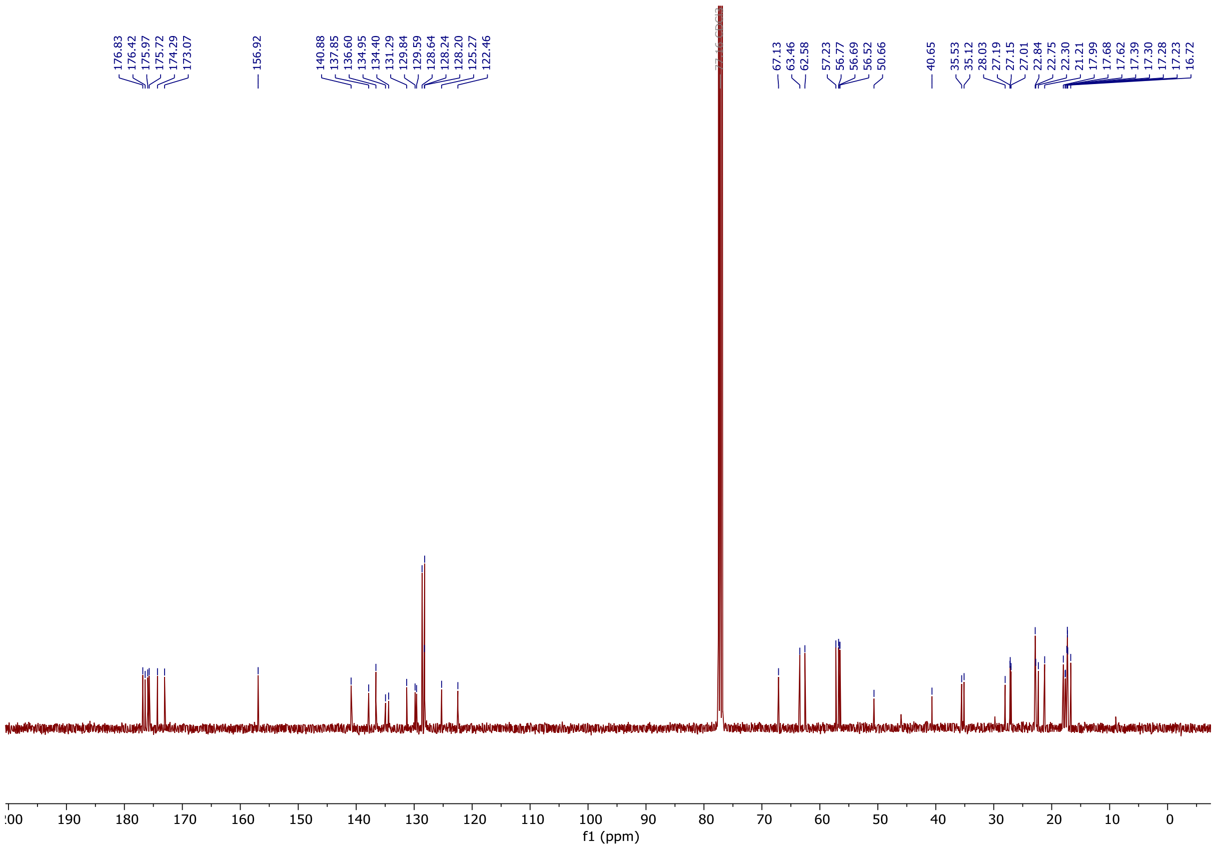
176.83
176.42
175.97
175.72
174.29
173.07
156.92
140.88
137.85
136.60
134.95
134.40
131.29
129.84
129.59
128.64
128.24
128.20
125.27
122.46
67.13
63.46
62.58
57.23
56.77
56.69
56.52
50.66
40.65
35.53
35.12
28.03
27.19
27.15
27.01
22.84
22.75
22.30
21.21
17.99
17.68
17.62
17.39
17.30
17.28
17.23
16.72
f1 (ppm)

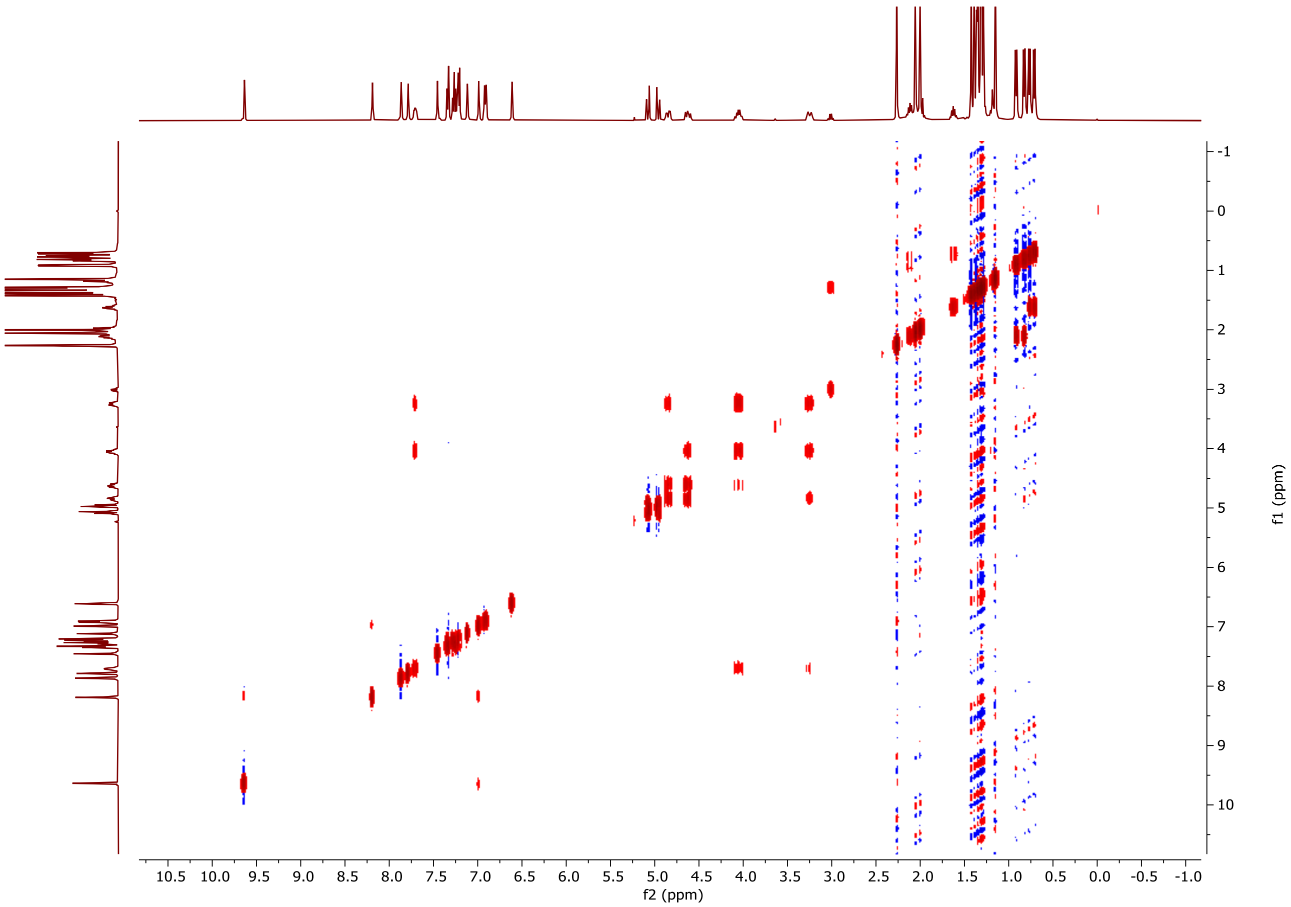
f2 (ppm)
f1 (ppm)

^{1}H ^{13}C HSQC

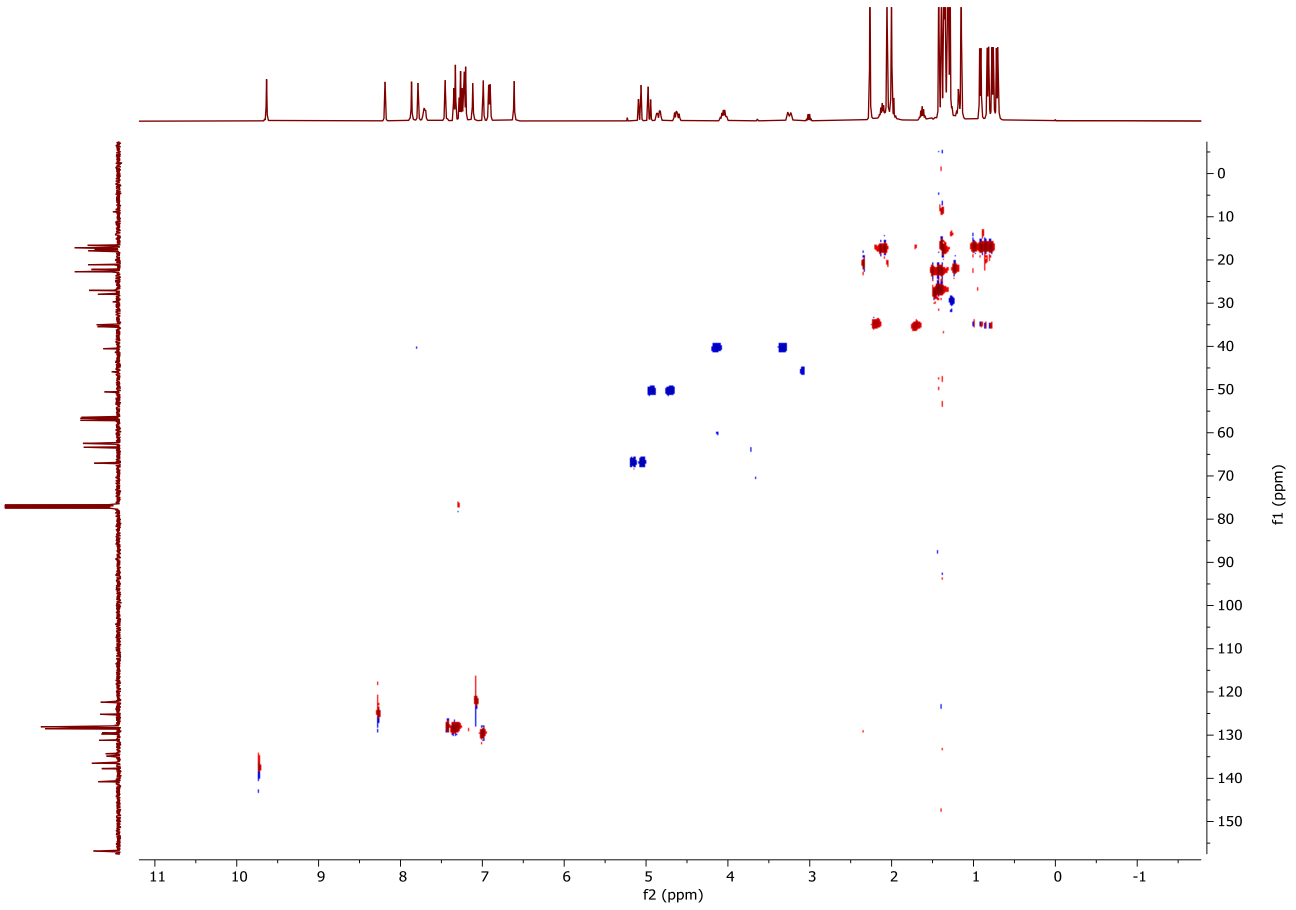

^{1}H ^{13}C HMBC

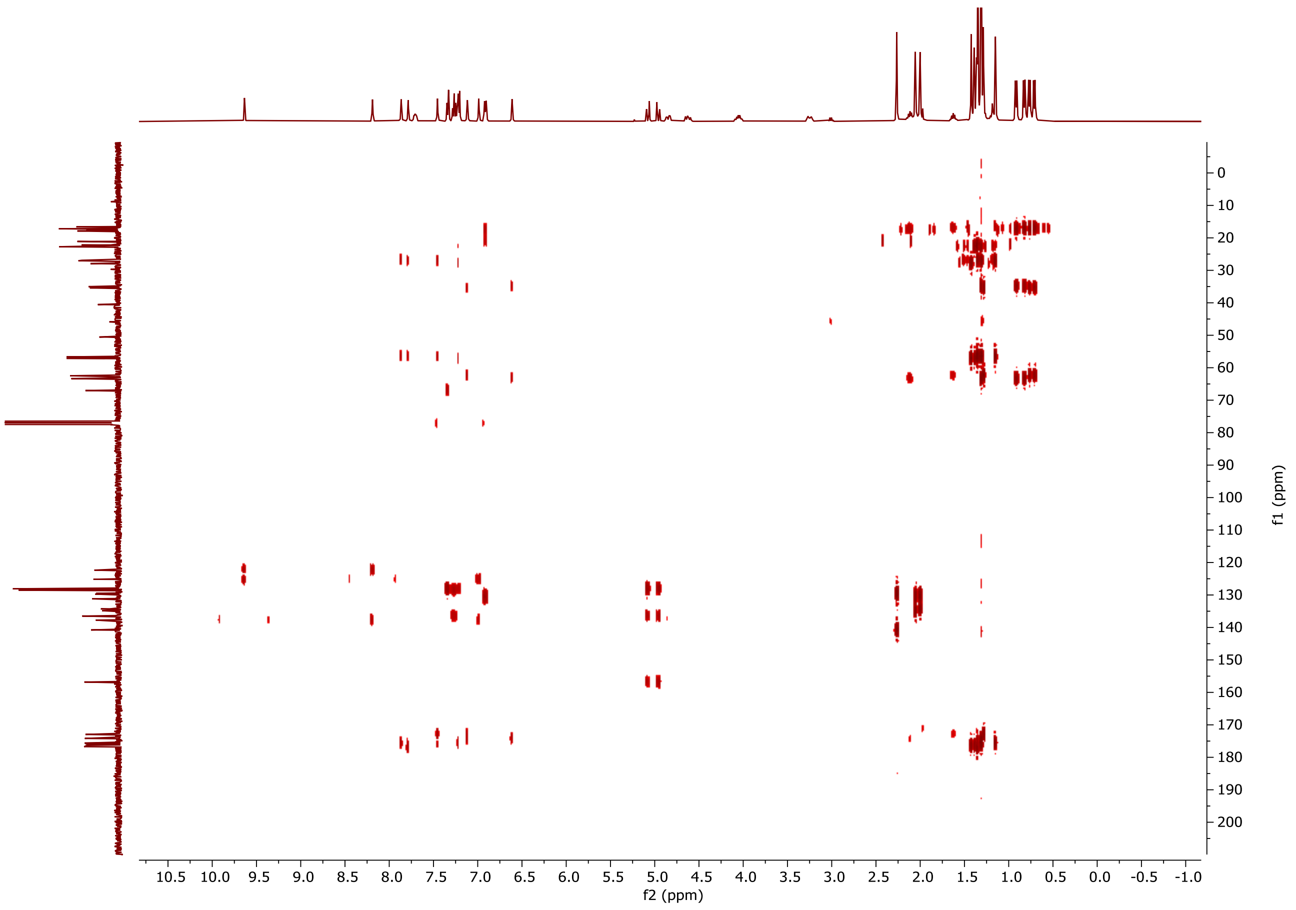

4.22 Foldamer 18 [(Cbz-(L-αMeVal)$_2$Aib$_4$NH(CH$_2$)$_2$-NHC-Mes)Rh(Cl)(COD)]

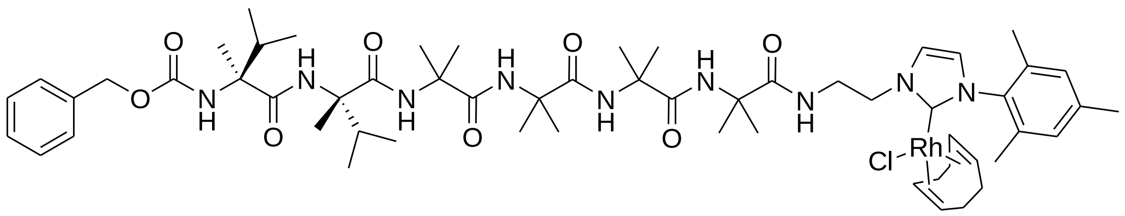

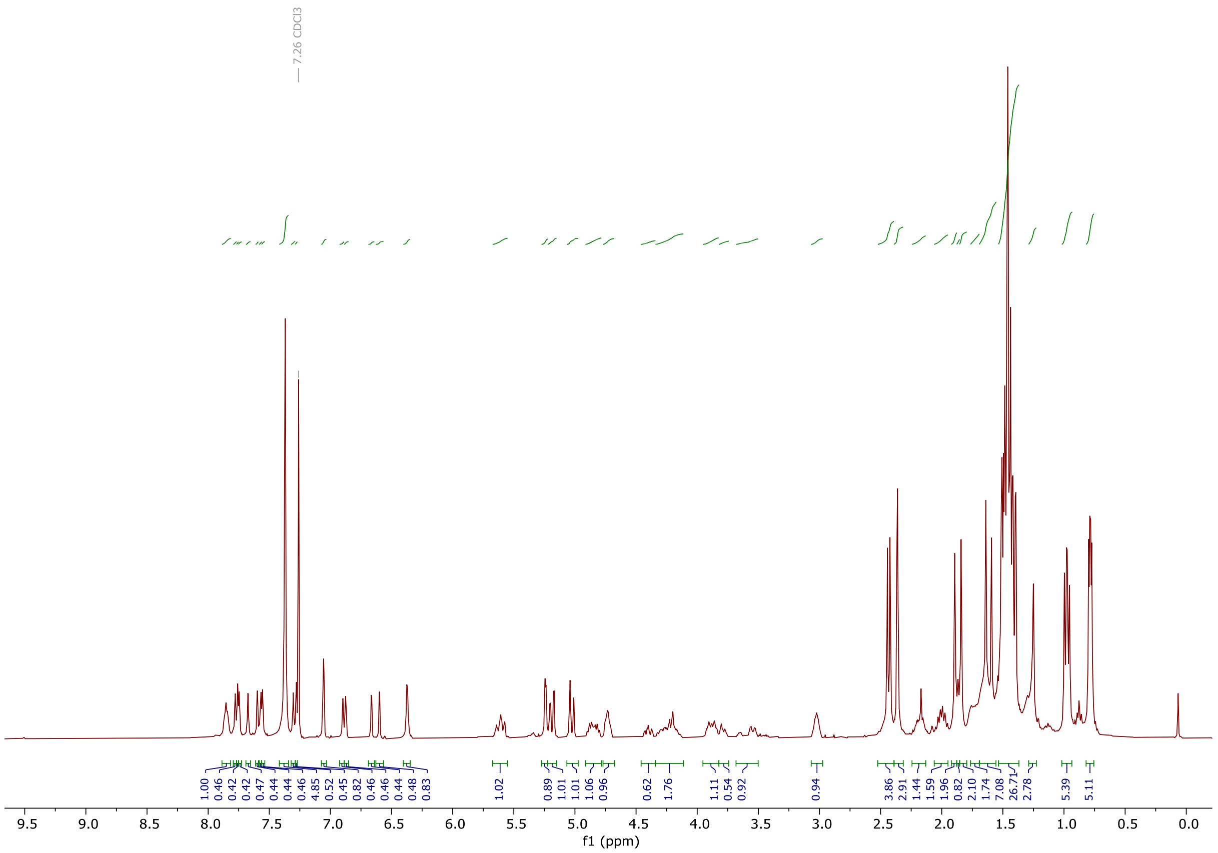

Expansion of ¹H NMR spectrum (CH_{imid} signals) and line fitting to determine the diastereomeric ratio

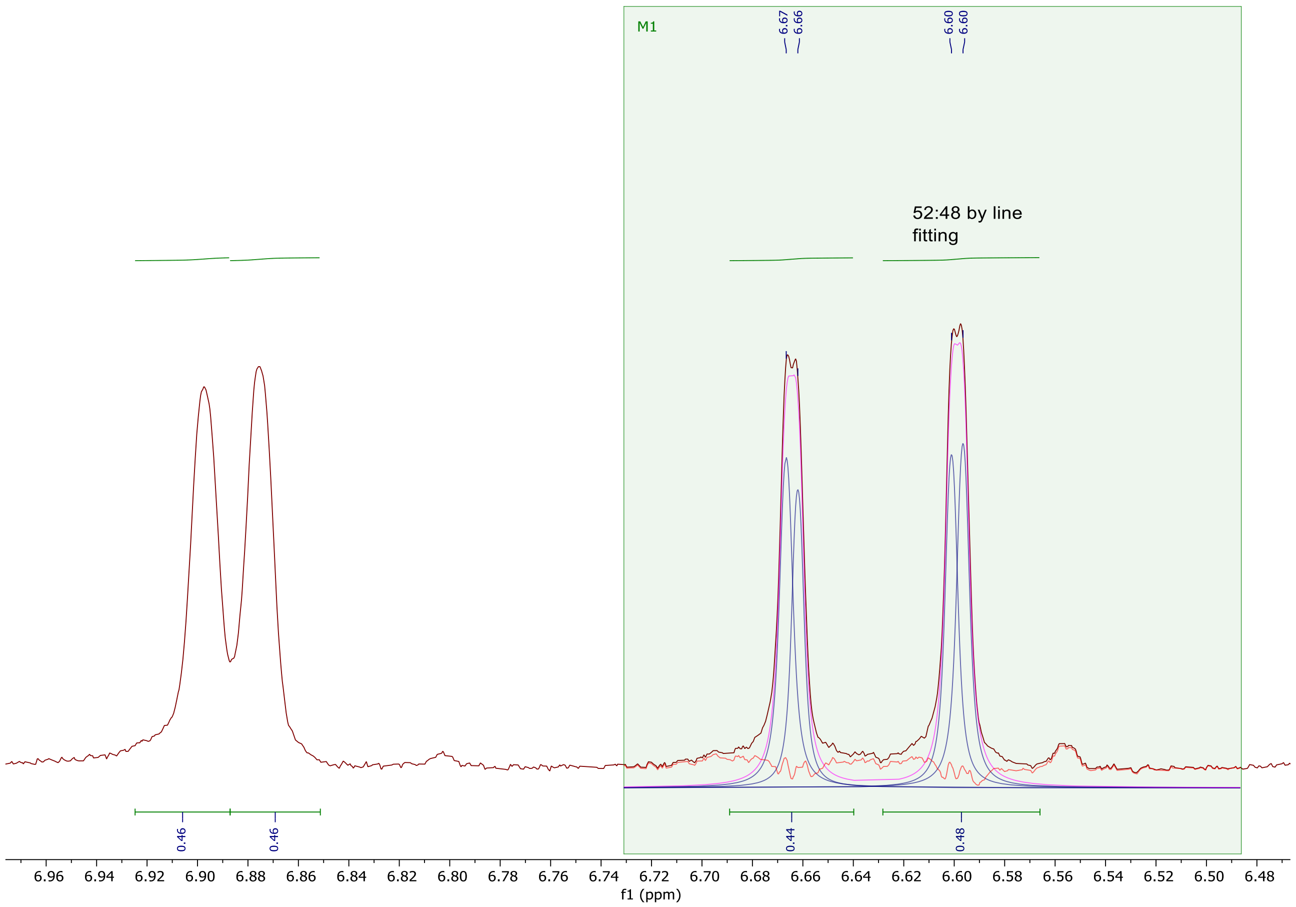

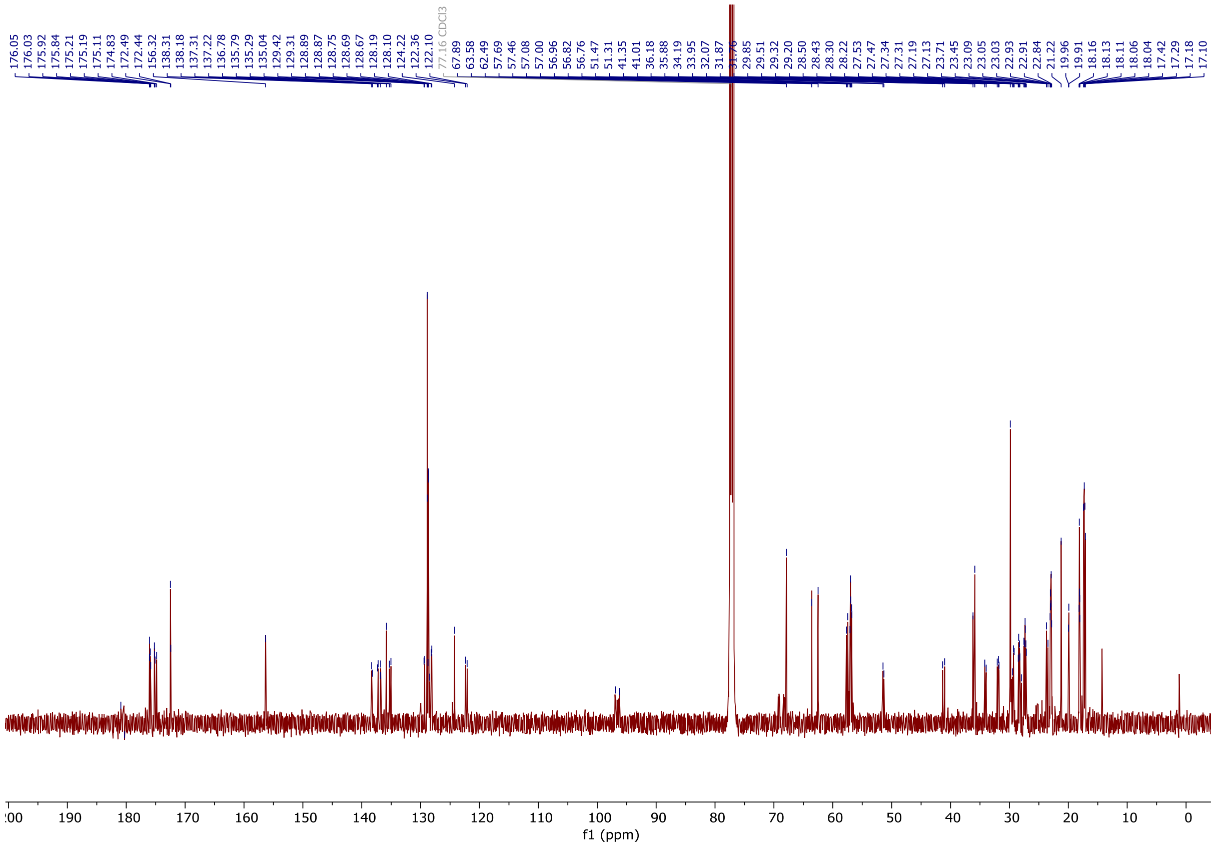

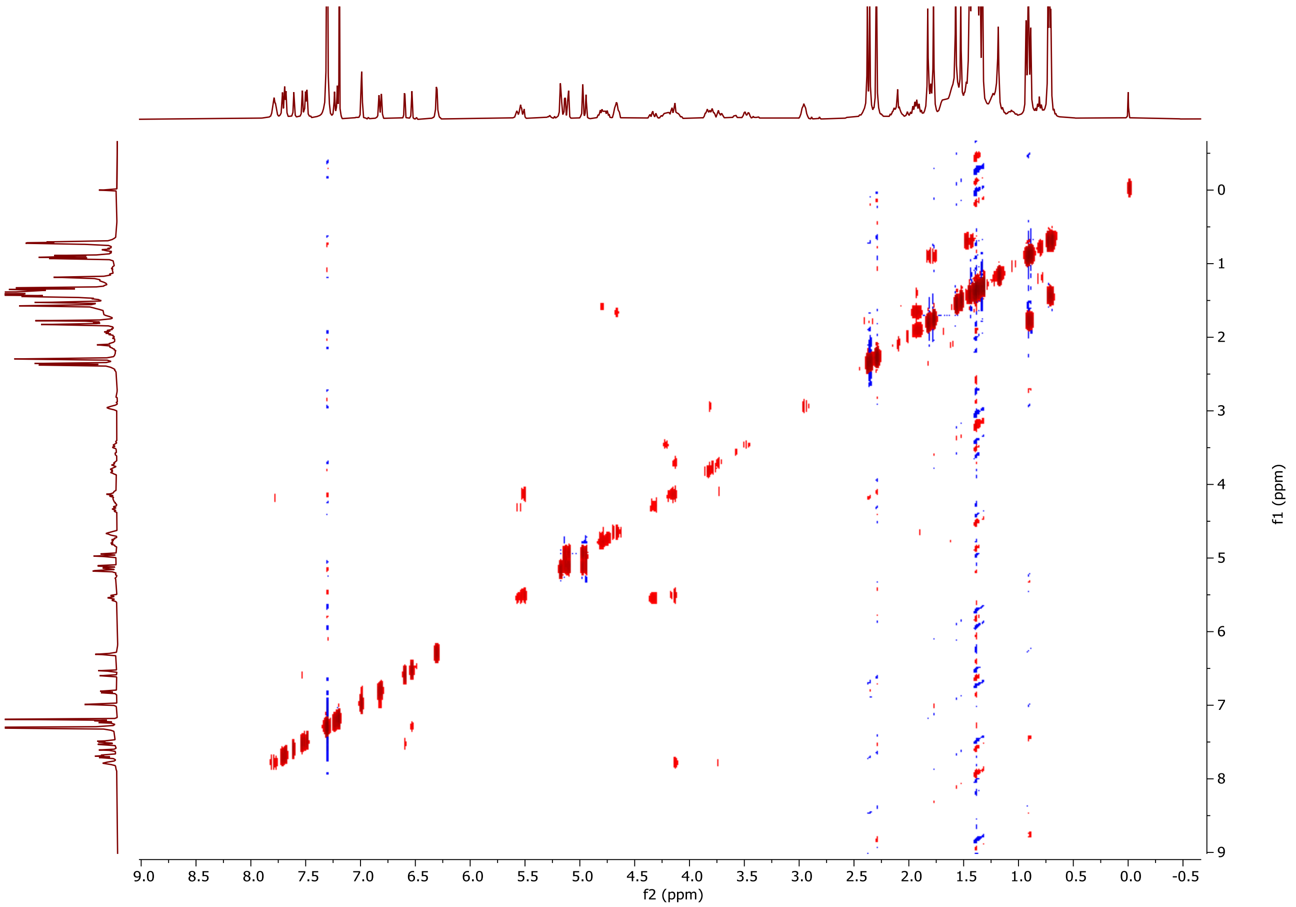

^{1}H ^{13}C HSQC

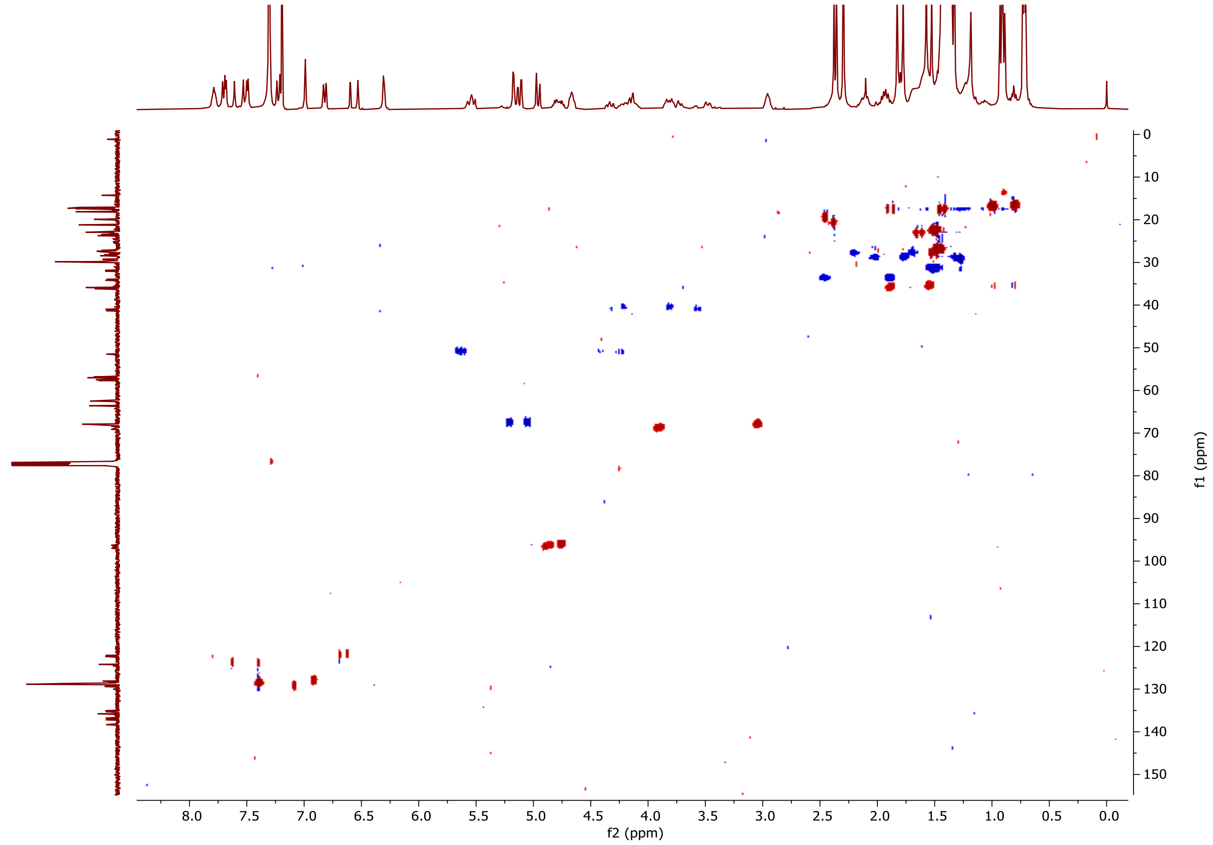

^{1}H ^{13}C HMBC

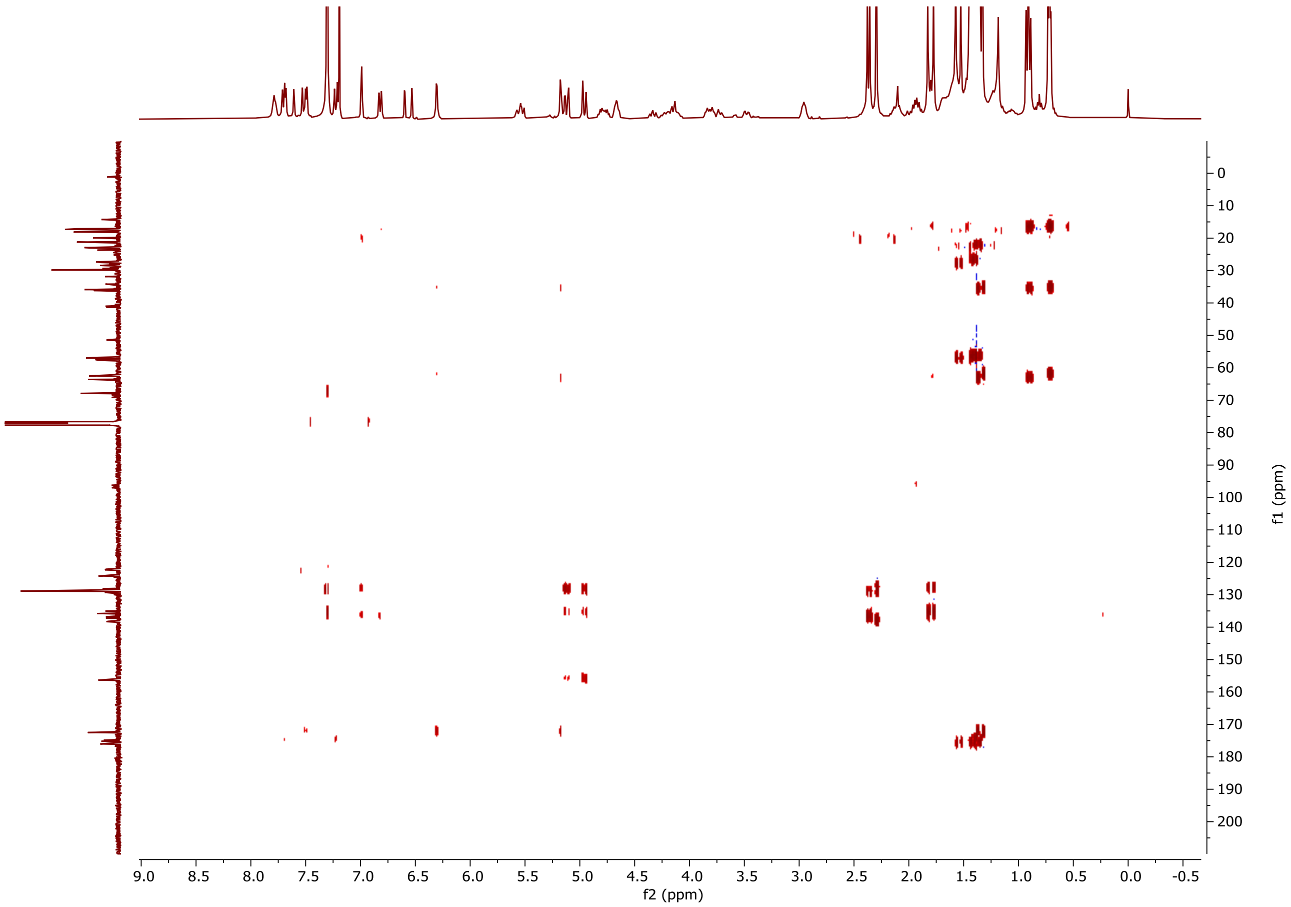

4.23 Precursor 13 [Cbz-(L-αMeVal)Aib$_5$NH(CH$_2$)$_2$(Im-Mes)]$^+$Br$^−$

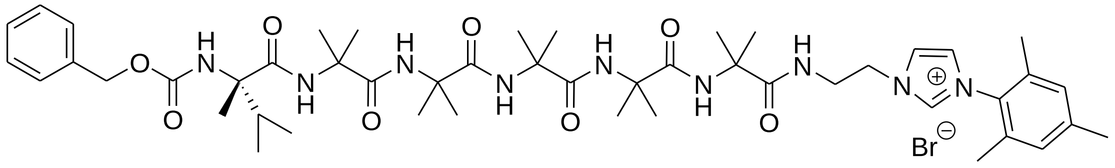

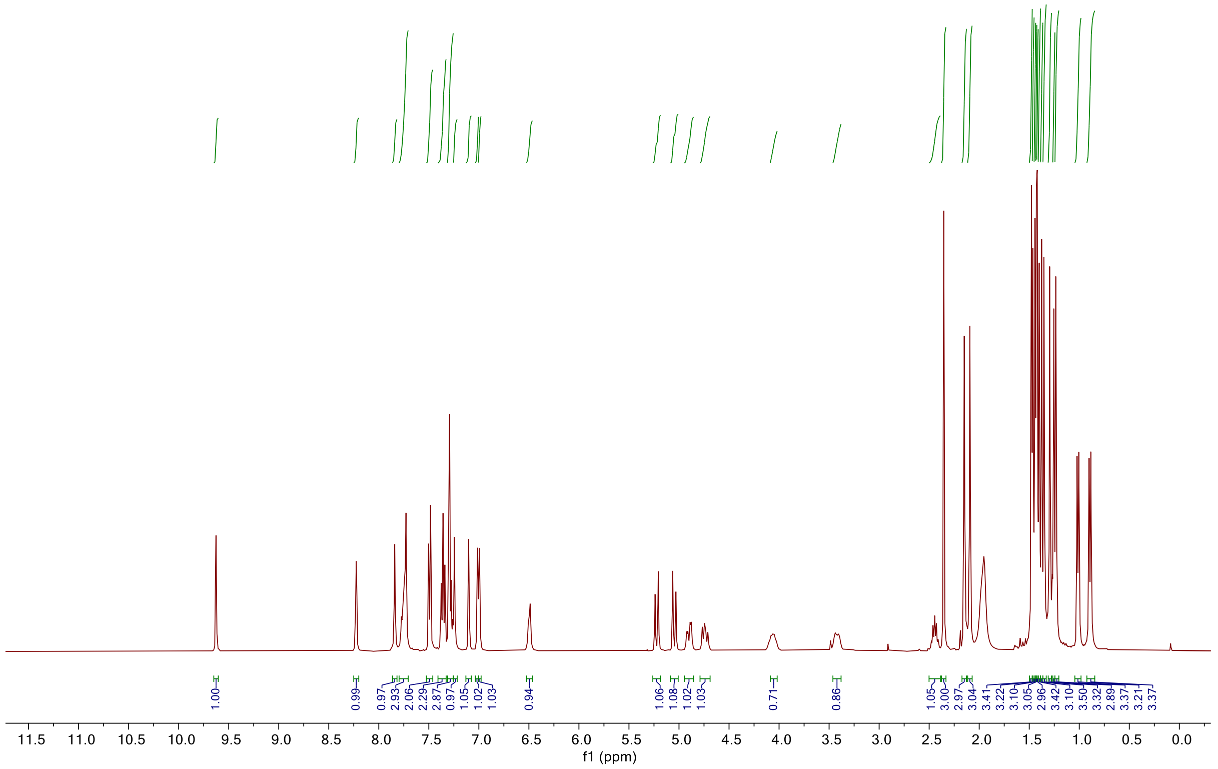

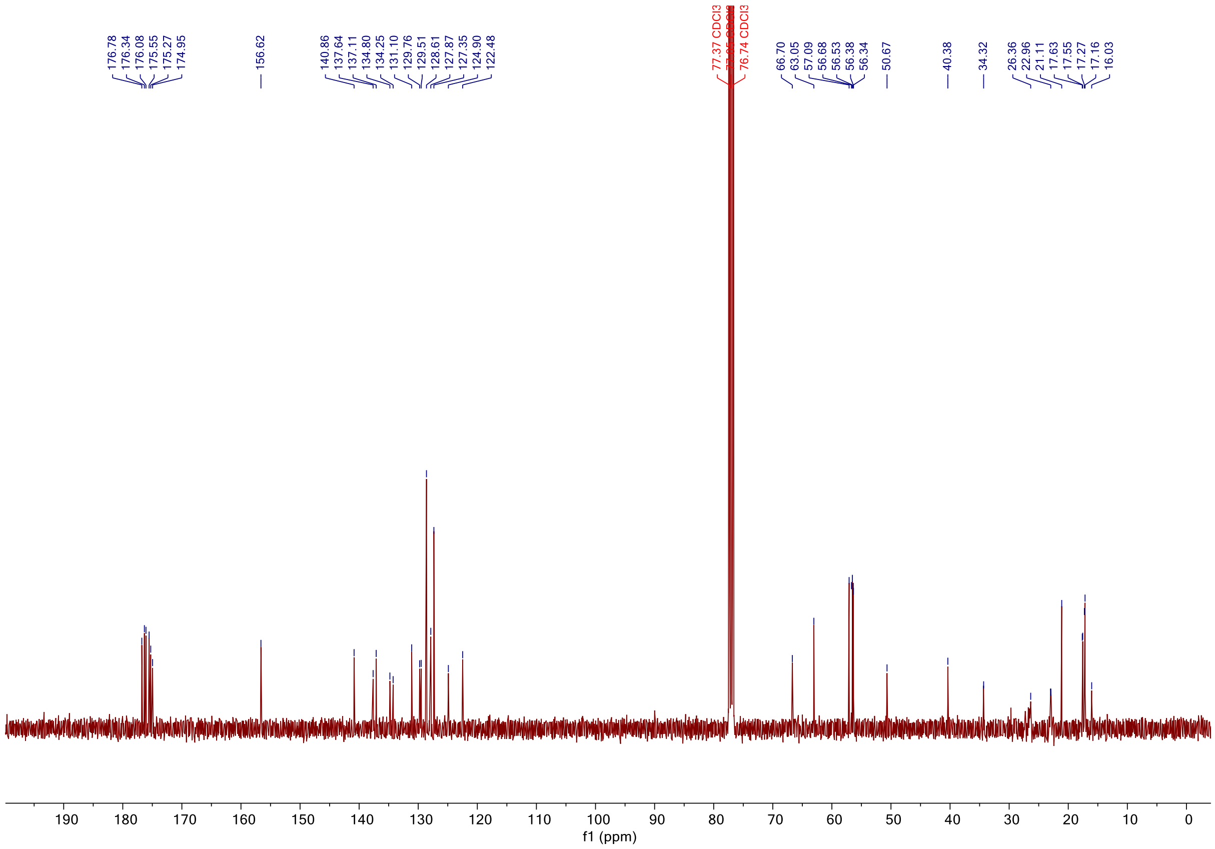
176.78
176.34
176.08
175.55
175.27
174.95
156.62
140.86
137.64
137.11
134.80
134.25
131.10
129.76
129.51
128.61
127.87
127.35
124.90
122.48
77.37 CDCl3
76.74 CDCl3
66.70
63.05
57.09
56.68
56.53
56.38
56.34
50.67
40.38
34.32
26.36
22.96
21.11
17.63
17.55
17.27
17.16
16.03
f1 (ppm)

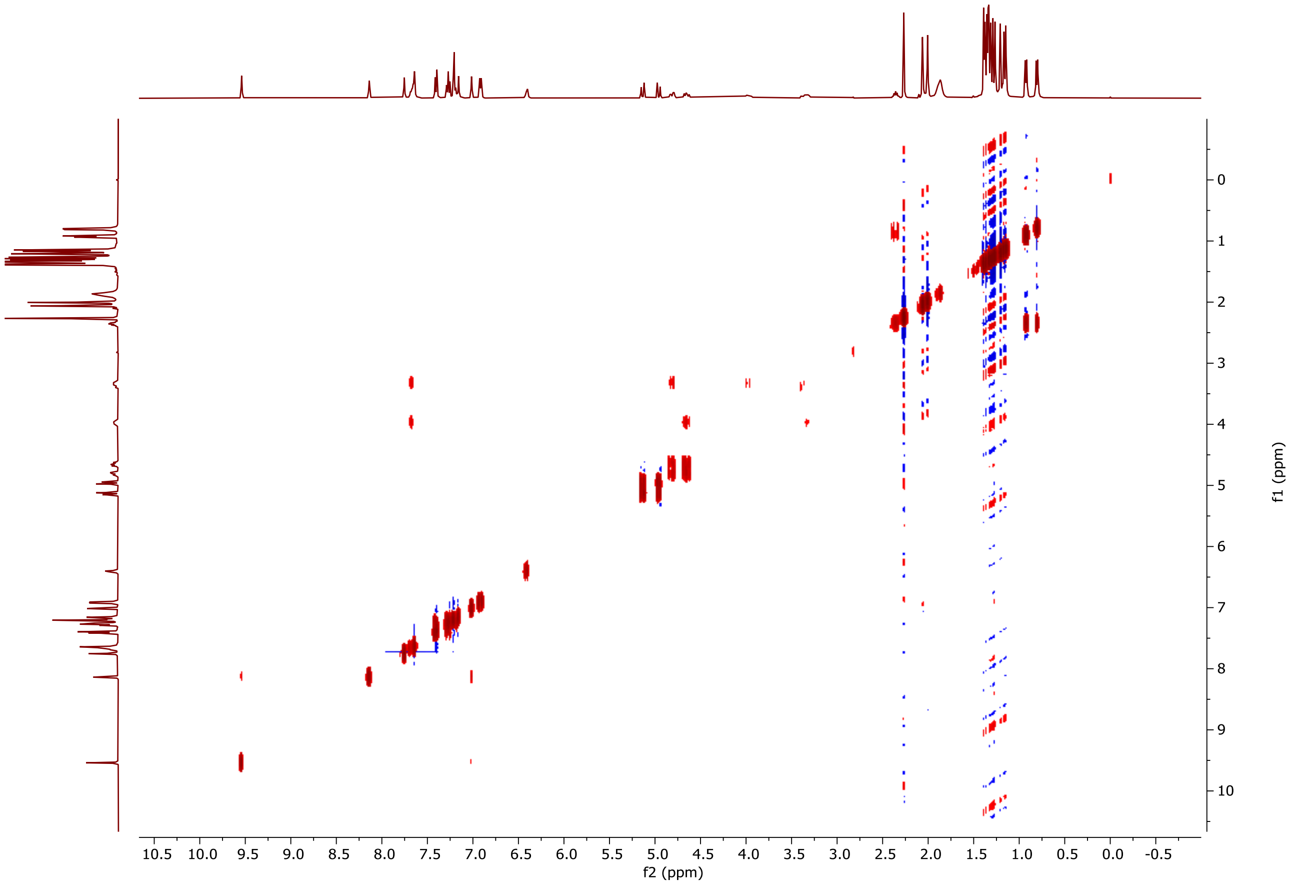
f2 (ppm)
f1 (ppm)

^{1}H ^{13}C HSQC

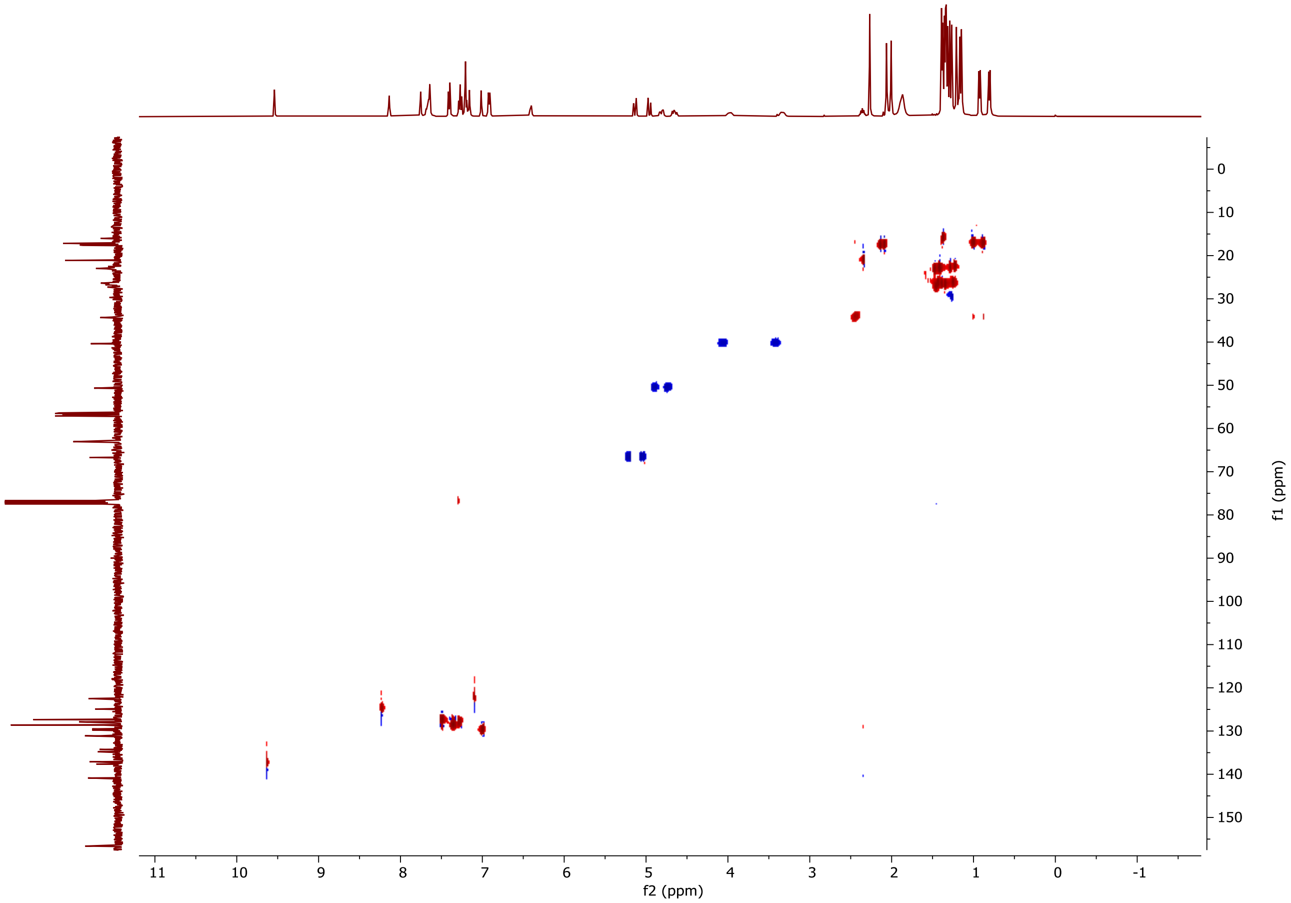

^{1}H ^{13}C HMBC

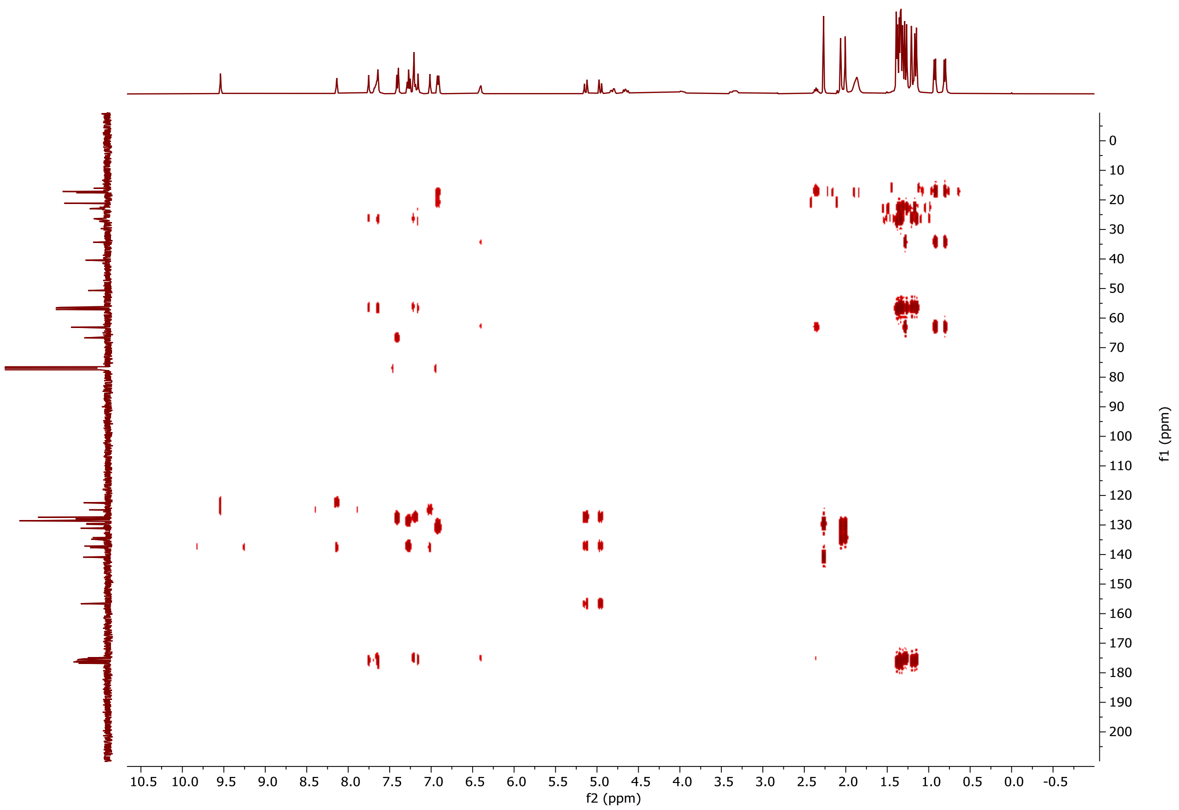

4.24 Foldamer 19 [(Cbz-(L-αMeVal)Aib$_5$NH(CH$_2$)$_2$-NHC-Mes)Rh(Cl)(COD)]

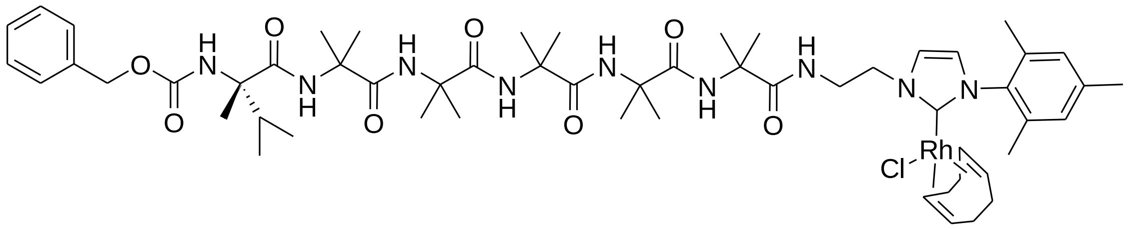

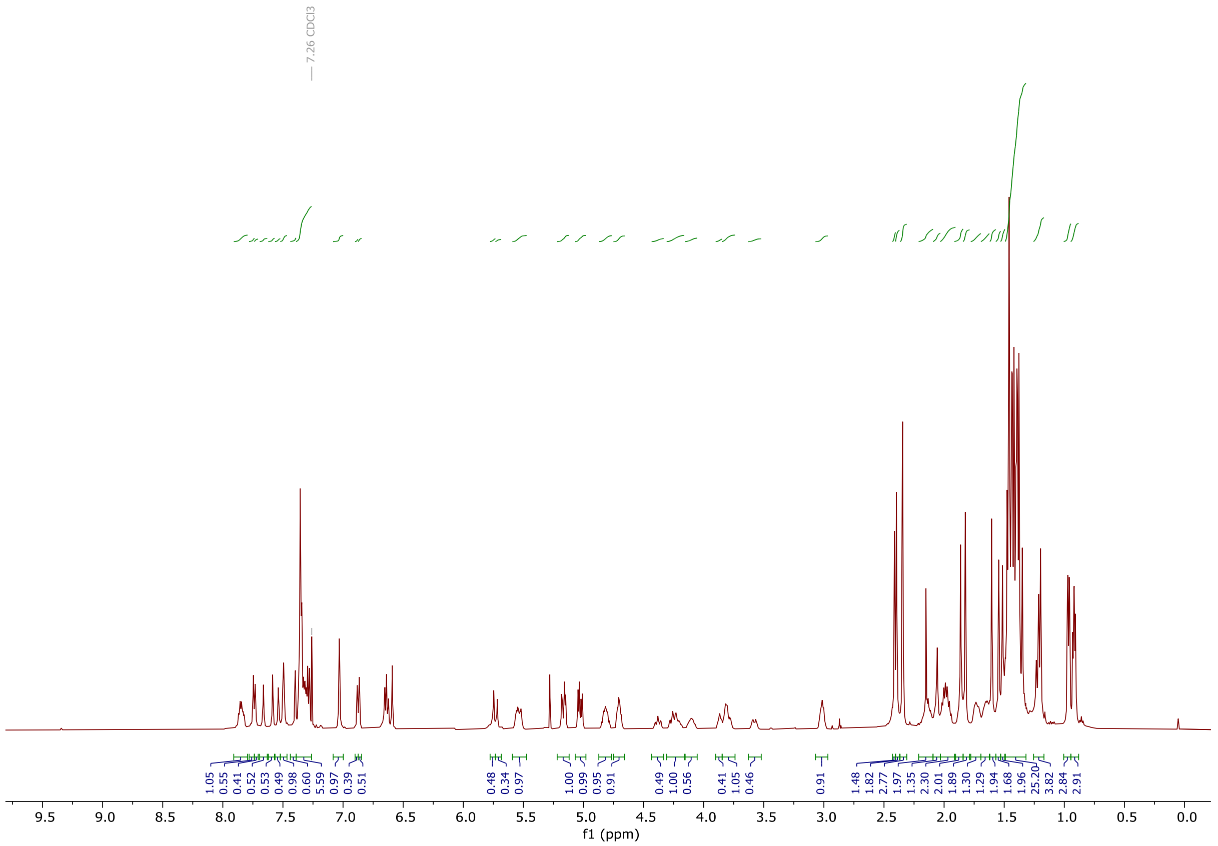

Line fitting of the ^{1}H NMR C*H*$_{Imid}$ signal to determine the diastereomeric ratio.

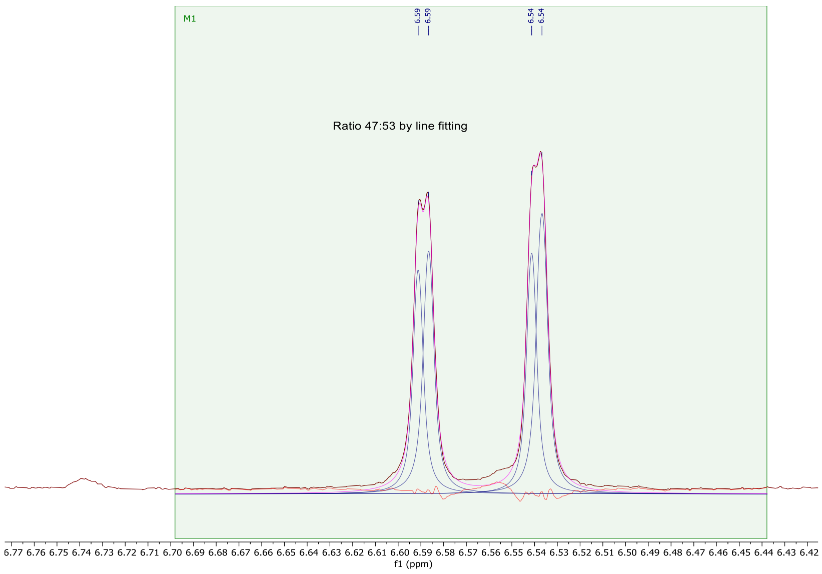

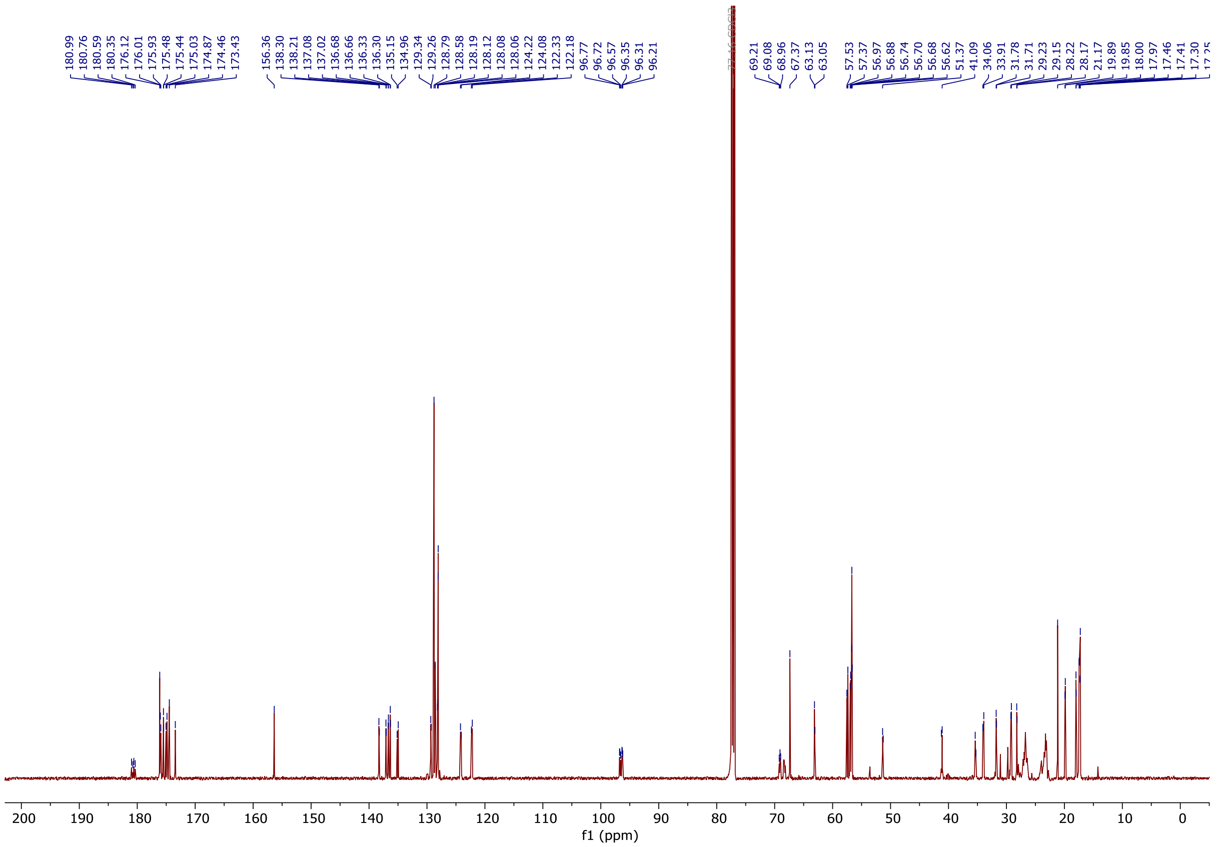
180.99
180.76
180.59
180.35
176.12
176.01
175.93
175.48
175.44
175.03
174.87
174.46
173.43
156.36
138.30
138.21
137.08
137.02
136.68
136.66
136.33
136.30
135.15
134.96
129.34
129.26
128.79
128.58
128.19
128.12
128.08
128.06
124.22
124.08
122.33
122.18
96.77
96.72
96.57
96.35
96.31
96.21
69.21
69.08
68.96
67.37
63.13
63.05
57.53
57.37
56.97
56.88
56.74
56.70
56.68
56.62
51.37
41.09
34.06
33.91
31.78
31.71
29.23
29.15
28.22
28.17
21.17
19.89
19.85
18.00
17.97
17.46
17.41
17.30
f1 (ppm)

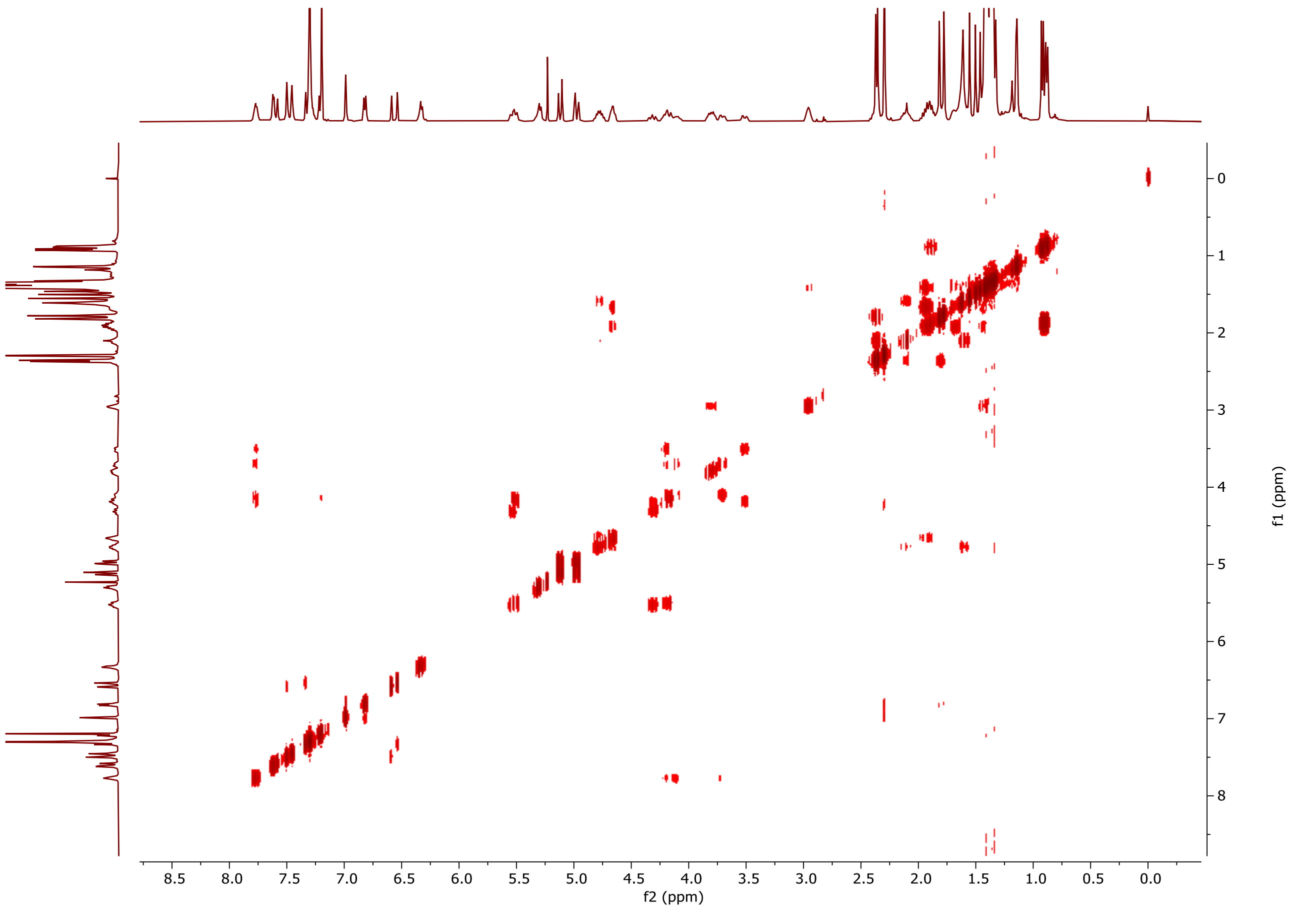
f2 (ppm)
f1 (ppm)

^1H ^{13}C HSQC

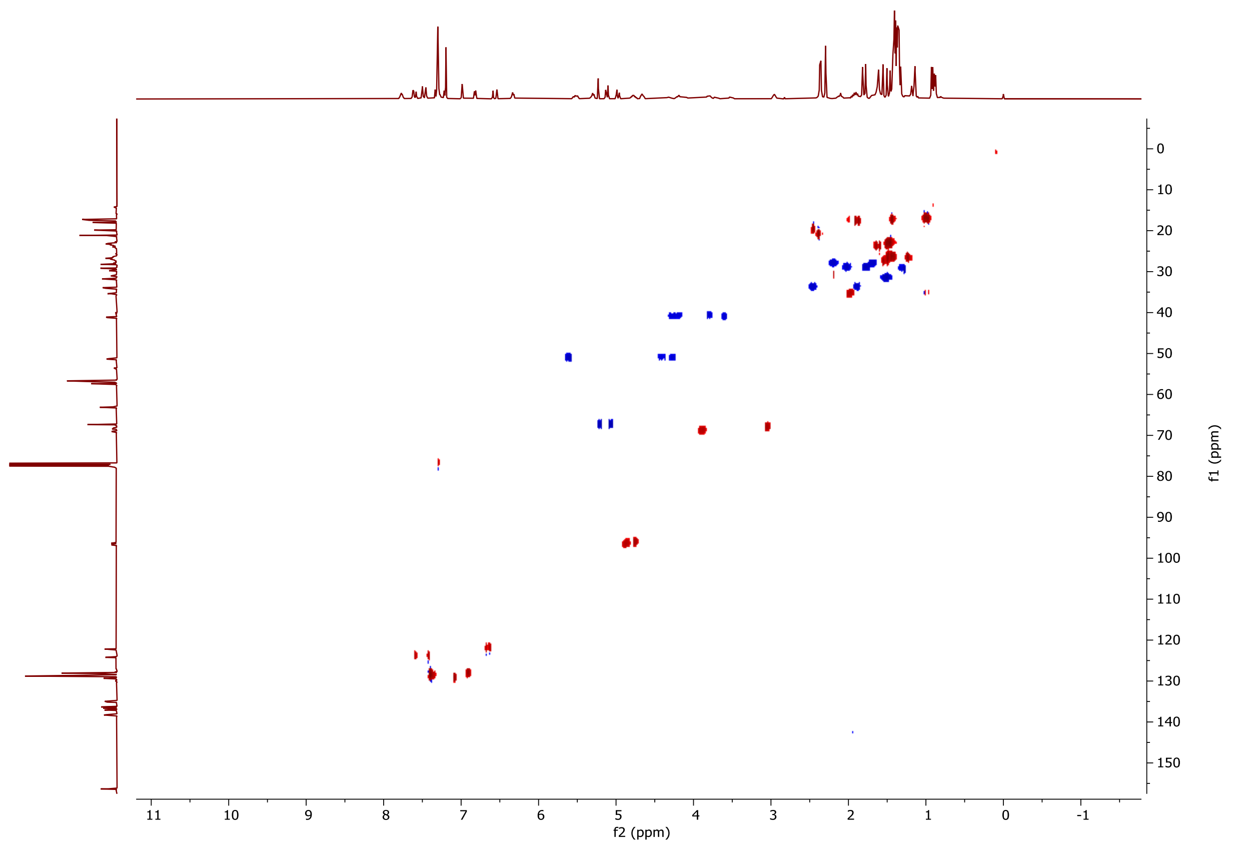

^1H ^{13}C HMBC

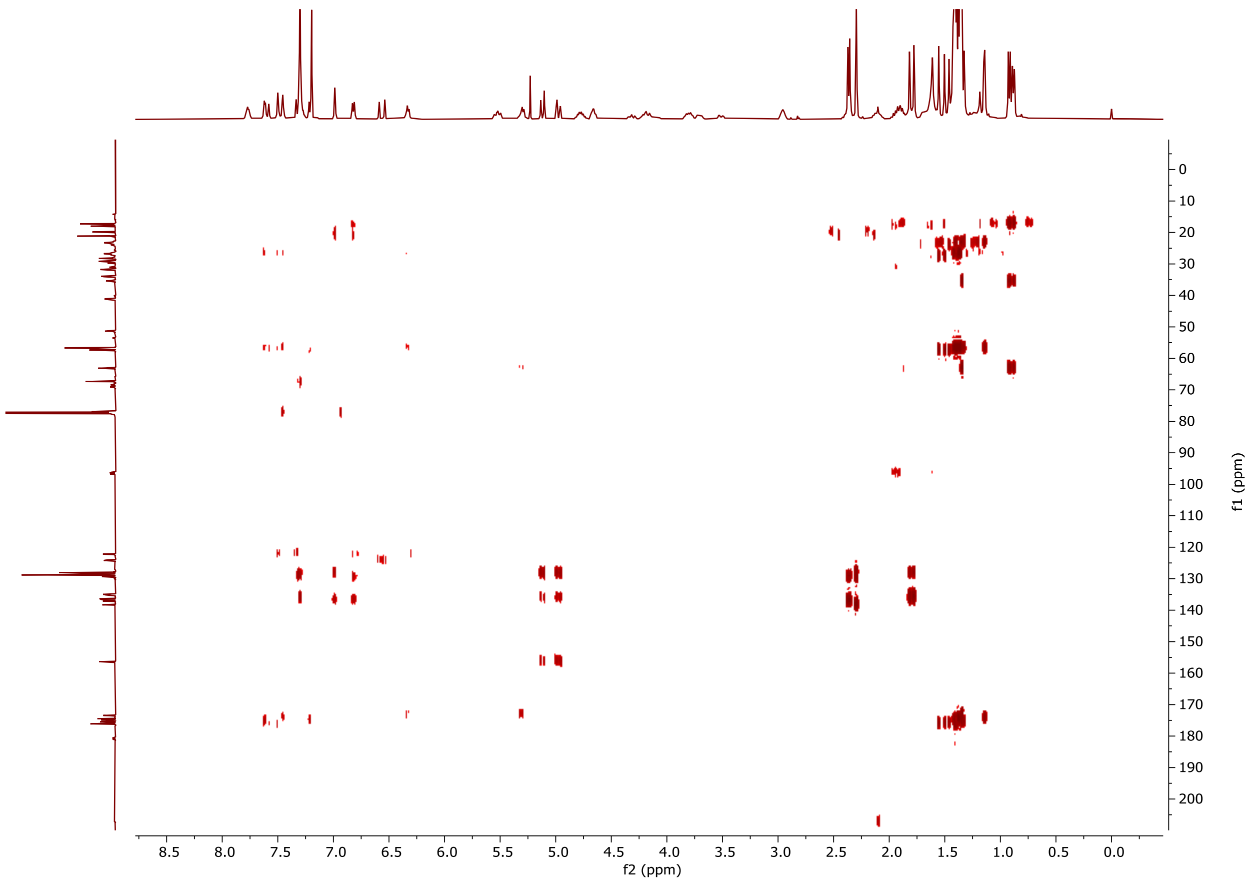

4.25 Intermediate [(+)-Mosher-NH(CH$_2$)$_2$(Im-Mes)]$^+$Br$^-$

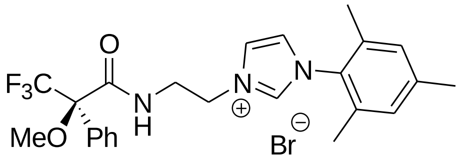

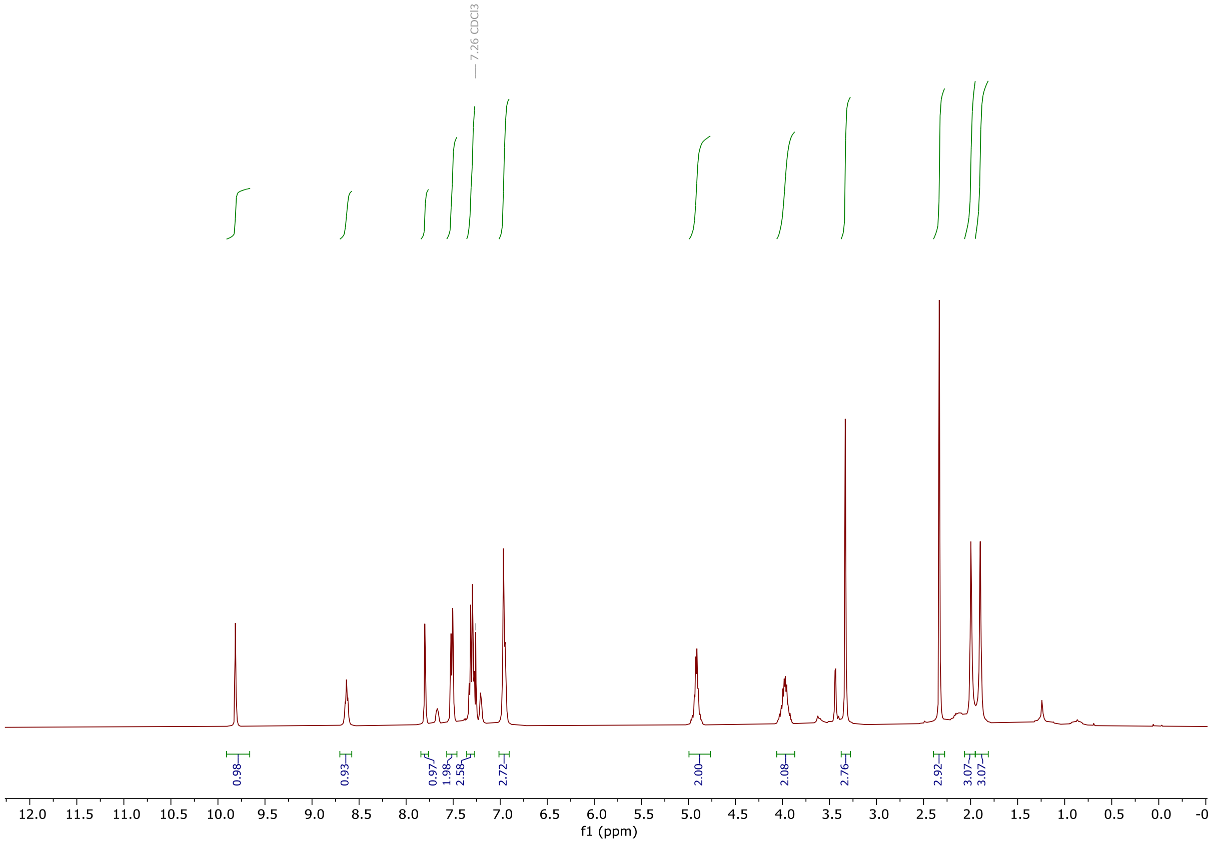

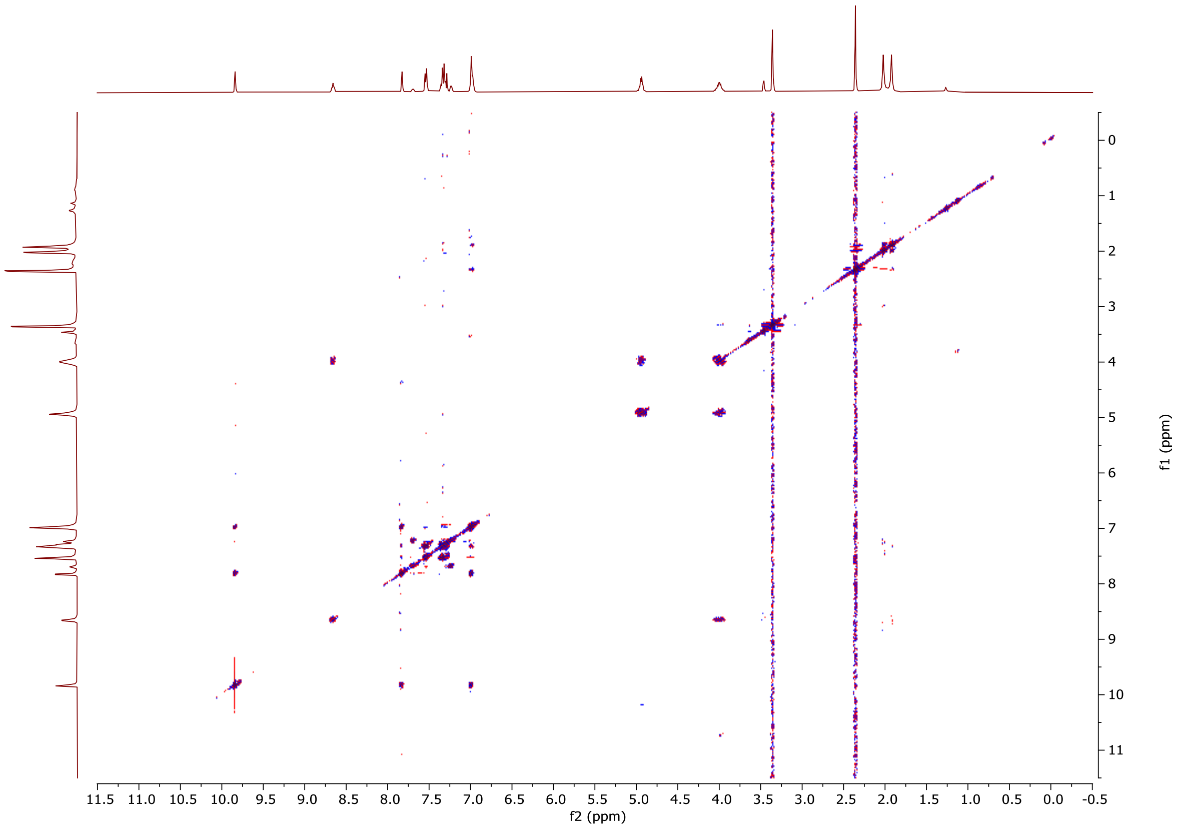

11.5 11.0 10.5 10.0 9.5 9.0 8.5 8.0 7.5 7.0 6.5 6.0 5.5 5.0 4.5 4.0 3.5 3.0 2.5 2.0 1.5 1.0 0.5 0.0 -0.5
f2 (ppm)
0 1 2 3 4 5 6 7 8 9 10 11
f1 (ppm)

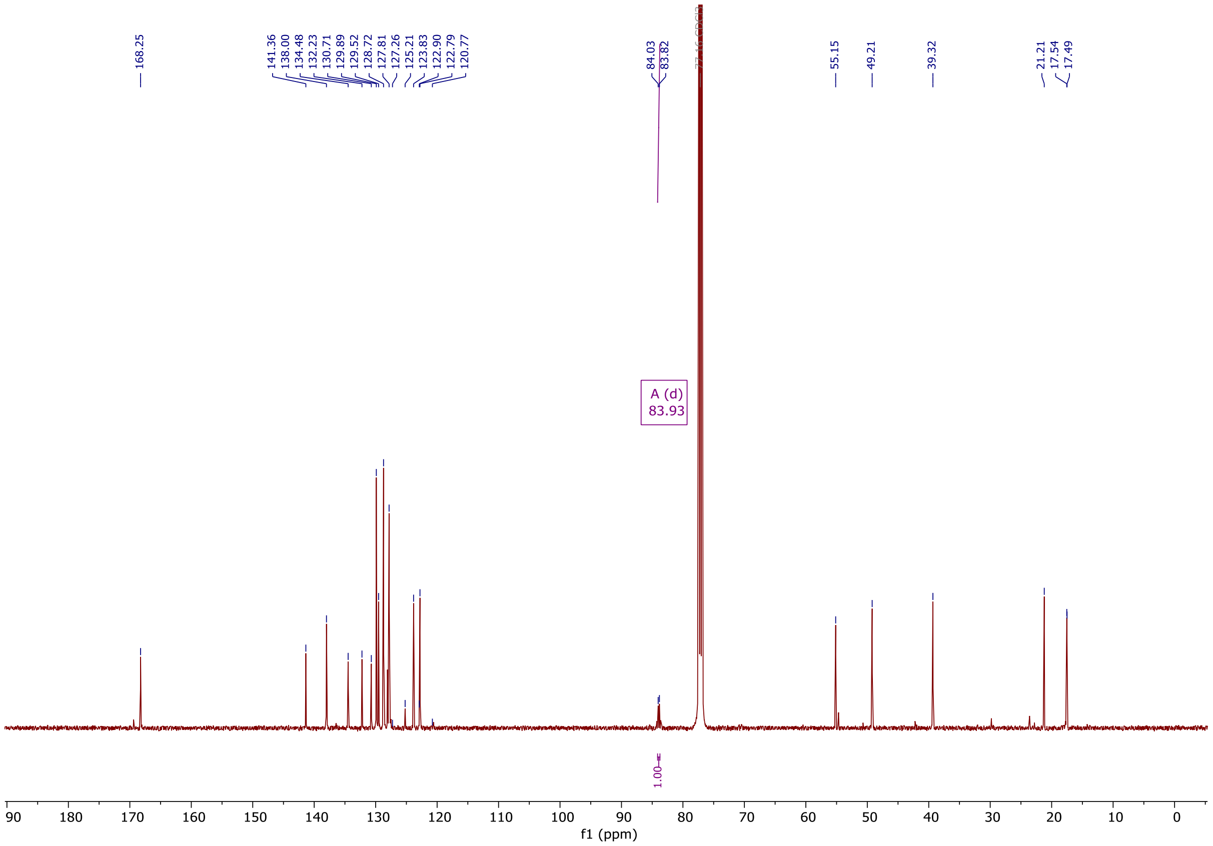

168.25
141.36 138.00 134.48 132.23 130.71 129.89 129.52 128.72 127.81 127.26 125.21 123.83 122.90 122.79 120.77
84.03 83.82
55.15
49.21
39.32
21.21 17.54 17.49
A (d)
83.93
1.00
90 180 170 160 150 140 130 120 110 100 90 80 70 60 50 40 30 20 10 0
f1 (ppm)

^{1}H ^{13}C HSQC

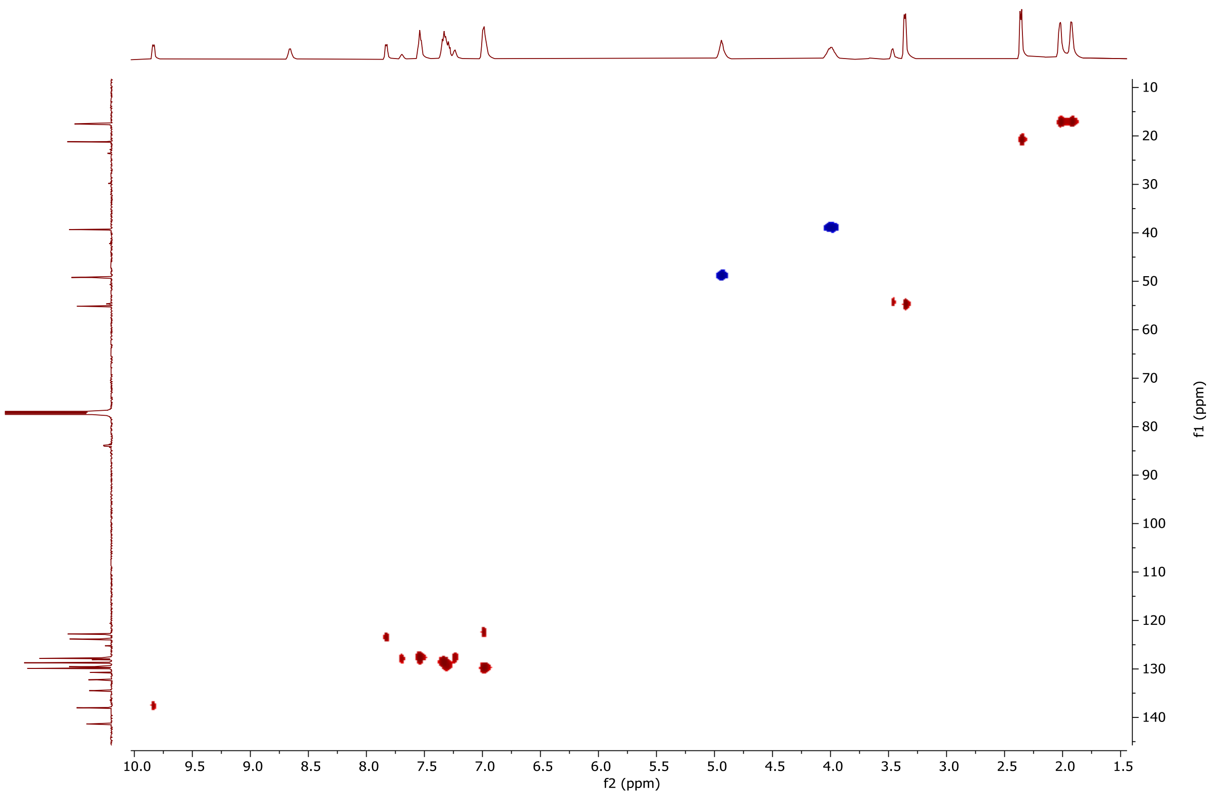

^{1}H ^{13}C HMBC

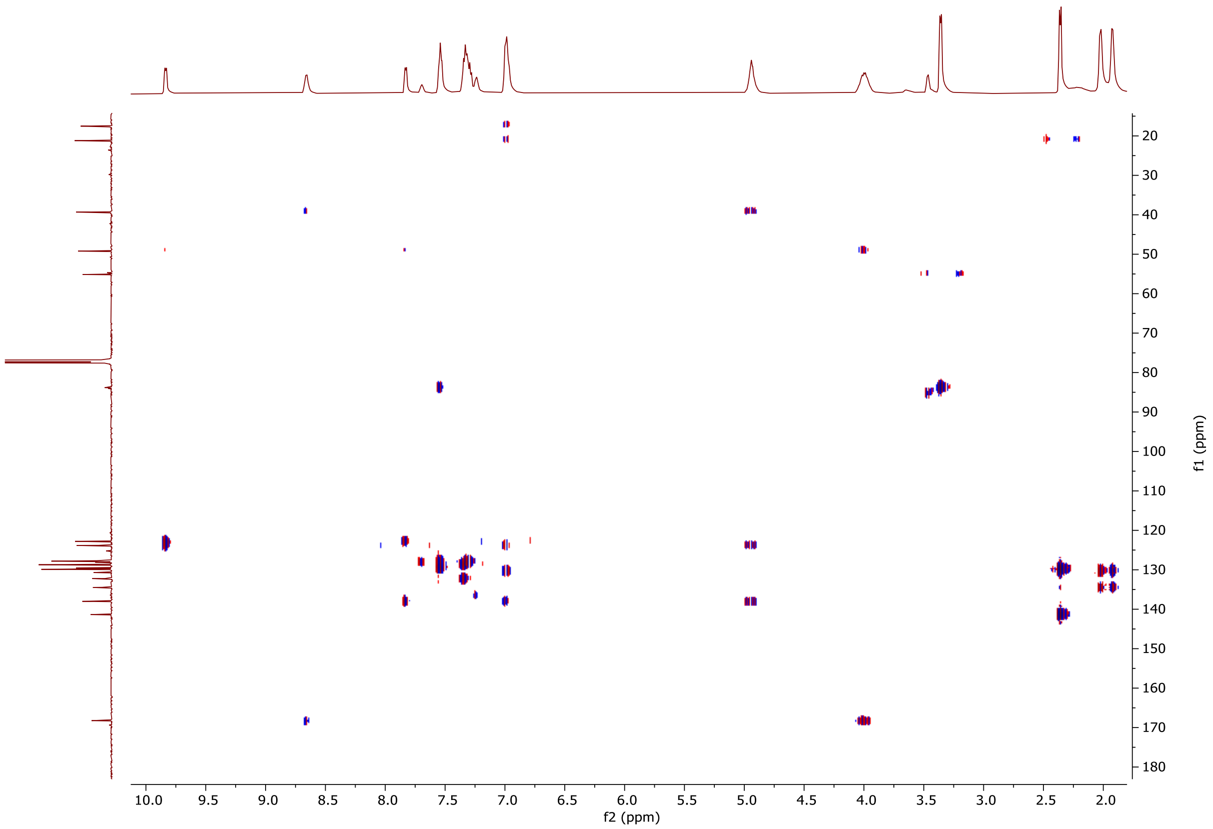

4.26 Foldamer 20 [(+)-MosherNH(CH₂)₂-NHC-Mes)Rh(Cl)(COD)]

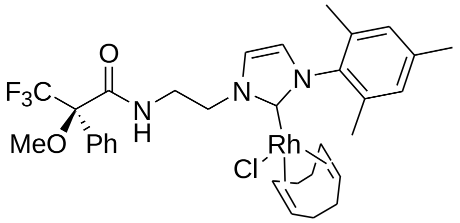

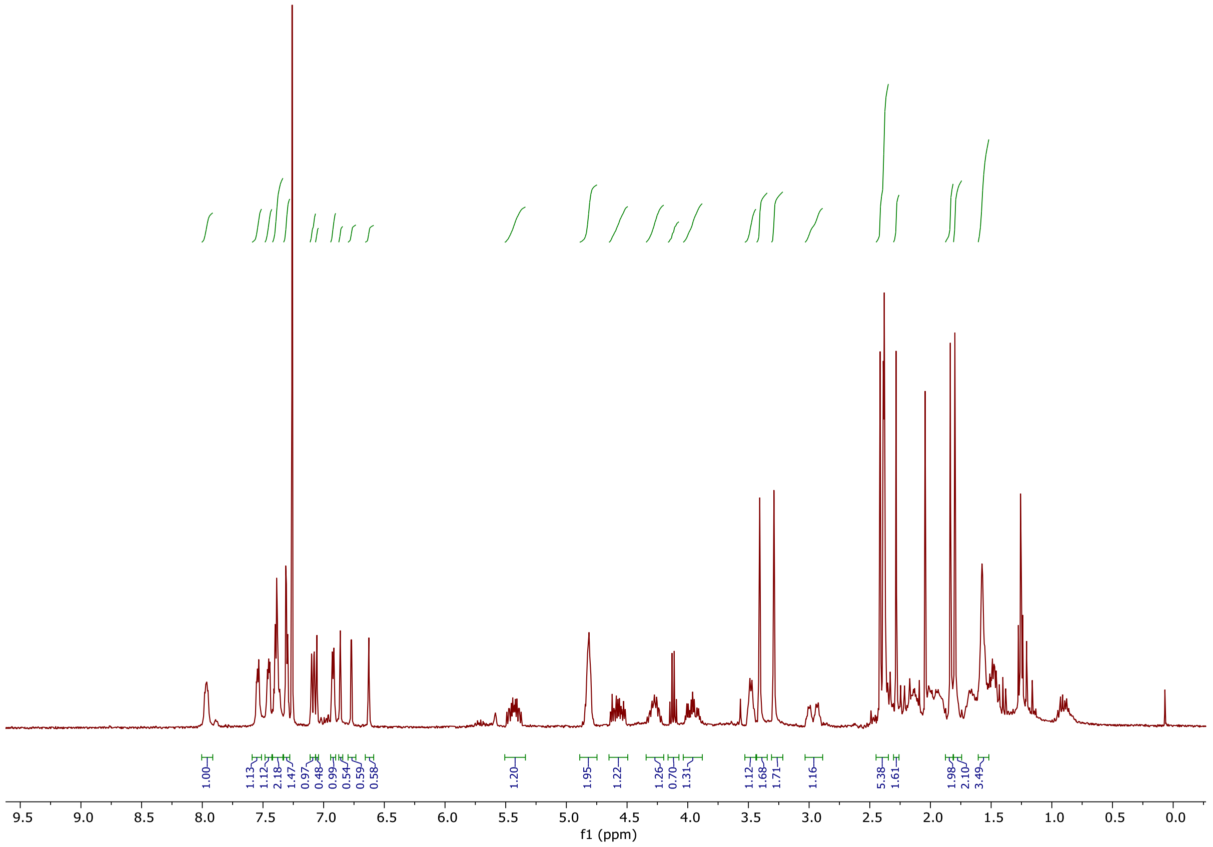

Expansion of ^{1}H NMR spectrum (CH_{imid} signals) to determine the diastereomeric ratio.

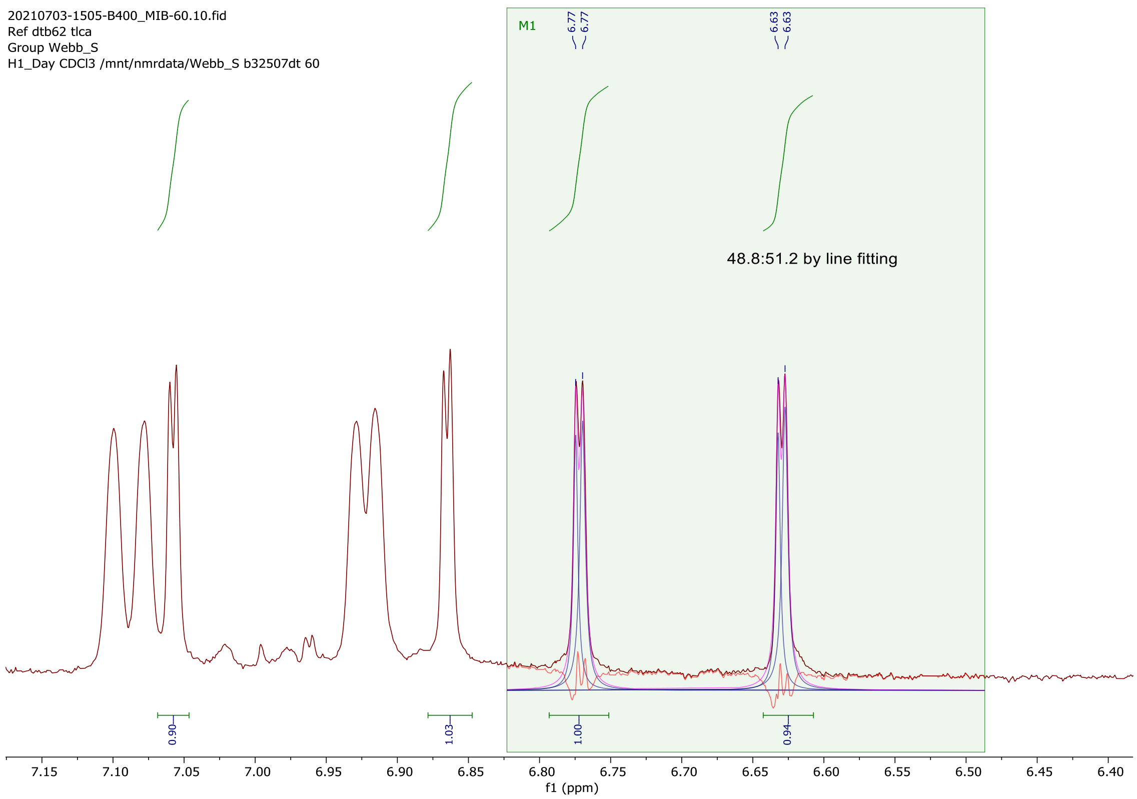

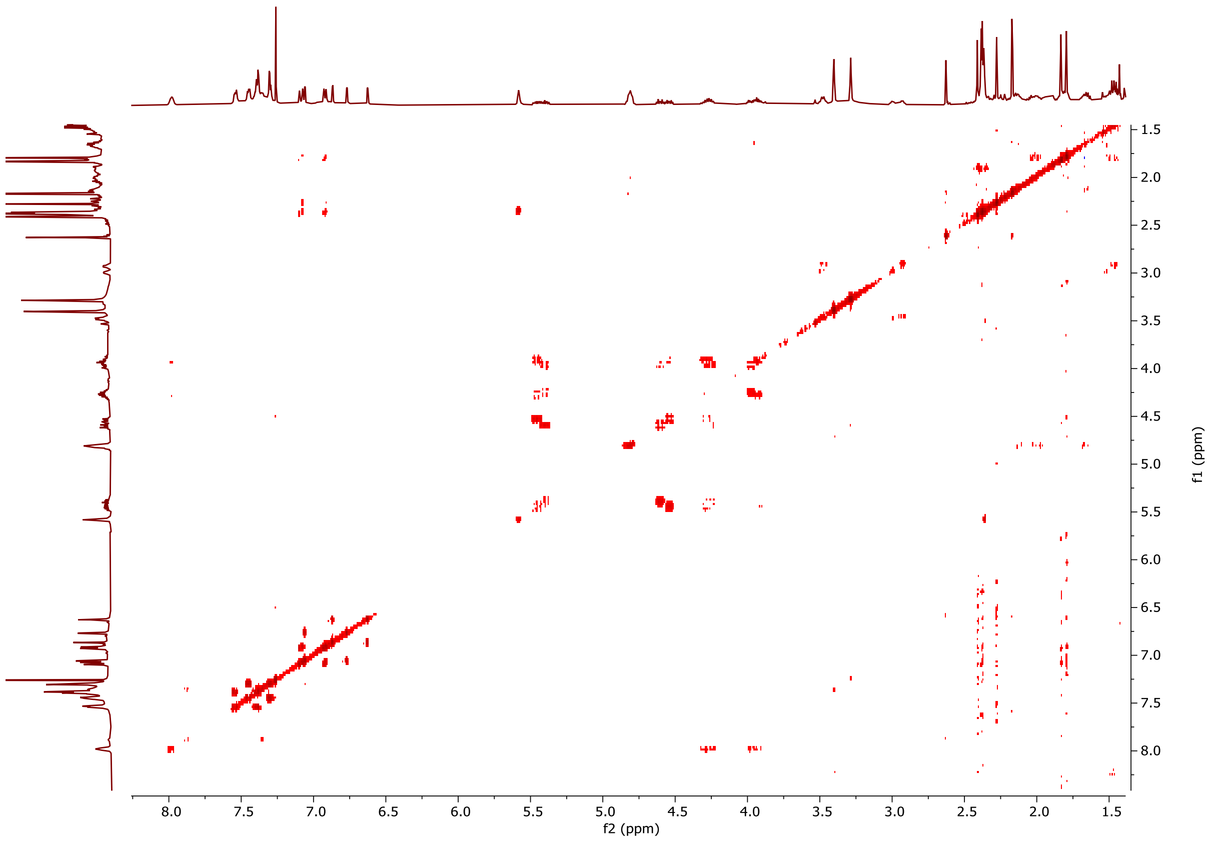

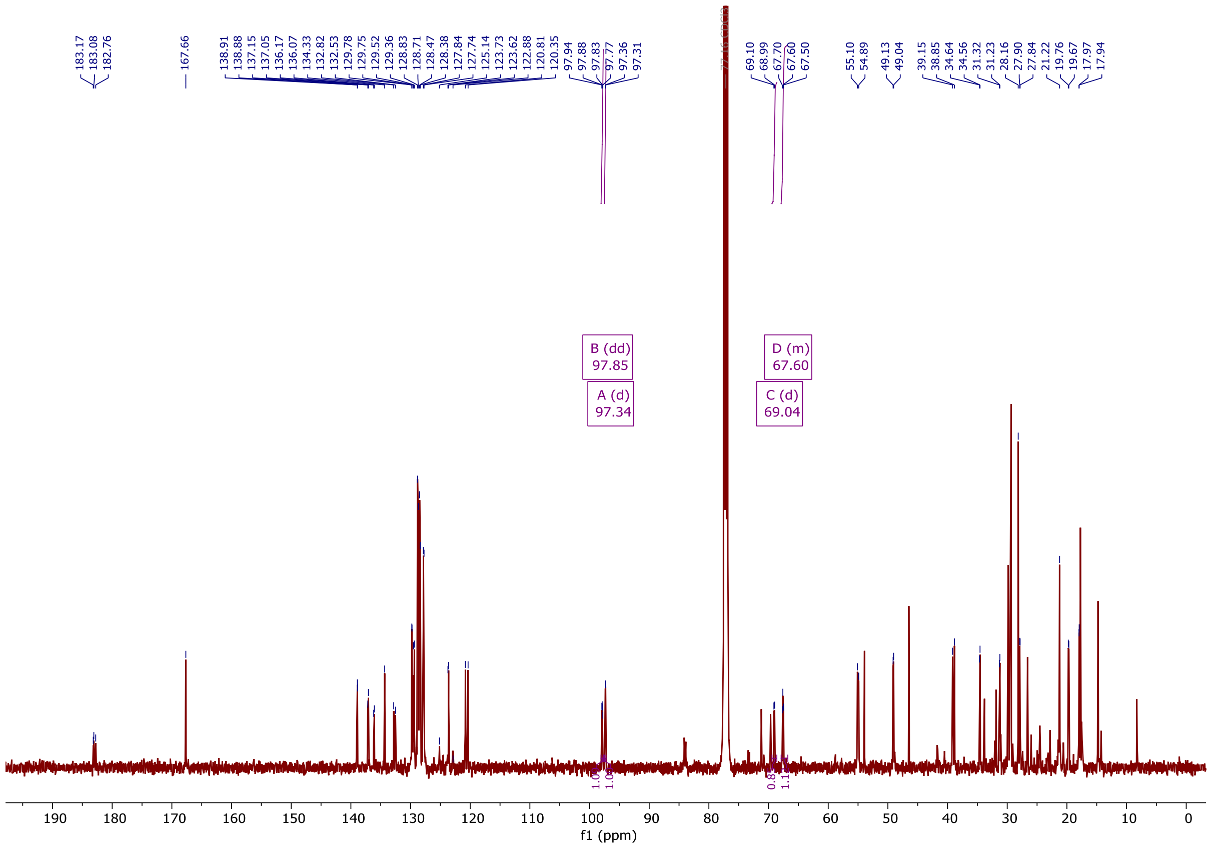
183.17
183.08
182.76
167.66
138.91
138.88
137.15
137.05
136.17
136.07
134.33
132.82
132.53
129.78
129.75
129.52
129.36
128.83
128.71
128.47
128.38
127.84
127.74
125.14
123.73
123.62
122.88
120.81
120.35
97.94
97.88
97.83
97.77
97.36
97.31
69.10
68.99
67.70
67.60
67.50
55.10
54.89
49.13
49.04
39.15
38.85
34.64
34.56
31.32
31.23
28.16
27.90
27.84
21.22
19.76
19.67
17.97
17.94
B (dd) 97.85
A (d) 97.34
D (m) 67.60
C (d) 69.04
f1 (ppm)

^{1}H ^{13}C HSQC

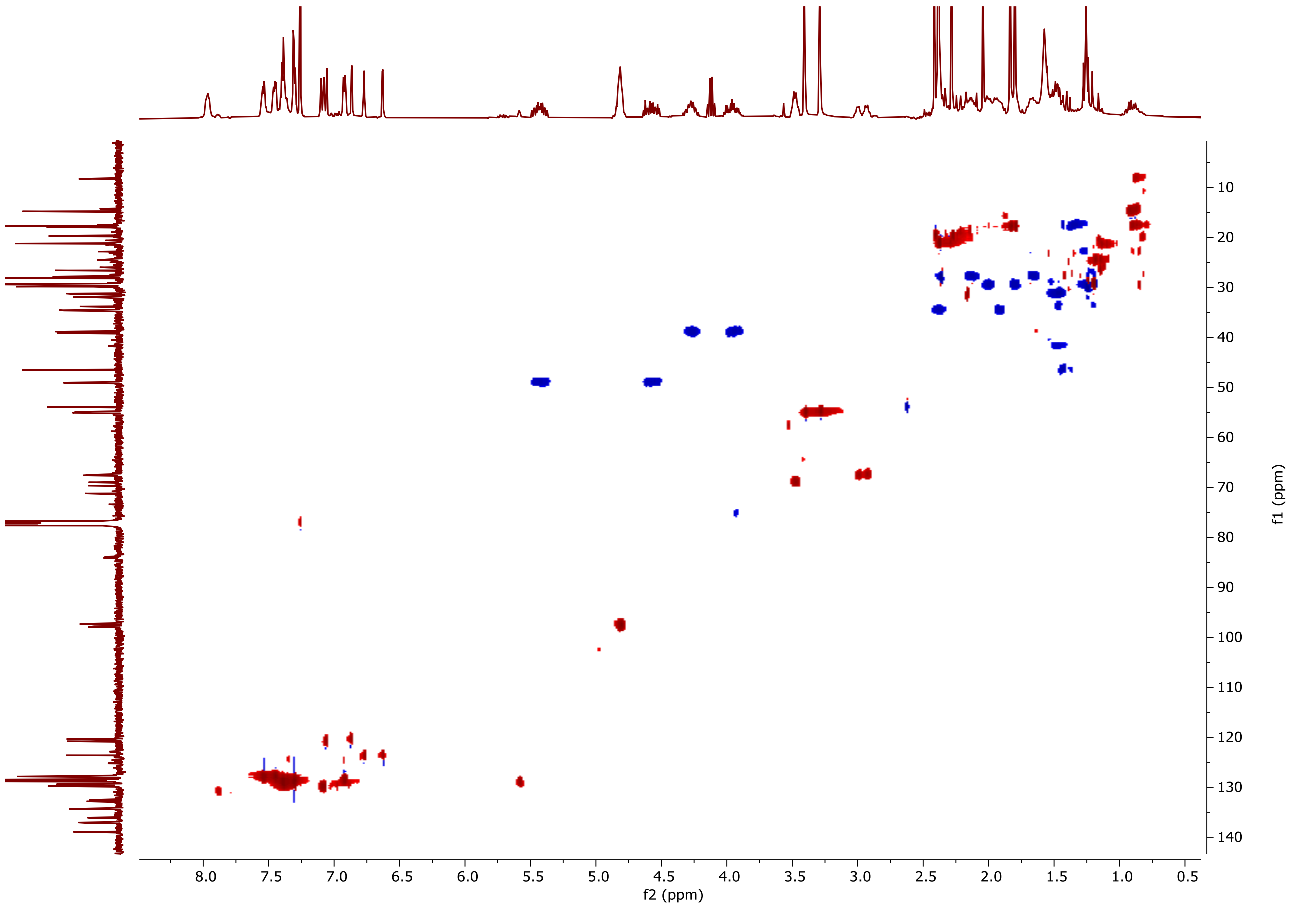
f2 (ppm)
f1 (ppm)

^{1}H ^{13}C HMBC

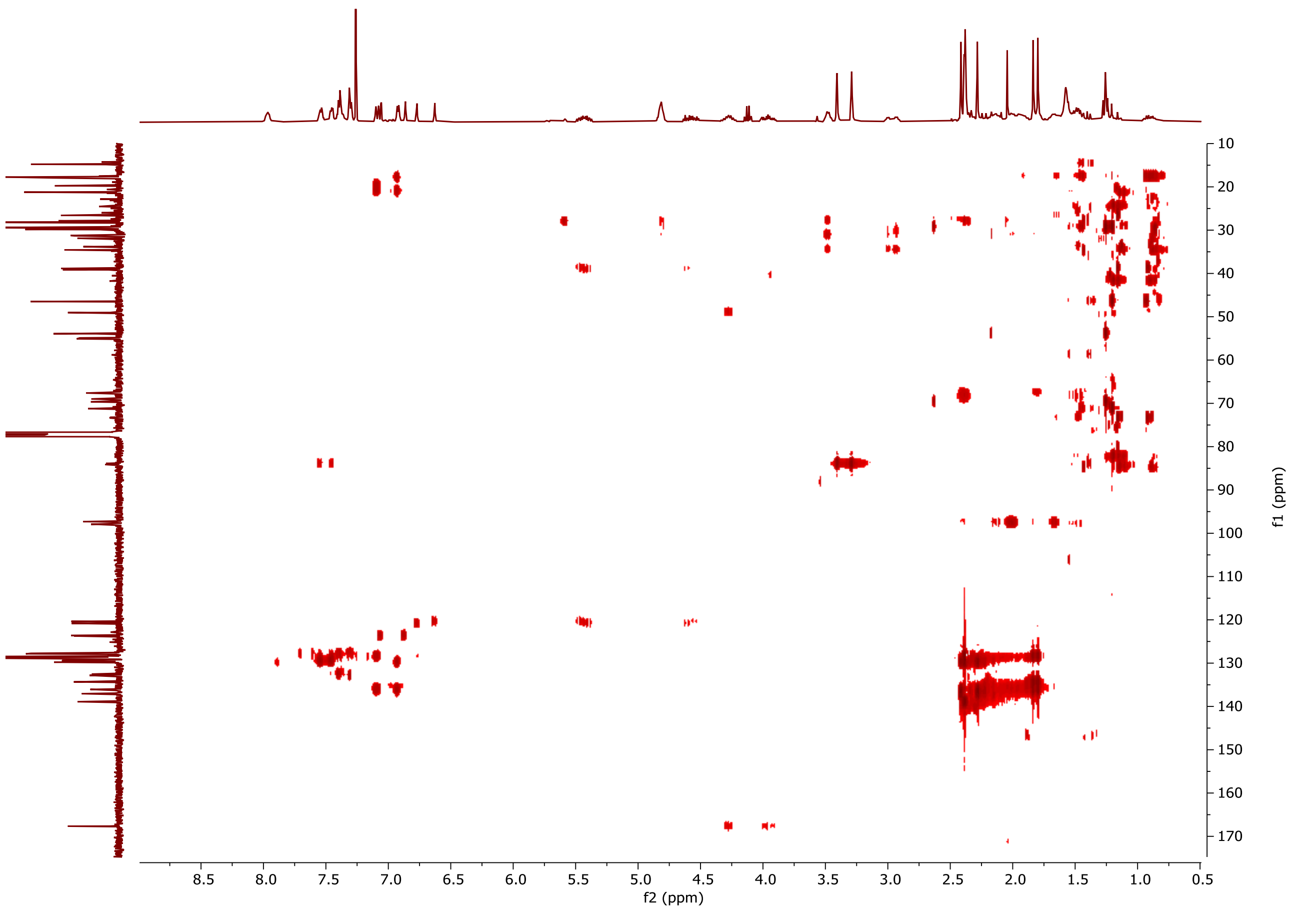

^{19}F NMR

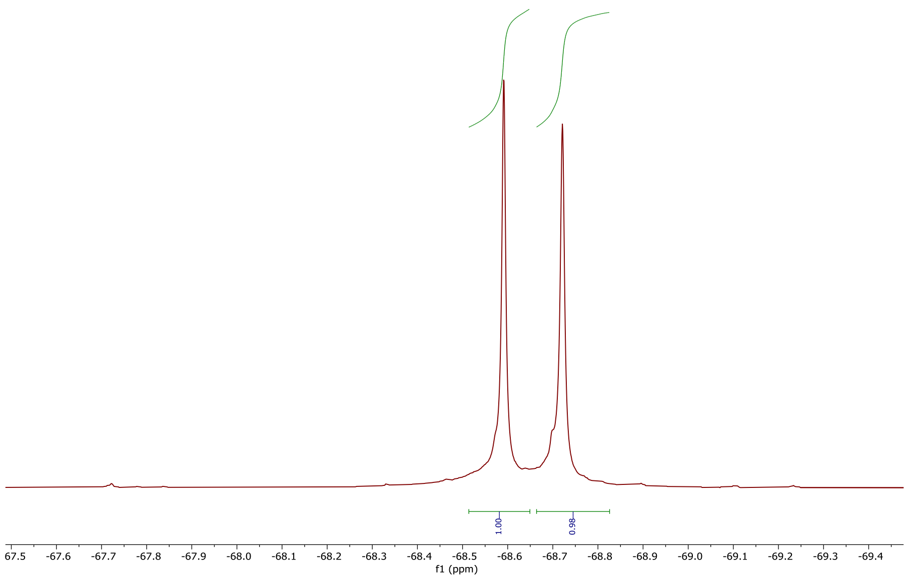

5. Variable temperature ^{1}H NMR studies (VT-NMR)

5.1 Procedure

In a dry NMR tube, the foldamer **15** (10 mg) was dissolved in 0.5 mL of deuterated solvent (chloroform for VT-NMR between −40 °C to +50 °C, or tetrachloroethane CD_2Cl_4 for VT-NMR between −20 °C to +80 °C). Variable temperature ^{1}H NMR experiments were recorded on a 500 MHz Bruker Advance II apparatus with temperatures varying with 10 °C increments between each measurement.

5.2 Fitting

Line fitting was carried on the resonances between 8.0 and 8.3 ppm. The first of these arises from the *ortho* phenyl protons on the NHC that give a doublet from each diastereoisomer (^3J ~ 7.8 Hz, with smaller J couplings evident at higher temperatures), which coalesce at *ca*. 20 °C. The other resonance in this region is an apparent singlet that arises from the NH protons on each diastereoisomer (two signals that overlap at all temperatures). Fitting was perfomed using the DNMR module within TopSpin 4.1.1 (Bruker). The model had two spin systems, one of which had two exchanging molecules (representing each diastereoisomer) in a 55:45 ratio. The NH resonances (an apparent singlet) were represented by another spin system. The line broadening was initially set at 1.1 Hz and estimates of chemical shifts for the resonances were taken from the low temperature spectra. The exchange constants (k) were initially estimated for each temperature then allowed to minimise (1000 steps or until convergance was reached). Overlap values were between 93.1% and 96.5% except for the highest temperature of 80 °C, which gave 91.9% overlap.

Representative fitted resonances are shown in Figure S1(a-k).

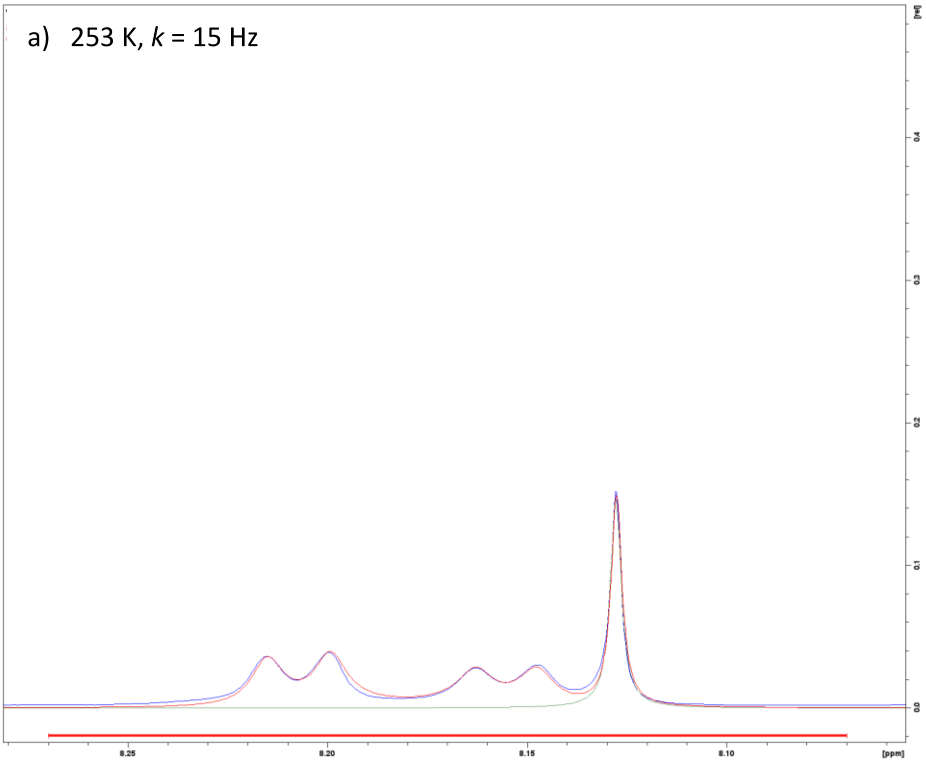
a) 253 K, k = 15 Hz

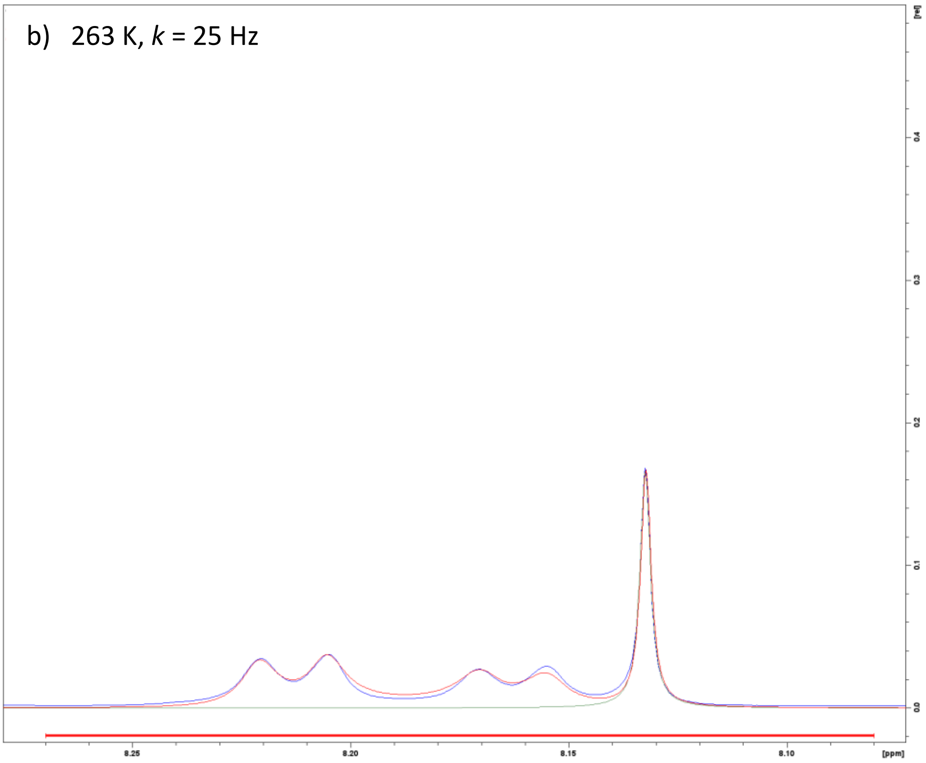
b) 263 K, k = 25 Hz

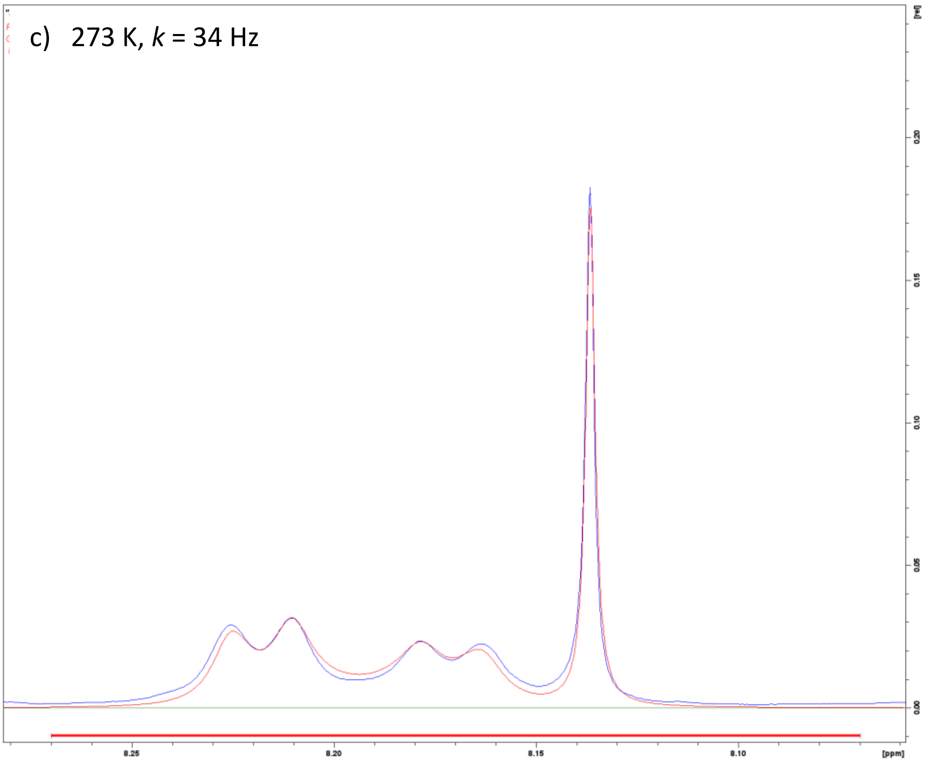
c) 273 K, *k* = 34 Hz
8.25
8.20
8.15
8.10
[ppm]
0.00
0.05
0.10
0.15
0.20
[rel]

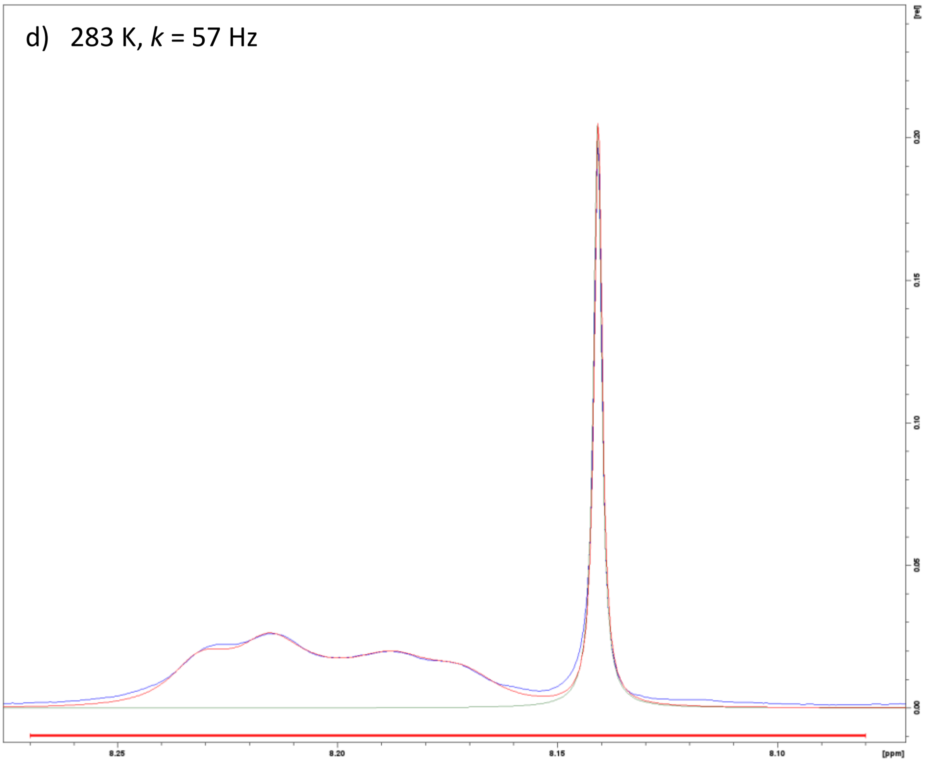
d) 283 K, *k* = 57 Hz
8.25
8.20
8.15
8.10
[ppm]
0.00
0.05
0.10
0.15
0.20
[rel]

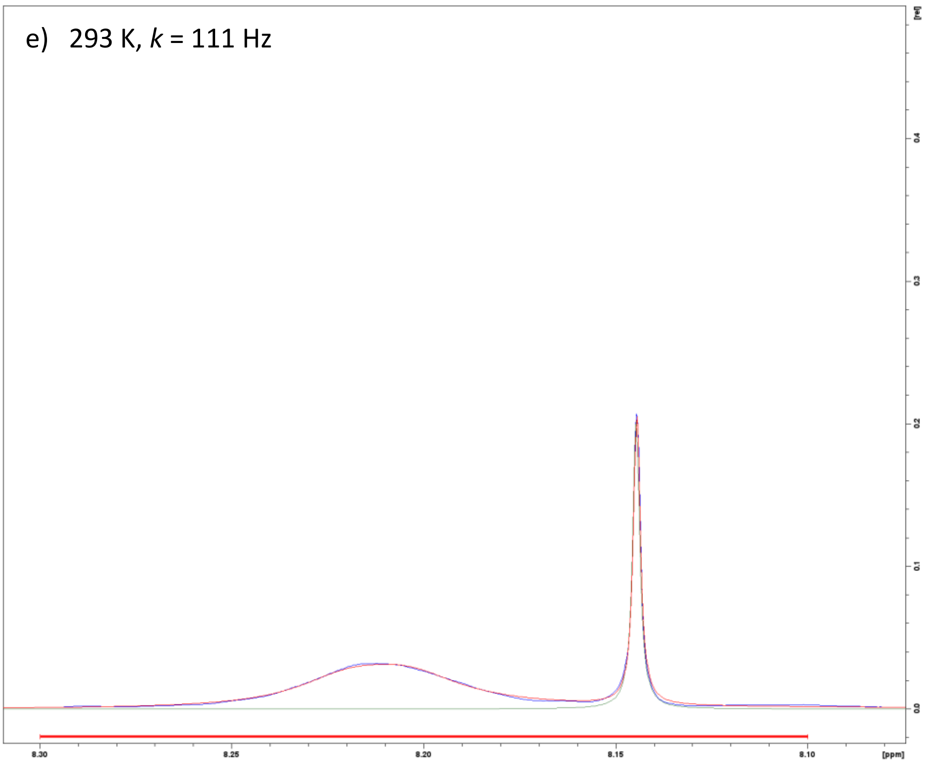
e) 293 K, *k* = 111 Hz
8.30
8.25
8.20
8.15
8.10
[ppm]
0.4
0.3
0.2
0.1
0.0
[rel]

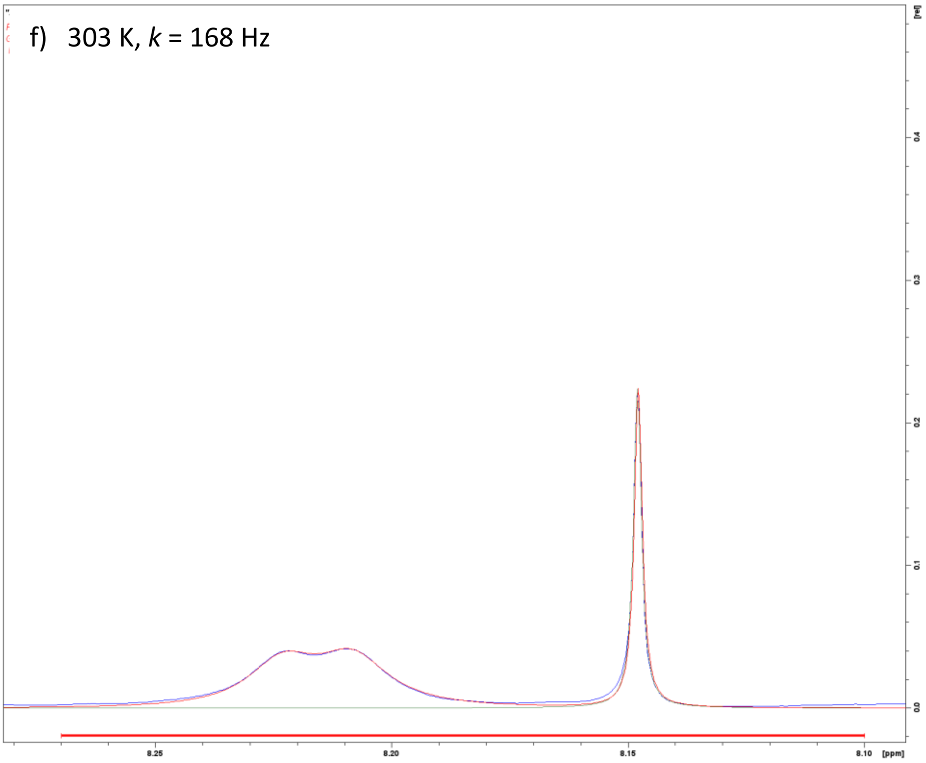
f) 303 K, *k* = 168 Hz
8.25
8.20
8.15
8.10
[ppm]
0.4
0.3
0.2
0.1
0.0
[rel]

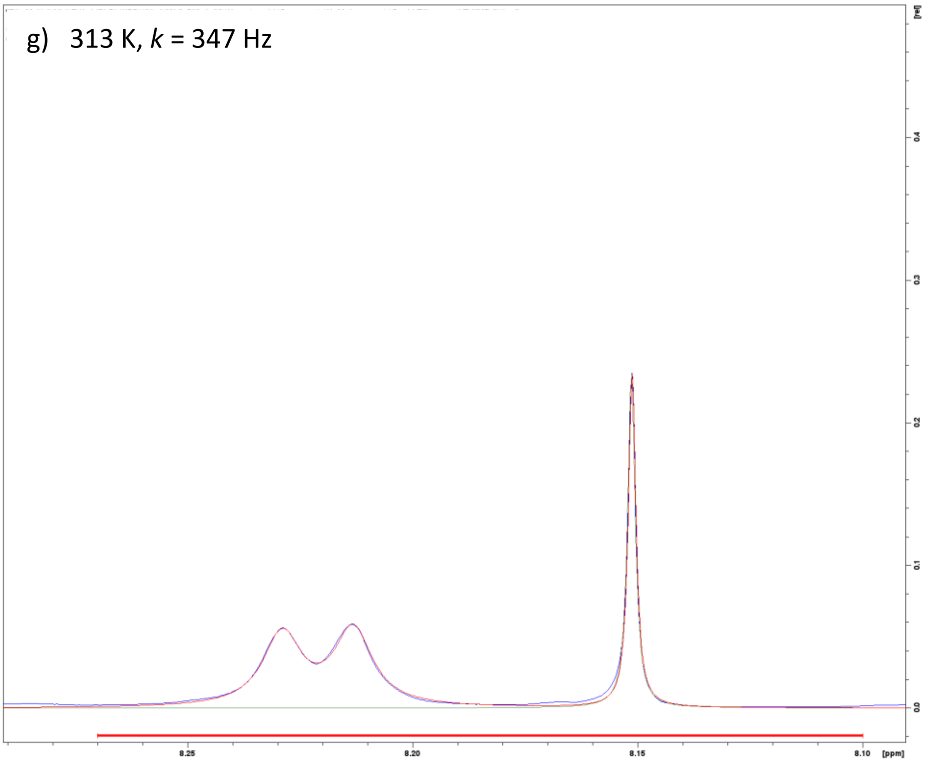
g) 313 K, k = 347 Hz
8.25
8.20
8.15
8.10
[ppm]

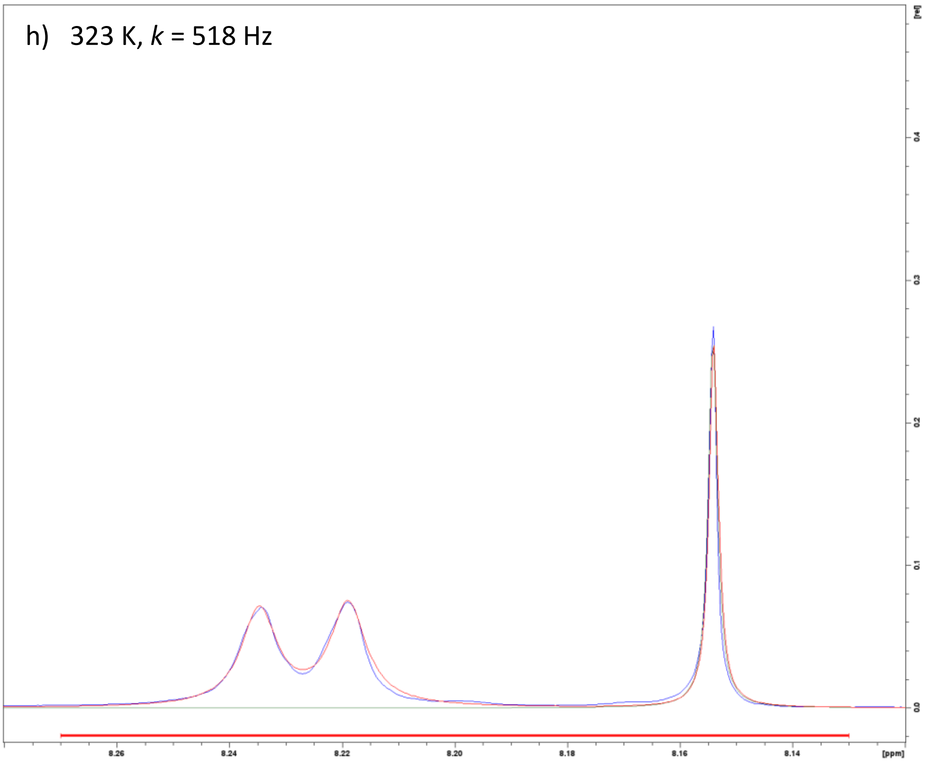
h) 323 K, k = 518 Hz
8.26
8.24
8.22
8.20
8.18
8.16
8.14
[ppm]

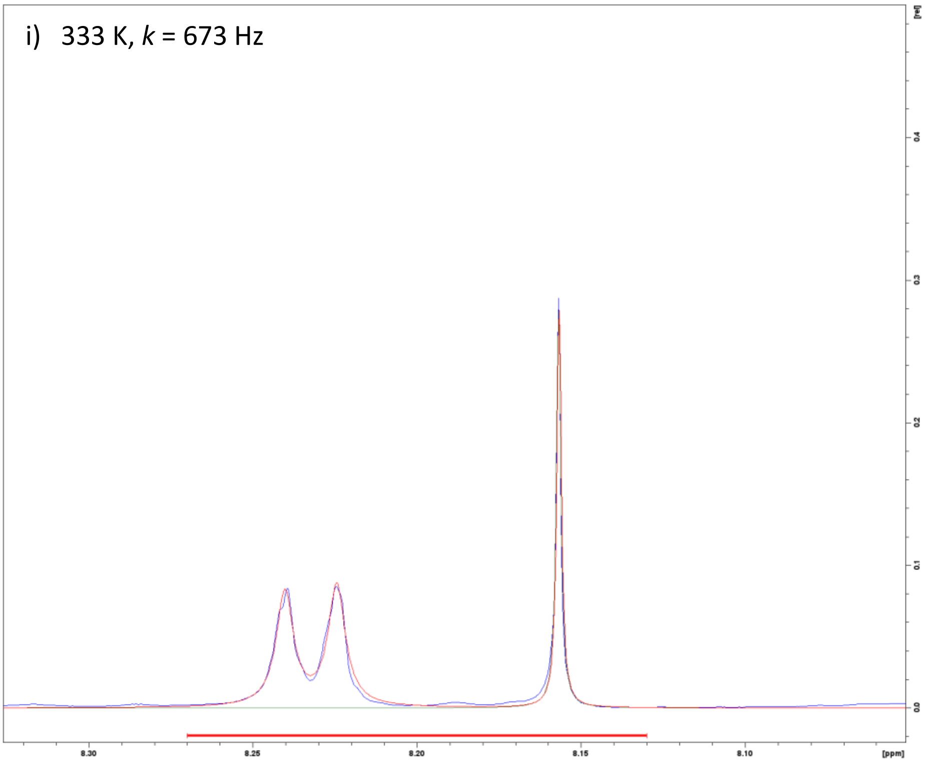
i) 333 K, k = 673 Hz

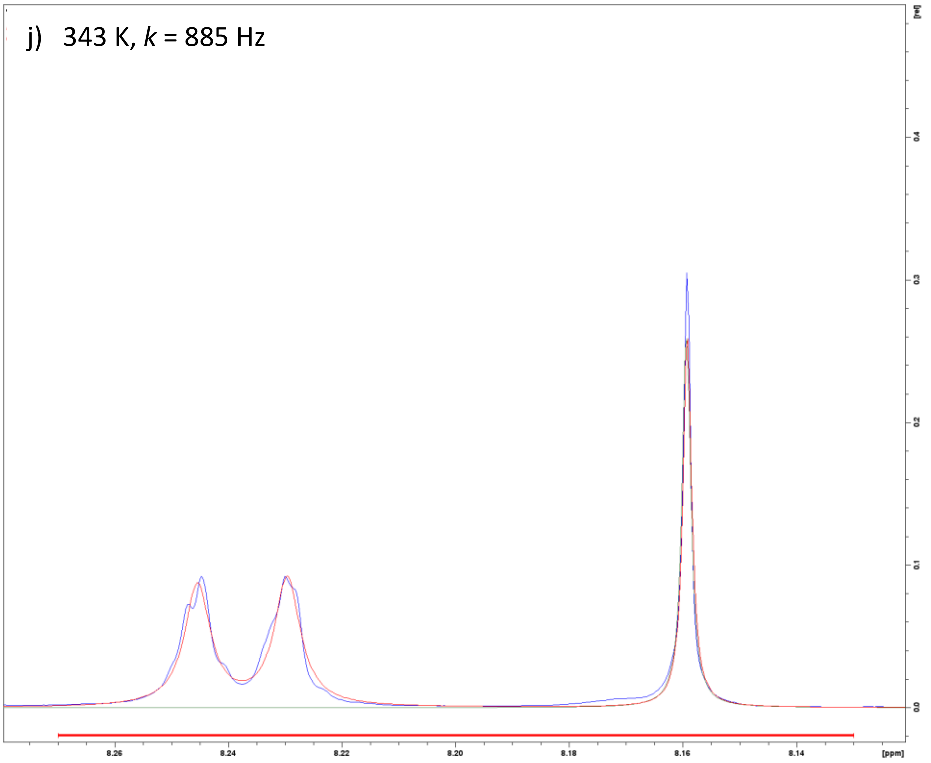
j) 343 K, k = 885 Hz

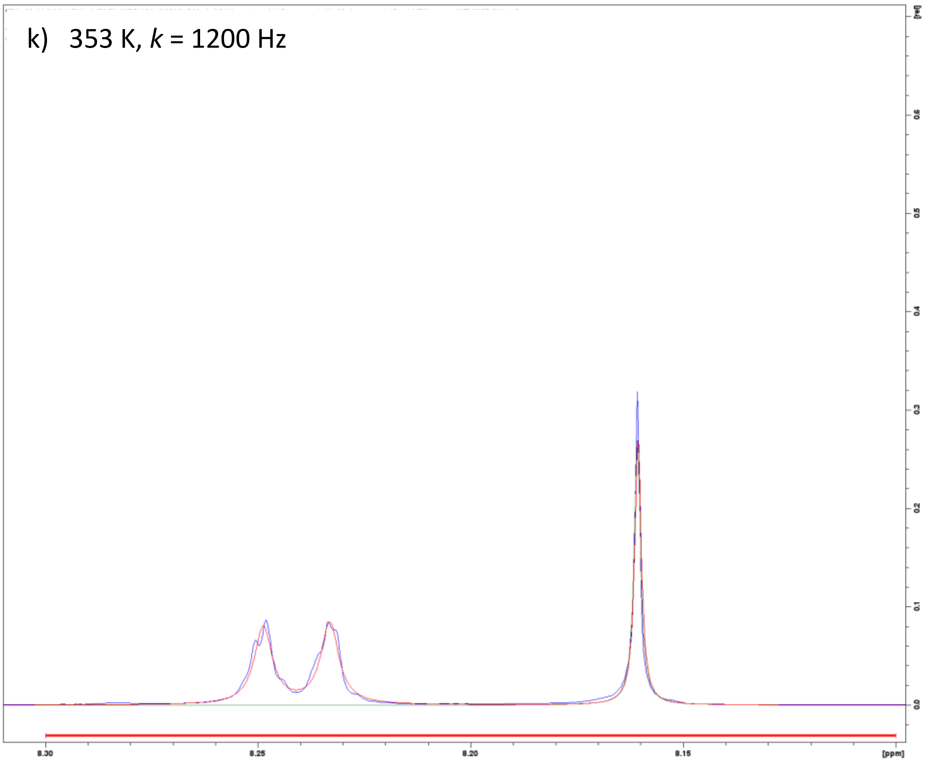

Figure S1: (a-j) Fitted VT-NMR resonances for *ortho* phenyl proton on the NHC and a singlet that arises from an NH proton on foldamer **14** from 253 to 353 K.

5.2.1 Procedure for determining the activation energy

The values of $\Delta H^{\ddagger}$, $\Delta G^{\ddagger}$, $\Delta S^{\ddagger}$ were calculated using the Eyring equation (Eqn. 1), using the experimentally determined values for k calculated from the fitting of the NMR line shapes.

$$ln(k/\mathrm{T}) = ln(k_b/h) - \Delta H^{\ddagger}/\mathrm{RT} + \Delta S^{\ddagger}/\mathrm{R} \qquad \text{Eqn. 1}$$

where k_{b} is the Boltzmann constant and h is the Planck constant.

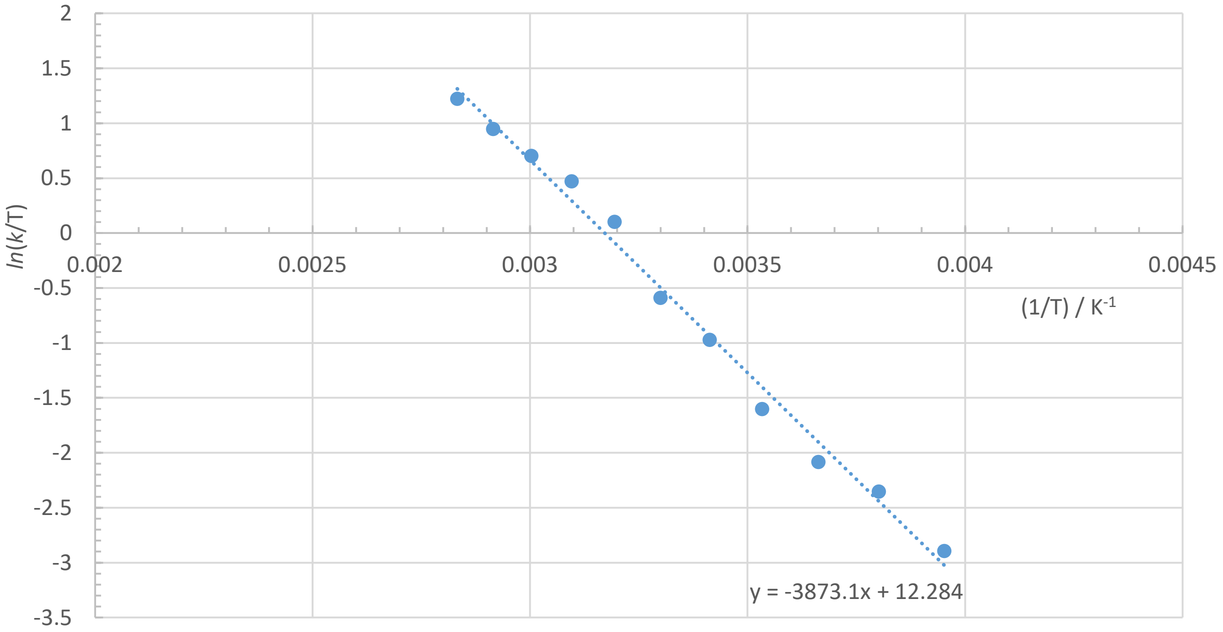

Figure S2: Eyring plot from the fitted VT-NMR data for foldamer **14**

Applying the values from the Eyring plot (Figure S2) to Eqn 1 gives:

Δ*H*‡ = +32.2 kJ/mol

Δ*S*‡ = −95.4 J/mol/K

At 298 K, Δ*G*‡ = +60.6 kJ/mol.

6. Catalysis

6.1 Procedure for alkyne hydrosilylation

In a dry NMR tube containing 0.3 mL of $CDCl_3$ pre-dried over K_2CO_3, 0.077 mmol of alkyne was added, followed with 0.085 mmol (1.1 equiv) of the silane. Each rhodium NHC catalyst (1 mol %) was dissolved in 0.1 mL of dry $CDCl_3$, then added to the mixture. The NMR tube was sealed and shaken. ^{1}H NMR spectra were recorded over time to monitor the reaction.

Hydrosilylation of 1-hexyne: 1-hexyne (0.01 mL, 0.077 mmol), dimethylphenylsilane Me_2PhSiH (0.013 mL, 0.085 mmol).

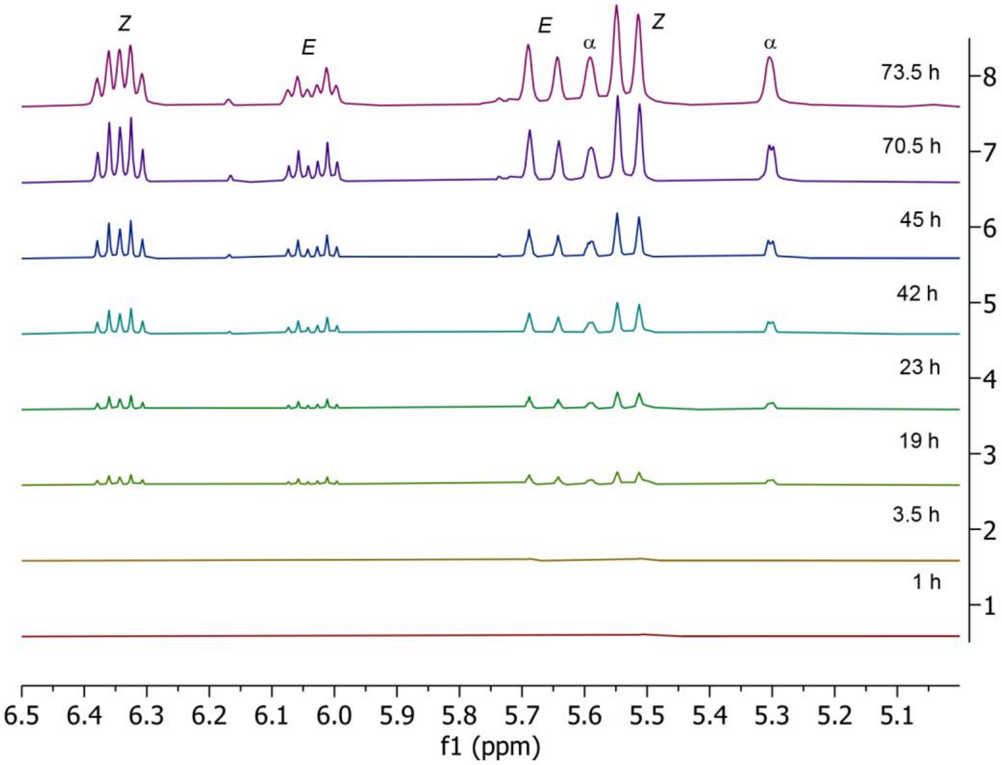

Figure S3: a) Expansion of ^{1}H NMR spectra showing the alkene resonances for the *Z*- (*cis*-), *E*- (*trans*-) and α-isomers of the silyl alkenes forming upon the hydrosilylation of 1-hexyne with catalysis by **2** under standard conditions.

Hydrosilylation of phenylacetylene: phenylacetylene (0.08 mL, 0.077 mmol), dimethylphenylsilane Me_2PhSiH (0.013 mL, 0.085 mmol).

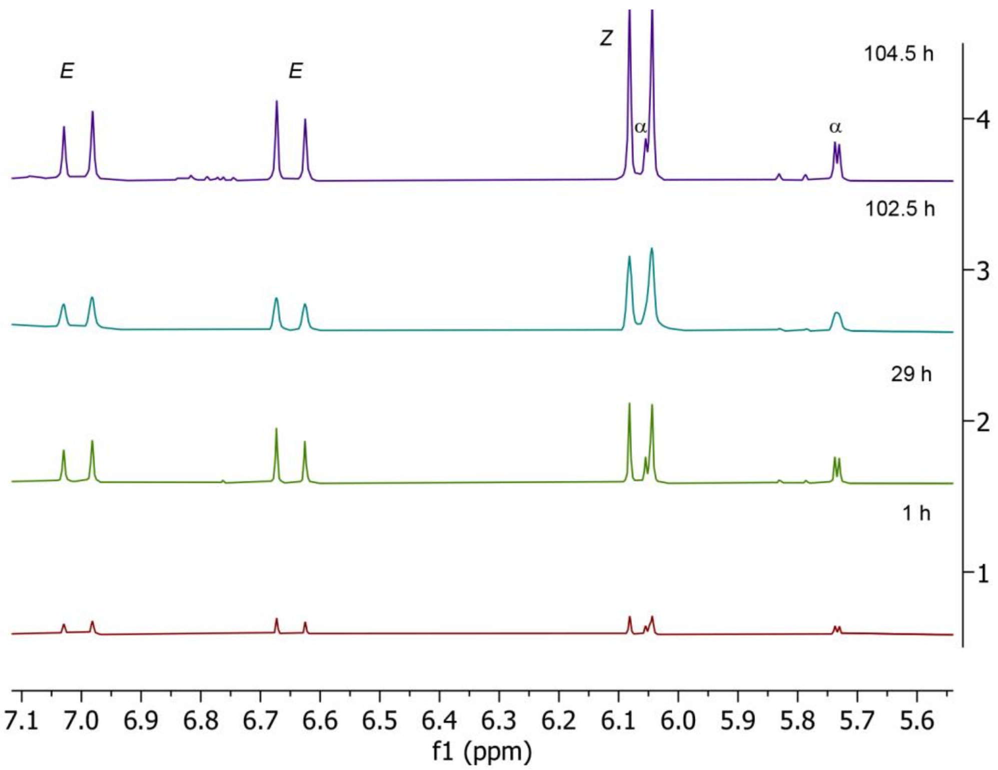

Figure S4: a) Expansion of ^{1}H NMR spectra showing the alkene resonances for the *Z*- (*cis*-), *E*- (*trans*-) and α-isomers of the silyl alkenes forming upon the hydrosilylation of phenylacetylene with catalysis by **14** under standard conditions. The other alkene proton for the *Z* isomer is underneath the aromatic signals.

Alkyne	Catalyst	Time / h	Conversion / %	Z / %	E / %	α / %	2-ene / %
1-hexyne	**2**	227	49	50	31	23	0
1-hexyne	**14**	126	100	18	41	12	30
1-hexyne	**3**	42.5	64	81	10	7	2
1-hexyne	**19**	20	100	66	20	8	6
phenylacetylene	**2**	194.5	71	38	44	18	-
phenylacetylene	**14**	126	96	59	28	13	-
phenylacetylene	**3**	44.5	74	83	14	3	-
phenylacetylene	**19**	45.5	91	78	17	5	-
phenylacetylene	**18**	216	100	0	88	12	-

Table S1. Hydrosilylation of terminal alkynes with Aib-functionalized NHC rhodium(I) complexes. Reactant alkynes 77 µmol, 1 eq.; Me_2PhSiH 85 µmol, 1.1 eq.; Rh cat. 1 mol % in solvent $CDCl_3$ 0.4 mL.

6.2 Procedure for carvone hydrosilylation

In a dry NMR tube, the rhodium NHC catalyst (1 mol %) was dissolved in 0.4 mL of CD_2Cl_2 (pre-dried over K_2CO_3). Diphenylsilane Ph_2SiH_2 (0.045 mL, 0.24 mmol, 1.8 equiv.) was added, followed with carvone (*R* or *S*) (0.02 mL, 0.1325 mmol, 1 equiv). The tube was sealed (some increase in pressure, ascribed to hydrogen buildup, the lid sealed with parafilm) and shaken. ^{1}H NMR spectra were recorded over time to monitor the reaction.

Shown below (Figure S5) is an example of a carvone hydrosilylation catalysed by **2**, including time course and analysis of the ^{1}H NMR spectra over time.

a) b)

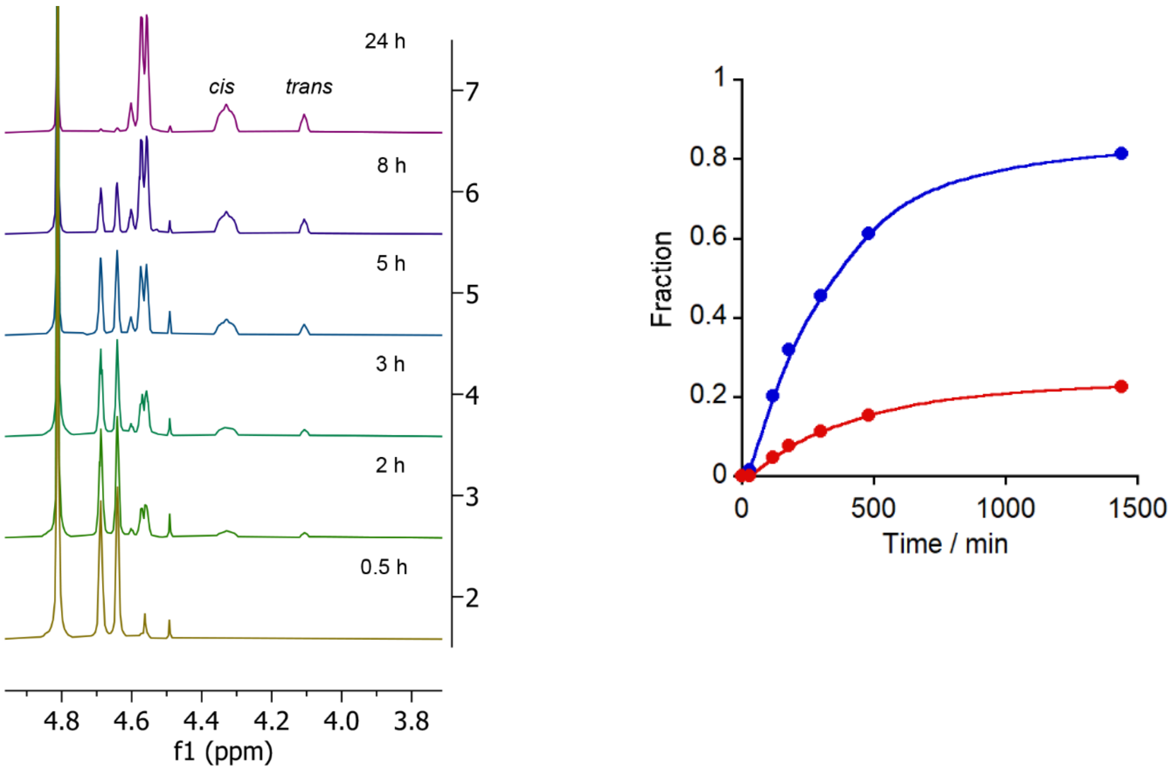

Figure S5: a) Expansion of ^{1}H NMR spectra showing the CH-OSiPh$_2$H proton resonances for the *cis* and *trans* isomers of the silyl ether of carveol, which had been formed by **2** catalysis under standard conditions. Spectra acquired *in situ* at 0.5, 2, 3, 5, 8 and 24 h. b) Plots showing the mol fraction of the *cis* (blue trace) and *trans* (red trace) silyl ethers over 24 h period. Curve fits are to guide the eye. The *cis*:*trans* ratio declined slightly between 2 h and 24 h, from 4.3 to 3.6.

Shown below are examples of the hydrosilyation of the enantiomers of carvone, each catalysed by **20**, showing the analysis of the ^{1}H NMR spectra. The *cis*:*trans* ratio is unchanged for each enantiomeric substrate, showing that the chirality in catalyst **20** does not affect the stereoselectivity of this reaction.

a)

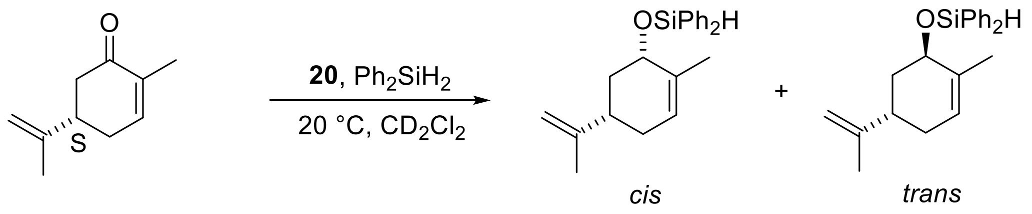

b)

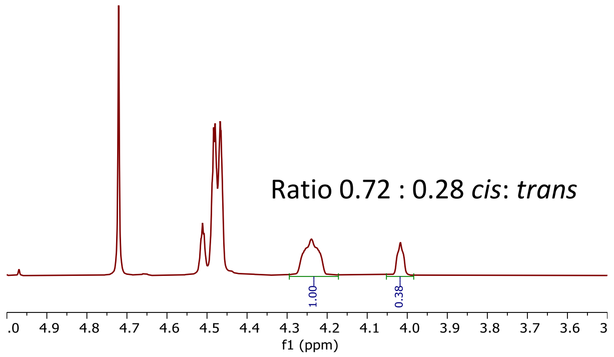

Figure S6: a) Hydrosilyation of *S*-carvone under standard conditions using chiral catalyst **20**. b) Expansion of ^{1}H NMR spectrum showing the CH-OSiPh$_2$H proton resonances for the *cis* and *trans* isomers of the silyl ether of carveol, which had been formed by **20** catalysis under standard conditions. Spectrum acquired *in situ* at 14 h.

a)

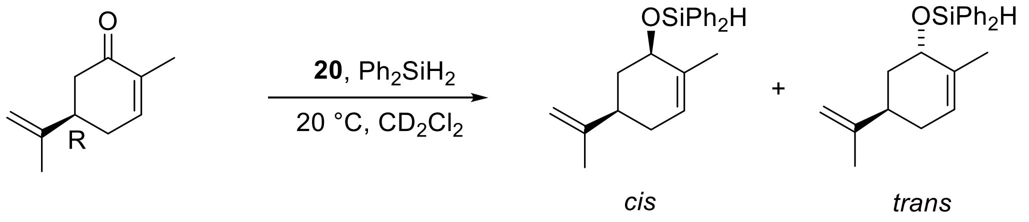

b)

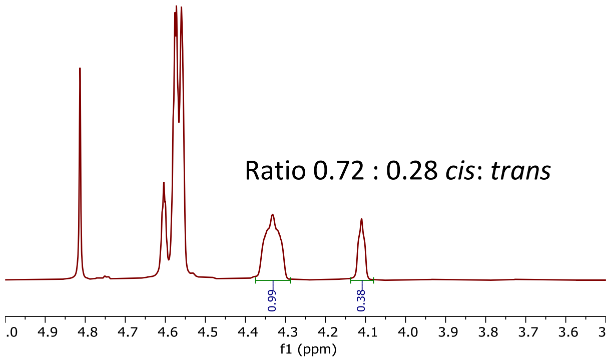

Figure S7: a) Hydrosilyation of *R*-carvone under standard conditions using chiral catalyst **20**. b) Expansion of ^{1}H NMR spectrum showing the CH-OSiPh$_2$H proton resonances for the *cis* and *trans* isomers of the silyl ether of carveol, which had been formed by **20** catalysis under standard conditions. Spectrum acquired *in situ* at 14 h.

Catalyst	Carvone isomer	[Carvone] / M	Additive (1 eq.)	Ratio *cis* : *trans*
[($N_3Aib_4(CH_2)_2$-NHC-Ph)Rh(Cl)(COD)] **2**	*S*	0.31	-	0.79 : 0.21
[($N_3Aib_4(CH_2)_2$-NHC-Ph)Rh(Cl)(COD)] **2**	*S*	0.31	$AgBF_4$	0.80 : 0.20
[($N_3Aib_4(CH_2)_2$-NHC-Ph)Rh(Cl)(COD)] **2**	*R*	0.6125	-	0.71 : 0.29
[($N_3Aib_4(CH_2)_2$-NHC-Ph)Rh(Cl)(COD)] **2**	*R*	0.31	-	0.74 : 0.26
[($N_3Aib_4(CH_2)_2$-NHC-Ph)Rh(Cl)(COD)] **2**	*R*	0.31	$AgBF_4$	0.82 : 0.18
[($N_3Aib_4(CH_2)_2$-NHC-Mes)Rh(Cl)(COD)] **3**	*R*	0.31	-	0.75 : 0.25
[($N_3Aib_4(CH_2)_2$-NHC-Mes)Rh(Cl)(COD)] **3**	*S*	0.31	-	0.75 : 0.25
[($N_3Aib_4(CH_2)_2$-NHC-Mes)Rh(Cl)(COD)] **3**	*S*	0.31	$AgBF_4$	0.81 : 0.19
[($N_3Aib_4(CH_2)_2$-NHC-Me)Rh(Cl)(COD)] **4**	*S*	0.6125	-	0.78 : 0.22
[($N_3Aib_4(CH_2)_2$-NHC-Ph)Rh(Cl)(NBD)] **8**	*S*	0.6125	-	0.78 : 0.22
[(Cbz-(L-αMeVal)$Aib_4(CH_2)_2$-NHC-Ph)Rh(Cl)(COD)] **14**	*S*	0.31	-	0.76 : 0.24
[(Cbz-(L-αMeVal)$Aib_4(CH_2)_2$-NHC-Ph)Rh(Cl)(COD)] **14**	*R*	0.31	-	0.75 : 0.25
[(Cbz-(L-αMeVal)$Aib_4(CH_2)_2$-NHC-Ph)Rh(Cl)(COD)] **14**	*S*	0.31	$AgBF_4$	0.78 : 0.22
[(Cbz-(L-αMeVal)$Aib_4(CH_2)_2$-NHC-Ph)Rh(Cl)(COD)] **14**	*S*	0.31	$AgBF_4$	0.70 : 0.30
[(Cbz-(L-αMeVal)$Aib_4(CH_2)_2$-NHC-Ph)Rh(Cl)(COD)] **14**	*R*	0.31	$AgBF_4$	0.76 : 0.24
[(Cbz-(L-αMeVal)$Aib_4(CH_2)_2$-NHC-Ph)Rh(Cl)(COD)] **14**	*R*	0.31	$AgBF_4$	0.69 : 0.31
[(Cbz-(L-αMeVal)$Aib_4(CH_2)_2$-NHC-Ph)Rh(Cl)(NBD)] **15**	*R*	0.6125	-	0.77 : 0.23
[(Cbz-(L-αMeVal)$Aib_4(CH_2)_2$-NHC-Ph)Rh(Cl)(NBD)] **15**	*S*	0.6125	-	0.75 : 0.25
[(Cbz-(L-αMeVal)$Aib_4(CH_2)_2$-NHC-Ph)Rh(Cl)(NBD)] **15**	*R*	0.6125	$AgBF_4$	0.79 : 0.21
[(Cbz-(L-αMeVal)$Aib_4(CH_2)_2$-NHC-Ph)Rh(Cl)(NBD)] **15**	*S*	0.6125	$AgBF_4$	0.81 : 0.19
[(Cbz-(L-αMeVal)$Aib_4(CH_2)_2$-NHC-Mes)Rh(Cl)(COD)] **16**	*S*	0.31	-	0.84 : 0.16
[(Cbz-(L-αMeVal)$Aib_4(CH_2)_2$-NHC-Mes)Rh(Cl)(COD)] **16**	*R*	0.31	-	0.79 : 0.21
[(Cbz-(L-αMeVal)$Aib_4(CH_2)_2$-NHC-Mes)Rh(Cl)(COD)] **16**	*R*	0.31	-	0.79 : 0.21
[(Cbz-(L-αMeVal)$_2Aib_4(CH_2)_2$-NHC-Mes)Rh(Cl)(COD)] **18**	*S*	0.31	-	0.79 : 0.21
[(Cbz-(L-αMeVal)$_2Aib_4(CH_2)_2$-NHC-Mes)Rh(Cl)(COD)] **18**	*S*	0.31	-	0.8 : 0.2
[(Cbz-(L-αMeVal)$_2Aib_4(CH_2)_2$-NHC-Mes)Rh(Cl)(COD)] **18**	*R*	0.31	-	0.77 : 0.23
[(Cbz-(L-αMeVal)$_2Aib_4(CH_2)_2$-NHC-Mes)Rh(Cl)(COD)] **18**	*R*	0.31	-	0.76 : 0.24
[(Cbz-(L-αMeVal)$Aib_5(CH_2)_2$-NHC-Mes)Rh(Cl)(COD)] **19**	*S*	0.31	-	0.77 : 0.23
[(Cbz-(L-αMeVal)$Aib_5(CH_2)_2$-NHC-Mes)Rh(Cl)(COD)] **19**	*S*	0.31	-	0.76 : 0.24
[(Cbz-(L-αMeVal)$Aib_5(CH_2)_2$-NHC-Mes)Rh(Cl)(COD)] **19**	*S*	0.31	-	0.73 : 0.27
[(Cbz-(L-αMeVal)$Aib_5(CH_2)_2$-NHC-Mes)Rh(Cl)(COD)] **19**	*S*	0.31	$AgBF_4$	0.81 : 0.19
[(Cbz-(L-αMeVal)$Aib_5(CH_2)_2$-NHC-Mes)Rh(Cl)(COD)] **19**	*R*	0.31	-	0.77 :0.23
[(Cbz-(L-αMeVal)$Aib_5(CH_2)_2$-NHC-Mes)Rh(Cl)(COD)] **19**	*R*	0.31	-	0.71 : 0.29
[(Cbz-(L-αMeVal)$Aib_5(CH_2)_2$-NHC-Mes)Rh(Cl)(COD)] **19**	*R*	0.31	-	0.72 : 0.28
[(Cbz-(L-αMeVal)$Aib_5(CH_2)_2$-NHC-Mes)Rh(Cl)(COD)] **19**	*R*	0.31	$AgBF_4$	0.81 : 0.19

Table S2. Hydrosilylation of *R*- or *S*- carvone with Aib-functionalized rhodium(I) NHC complexes under standard conditions in the presence or absence of $AgBF_4$ (1 eq., to remove chloride ligand).

7. Crystal data and structure refinement

7.1 Foldamer 2

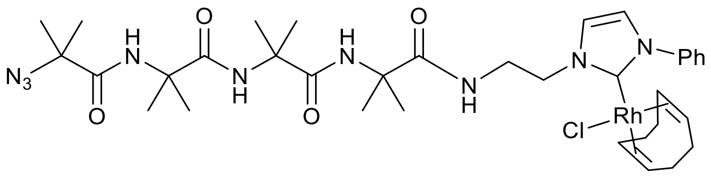

Single crystals suitable for X-ray diffraction analysis were grown by slow evaporation of an acetonitrile solution containing the foldamer. Data were collected on a dual source Rigaku FR-X rotating anode diffractometer using CuK_{α} wavelength radiation (λ = 1.54184) at a temperature of 100 K. Data for foldamer **2** were found to be twinned with 2 components. The data were reduced for each component using CrysAlisPro 1.71.40.14d obtaining a 0.2/0.8 proportion.Absorption correction was performed using empirical methods (SCALE3 ABSPACK) based upon symmetry-equivalent reflections combined with measurements at different azimuthal angles.[10] The structure was solved and refined against F^2 using Shelximplemented through Olex2.[11] Hydrogen atoms were placed in the calculated positions and assigned fixed thermal parameters. All atoms were refined anysotropically.

CCDC 2124905 contains the supplementary crystallographic data for this paper. These data can be obtained free of charge via www.ccdc.cam.ac.uk/conts/retrieving.html (or from the Cambridge Crystallographic Data Centre, 12 Union Road, Cambridge CB2 1EZ, UK; fax: (+44)1223-336-033; or deposit@ccdc.cam.ac.uk).

Table S3. Crystal data and structure refinement for foldamer **2**

Identification code	**2**
Empirical formula	$C_{38}H_{57}ClN_9O_4Rh$
Formula weight	841.26
Temperature/K	100
Crystal system	monoclinic
Space group	$P2_1/c$
a/Å	16.2287(3)
b/Å	13.8235(3)
c/Å	19.2693(4)
α/°	90
β/°	111.694(2)
γ/°	90
Volume/Å^3	4016.65(15)
Z	4
ρ_{calc}g/cm^3	1.3911
μ/mm^{-1}	4.466
F(000)	1766.7
Crystal size/mm^3	0.45 × 0.1 × 0.1
Radiation	Cu Kα (λ = 1.54184)
2Θ range for data collection/°	8.68 to 154.46
Index ranges	$-20 \le h \le 20$, $-17 \le k \le 16$, $-22 \le l \le 22$
Reflections collected	18994
Independent reflections	11649 [R_{int} = N/A, R_{sigma} = N/A]
Data/restraints/parameters	11649/0/488
Goodness-of-fit on F^2	1.105
Final R indexes [I>=2σ (I)]	R_1 = 0.0682, wR_2 = 0.2145
Final R indexes [all data]	R_1 = 0.0755, wR_2 = 0.2336
Largest diff. peak/hole / e Å^{-3}	2.74/-1.55

Table S4 Fractional Atomic Coordinates (×10^4) and Equivalent Isotropic Displacement Parameters (Å^2×10^3) for s5569l_twin1_hklf4. U_{eq} is defined as 1/3 of of the trace of the orthogonalised U_{IJ} tensor.

**Atom**	***x***	***y***	***z***	**U(eq)**
Rh01	3553.76(17)	4242.6(2)	5859.10(14)	24.12(15)
Cl02	2358.2(5)	4729.5(6)	6267.9(4)	22.4(2)
N004	4483(2)	6192(2)	6182.9(18)	26.2(7)
O005	2673.0(18)	7703(2)	3484.7(15)	28.7(6)
O006	945(2)	7838(3)	5169.3(18)	38.9(7)
O007	563.5(19)	8245(3)	2435.8(16)	33.5(7)
N008	3355(2)	6262(3)	5162.0(18)	27.4(7)
O009	2944(2)	9401(2)	4783.4(17)	34.3(7)
N00A	2424(2)	9103(3)	2840.4(19)	27.8(7)
N00B	1574(2)	9296(3)	3873(2)	28.2(7)
C00C	5546(3)	4917(4)	7992(2)	36.6(9)
N00D	1242(2)	8036(3)	1612.4(17)	26.4(7)

C00E	2396(2)	8135(3)	2881(2)	25.7(7)
N00F	1619(2)	7426(3)	4376.8(19)	28.1(7)
C00G	3806(3)	5644(3)	5715(2)	26.1(8)
C00H	3733(3)	7176(3)	5275(2)	30.0(8)
N00I	-904(3)	9098(4)	1408(2)	45.0(10)
C00J	4449(3)	7128(3)	5924(2)	30.6(8)
C00K	4742(3)	3839(3)	5730(2)	32.1(9)
C00L	4895(3)	5259(3)	7335(2)	30.9(8)
C00M	2458(3)	9429(3)	4122(2)	28.8(8)
C00N	1062(3)	9089(3)	4349(2)	29.8(8)
C00O	2043(2)	7574(3)	2143(2)	26.2(7)
C00P	2837(3)	9698(3)	3517(2)	33.6(9)
C00Q	6429(3)	5162(4)	8170(3)	41.2(10)
C00R	544(3)	8261(3)	1794(2)	27.4(8)
C00S	1257(3)	9831(4)	4981(3)	39.4(10)
C00T	-769(3)	7548(4)	798(3)	43.0(11)
C00U	1830(3)	6528(3)	2284(2)	32.1(8)
C00V	4016(3)	3782(3)	5033(2)	29.3(8)
C00X	5164(3)	2942(4)	6176(3)	41.6(10)
C00Y	1226(2)	8058(3)	4680(2)	27.8(8)
C00Z	6670(3)	5746(4)	7696(3)	41.6(12)
C010	3141(3)	2707(3)	5810(3)	35.3(9)
C011	2761(3)	7583(3)	1800(2)	30.5(8)
C012	5145(3)	5837(3)	6862(2)	27.5(8)
C014	3544(3)	2858(4)	4666(3)	37.3(9)
C015	1708(3)	6402(3)	4568(2)	31.7(8)
N016	-1240(3)	8697(4)	1795(2)	52.2(12)
C017	-319(3)	8506(4)	1131(2)	32.4(9)
C018	82(3)	9159(4)	3846(3)	38.1(11)
C019	2552(3)	5971(3)	4528(2)	29.3(8)
C01A	-173(3)	9134(4)	545(3)	37.5(10)
C01B	3775(3)	2890(3)	6496(2)	34.3(9)
C01C	6029(3)	6087(4)	7030(2)	34.9(9)
N01F	-1623(3)	8401(6)	2147(3)	70.7(18)
C01G	4754(3)	2649(4)	6741(3)	41.1(10)
C01J	3325(3)	2159(4)	5202(3)	38.3(10)
C01N	765(6)	3222(7)	6491(5)	85(3)
C01O	2591(5)	10761(4)	3291(3)	48.6(13)
C6	454(6)	3653(9)	7029(6)	93(3)
N10	227(7)	4041(10)	7478(6)	124(4)
C1	3844(3)	9580(5)	3832(3)	48.9(13)

Table S5 Anisotropic Displacement Parameters (Å^2×10^3) for s5569l_twin1_hklf4. The Anisotropic displacement factor exponent takes the form: $-2\pi^2[h^2a^{*2}U_{11}+2hka^*b^*U_{12}+\ldots]$.

**Atom**	**U_{11}**	**U_{22}**	**U_{33}**	**U_{12}**	**U_{13}**	**U_{23}**
Rh01	23.23(19)	25.8(2)	23.1(2)	1.95(9)	8.29(12)	0.02(9)
Cl02	22.0(4)	30.0(4)	17.5(4)	6.5(3)	10.1(3)	-3.5(3)
N004	24.3(14)	26.6(17)	27.2(15)	0.6(12)	8.9(12)	-0.9(12)
O005	27.6(13)	34.9(16)	25.5(13)	7.7(11)	12.0(10)	3.7(11)
O006	43.4(17)	48.0(19)	34.2(16)	9.2(14)	24.6(13)	9.6(13)
O007	30.1(14)	49.5(19)	23.1(13)	6.2(13)	12.3(10)	-1.8(12)
N008	26.2(15)	30.0(18)	25.4(15)	4.9(13)	8.8(12)	4.2(13)
O009	35.0(15)	38.7(16)	25.4(14)	2.0(13)	6.6(11)	-4.5(12)
N00A	32.4(17)	28.8(17)	23.7(16)	0.5(13)	12.0(13)	-0.2(12)
N00B	33.2(18)	31.5(19)	21.4(16)	7.9(13)	11.9(13)	3.0(11)
C00C	40(2)	36(2)	29(2)	4.7(18)	7.2(16)	1.2(16)
N00D	26.7(15)	33.7(18)	20.3(14)	3.8(13)	10.4(11)	1.6(12)
C00E	23.6(16)	31(2)	25.3(17)	1.5(15)	12.4(13)	-3.0(14)
N00F	27.6(15)	31.5(18)	26.9(16)	4.4(13)	12.1(12)	4.9(13)
C00G	24.5(17)	27.7(19)	27.3(18)	1.9(14)	11.0(14)	-5.7(14)
C00H	27.5(18)	28(2)	35(2)	4.0(15)	11.4(15)	1.9(15)
N00I	36(2)	61(3)	40(2)	19.1(19)	16.2(17)	4.6(18)
C00J	29.4(18)	31(2)	33(2)	1.9(16)	13.0(15)	0.1(16)
C00K	27.9(18)	39(2)	34(2)	5.7(17)	15.8(15)	4.6(17)
C00L	27.4(18)	33(2)	30.2(19)	1.4(16)	8.1(14)	-3.4(16)
C00M	35(2)	25.6(18)	26.6(19)	4.9(16)	12.6(15)	-0.8(15)
C00N	31(2)	38(2)	24.0(18)	8.7(17)	14.1(15)	1.9(15)
C00O	23.2(16)	31(2)	25.7(18)	2.5(15)	10.5(13)	0.8(14)
C00P	43(2)	32(2)	29(2)	-7.2(18)	17.2(16)	-5.8(16)
C00Q	34(2)	46(3)	34(2)	10.9(19)	1.6(17)	-2.9(19)
C00R	25.1(17)	32(2)	26.4(19)	1.2(15)	11.0(14)	0.5(15)
C00S	48(2)	41(3)	34(2)	7(2)	21.4(18)	-6.3(18)
C00T	34(2)	52(3)	39(2)	-7(2)	9.0(17)	-2(2)
C00U	35(2)	30(2)	31.1(19)	-0.3(16)	12.4(15)	-0.4(15)
C00V	32.0(19)	31(2)	29.4(19)	1.1(16)	16.7(15)	-2.0(15)
C00X	37(2)	41(3)	48(3)	12(2)	16.8(19)	7(2)
C00Y	23.2(16)	38(2)	20.7(16)	4.9(15)	6.8(12)	3.2(14)
C00Z	27(2)	54(3)	37(2)	4.1(18)	3.9(17)	-7.0(19)
C010	35(2)	36(2)	38(2)	-5.0(18)	17.4(17)	-1.4(17)
C011	29.3(18)	39(2)	28.4(19)	2.8(16)	16.6(14)	-3.1(16)
C012	26.6(19)	30(2)	24.7(19)	5.1(14)	8.0(15)	-3.8(14)
C014	43(2)	38(2)	35(2)	-3.1(19)	18.7(17)	-11.1(18)
C015	27.4(18)	33(2)	32.5(19)	1.0(16)	8.4(14)	5.8(16)
N016	32.4(19)	89(4)	36(2)	17(2)	13.4(16)	3(2)
C017	25.3(17)	44(2)	28.0(19)	6.9(17)	10.3(14)	0.7(16)
C018	31(2)	56(3)	29(2)	14.9(18)	13.4(17)	4.4(17)
C019	31.6(19)	26.8(19)	25.6(18)	4.2(16)	6.0(14)	1.6(15)
C01A	36(2)	43(3)	32(2)	7.4(18)	10.9(17)	8.2(17)
C01B	43(2)	27(2)	35(2)	4.1(17)	16.2(17)	7.8(16)

C01C	27.9(19)	44(3)	34(2)	2.7(18)	12.0(15)	-2.7(18)
N01F	41(2)	130(6)	46(3)	17(3)	22(2)	5(3)
C01G	43(2)	34(2)	40(2)	7.7(19)	9.1(18)	10.0(18)
C01J	43(2)	33(2)	43(2)	-7.4(19)	20.6(19)	-9.6(19)
C01N	70(5)	77(5)	83(5)	-22(4)	-1(4)	11(4)
C01O	80(4)	33(3)	41(3)	-9(2)	32(3)	-4.7(18)
C6	64(5)	104(7)	86(6)	-23(5)	0(4)	26(5)
N10	96(7)	179(12)	99(7)	-1(7)	40(6)	-1(7)
C1	39(2)	72(4)	39(2)	-16(2)	18.2(19)	-20(2)

Table S6: Bond Lengths for s5569l_twin1_hklf4.

Atom	Atom	Length/Å	Atom	Atom	Length/Å
Rh01	Cl02	2.4430(8)	N00I	C017	1.493(6)
Rh01	C00G	2.020(4)	C00K	C00V	1.424(6)
Rh01	C00K	2.108(4)	C00K	C00X	1.520(6)
Rh01	C00V	2.094(4)	C00L	C012	1.380(6)
Rh01	C010	2.217(5)	C00M	C00P	1.552(6)
Rh01	C01B	2.192(4)	C00N	C00S	1.533(6)
N004	C00G	1.364(5)	C00N	C00Y	1.543(6)
N004	C00J	1.381(6)	C00N	C018	1.529(6)
N004	C012	1.438(5)	C00O	C00U	1.534(6)
O005	C00E	1.235(5)	C00O	C011	1.539(5)
O006	C00Y	1.228(5)	C00P	C01O	1.542(7)
O007	C00R	1.226(5)	C00P	C1	1.527(7)
N008	C00G	1.351(5)	C00Q	C00Z	1.379(8)
N008	C00H	1.385(6)	C00R	C017	1.544(5)
N008	C019	1.475(5)	C00T	C017	1.535(7)
O009	C00M	1.226(5)	C00V	C014	1.523(6)
N00A	C00E	1.342(6)	C00X	C01G	1.524(7)
N00A	C00P	1.476(5)	C00Z	C01C	1.401(6)
N00B	C00M	1.347(6)	C010	C01B	1.365(6)
N00B	C00N	1.474(6)	C010	C01J	1.515(6)
C00C	C00L	1.398(6)	C012	C01C	1.394(6)
C00C	C00Q	1.386(7)	C014	C01J	1.549(7)
N00D	C00O	1.470(5)	C015	C019	1.520(6)
N00D	C00R	1.340(5)	N016	N01F	1.152(7)
C00E	C00O	1.533(5)	C017	C01A	1.510(6)
N00F	C00Y	1.337(5)	C01B	C01G	1.519(7)
N00F	C015	1.456(6)	C01N	C6	1.440(17)
C00H	C00J	1.359(6)	C6	N10	1.185(16)
N00I	N016	1.209(7)			

Table S7: Bond Angles for s5569l_twin1_hklf4.

Atom	Atom	Atom	Angle/˚	Atom	Atom	Atom	Angle/˚
C00G	Rh01	Cl02	90.35(11)	C00E	C00O	N00D	110.9(3)
C00K	Rh01	Cl02	168.77(12)	C00U	C00O	N00D	109.5(3)
C00K	Rh01	C00G	89.79(17)	C00U	C00O	C00E	110.1(3)
C00V	Rh01	Cl02	151.55(11)	C011	C00O	N00D	108.2(3)
C00V	Rh01	C00G	92.23(16)	C011	C00O	C00E	108.2(3)
C00V	Rh01	C00K	39.63(15)	C011	C00O	C00U	109.9(3)
C010	Rh01	Cl02	90.81(12)	C00M	C00P	N00A	110.7(3)
C010	Rh01	C00G	170.21(16)	C01O	C00P	N00A	107.2(4)
C010	Rh01	C00K	90.95(17)	C01O	C00P	C00M	107.8(4)
C010	Rh01	C00V	82.17(17)	C1	C00P	N00A	111.3(4)
C01B	Rh01	Cl02	92.24(12)	C1	C00P	C00M	109.8(4)
C01B	Rh01	C00G	153.54(17)	C1	C00P	C01O	110.0(5)
C01B	Rh01	C00K	82.71(17)	C00Z	C00Q	C00C	119.9(4)
C01B	Rh01	C00V	97.82(17)	N00D	C00R	O007	123.4(3)
C01B	Rh01	C010	36.07(17)	C017	C00R	O007	121.0(3)
C00J	N004	C00G	111.5(3)	C017	C00R	N00D	115.5(3)
C012	N004	C00G	124.0(4)	C00K	C00V	Rh01	70.7(2)
C012	N004	C00J	124.4(3)	C014	C00V	Rh01	110.4(3)
C00H	N008	C00G	111.9(3)	C014	C00V	C00K	125.7(4)
C019	N008	C00G	122.4(4)	C01G	C00X	C00K	113.3(4)
C019	N008	C00H	125.7(3)	N00F	C00Y	O006	123.1(4)
C00P	N00A	C00E	121.2(3)	C00N	C00Y	O006	119.4(4)
C00N	N00B	C00M	125.1(4)	C00N	C00Y	N00F	117.4(3)
C00Q	C00C	C00L	120.5(5)	C01C	C00Z	C00Q	120.5(4)
C00R	N00D	C00O	122.3(3)	C01B	C010	Rh01	71.0(3)
N00A	C00E	O005	121.9(4)	C01J	C010	Rh01	111.7(3)
C00O	C00E	O005	120.7(4)	C01J	C010	C01B	123.3(4)
C00O	C00E	N00A	117.3(3)	C00L	C012	N004	119.7(4)
C015	N00F	C00Y	123.0(3)	C01C	C012	N004	119.0(4)
N004	C00G	Rh01	126.6(3)	C01C	C012	C00L	121.3(4)
N008	C00G	Rh01	129.6(3)	C01J	C014	C00V	114.4(4)
N008	C00G	N004	103.8(3)	C019	C015	N00F	112.0(3)
C00J	C00H	N008	106.2(4)	N01F	N016	N00I	172.5(6)
C017	N00I	N016	117.5(5)	C00R	C017	N00I	109.1(3)
C00H	C00J	N004	106.5(4)	C00T	C017	N00I	110.8(4)
C00V	C00K	Rh01	69.7(2)	C00T	C017	C00R	107.6(4)
C00X	C00K	Rh01	113.0(3)	C01A	C017	N00I	104.4(4)
C00X	C00K	C00V	122.0(4)	C01A	C017	C00R	113.5(4)
C012	C00L	C00C	119.0(4)	C01A	C017	C00T	111.4(4)
N00B	C00M	O009	123.9(4)	C015	C019	N008	112.8(3)
C00P	C00M	O009	120.5(4)	C010	C01B	Rh01	73.0(3)
C00P	C00M	N00B	115.5(4)	C01G	C01B	Rh01	107.2(3)
C00S	C00N	N00B	111.5(4)	C01G	C01B	C010	125.9(4)
C00Y	C00N	N00B	112.7(3)	C012	C01C	C00Z	118.8(5)
C00Y	C00N	C00S	109.6(3)	C01B	C01G	C00X	113.8(4)

C018	C00N	N00B	106.7(4)	C014	C01J	C010	111.3(4)
C018	C00N	C00S	108.7(4)	N10	C6	C01N	176.9(11)
C018	C00N	C00Y	107.5(4)				

Table S9: Hydrogen Atom Coordinates (Å×10[4]) and Isotropic Displacement Parameters (Å[2]×10[3]) for s5569l_twin1_hklf4.

**Atom**	***x***	***y***	***z***	**U(eq)**
H00A	2198(2)	9382(3)	2412.4(19)	33.4(8)
H00B	1285(2)	9335(3)	3399(2)	33.8(9)
H00C	5386(3)	4522(4)	8312(2)	43.9(11)
H00D	1221(2)	8167(3)	1169.8(17)	31.7(8)
H00F	1828(2)	7633(3)	4054.3(19)	33.7(8)
H00H	3536(3)	7713(3)	4968(2)	36.0(10)
H00J	4839(3)	7628(3)	6150(2)	36.7(10)
H00K	5154(3)	4373(3)	5770(2)	38.6(10)
H00L	4302(3)	5100(3)	7218(2)	37.1(10)
H00Q	6859(3)	4932(4)	8608(3)	49.5(13)
H00e	1876(6)	9810(20)	5288(14)	59.1(15)
H00g	1100(30)	10466(5)	4771(3)	59.1(15)
H00i	910(20)	9678(18)	5278(14)	59.1(15)
H00m	-910(30)	7194(15)	1170(8)	64.6(16)
H00n	-1306(16)	7678(4)	380(16)	64.6(16)
H00o	-376(12)	7172(15)	640(20)	64.6(16)
H00p	1600(20)	6188(8)	1818(3)	48.2(12)
H00r	2361(5)	6214(9)	2606(17)	48.2(12)
H00s	1399(18)	6529(3)	2517(19)	48.2(12)
H00V	4016(3)	4289(3)	4678(2)	35.1(10)
H00t	5104(3)	2409(4)	5833(3)	50.0(12)
H00u	5792(3)	3060(4)	6438(3)	50.0(12)
H00Z	7262(3)	5916(4)	7820(3)	49.9(14)
H010	2544(3)	2611(3)	5812(3)	42.3(11)
H01a	3296(8)	7300(20)	2145(9)	45.8(12)
H01b	2560(10)	7220(20)	1344(11)	45.8(12)
H01c	2876(18)	8238(4)	1696(19)	45.8(12)
H01d	2997(3)	3031(4)	4262(3)	44.7(11)
H01e	3914(3)	2520(4)	4448(3)	44.7(11)
H01f	1713(3)	6320(3)	5069(2)	38.1(10)
H01g	1200(3)	6055(3)	4227(2)	38.1(10)
H01h	-277(3)	8990(30)	4126(7)	57.1(16)
H01i	-52(7)	9810(8)	3665(19)	57.1(16)
H01j	-39(7)	8730(20)	3431(13)	57.1(16)
H01k	2609(3)	6175(3)	4066(2)	35.1(9)
H01l	2505(3)	5272(3)	4518(2)	35.1(9)
H01m	-735(3)	9300(30)	171(13)	56.3(15)
H01n	140(20)	9711(14)	774(5)	56.3(15)

H01o	170(20)	8784(11)	316(16)	56.3(15)
H01p	3551(3)	2897(3)	6904(2)	41.2(11)
H01q	6192(3)	6473(4)	6706(2)	41.9(11)
H01r	4831(3)	1958(4)	6827(3)	49.3(12)
H01s	5072(3)	2973(4)	7211(3)	49.3(12)
H01t	3819(3)	1721(4)	5428(3)	46.0(12)
H01u	2809(3)	1775(4)	4920(3)	46.0(12)
H01y	277(11)	2920(50)	6100(20)	128(4)
H	1020(50)	3714(11)	6280(30)	128(4)
Ha	1210(40)	2740(40)	6732(10)	128(4)
H01v	1959(6)	10838(8)	3130(30)	73(2)
H01w	2880(30)	11173(5)	3714(8)	73(2)
H01x	2780(30)	10933(11)	2893(19)	73(2)
H1a	4093(4)	9960(30)	4278(16)	73(2)
H1b	3992(3)	8912(7)	3950(30)	73(2)
H1c	4080(5)	9790(30)	3470(11)	73(2)

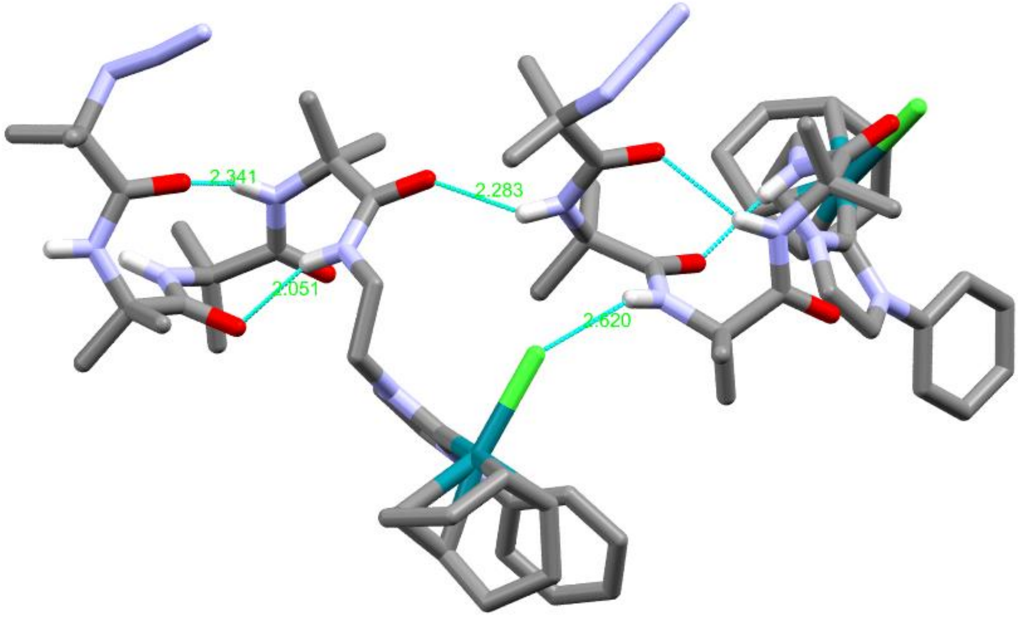

Figure S8: Solid state structure of **2** showing that the chloride is intermolecularly hydrogen bonded to the NH of the third Aib of a neighbouring foldamer. Intramolecular hydrogen bonds also shown. C atoms are shown in grey, N in light blue, O in red, Cl in green and Rh in teal. Some H atoms have been removed and acetonitrile of solvation not shown for clarity.

8. References

[1]. J. Clayden, A. Castellanos, J. Solà, G. A. Morris, *Angew. Chem. Int. Ed.*, **2009**, *48*, 5962–5965.

[2]. A.D. Peters, S. Borsley, F. della Sala, D. F. Cairns-Gibson, M. Leonidou, J. Clayden, G. F. S. Whitehead, I. J. Vitorica-Yrezabal, E. Takano, J. Burthem, S. L. Cockroft, S. J. Webb *Chem. Sci.*, **2020**, *11*, 7023.

[3]. S. J. Pike, J. Raftery, S. J. Webb, J. Clayden, *Org. Biomol. Chem*. **2014**, *12*, 4124

[4]. S. J. Pike, V. Diemer, J. Raftery, S. J. Webb, J. Clayden, *Chem. Eur. J.* **2014**, *20*, 15981-15990.

[5]. S. J. Pike, J. E. Jones, J. Raftery, J. Clayden, S. J. Webb, *Org. Biomol. Chem.*, **2015**, *13*, 9580-9584.

[6]. L. Byrne, J. Solà, T. Boddaert, T. Marcelli, R. W. Adams, G. A. Morris, J. Clayden, *Angew. Chem. Int. Ed.*, **2014**, *53*, 151-155.

[7]. A. Szadkowska , E. Zaorska, S. Staszko, R. Pawłowski, D. Trzybiński, K. Woźniak, *Eur. J. Org. Chem*. **2017**, 4074–4084.

[8]. D. Toummini, A. Tlili, J. Berges, F. Ouazzani, M. Taillefer, *Chem. Eur. J*. **2014**, *20*, 14619-14623.

[9]. P. Sharma, S. D. Park, K. T. Park, S. C. Nam, S. K. Jeong, Y. I. Yoon, I. H. Baek, *Chem. Eng. J*. **2012**, *193–194*, 267–275.

[10]. (a) G. Winter, *J. Appl. Cryst*. **2010**, *43*, 186-190. (b) Bruker 2001, SADABS 2008/1. Bruker AXS Inc., Madison, Wisconsin, USA.

[11]. (a) G. M. Sheldrick, *Acta Cryst*. **2015**, *A71*, 3-8. (b) G. M. Sheldrick, *Acta Cryst*. **2015**, *C71*, 3-8. (c) O. V. Dolomanov, L. J. Bourhis, R. J. Gildea, J. A. K. Howard, H. Puschmann, *J. Appl. Cryst*. **2009**, *42*, 339-341.
